# Sb- and Bi-Based Salphen Capsules: Pnictogen Bonding, Macrocycle Formation, and ON/OFF Catalysis

**DOI:** 10.1021/acs.inorgchem.6c03201

**Published:** 2026-09-29

**Authors:** Lara Garriel-Muñoz, Héctor Barbero, Álvaro García-Romero, Raúl García-Rodríguez

**Affiliations:** GIR MIOMeT-IU Cinquima-Química Inorgánica Facultad de Ciencias, 16782 Universidad de Valladolid, Campus Miguel Delibes, 47011 Valladolid, Spain

## Abstract

In this work, we explore the use of heavier tris­(3-pyridyl) pnictogen linkers E­(3-py)_3_ (E = Sb, Bi) with metallosalphen building blocks for the construction of supramolecular capsules and macrocycles. The incorporation of these heavier pnictogen centers has a pronounced influence on both self-assembly and reactivity. Depending on the substitution pattern of the salphen and the nature of the metal center (M = Zn, Mg), 1D coordination polymers, discrete 1:3 supramolecular capsule-like assemblies or hierarchically assembled multicapsule macrocycles can be formed. Combining Bi­(3-py)_3_ with unsubstituted Zn-salphen gives an unprecedented polymeric architecture supported by synergistic pyridyl coordination and intermolecular Bi···O pnictogen bonding (PnB). In contrast, Zn salphens with bulky substituents suppress PnB-mediated aggregation and favor capsule formation. Replacing Zn­(II) with Mg­(II) in the salphen unit redirects the assembly toward heterobimetallic macrocycles {[E­(3-py)_3_]_6_·(MgSal-^*t*^Bu)_10_} derived from capsule-like subunits. Finally, we show that encapsulation of Sb and Bi within Zn-salphen capsules modulates Sb- and Bi-mediated catalytic oxidation of α-hydroxyketones and organic diols, and the reversible encapsulation of Sb­(3-py)_3_ enables supramolecular ON/OFF control of the catalysis.

## Graphical abstract

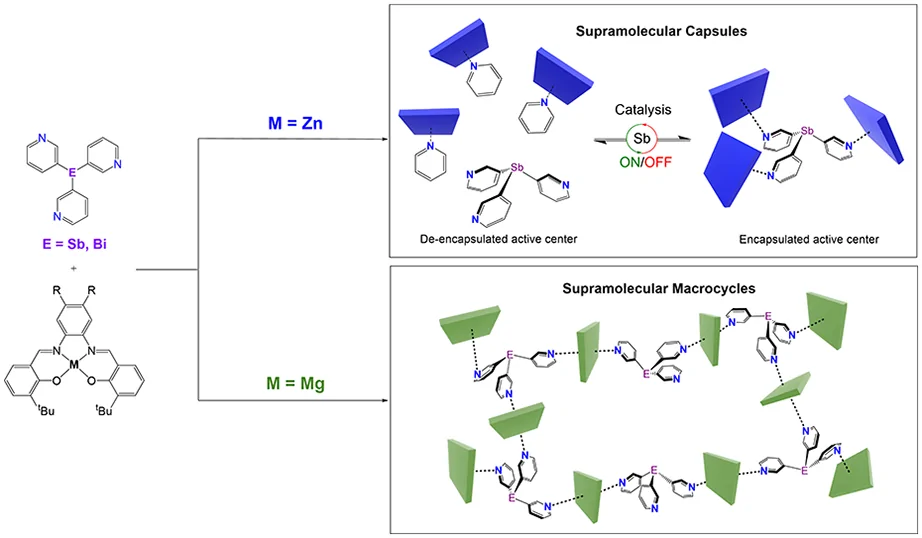

## Introduction

The exquisite selectivity and reactivity of enzymes have inspired the design of molecular architectures that mimic enzyme binding pockets, leading to the development of capsule-like molecules^1,2^ with applications in areas such as sensing,^3,4^ gas storage,^5–7^ drug delivery,^8–11^ and the development of molecular and porous nanoreactors for stoichiometric and catalytic transformations.^12–15^ Confinement within the cavities of these artificial systems can give rise to reactivity and selectivity patterns not accessible by traditional means, as well as the stabilization of reactive species.^16–21^ More recently, efforts have been focused on developing sophisticated artificial switchable catalysts that can be turned on or off in response to external stimuli, reminiscent of the enzymatic regulation of catalytic activity.^18,22–25^ However, these systems remain far from achieving the level of control exhibited by enzymes, and a major limitation is the difficulty of reaching a fully inactive (OFF) state.^26–28^

One important family of supramolecular capsules is those obtained via the coordination of the phosphine P­(3-py)_3_ to Zn­(II)-based building blocks.^15,29,30^ Reek and collaborators developed the first example, which consisted of a tris­(3-pyridyl)­phosphine ligand coordinated to three zinc tetraphenylporphyrin (ZnTPPH) units, {[P­(3-py)_3_]·(ZnTPPH)_3_}.^31^ This “ligand-template” approach was later extended to substituted Zn-porphyrins and other Zn-based building blocks, as well as related P-based ligands (Figure 1a).^32–40^ The resulting supramolecular P-based capsules can encapsulate Rh- and Pd-based catalysts and achieve unprecedented selectivities in the hydroformylation of olefins.^41^ Importantly, it was shown that small variations in the capsule size and cavity have a pronounced impact on the reactivity and selectivity.^24,37,42,43^

**Figure 1:**
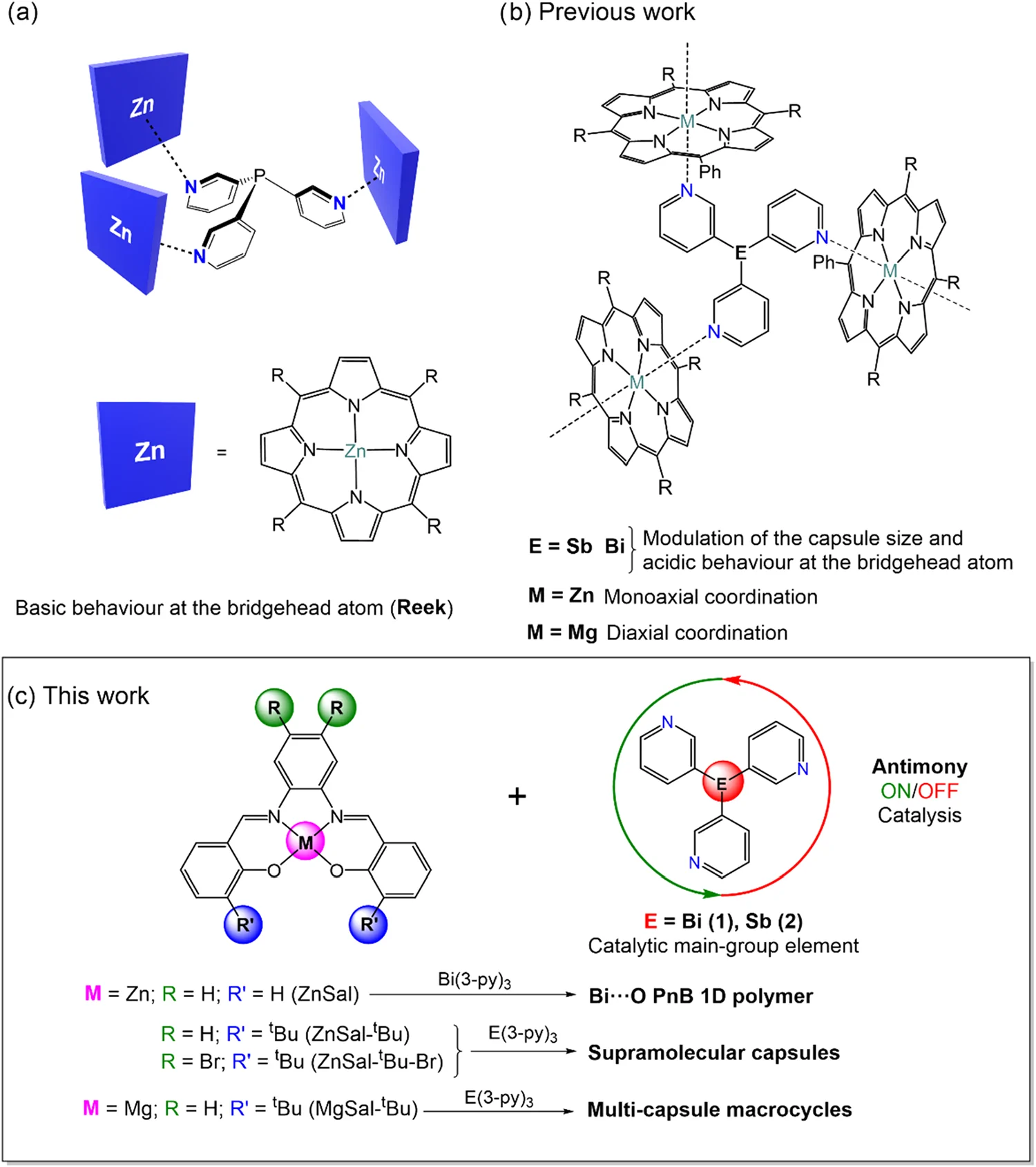
(a) Family of {[P­(3-py)_3_]·(ZnTPPR)_3_} capsules developed by Reek and co-workers (see refs 29–40 ). (b) Previous work on the formation of discrete and extended structures based on heavier group 15 bridgeheads (E = Sb, Bi; M = Zn, Mg; R = Ph, *p*-MeOC_6_H_4_, *p*-BrC_6_H_4_, or C_6_F_5_; see refs 69,70 ). (c) This work: heavier-based supramolecular capsules and macrocycles based on salphen fragments.

In contrast to the extensive use of lighter Group 15 based building blocks in supramolecular coordination chemistry, the incorporation of the heavier antimony and bismuth remains very limited. Sb- and Bi-based compounds present distinct structural and electronic features from their lighter group 15 counterparts, due to the increased size, higher Lewis acidity and greater ability to act as pnictogen bond (PnB) donors moving down the group.^44–48^ Recent studies have also revealed reactivity patterns reminiscent of transition metals due to their redox versatility.^49–53^ These features have spurred increasing interest in organoantimony and organobismuth compounds for applications in catalysis, anion recognition, and anion transport.^54–62^ However, their reactivity under supramolecular confinement remains largely unexplored. As part of our interest in main group chemistry involving heavier and more metallic ligands,^63–67^ we have recently prepared antimony- and bismuth-based tris-pyridyl ligands [E­(3-py)_3_; E = Bi (**1**), Sb (**2**)][Bibr ref68] that can form capsule-like assemblies by coordination to MTPP (M = Zn, Mg) porphyrins (Figure 1b).^69,70^ Changing the bridgehead (E) in the linker was shown to be an effective strategy to modulate the size of these types of porphyrin-based assemblies, and the encapsulation of E could be used to modulate catalytic activity in the oxidation of α-hydroxyketones and the oxidative cleavage of organic diols.^69^

However, these heavier-pnictogen porphyrin-based assemblies also exhibited important limitations. The increased size of the Sb and Bi bridgeheads generated larger, more weakly associated assemblies in solution,^69^ and their formation was easily disrupted by the substitution pattern of the porphyrin.^70^ This prompted us to consider alternative building blocks. Zn-metallosalen-type complexes represent a particularly attractive platform in this regard. While salen-type complexes are ubiquitous in catalysis,^71,72^ increasing attention has been devoted over the last two decades to their use in supramolecular chemistry.^73–77^ Compared with Zn­(II) porphyrins, Zn­(II) salphen complexes (salphen = *N*,*N*′-bis­(salicylidene)­imine-1,2-phenylenediamine dianion) are easier to prepare and modify, coordinate pyridyl donors more strongly, and possess a smaller, more conformationally adaptable framework.^37,74^ These features suggest that Zn­(II) salphens could enable heavier-pnictogen capsule-like assemblies that are both robust and dynamic, with their smaller and more adaptable framework providing a distinct microenvironment around the Sb or Bi center.

In this work, we explore the use of heavier Group 15 E­(3-py)_3_ ligands [E = Bi (**1**), Sb (**2**)] in combination with Zn- and Mg-salphen building blocks for the construction of supramolecular capsule-like assemblies (Figure 1c). The pronounced Lewis acidity of Bi is found to promote directional pnictogen-bonding (PnB) interactions that influence the self-assembly of these systems. While Zn-salphen building blocks give rise to discrete heavier-based capsules even in coordinating media, the use of Mg-salphen, an unexplored motif in supramolecular chemistry, promotes the integration of capsule-like units into macrocyclic assemblies. We also study the effect of the encapsulation of **1** and **2** within Zn-salphen capsules on the catalytic oxidative cleavage of organic diols and the oxidation of α-hydroxyketones. Notably, the dynamic behavior of these capsules enables the reversible ON/OFF switching of catalysis for the aerobic oxidation of α-hydroxyketones through disassembly around the Sb center and release of **2**.

## Results and Discussion

### Bi···O Pnictogen Bonding in the Formation of the 1D Polymer 1·ZnSal

We began our studies by evaluating the coordination of the heavier ligand Bi­(3-py)_3_ (**1**) with the unsubstituted zinc metallosalphen ZnSal. The addition of three equivalents of ZnSal to a solution of **1** in CHCl_3_ led to the formation of a highly insoluble yellow solid. Single-crystal X-ray diffraction analysis revealed that the structure of the product did not correspond to the targeted supramolecular capsule-like structure {[Bi­(3-py)_3_]·(ZnSal)_3_}, but instead a one-dimensional polymer, [Bi­(3-py)_3_·(ZnSal)]_*n*_ (**1**·ZnSal) (Figure 2). **1**·ZnSal consists of monomeric {Bi­(3-py)_3_·(ZnSal)} units linked in a 1D polymeric chain. In each unit, the bismuth ligand (**1**) coordinates to a zinc salphen complex through one *N*-donor atom, while the other two pyridine arms remain uncoordinated. Although the formation of **1**·ZnSal was observed irrespective of the stoichiometry used (**1**:ZnSal ratios of 1:3 or 1:1), higher yields (30% yield) were obtained using the required 1:1 stoichiometry.

**Figure 2:**
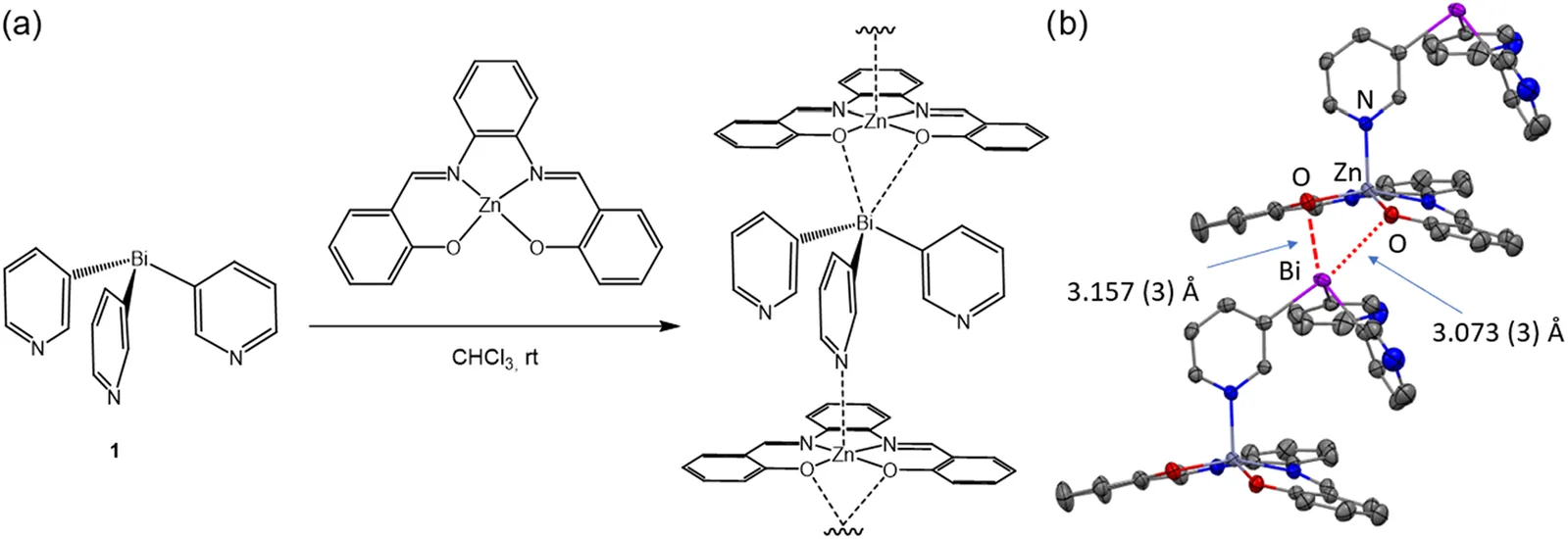
(a) Synthesis of the 1D polymeric complex [Bi­(3-py)_3_·(ZnSal)]_*n*_ (**1**·ZnSal). (b) X-ray structure of **1**·ZnSal showing two monomeric units [Bi­(3-py)_3_·(ZnSal)]. The 1D polymeric structure is constructed through Bi···O interactions between the monomeric units. Displacement ellipsoids shown at 50% probability. H atoms are omitted for clarity. Selected bond lengths (Å) and angles (°): **1**·Synthesis of the 1D ZnSal Bi–C_py_ range 2.257(5)–2.278(4); N_py_–Zn 2.088­(5); C_py_–Bi–C_py_ range 89.0(2)–93.9(2). Color key: C (gray), Zn (dark gray), N (blue), O (red), Bi (purple).

Zn­(II) salphen systems without significant steric hindrance have a pronounced tendency to self-associate through axial Zn···O coordination.^76,78^ This aggregation can be disrupted by the addition of strong monotopic nitrogen donors such as pyridine to generate the corresponding monomeric five-coordinate Zn-salphen complex via axial Zn···N coordination.^79–84^ This well-established behavior has been exploited to construct discrete supramolecular assemblies in a predictable manner by using polytopic Lewis bases to link multiple Zn-salphen units through Zn axial coordination. Compound **1**·ZnSal, however, deviates from this established behavior. In addition to the expected axial Zn–pyridine coordination, its assembly is directed by short, and presumably strong Bi···O pnictogen-bonding interactions (*d*_Bi–O_ = 3.073 (3) and 3.157 (3) Å, *cf*. sum of van der Waals radii ∑_VDW_(Bi···O) = 3.59 Å).^85^ These pnictogen bonds (PnB) reflect the Lewis acidity of Bi and propagate the monomeric {Bi­(3-py)_3_·(ZnSal)} into an infinite chain structure instead of the anticipated discrete supramolecular assembly. As a result of its polymeric structure, **1**·ZnSal is insoluble in most common organic solvents except DMSO, in which it breaks down into ZnSal and free ligand **1** in the expected 1:1 ratio as observed by ^1^H NMR spectroscopy (see SI; Figures S1, S2).

A closer look at the solid-state structure reveals that the shortest Bi···O PnB is located approximately along the extension of the Bi**–**C_py_ bond of the Zn-coordinated pyridyl arm. We wondered whether coordination of this pyridyl arm to ZnSal could remotely enhance the Lewis acidity of the Bi­(III) bridgehead. To evaluate this, the interaction between the O and the Bi atoms was studied computationally using a simplified model consisting of two {Bi­(3-py)_3_·(ZnSal)} monomeric units (see SI, Figure S65). The Quantum Theory of Atoms-In-Molecules (QTAIM) approach revealed bond critical points (BCPs) along the Bi···O axis (see SI, Figure S67). Additionally, NBO analysis identified several O → Bi donor–acceptor interactions between the lone pairs of the two oxygen atoms of the salphen and the unoccupied σ*­(Bi–C_py_) orbitals. Total attractive interactions of 4.91 and 7.09 kcal/mol were found, with the stronger interaction corresponding to the oxygen atom closest to bismuth and located approximately along the extension of the Zn-coordinated pyridyl arm (see SI, Table S13, Figure S66). This strongest O → Bi interaction was further studied using Electrostatic Surface Potential (ESP) maps. For this purpose, columnar assemblies comprising three {Bi­(3-py)_3_·(ZnSal)} monomeric units, i.e., three ligands and three ZnSal units, were built up in a stepwise manner starting from either a free Bi­(3-py)_3_ ligand or a free ZnSal unit (see SI for details, pages S52–S55). The key local maxima (*V*_max_) and minima (*V*_min_), which were assigned to the Bi σ-hole and the ZnSal oxygen lone pairs, respectively, were monitored to follow the changes in the charge depletion at the Bi σ-hole and charge concentration at the ZnSal oxygen lone pairs during assembly (Figure 3). The pyridyl N–Zn coordination of the initial Bi­(3-py)_3_ to a single ZnSal unit deepened the sigma hole on Bi by 6.31 kcal/mol (columnar assembly length = 1, Figure 3, blue), making it more electrophilic. Similarly, the charge concentration on the oxygen atoms of the initial ZnSal increased by 7.64 kcal/mol upon coordination of a single Bi­(3-py)_3_ unit (columnar assembly length = 1, Figure 3, purple). Interestingly, as the chain is extended through alternating Bi···O and N–Zn interactions, the sigma hole of the initial Bi­(3-py)_3_ continues to deepen and the charge concentration on the oxygen atoms of the initial ZnSal continues to increase. However, these changes are quickly dampened with increasing column length as the newly added units are further away. The total deepening of the sigma hole (charge depletion) in the final assembly (length 5, Figure 3) reached 49% with respect to the free Bi­(3-py)_3_ ligand, whereas the charge concentration on the oxygen lone pairs increased by 31% with respect to the free ZnSal unit. This electrostatic analysis suggests that the mutual reinforcement between charge depletion at the Bi σ-hole and charge concentration at the ZnSal oxygen atoms (Figure 3) contributes to the observed alternating stacking, alongside the O → Bi donor–acceptor interactions identified by NBO analysis (see SI Figure S66 and discussion on page S50) and likely contributions from London dispersion.

**Figure 3:**
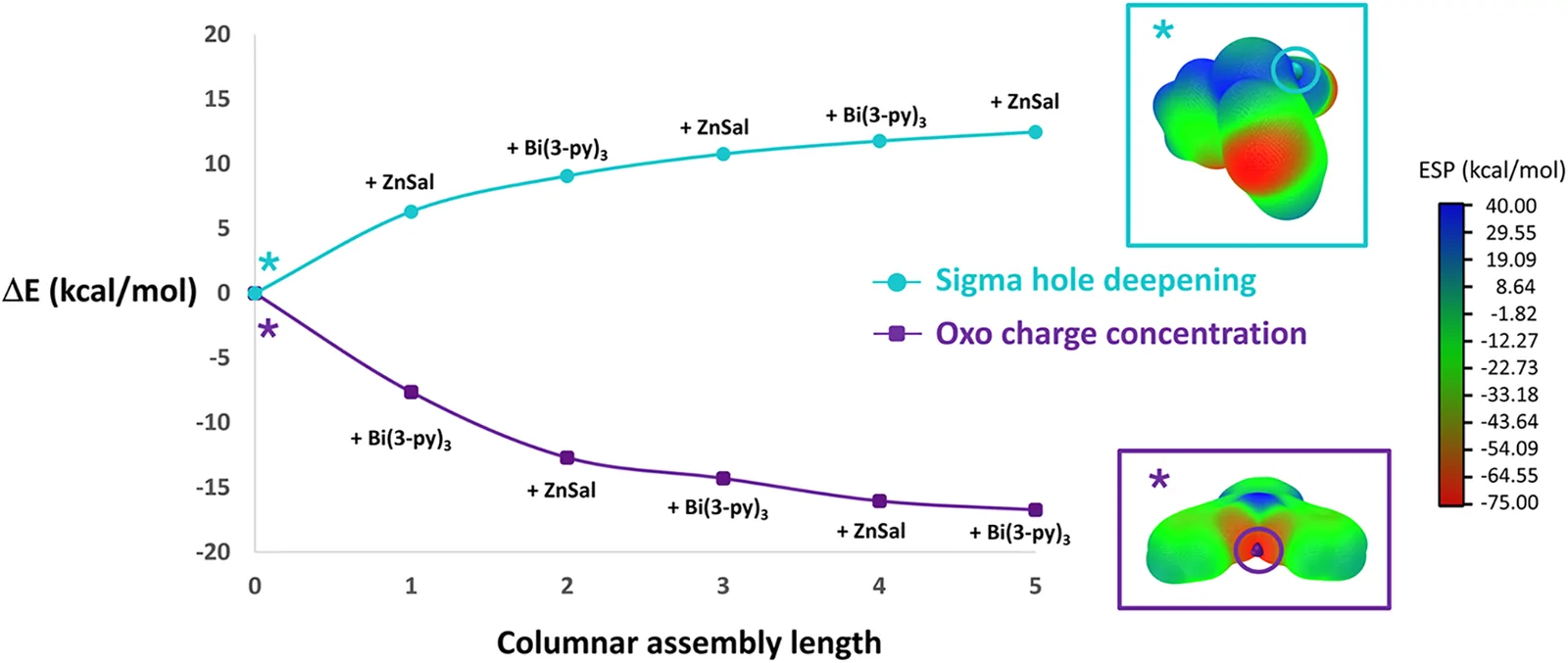
Key local maximum (*V*_max_ in blue) and key local minimum (*V*_min_ in purple) in the electrostatic surface potential (ESP) energy differences with respect to their corresponding initial states (Bi­(3-py)_3_ and the salphen complex ZnSal, respectively) as a function of the columnar length in [Bi­(3-py)_3_·(ZnSal)]_*n*_ (**1**·ZnSal).

Overall, our studies indicate that the Bi···O pnictogen bonding plays an important role in directing the formation of the 1D polymer structure. The intrinsically high Lewis acidity of the Bi center is further enhanced by the strong remote coordination of the pyridyl arm to the Zn-salphen unit, in line with our recent observations in the coordination of **1** to Zn-porphyrins.^70^ At the same time, pyridyl coordination in **1**·ZnSal also enhances the basicity of the salphen oxygen atoms, favoring the PnB-directed alternating 1D polymer observed in the solid state rather than the anticipated discrete assembly. This fundamental departure from the well-established Zn-salphen supramolecular behavior highlights the strong structure-directing role of the Lewis-acidic bismuth bridgehead. The structure of **1**·ZnSal therefore illustrates the potential of PnB as a previously unexplored structure-directing element for the self-assembly of this important class of architectures and opens new opportunities for organizing metallosalphen units through unconventional interactions and designing functional supramolecular systems.

### Effect of Substitution in the Zinc Metallosalphen

#### 1·ZnSal-^*t*^Bu and 2·ZnSal-^*t*^Bu

We reasoned that the introduction of bulky substituents at the 3 and 3′-positions of the salicylidene rings could hinder intermolecular Bi···O pnictogen bonding and thus favor the formation of discrete capsules rather than a polymeric arrangement. A similar strategy has previously been shown to disfavor self-dimerization via μ_2_-O bridging in Zn­(salphen) systems.^76,79,84,86^ With this goal in mind, we employed ZnSal-^*t*^Bu, with ^*t*^Bu groups at the 3 and 3′-positions (*i*.*e*., adjacent to the phenoxo oxygen atoms). The addition of 3 equiv of ZnSal-^*t*^Bu to a solution of **1** or **2** in CDCl_3_ gave rise to an upfield shift of the pyridyl proton signals (*ca*. 0.4 ppm for the ortho protons), consistent with pyridyl coordination to ZnSal-^*t*^Bu (see SI, Figures S8 and S14). Slow diffusion of *n*-hexane into a CH_2_Cl_2_ solution of ligand **1** or **2** (1 equiv) and ZnSal-^*t*^Bu (3 equiv) afforded crystals of **1**·ZnSal-^*t*^Bu and **2**·ZnSal-^*t*^Bu in 39% and 43% yields, respectively (Scheme 1). X-ray analysis confirmed the formation of discrete supramolecular capsules through pyridyl-N coordination in both cases, and ESI-QTOF high-resolution mass spectrometry showed the presence of the expected [M + H]^+^ peaks for the 1:3 complexes (see SI, Figures S73 and S74). Both were also characterized using NMR spectroscopy, FTIR and elemental analysis.

**Scheme 1:**
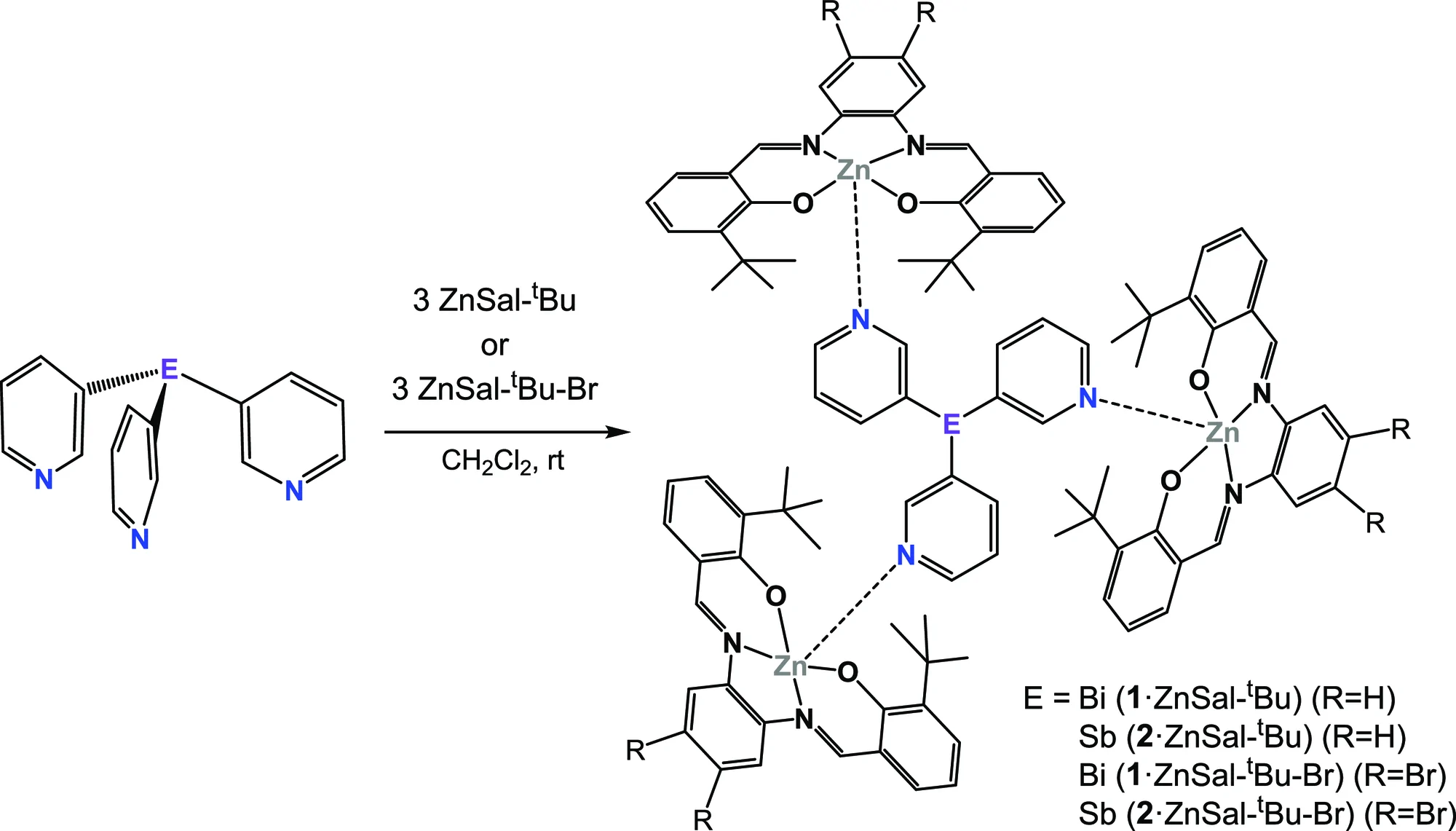
Synthesis of the Supramolecular Capsules [E­(3-py)_3_ **·**(ZnSal-Bu)_3_] (E = Bi (**1·**ZnSal-Bu)), Sb (**2·**ZnSal-Bu) and [E­(3-py)_3_ **·**(ZnSal-Bu-Br)_3_] (E = Bi (**1·**ZnSal-Bu-Br)), Sb (**2·**ZnSal-Bu-Br)

Despite their overall similarity, the solid-state structures of [E­(3-py)_3_·(ZnSal-*^t^* Bu)_3_]­[E = Bi (**1·**ZnSal-*^t^* Bu) and Sb (**2·**ZnSal-*^t^* Bu)] (Figure 5) display subtle but significant differences, revealing the marked conformational flexibility of this class of assemblies. In addition to rotation around the Zn–N­(pyridyl) bonds, the metallosalphens adopt different orientations relative to the bridgehead atom (E = Sb, Bi) via rotation of the E–C_py_ bonds (Figure 4). In [Sb­(3-py)_3_ **·**(ZnSal-*^t^* Bu)_3_], two salphens point upward (toward the bridgehead atom), while the third lies approximately in the plane defined by the three C_ipso_ atoms of the pyridyl rings (Figure 4, left and Figure 5b), reminiscent of the “In” (“closed”) conformation reported by Reek for [P­(3-py)_3_]**·**(ZnSal-*^t^* Bu)_3_,^37^ which was also found to adopt an out conformation (Figure 4, right). In contrast, the X-ray structure of the Bi analogue [Bi­(3-py)_3_]**·**(ZnSal-*^t^* Bu)_3_ exhibits an intermediate conformation, with two salphen units pointing downward (away from the bridgehead atom) while the third is oriented upward toward the bismuth center, leaving this center relatively exposed (Figure 4, center and Figure 5a)

**Figure 4:**
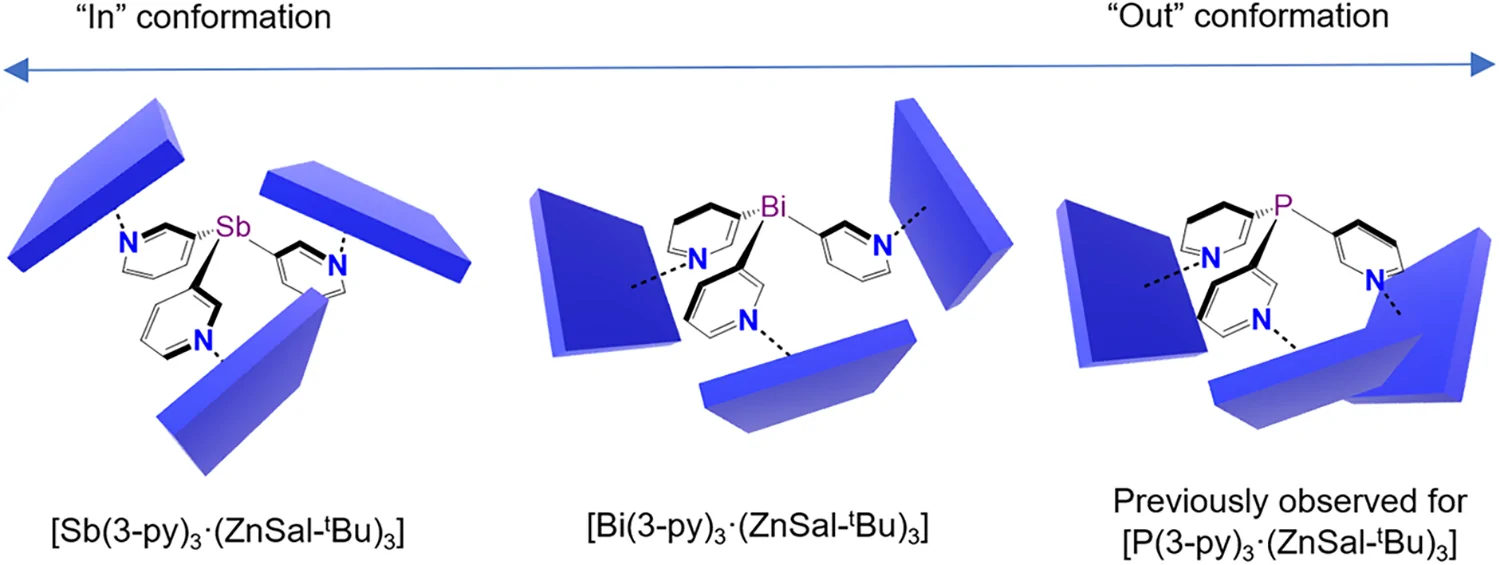
Conformations of capsules of the type [E­(3-py)_3_]·(ZnSal-^*t*^Bu)_3_ (E = P,^37^ Sb, Bi).

No intermolecular pnictogen bonding between the pnictogen center and the sterically congested phenoxo groups of adjacent salphen units is observed for either of the heavier-pnictogen assemblies **1·**ZnSal-^*t*^Bu or **2·**ZnSal-^*t*^Bu. Thus, steric congestion at the 3,3′-positions of the salphen framework appears to disfavor PnB-mediated aggregation, facilitating discrete capsule formation. Interestingly, in the solid-state structure of **1·**ZnSal-^*t*^Bu, a MeOH molecule originating from residual solvent retained by the ZnSal-*^t^* Bu building block during its synthesis is accommodated within the capsule cavity and forms an intermolecular Bi···O pnictogen bond (Bi···O distance of 2.9185 Å cf. Σ_VDW_ (Bi–O) 3.59 Å), reflecting the high Lewis acidity of the Bi center (Figure 5a). The MeOH oxygen approaches the bismuth center approximately along the extension of the E–C_py_ bond associated with the upward-oriented salphen unit (O···Bi–C_py_ 176(2)°), where the Bi atom is more exposed as a consequence of the downward orientation of the remaining salphen fragments. This arrangement highlights the importance of the conformational flexibility in enabling guest access to the bridgehead atom. More importantly, it shows that small polar molecules can be encapsulated within the cavity and stabilized by intermolecular pnictogen bonding, underscoring the unique influence of the heavy pnictogen atom on host–guest recognition.

**Figure 5:**
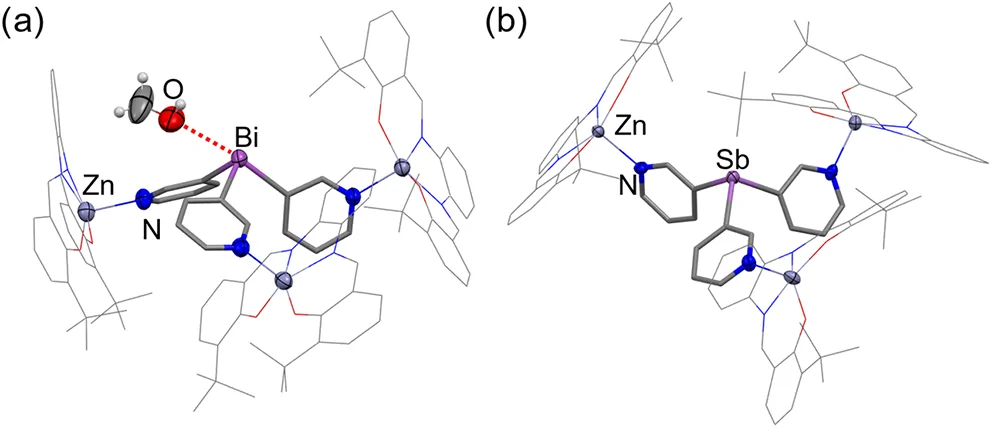
Solid-state structures of (a) [Bi­(3-py)_3_·(ZnSal-^*t*^Bu)_3_] (**1·**ZnSal-^*t*^Bu), (b) [Sb­(3-py)_3_·(ZnSal-^*t*^Bu)_3_] (**2·**ZnSal-^*t*^Bu). Displacement ellipsoids shown at 50% probability; H atoms and solvent molecules (CH_2_Cl_2_) are omitted for clarity. Selected bond lengths (Å) and angles (°): **1·**ZnSal-^*t*^Bu: Bi–C_py_ range 2.261(7)–2.271(6); N_py_–Zn range 2.066(4)–2.116(4); C_py_–Bi–C_py_ range 91.1(2)–96.7(2). **2·**ZnSal-^*t*^Bu: Sb–C_py_ range 2.13(1)–2.157(8); N_py_–Zn range 2.094(7)–2.157(8); C_py_–Sb–C_py_ range 96.0(3)–98.0(3). Color key: C (gray), Zn (dark gray), N (blue), O (red), Sb (light purple), Bi (purple).

The room-temperature ^1^H NMR spectra of **1**·ZnSal-^*t*^Bu and **2**·ZnSal-^*t*^Bu in CDCl_3_ show a single set of signals for both the E­(3-py)_3_ ligands (E = Bi, Sb). The simple pattern suggests that the observed solid-state conformations are averaged in solution, resulting in effective *C*_3*v*_ and *C*_2*v*_ symmetries for the tris­(3-pyridyl) ligand and the Zn-salphen, respectively. Variable-temperature (VT) ^1^H NMR experiments show no splitting of signals even at 218 K, indicating that this conformational exchange remains fast on the NMR time scale. However, samples containing excess ZnSal-^*t*^Bu show separate signals for the intact capsule and free ZnSal-^*t*^Bu at this temperature, revealing slow bound/free salphen exchange (see SI, Figures S63, S64). This contrasts with the porphyrin capsules [E­(3-py)_3_·(ZnTPPH)_3_] (E = P, Sb, Bi), for which Zn–pyridine exchange remained fast. Overall, these observations suggest that **1**·ZnSal-^*t*^Bu and **2**·ZnSal-^*t*^Bu retain a significant degree of dynamicity in solution, which arises primarily from intramolecular processes, (*e*.*g*. rotation around the Zn–N­(py) and C–E bonds) rather than through pyridyl dissociation. This points to more robust assemblies than the corresponding porphyrin-based capsules, consistent with the stronger Zn–N­(pyridyl) binding expected for Zn-salphen systems, as noted by Reek for P­(3-py)_3_ salphen-based capsules.^33,37^ Further supporting the integrity of the capsules in CDCl_3_, ^1^H NMR Job plot analyses indicate a 1:3 stoichiometry while ^1^H DOSY NMR are consistent with a single supramolecular species with a hydrodynamic radius similar to that estimated from their solid-state structures for **1**·ZnSal-^*t*^Bu and **2**·ZnSal-^*t*^Bu (see SI, pages S27–S28; S35–S37). Importantly, **1·**ZnSal-^*t*^Bu and **2·**ZnSal-^*t*^Bu can still form even in coordinating solvents. Titration NMR experiments in CD_3_CN gave binding constants of *K*_1_ [(1.93 ± 0.01) × 10^4^ M^–1^], *K*_2_ [(6.45 ± 0.04) × 10^3^ M^–1^], *K*_3_ [(2.15 ± 0.01) × 10^3^ M^–1^] and *K*_1_ [(1.31 ± 0.01) × 10^4^ M^–1^], *K*_2_ [(4.38 ± 0.03) × 10^3^ M^–1^], *K*_3_ [(1.46 ± 0.01) × 10^3^ M^–1^], respectively, for **1·**ZnSal-^*t*^Bu and **2·**ZnSal-^*t*^Bu (see SI for details, pages S39–S46). These results point to the greater robustness of the Zn–salphen capsule-like assemblies relative to the corresponding Sb- and Bi based porphyrin ones, expanding the conditions under which heavier pnictogen capsules can be formed to coordinating solvents such as CH_3_CN.^87^

#### 1·ZnSal-^*t*^Bu-Br and 2·ZnSal-^*t*^Bu*-*Br

To assess the generality of this approach for accessing Sb- and Bi- based salphen capsule-like assemblies, we further modified the salphen scaffold using ZnSal-^*t*^Bu-Br. This salphen retains the 3,3′-di-*tert*-butyl groups, which help to prevent pnictogen-bond-mediated aggregation in these Lewis-acidic heavier Group 15 assemblies, while introducing two electron-withdrawing Br substituents into the phenylene bridge, which could potentially increase the robustness of the capsules. Slow diffusion of *n*-hexane into a CH_2_Cl_2_ or toluene solution of ligand **1** or **2** (1 equiv) and ZnSal-^*t*^Bu-Br (3 equiv) afforded X-ray-quality crystals of [Bi­(3-py)_3_·(ZnSal-^*t*^Bu-Br)_3_] (**1·**ZnSal-^*t*^Bu-Br) and [Sb­(3-py)_3_·(ZnSal-^*t*^Bu-Br)_3_] (**2·**ZnSal-^*t*^Bu-Br) in 65 and 78% yields, respectively (Scheme 1). Both were also characterized by elemental analysis, mass spectrometry, FTIR and, despite their low solubility, ^1^H and ^13^C NMR spectroscopy (see SI pages S15–S20; Figures S77, S78, S83, and S84). In contrast to the [E­(3-py)_3_ **·**(ZnSal-^*t*^Bu)_3_] analogues, the solid-sate structures of **1·**ZnSal-^*t*^Bu-Br and **2·**ZnSal-^*t*^Bu-Br both display an “open” conformation in which one salphen unit is displaced downward while the other two point upward toward the pnictogen center (Figure 6a,b), resembling the conformation observed in the heavier porphyrin-based capsules [E­(3-py)_3_]**·**(ZnTPPH)_3_ (E = Bi and Sb).^69^ Although closely related, **1·**ZnSal-^*t*^Bu-Br and **2·**ZnSal-^*t*^Bu-Br are not isostructural, displaying subtle geometric differences that reflect the distinct steric and geometric profiles imposed by the Bi and Sb bridgehead. In addition, the downward-oriented salphen unit adopts different rotational orientations in the two structures (Figure 6c), further highlighting the conformational flexibility of these assemblies. NMR studies in CDCl_3_, including DOSY and Job plot analysis (see SI, pages S27, S29, S35, S37, S38), indicate that the 1:3 stoichiometry is retained in solution and the capsules remain intact. Nevertheless, the simplicity of the room-temperature ^1^H NMR spectra of **1·**ZnSal-^*t*^Bu-Br and **2·**ZnSal-^*t*^Bu-Br in CDCl_3_ is consistent with rapid conformational averaging on the NMR time scale analogous to that observed for the **1·**ZnSal-^*t*^Bu and **2·**ZnSal-^*t*^Bu capsules, reflecting the conformational flexibility of these systems.

**Figure 6:**
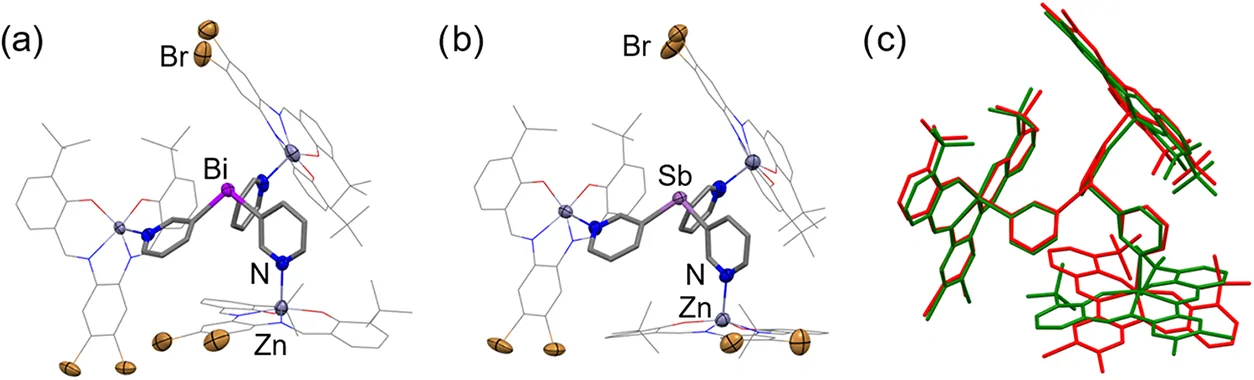
Solid-state structures of (a) [Bi­(3-py)_3_·(ZnSal-^*t*^Bu-Br)_3_] (**1·**ZnSal-^*t*^Bu-Br), (b) [Sb­(3-py)_3_·(ZnSal-^*t*^Bu-Br)_3_] (**2·**ZnSal- ^*t*^Bu-Br). (c) Both structures overlapped (**1·**ZnSal-^*t*^Bu-Br in red and **2·**ZnSal-^*t*^Bu-Br in green), showing the rotation of one of the salphen groups. Displacement ellipsoids shown at 50% probability; H atoms and solvent molecules are omitted for clarity. Selected bond lengths (Å) and angles (°): Owing to the relatively high residual factors, bond lengths and angles of **1·**ZnSal-^*t*^Bu-Br are not quoted here. **2·**ZnSal-^*t*^Bu-Br: Sb–C_py_ range 2.12(1)–2.14(1); N_py_–Zn range 2.11(1)–2.12(1); C_py_–Sb–C_py_ range 95.4(4)–97.0(5). Color key: C (gray), Zn (dark gray), N (blue), O (red), Br (brown), Sb (light purple), Bi (purple).

### Formation of Mg-Based Metallomacrocycles

Although a wide range of metal ions can be introduced into the N_2_O_2_ pocket of salphen ligands, salphen-based Mg­(II) complexes remain largely unexplored. This is particularly striking given their close structural resemblance to porphyrins, whose Mg­(II) derivatives are well established. Motivated by our previous studies of MgTPPH, which showed that E­(3-py)_3_ linkers (E = Sb, Bi) form extended 2D {[E­(3-py)_3_]_2_·(MgTPPH)_3_}_∞_ assemblies through diaxial coordination (Figure 1b), we examined whether the smaller and more flexible MgSal-^*t*^Bu (the magnesium analogue of ZnSal-^*t*^Bu) could provide access to higher-order architectures.

The lack of studies on Mg-based salphen complexes is most likely due to synthetic challenges. Indeed, we found that isolation of MgSal-^*t*^Bu in pure form was difficult due to its tendency toward hydrolysis. However, while MgSal-^*t*^Bu readily demetallates in wet CDCl_3_, it remains stable in DMSO-*d*_6_ even in the presence of large amounts of water (*ca*. 15 equiv). This suggests stabilization of the Mg­(II) center by axial DMSO coordination, consistent with previous reports in which pyridine was used to stabilize Mg­(salen)-type complexes.^88^ With this in mind, MgSal-^*t*^Bu was generated *in situ* by treating H_2_Sal-^*t*^Bu with Mg­(^*n*^Bu)_2_ in CH_2_Cl_2_, with Mg­(^*n*^Bu)_2_ acting as both base and magnesium source.^89^ Addition of ligand **1** or **2** (0.33 equiv) followed by concentration and slow diffusion of hexane afforded orange crystals corresponding to the macrocycles {[Bi­(3-py)_3_]_6_·(MgSal-^*t*^Bu)_10_} (**1**·MgSal-^*t*^Bu) and {[Sb­(3-py)]_6_·(MgSal-^*t*^Bu)_10_} (**2**·MgSal-^*t*^Bu). These reactions were subsequently optimized using the crystallographically determined 6:10 stoichiometric ratio (Figure 7a). The solid-state structure of **1·**MgSal-^*t*^Bu reveals the formation of a centrosymmetric rectangle-like macrocycle assembled from six tris­(3-pyridyl)­bismuthine ligands and ten MgSal-^*t*^Bu units (Figure 7b). Within this Bi_6_Mg_10_ heterometallic macrocycle framework, four ligands **1** act as vertices, each coordinating three MgSal-^*t*^Bu through its three 3-Py N donor atoms, giving rise to a capsule-like arrangement around the bridgehead atom. The remaining two ligands **1** act as linkers along the long edges of the macrocycle, with only two pyridyl arms coordinating MgSal-^*t*^Bu units (*i*.*e*., forming 1:2 semicapsules instead of full 1:3 assembly). Interestingly, all six bismuthine ligands present in the macrocycle exhibit a similar conformation, with one pyridyl group pointing upward (toward the Bi center) and two downward, somewhat reminiscent of the configuration observed in the discrete Zn-based assembly [Bi­(3-py)_3_ **·**(ZnSal-^*t*^Bu)_3_] (**1·**ZnSal-^*t*^Bu) (Figure 4, middle and Figure 5a). The MgSal-^*t*^Bu units present two coordination environments; six are diaxially coordinated and link the vertex ligands to define the rectangular macrocycle, four located along the long edges and two in the short edges, while the remaining four MgSal-^*t*^Bu are monoaxially coordinated in a terminal fashion at the four vertices of the macrocycle and exhibit shorter Mg–N_py_ bond distances (2.140(4) and 2.158(4) Å vs 2.253(6)–2.363(5) Å). The rectangular macrocycle measures 12.305(5) × 23.031(4) Å between the bismuth bridgeheads at the vertices. The Sb analogue **2**·MgSal-^*t*^Bu shows minor structural differences due to the distinct geometric profile of the Sb linker, resulting in a slightly smaller macrocycle measuring 12.176(1) × 22.942(1) Å (see SI, Figure S92).

**Figure 7:**
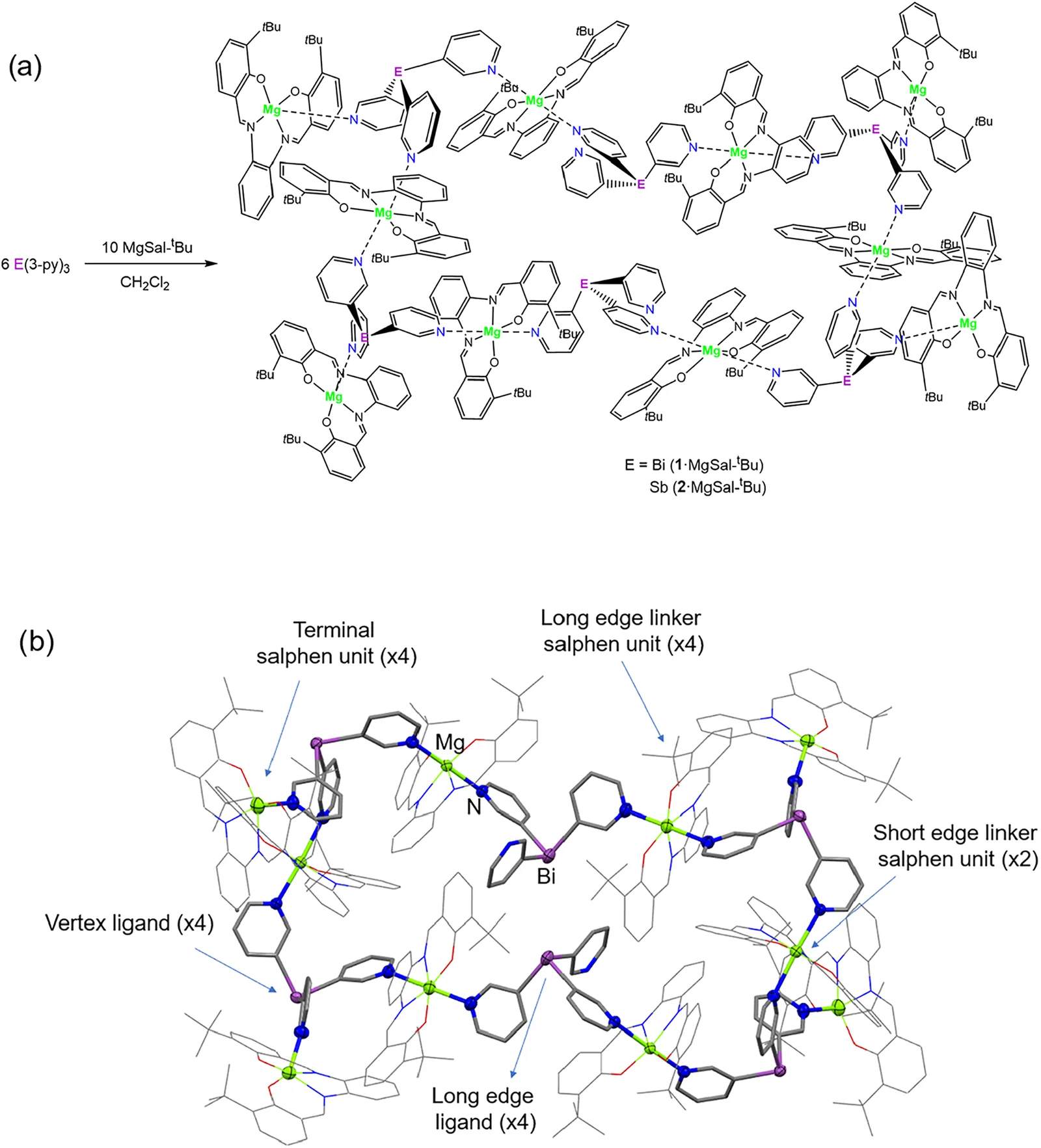
(a) Synthesis of the macrocycles {[Bi­(3-py)_3_]_6_·(MgSal-^*t*^Bu)_10_} (**1·**MgSal-^*t*^Bu) and {[Sb­(3-py)]_6_·(MgSal-^*t*^Bu)_10_} (**2·**MgSal-^*t*^Bu). (b) Solid-state structure of (**1·**MgSal-^*t*^Bu). Displacement ellipsoids shown at 50% probability; H atoms and solvent molecules are omitted for clarity. Selected bond lengths (Å) and angles (°): Bi–C_py_ range 2.229(6)–2.259(5); N_py_–Mg range 2.140(4)–2.363(5); C_py_–Bi–C_py_ range 90.8(2)–96.4(2).

To further investigate the formation of these macrocyclic assemblies and the structural influence of the bridgehead atom in the linker, we selected the lighter tris­(3-pyridyl)­amine linker N­(3-py)_3_ (**3**), which is commercially available and commonly employed in supramolecular chemistry. As the lightest group 15 analogue, N­(3-py)_3_ presents a planar geometry corresponding to sp^2^ hybridization. This contrasts sharply with the pyramidal geometries of Sb­(III) and Bi­(III), the latter of which exhibits a Bi­(III) lone pair dominated by 6s character due to relativistic stabilization, resulting in reduced s–p mixing leading to greater pyramidalization.

Reaction of **3** and three equivalents of MgSal-^*t*^Bu under similar conditions as previously afforded crystals suitable for X-ray diffraction analysis of {[N­(3-py)_3_]_4_·(MgSal-^*t*^Bu)_6_} (**3·**MgSal-^*t*^Bu). After optimization of the reaction conditions using the 4:6 ligand-to-salphen ratio determined from the X-ray structure, **3·**MgSal-^*t*^Bu was obtained in 14% crystalline yield (Figure 8).

**Figure 8:**
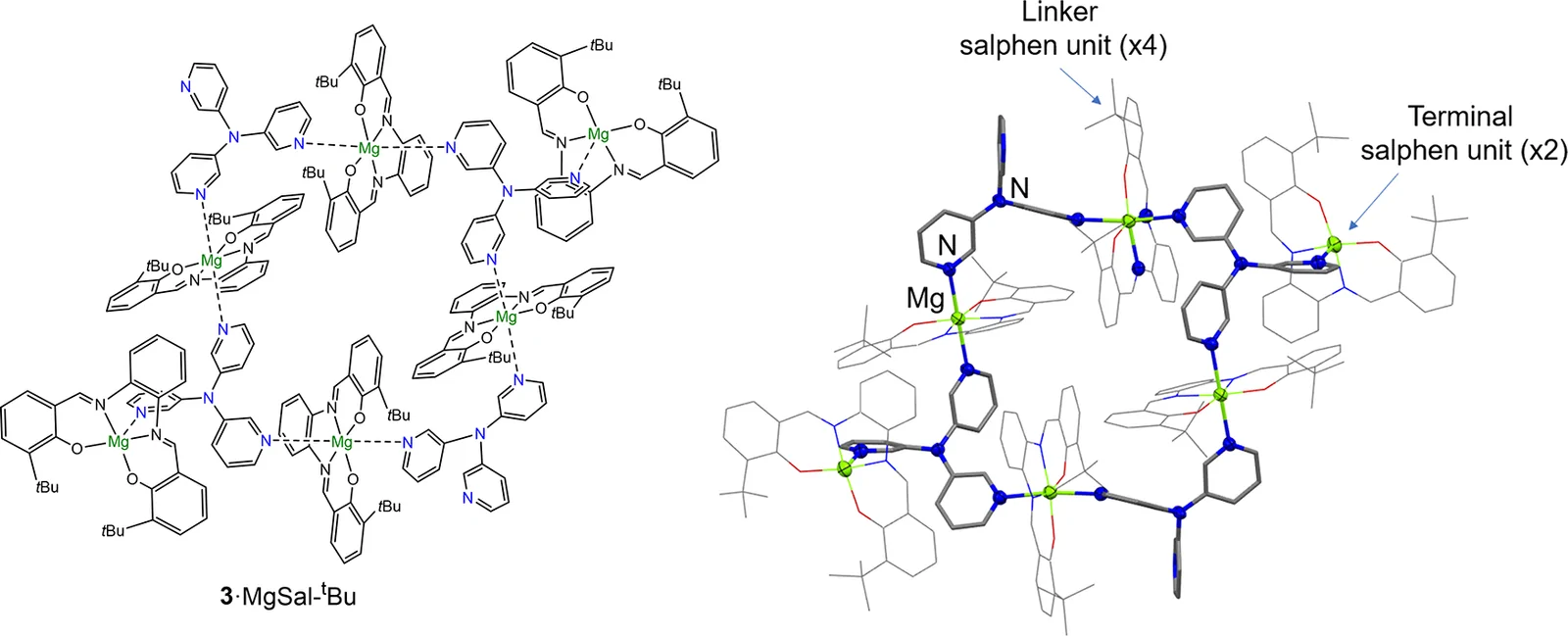
(Left) line drawing of the macrocycle {[N­(3-py)_3_]_4_·(MgSal-^*t*^Bu)_6_} (**3·**MgSal-^*t*^Bu) and (right) X-ray structure of (**3·**MgSal-^*t*^Bu). Displacement ellipsoids shown at 50% probability; H atoms and solvent molecules are omitted for clarity. Selected bond lengths (Å) and angles (°): N–C_py_ range 1.397(4)–1.430(4); N_py_–Mg range 2.178(3)–2.326(4); C_py_–N–C_py_ range 118.7(3)–120.4(3).

The solid-state structure of **3·**MgSal-^*t*^Bu reveals a centrosymmetric square-like macrocycle formed by six MgSal-^*t*^Bu units and four N­(3-py)_3_ ligands at the vertices (Figure 8, right). This assembly is substantially smaller than the heavier Bi and Sb analogues **1·**MgSal-^*t*^Bu and **2·**MgSal-^*t*^Bu, with distances between the bridgehead nitrogen atoms of 10.745(5) × 10.998(4) Å. Four MgSal-^*t*^Bu units are diaxially coordinated and act as linkers; the remaining two are terminal, monoaxially coordinated salphen fragments at opposite vertices. Thus, all four N­(3-py)_3_ ligands act as vertices, but only two coordinate an additional terminal salphen to form capsule-like substructures in which the planar nitrogen center is flanked by three salphen units; the other two bind only two diaxial salphen linkers, forming semicapsule-like motifs. In **3·**MgSal-^*t*^Bu, all four N­(3-py)_3_ ligands present a similar conformation in which the pyridyl nitrogen atoms are oriented toward the same face, away from the central N, resembling the “out” conformation (see Figure 4).^90^ This generates a funnel-like arrangement of the MgSal-^*t*^Bu panels, with the planar bridgehead nitrogen located inside the cavity.

In contrast to the Zn­(salphen)-based assemblies, the solid-state structures of **1**·MgSal-^*t*^Bu, **2**·MgSal-^*t*^Bu and **3**·MgSal-^*t*^Bu are not retained in CDCl_3_. Although the stoichiometry of the dissolved crystalline samples confirms the expected ligand-to-salphen ratios observed in the solid state, ^1^H DOSY experiments indicate that the assemblies collapse into smaller aggregates in solution, reflecting the more labile nature of the Mg­(II)-salphen systems as compared to the Zn­(II)-salphen counterparts (see SI, pages S30–S34).

As background to this study, we also investigated the ability of N­(3-py)_3_ (**3**) to assemble supramolecular capsule-like assemblies with ZnSal-^*t*^Bu and ZnSal-^*t*^Bu-Br. Solution NMR experiments and solid-state analyses confirmed the formation of discrete 1:3 supramolecular capsules in both phases (see SI, pages S28–S29, pages S71–S74). In the solid state, the [N­(3-py)_3_·(ZnSal-^*t*^Bu)_3_] (**3·**ZnSal-^*t*^Bu) and [N­(3-py)_3_·(ZnSal-^*t*^Bu-Br)_3_] (**3·**ZnSal-^*t*^Bu-Br) assemblies exhibit the same conformation, with the three pyridyl arms oriented toward the same face of the assembly, adopting the funnel-like arrangement also observed in **3**·MgSal-^*t*^Bu. The structures of **3·**ZnSal-^*t*^Bu and **3·**ZnSal-^*t*^Bu-Br are more compact than those of their heavier Sb and Bi analogues, consistent with the shorter N–C bonds relative to the Sb–C and Bi–C bonds, which lead to a contraction of the cavity despite the wider C–N–C angle. This contraction is reflected in the shorter average Zn···Zn separation for the nitrogen-based assemblies (8.75 Å for **3·**ZnSal-^*t*^Bu vs 10.04 Å for **2·**ZnSal-^*t*^Bu and 9.72 Å for **1·**ZnSal-^*t*^Bu, with a similar trend for the ZnSal-^*t*^Bu-Br series, see SI Figures S88 and S90). This bridgehead-dependent contraction is echoed in the Mg-salphen macrocycles, with the larger Sb­(III) and Bi­(III) bridgeheads in **1** and **2** giving more expanded capsule-like arrangements embedded within rectangular macrocycles and **3** forming a compact and smaller square-like macrocycle.

Taken together, these results show that the metal center in the salphen building block plays a dominant role in determining the outcome of the self-assembly process, with Zn­(II)-salphen derivatives leading to discrete capsule formation and Mg­(II)-salphen promoting their organization into higher-order macrocycles through diaxial coordination, with the identity of the bridgehead atom providing a further handle to modulate the size and shape of the resulting assemblies.

### Proof-of-Concept Catalytic Studies and Reversible ON/OFF Control of Sb Catalysis

As noted in the introduction, organoantimony and organobismuth catalysis have recently emerged as an attractive complement to transition-metal-based processes, and significant advances in catalysis have been accomplished in recent years and highlighted in recent reviews.^31,49,51,62^ In contrast to transition metal catalysis, however, their reactivity in confined spaces remains largely unexplored. Building on our previous studies with Zn-metalloporphyrin-based assemblies, we investigated the effect of the encapsulation of Sb and Bi in more-robust Zn-salphen assemblies on the catalytic behavior of ligands **1** and **2** using [E­(3-py)_3_ **·**(ZnSal-^*t*^Bu)_3_] (E = Bi, Sb; **1·**ZnSal-^*t*^Bu and **2·**ZnSal-^*t*^Bu). We selected two model reactions that involve E­(III)/E­(V) redox cycling and have been recently reviewed, namely, the Bi-catalyzed oxidative cleavage of 1,2-diols, using the previously studied substrates pinacol and benzopinacol, and the Sb-catalyzed oxidation of α-hydroxyketones.^50,91–93^

The catalytic performance of the free ligand tris­(3-pyridyl)­bismuthine (**1**) was evaluated in the Bi-catalyzed oxidative cleavage of 1,2-diols (pinacol (Me_2_C­(OH)­C­(OH)­Me_2_) and benzopinacol (Ph_2_C­(OH)­C­(OH)­Ph_2_)) in the presence of the stoichiometric oxidant *N*-bromosuccinimide (NBS) under the reaction conditions previously established by Barton.^91,92^ In the absence of **1**, no oxidation was observed. When free bismuthine **1** was used as the catalyst (1 mol %), the oxidation of both pinacol and benzopinacol to acetone and benzophenone was accomplished with yields of 65 and 31%, respectively, after 90 min (Table 1, entry 1).^69^ In contrast, the use of its encapsulated form, **1·**ZnSal-^*t*^Bu, led to a dramatic decrease in the oxidation of pinacol (<5%), while the oxidation of benzopinacol was reduced to a lesser extent (15%, entry 2). To assess the effect of encapsulation on substrate selectivity, competitive oxidative cleavage experiments were carried out in the presence of both substrates. Using free ligand **1**, pinacol and benzopinacol were oxidized in a 3.8:1 ratio (entry 3).^69^ This selectivity was reversed when the capsule **1·**ZnSal-^*t*^Bu was used, favoring benzopinacol oxidation by at least 5.8 fold (product ratio of 1:5.8, entry 4). This inversion of selectivity upon encapsulation is consistent with that previously observed for the porphyrin-based system [Bi­(3-py)_3_]**·**(ZnTPPH)_3_, which gave a 1:3.5 product ratio.^69^ The more pronounced selectivity observed for **1·**ZnSal-^*t*^Bu likely reflects the greater robustness of the Zn-salphen assemblies, which in turn leads to more effective shielding of the Bi center. Although the origin of this selectivity inversion remains unclear, the observed difference is unlikely to be governed solely by steric effects. Nonetheless, these results suggest that supramolecular encapsulation does not simply attenuate Bi-centered catalysis, but can fundamentally change its substrate preferences, altering substrate preference under competitive conditions.

**Table 1:**
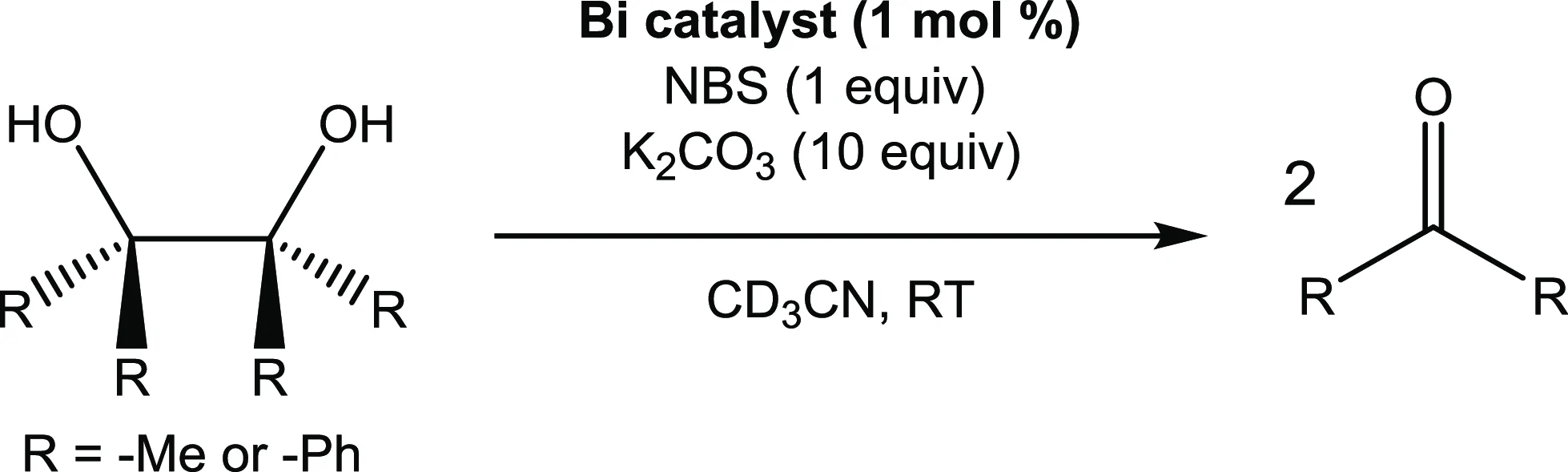
Oxidative Cleavage of 1,2-Diols in the Presence of **1** or **1·**ZnSal-^*t*^Bu

		Yield [%]	
Entry	Bi Catalyst	R = Me	R = Ph
**1** ^a^	Bi(3-py)_3_	65	31
**2** ^a^	[Bi(3-py)_3_·(ZnSal-^*t*^Bu)_3_]	<5	15
**3** (Competitive)^b^	Bi(3-py)_3_	76	20
**4** (Competitive)^b^	[Bi(3-py)_3_·(ZnSal-^*t*^Bu)_3_]	<5	29

a Oxidative cleavage of 1,2-diols in the presence of 1 or 1·ZnSal-^*t*^Bu. Reaction conditions: 1,2-diol (0.14 mmol), NBS (0.14 mmol), K_2_CO_3_ (1.4 mmol), 1.5 mL of CD_3_CN.

b Reaction conditions: for competitive experiments, 0.07 mmol of each 1,2-diol was added. Yields were determined by ^1^H NMR after 90 min. Entries 1 and 3 reproduce previously reported data (ref 67) and are included here for comparison.

To evaluate the effect of the encapsulation of the stibine **2**, we examined the Sb-catalyzed aerobic (under air) oxidation of the α-hydroxyketones^50,93^ acetoin (MeCOCH­(OH)­Me), butyroin (EtCOCH­(OH)­Et) and benzoin (PhCOCH­(OH)­Ph) to the corresponding diketones diacetyl (MeCOCOMe), hexane-2,3-dione (EtCOCOEt) and benzil (PhCOCOPh). No oxidation was observed in the absence of **2**, whereas the free stibine **2** catalyzed the oxidation of all the substrates in moderate yields (Table 2, entry 1), consistent with our previous studies for acetoin and benzoin.^69,70^ To our surprise, encapsulated **2·**ZnSal-^*t*^Bu was essentially inactive toward all three substrates (entry 2). This behavior contrasts with that observed in the previous capsules based on **2** and Zn-porphyrin building blocks, which showed substrate-dependent catalytic activity^69^ that could be modulated via substitution in the periphery of the porphyrin (*i*.*e*., ZnTPPF_5_ stabilized a blocked capsule conformation that led to a decrease in the reaction rate relative to the ZnTPPH assemblies).^70^ The formation of the robust assembly **2·**ZnSal-^*t*^Bu therefore seems to lead to a more-protected bridgehead center, effectively suppressing its catalytic activity.

**Table 2:**
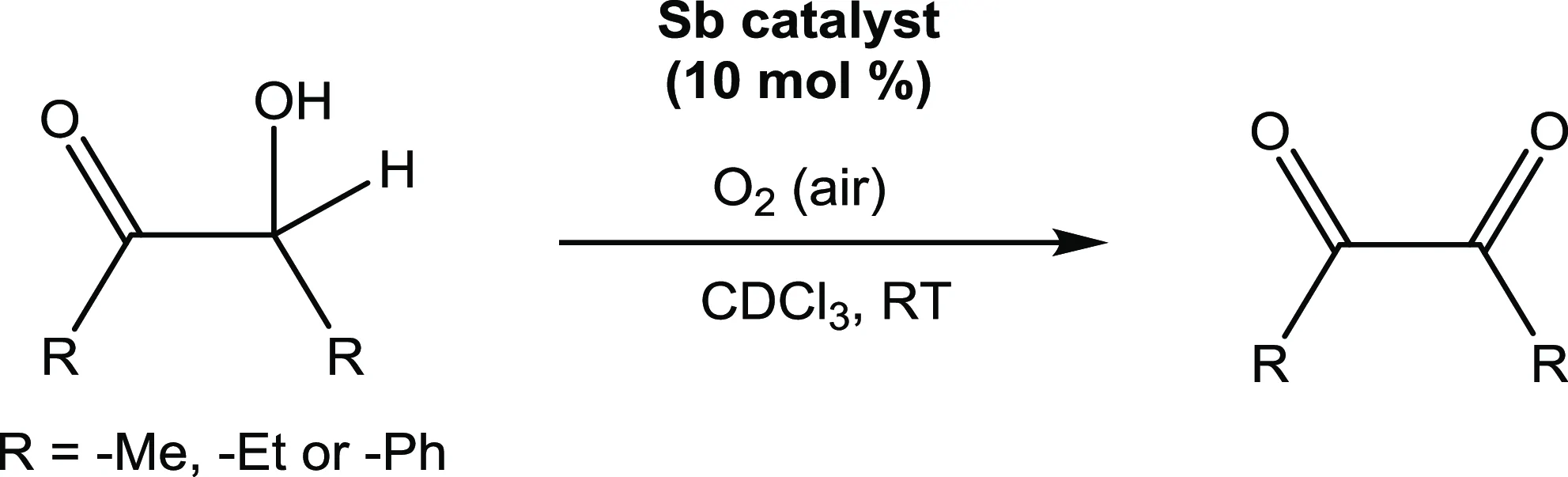
Aerobic Oxidation of α-Hydroxyketones in the Presence of **2** or **2·**ZnSal-^*t*^Bu

			Yield [%]	
Entry	Sb Catalyst	R = Me	R = Et	R = Ph
**1** ^a^	Sb(3-py)_3_	42	35	46
**2** ^a^	[Sb(3-py)_3_·(ZnSal-^*t*^Bu)_3_]	<5	<5	<5

a Sb-catalyzed aerobic oxidation of α-hydroxyketones. Reaction conditions: α-hydroxyketone (0.02 mmol), Sb catalyst (**2** and **2·**ZnSal-^*t*^Bu, 10% mol), 1 mL of CDCl_3_, prepared under air in a J. Young valve NMR tube. Yields were determined by ^1^H NMR after 24 h.

The complete inhibition of catalysis suggested the possibility of switching between two catalytic states (ON/OFF) through the reversible encapsulation and release of the Sb center. This is particularly significant, as achieving a fully OFF state remains a common limitation in this type of systems. Although supramolecular ON/OFF behavior is well precedented in organocatalysis and transition-metal catalysis,^25,26^ analogous control over main-group catalysts, particularly those based on heavier p-block elements such as Sb and Bi, remains largely unexplored.

We reasoned that de-encapsulation of Sb­(3-py)_3_ (**2**) could be accomplished using a coordinating ligand that could compete with **2** for Zn coordination. We found that while the addition of excess CD_3_CN (a few drops) did not substantially affect the ^1^H NMR chemical shifts of [Sb­(3-py)_3_ **·**(ZnSal-^*t*^Bu)_3_] (**2·**ZnSal-^*t*^Bu), indicating that the assembly remained intact, the addition of an excess of deuterated pyridine (2 equiv per ZnSal-^*t*^Bu) resulted in an immediate shift of the ortho-pyridyl protons (*ca*. 0.4 ppm), consistent with disassembly and release of free Sb­(3-py)_3_ (**2**) (Figure 9c). A smaller shift of the Zn-salphen signals was also observed, consistent with the formation of the pyridine-coordinated ZnSal-^*t*^Bu (Py**·**ZnSal-^*t*^Bu).^94^ These observations reflect the stronger coordination of pyridine to Zn-salphen relative to acetonitrile and demonstrate that competitive ligand binding can trigger the release of the Sb catalyst **2**. Importantly, the process is reversible. Removal of pyridine resulted in reassembly of the capsule and re-encapsulation of ligand **2**, as demonstrated by ^1^H NMR (Figure 9d).

**Figure 9:**
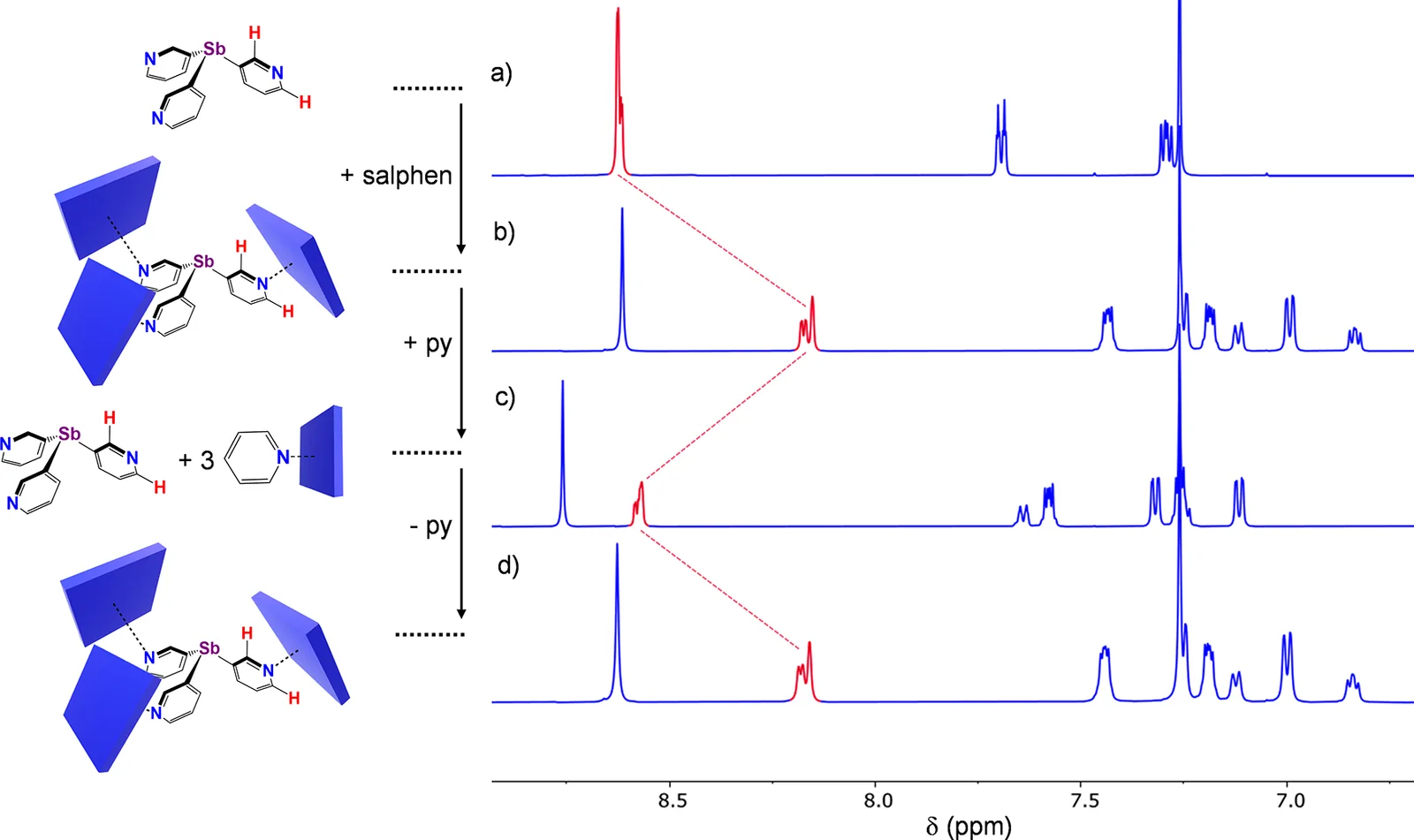
Stacked ^1^H NMR CDCl_3_ spectra of the reversible assembly and release of ligand Sb­(3-py)_3_ (**2**) in CDCl_3_, with selected signals of the ligand **2** highlighted in red. (a) Free ligand **2**. (b) After the addition of three equivalents of ZnSal-^*t*^Bu, showing capsule formation. (c) After the addition of py-*d*_5,_ showing capsule disassembly and release of **2.** (d) After removal of py-*d*_5_, showing capsule reassembly.

Having achieved the reversible encapsulation and release of catalyst **2** in CDCl_3_, we next examined whether the catalytic activity could be switched between the ON and OFF states on demand under catalytic conditions. As a proof of concept, the oxidation of benzoin (PhCOCH­(OH)­Ph) to benzil (PhCOCOPh) was monitored via ^1^H NMR spectroscopy (Figure 10). When the Sb center was encapsulated in [Sb­(3-py)_3_ **·**(ZnSal-^*t*^Bu)_3_] (OFF state), no reaction was observed (*t* = 0 to 4 h). Upon the addition of pyridine (2 equiv per ZnSal-^*t*^Bu, *t* = 4 h), capsule disassembly and the release of **2** was observed, accompanied by the oxidation of benzoin to benzil (*t* = 4 to 10 h), indicating successful switching to the ON state. Removal of pyridine by either evaporation (*t* = 10 h) or the addition of ZnSal-^*t*^Bu (see SI Figures S95, S96) restored the supramolecular assembly [Sb­(3-py)_3_ **·**(ZnSal-^*t*^Bu)_3_], returning the system to the OFF state.

**Figure 10:**
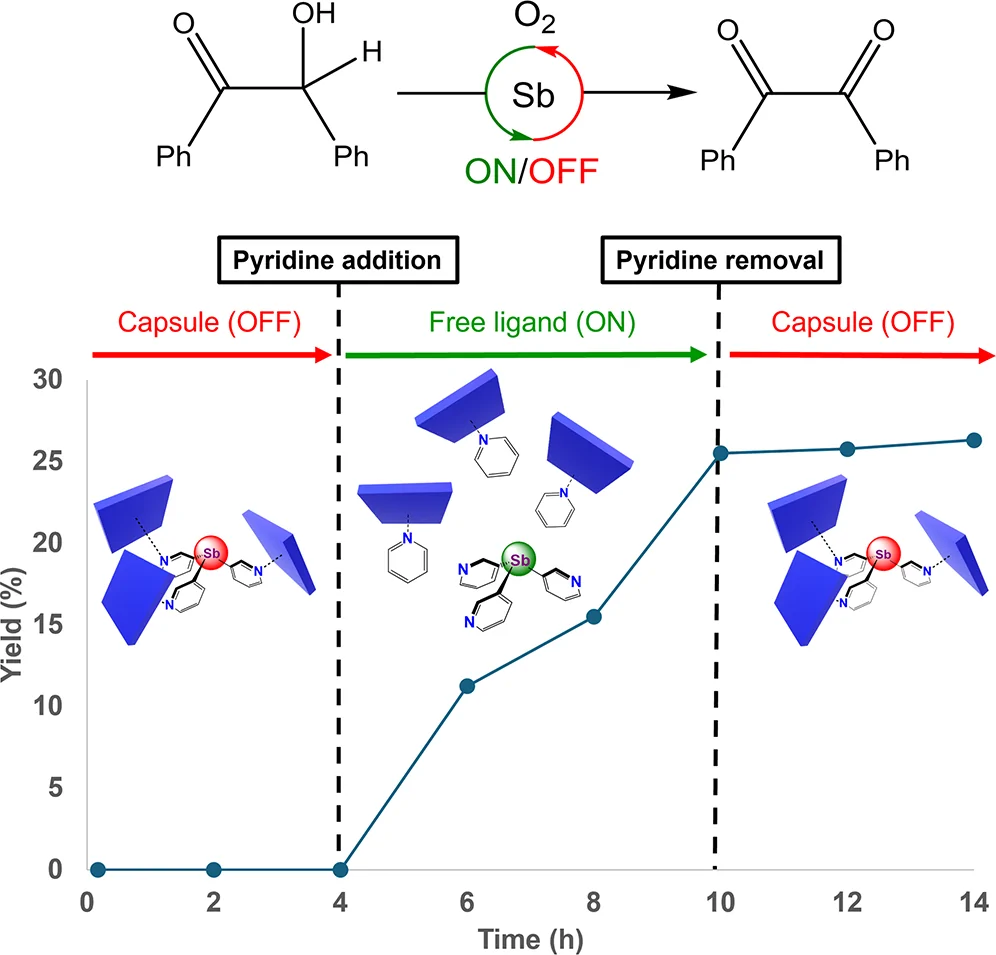
Reversible ON/OFF control of Sb­(3-py)_3_-catalyzed aerobic oxidation of benzoin to benzil through pyridine-induced capsule disassembly and re-encapsulation.

This system, to the best of our knowledge, is the first example of reversible ON/OFF control of antimony-mediated catalysis through supramolecular encapsulation. It provides the first step for extending stimulus-responsive supramolecular control to other pnictogen catalysis, including bismuth, as well as to alternative external triggers. To this end, we also found that the system exhibits pH responsiveness, as acidification with acetic acid promotes de-encapsulation (via salphen demetalation, see SI, Figure S98) and concomitant catalytic activation. The addition of NEt_3_ did not fully reverse this process under catalytic conditions, as complete reassembly was not observed, and, consequently, a complete OFF state was not restored. This limitation could, however, be circumvented by adding ZnSal-*^t^* Bu, which promoted reassembly of **2**·ZnSal-^t^Bu and restored the OFF state (see SI, Figures S99–S101).

## Conclusions

We have shown that the heavy Group 15 tris­(3-pyridyl) linkers E­(3-py)_3_ [E = Bi, (**1**), Sb (**2**)] can be combined with Zn- or Mg-metallosalphen building blocks to access supramolecular assemblies with controllable architectures ranging from 1D polymers to discrete capsule-like assemblies, and higher-order multicapsule macrocycles depending on the salphen substitution pattern and the choice of metal center. At the same time, the heavier pnictogen atoms open access to recognition and reactivity modes not available in lighter analogues.

The consequences of incorporating heavier, more Lewis-acidic pnictogen centers with stronger PnB donor character can be seen in the formation of the 1D polymer **1·**ZnSal, which illustrates how PnBs can change the traditional outcome of the assembly. This structure shows that conventional pyridyl nitrogen coordination and strong Bi···O pnictogen bonding (PnB), enhanced by salphen coordination, can operate together to access salphen-based architectures distinct from those obtained with traditional polytopic ligands.

Steric congestion at the 3,3′-positions of the salphen framework disfavors PnB-mediated aggregation and favors discrete capsule formation, while preserving the ability of the Bi center to engage in intermolecular pnictogen bonding, as illustrated by the encapsulation of a MeOH molecule within **1·**ZnSal-^*t*^Bu. Replacing Zn­(II) with the less commonly employed Mg­(II) in the metallosalphen redirects the assembly from discrete assemblies to large heterobimetallic macrocycles formed through hierarchical organization of capsule-like substructures. The size and shape of the resulting macrocycles can be modulated by changing the bridgehead atom E of the linker. This is further illustrated by the lighter N­(3-py)_3_ analogue, which forms smaller capsule-like substructures that assemble into a compact macrocycle, whereas the heavier Sb­(3-py)_3_ and Bi­(3-py)_3_ provide access to larger capsule-like substructures and more expanded rectangular macrocycles.

Finally, we have shown that the encapsulation of Bi and Sb centers within robust but reversible Zn-salphen assemblies can modulate both the rate and selectivity of main-group-mediated oxidative cleavage of organic diols and oxidation of α-hydroxyketones. The reversible encapsulation of the Sb center in **2** provides a supramolecularly controlled ON/OFF main-group catalyst for the aerobic oxidation of α-hydroxyketones, representing a rare example of main-group ON/OFF catalysis regulated by supramolecular confinement. Ongoing work is focused on examining how capsule size, shape, and the identity of the main-group bridgehead affect other catalytic reactions, as well as on extending this strategy to other main-group catalytic transformations, such as pnictogen-bonding catalysis, as well as to alternative external triggers for switchable catalysis.

## Experimental Section

### General Considerations

**Caution!** All Sb- and Bi-containing compounds were treated as potentially toxic, particularly the antimony derivatives, and must be handled in a well-ventilated fume hood while wearing appropriate personal protective equipment. All air- and moisture-sensitive manipulations were carried out under dry nitrogen using standard Schlenk techniques. Mg­(*^n^* Bu)_2_ is a pyrophoric, water-reactive, and hazardous organometallic reagent; it must be handled strictly under inert conditions and kept completely isolated from air and moisture.

### General Experimental Techniques

Products were isolated and handled under a nitrogen atmosphere. 3-Bromopyridine, NMR solvents, and reaction solvents were stored over molecular sieves and degassed using three freeze–pump–thaw cycles under nitrogen prior to use. Compounds ZnSal, ZnSal-^*t*^Bu, 2H-Sal-*^t^* Bu, Sb­(3-py)_3_ (**2**) and Bi­(3-py)_3_ (**1**) were synthesized as described previously.^68,74,95^ The remaining reagents were purchased from commercial suppliers and used without further purification.

NMR spectra were recorded using an Agilent DD2 500 MHz and an Agilent M2 400 MHz Agilent instrument, both equipped with a ONENMR probe in the NMR service of the Laboratory of Instrumental Techniques of the University of Valladolid (L.T.I., https://www.laboratoriotecnicasinstrumentales.es). ^1^H and ^13^C NMR chemical shifts (δ) are reported in parts per million (ppm) and are referenced to TMS, using solvents as internal references. For DOSY experiments, ^1^H spectra were acquired with a 500 MHz Agilent spectrometer using the 2D DOSY gradient compensated stimulated echo with convection compensation (DgcsteSL-cc) pulse sequence. Sixteen gradient levels ranging from 7 to 53 G/cm (12% to 88% of the maximum gradient strength) were used. The diffusion delay (Δ) was 50 ms, and the diffusion gradient length (δ) was 1.7 ms. For each DOSY NMR experiment, a series of 16 spectra was collected. Coupling constants (*J*) are reported in Hertz (Hz). Standard abbreviations are used to indicate multiplicity: s = singlet, d= doublet, t = triplet, m = multiplet, dd = doublet of doublets. NMR peak assignments were performed with the help of additional 2D NMR experiments (^1^H–^13^C gcHSQC, ^1^H–^13^C gcHMBC, ^1^H–^1^H gCOSY, ^1^H–^1^H ROESYAD and ^1^H–^1^H NOESY). Selected spectra can be found in the SI. NMR spectra were analyzed using MNova v14.2.1.^96^ High-resolution mass spectra were recorded at the mass spectrometry service of the Laboratory of Instrumental Techniques of the University of Valladolid. Samples were measured using a Bruker maXis Impact quadrupole time-of-flight (Q-TOF) mass spectrometer equipped with an electrospray ionization (ESI) source. Infrared spectra were recorded with Perkin–Elmer Frontier (4000–200 cm^–1^) equipped with an ATR accessory (Attenuated total reflection) for the direct recording of solid samples. Standard abbreviations are used to indicate intensity: vs (very strong), s (strong), m (medium), w (weak). Elemental analysis was obtained using a Thermo Scientific FLASH 2000 Elemental Analyzer at the Parque Científico Tecnológico (PCT) facilities of the University of Burgos. In cases where satisfactory elemental analyses could not be obtained, the bulk purity of the isolated materials was confirmed by ^1^H NMR spectroscopy using spectra with high signal-to-noise ratios. However, the presence of low-level and/or NMR silent impurities cannot be excluded.

Diffraction data were collected using an Oxford Diffraction Supernova diffractometer equipped with an Atlas CCD area detector and a four-circle kappa goniometer. For the data collection, a Mo microfocused source with multilayer optics was used. All crystals used for single-crystal X-ray diffraction were selected directly from the mother liquor and immediately mounted under perfluorohydrocarbon oil to minimize atmospheric oxidation, hydrolysis, and solvent loss. Data integration, scaling, and empirical absorption correction were performed using the CrysAlisPro software package.^97^ The structure was solved by direct methods and refined by full-matrix-least-squares against *F* ^2^ with SHELX^98^ in OLEX2.^99^ Non-hydrogen atoms were refined anisotropically, and hydrogen atoms were placed at idealized positions and refined using the riding model. Graphics were made with OLEX2 ^99^ and MERCURY.^100^ PXRD measurement was performed in the LTI of the University of Valladolid using a Bruker Discover D8 diffractometer. The PXRD pattern of the isolated compound was then compared with that predicted by the software package MERCURY from single-crystal X-ray analysis.

### Synthetic Procedures

#### Synthesis of [Bi­(3-py)_3_·(ZnSal)]*_n_* (1·ZnSal)

Bi­(3-py)_3_ (22 mg, 0.050 mmol) and salphen (ZnSal) (19 mg, 0.050 mmol) were combined in 15 mL of CHCl_3_. The resulting mixture was stirred for 30 min and filtered with a syringe filter into a narrow Schlenk flask to yield a yellow solution. Slow diffusion of *n*-hexane (30 mL) at low temperature (−25 °C) yielded **1·ZnSal** as orange crystals suitable for X-ray diffraction analysis, which were dried under vacuum. Yield (calculated as [Bi­(3-py)_3_·(ZnSal)]): 12 mg (0.015 mmol, 30%). Characterization of the resulting compound by nuclear magnetic resonance (NMR) and mass spectrometry (MS) could not be performed due to its insolubility in common organic solvents. However, in DMSO-*d*_6_, **1**·ZnSal dissociates into ZnSal and free ligand **1** in the expected 1:1 ratio, as observed by ^1^H NMR spectroscopy (Figure S1 and S2). Satisfactory elemental analysis could not be obtained.

#### Synthesis of [Bi­(3-py)_3_·(ZnSal-*^t^* Bu)_3_] (1·ZnSal-^*t*^Bu)

A narrow Schlenk tube was charged with Bi­(3-py)_3_ (11.9 mg, 0.027 mmol) and ZnSal-^*t*^Bu (40 mg, 0.081 mmol), and 3 mL of CH_2_Cl_2_ was then added. The resulting orange solution was stirred for 10 min at room temperature. Slow diffusion of *n*-hexane (15 mL) at room temperature yielded **1·**ZnSal-^*t*^Bu as orange needles suitable for X-ray diffraction analysis, which were dried under vacuum. Yield (calculated as [Bi­(3-py)_3_·(ZnSal-*^t^* Bu)_3_] based on elemental analysis that indicates complete solvent loss from the crystal): 20.4 mg (0.011 mmol, 39%).^**1**^ **H NMR (298 K, CDCl**_**3**_, **500 MHz):** δ 8.63 (s, 6H, H_10_), δ 8.30 (s, 3H, H_2_), δ 8.18 (d, ^3^ *J* = 5.20 Hz, 3H, H_6_), δ 7.45 (m, 9H, H_7_ + H_4_), δ 7.25 (m, 6H, H_14_), δ 7.18 (m, 6H, H_8_), δ 7.01 (d, ^3^ *J* = 7.61 Hz, 6H, H_12_), δ 6.89 (m, 3H, H_5_), δ 6.42 (t, ^3^ *J* = 7.61 Hz, 6H, H_13_), δ 1.37 (s, 54H, H_18_).^**13**^ **C­{** ^**1**^ **H} NMR (298 K, CDCl**_**3**_, **126 MHz):** δ 172.90 (s, C_16_), δ 162.51 (s, C_10_), δ 154.74 (s, C_2_py), δ 149.76 (s, C_3_py), δ 148.27 (s, C_6_py), δ 147.05 (s, C_4_py), δ 142.94 (s, C_15_), δ 140.15 (s, C_9_), δ 134.21 (s, C_12_), δ 131.33 (s, C_14_), δ 127.84 (s, C_5_py), δ 127.04 (s, C_8_), δ 119.55 (s, C_11_), δ 115.60 (s, C_7_), δ 113.12 (s, C_13_), δ 35.57 (s, C_17_), δ 29.71 (s, C_18_). **Elemental analysis** (%) calculated for **1·**ZnSal-^*t*^Bu (C_99_H_102_N_9_O_6_Bi_1_Zn_3_): C, 62.0; H, 5.4; N, 6.6. Found: C, 62.3; H, 5.6; N, 6.2. **HR-MS** (ESI, positive ion mode ESI-QTOF): *m*/*z* for C_99_H_103_N_9_O_6_Bi_1_Zn_3_ [**1·**ZnSal-^*t*^Bu + H]^+^ calculated: 1918.5686. Found: 1918.5713 (0.3 ppm error). **FT-IR** (ATR, ν/cm^–1^): 2953 (w), 1604 (s), 1580 (s), 1528 (s), 1429 (m), 1385 (m), 1179 (s), 1144 (m), 749 (vs), 530 (m), 500 (w).

#### Synthesis of [Sb­(3-py)_3_·(ZnSal-*^t^* Bu)_3_] (2·ZnSal-^*t*^Bu)

A narrow Schlenk tube was charged with Sb­(3-py)_3_ (9.6 mg, 0.027 mmol) and ZnSal-^*t*^Bu (40 mg, 0.081 mmol), and 3 mL of CH_2_Cl_2_ was then added. The resulting orange solution was stirred for 10 min at room temperature. Slow diffusion of *n*-hexane (15 mL) at room temperature yielded **2·**ZnSal-^*t*^Bu as an orange crystalline solid suitable for X-ray diffraction analysis, which was dried under vacuum. Yield (calculated as [Sb­(3-py)_3_·(ZnSal-*^t^* Bu)_3_] based on elemental analysis that indicates complete solvent loss from the crystal): 21.2 mg (0.012 mmol, 43%). ^**1**^ **H NMR (298 K, CDCl**_**3**_, **500 MHz):** δ 8.62 (s, 6H, H_10_), δ 8.17 (m, 6H, H_2_ + H_6_), δ 7.44 (s, 6H, H_7_), δ 7.24 (m, 6H, H_14_), δ 7.19 (s, 6H, H_8_), δ 7.12 (d, ^3^ *J* = 7.21 Hz, 3H, H_4_), δ 7.00 (d, ^3^ *J* = 7.29 Hz, 6H, H_12_), δ 6.84 (m, 3H, H_5_), δ 6.42 (t, ^3^ *J* = 7.29 Hz, 6H, H_13_), δ 1.36 (s, 54H, H_18_). ^**13**^ **C­{** ^**1**^ **H} NMR (298 K, CDCl**_**3**_, **126 MHz):** δ 172.78 (s, C_16_), δ 162.51 (s, C_10_), δ 153.70 (s, C_2_py), δ 149.10 (s, C_6_py), δ 145.29 (s, C_4_py), δ 142.95 (s, C_15_), δ 140.08 (s, C_9_), δ 134.19 (s, C_12_), δ 132.63 (s, C_3_py), δ 131.42 (s, C_14_), δ 127.08 (s, C_8_), δ 126.06 (s, C_5_py), δ 119.64 (s, C_11_), δ 115.59 (s, C_7_), δ 113.22 (s, C_13_), δ 35.57 (s, C_17_), δ 29.74 (s, C_18_). **Elemental analysis** (%) calculated for **2·**ZnSal-^*t*^Bu (C_99_H_102_N_9_O_6_SbZn_3_): C, 64.9; H, 5.6; N, 6.9. Found: C, 64.4; H, 5.8; N, 6.4. **HR-MS** (ESI, positive ion mode ESI-QTOF): *m*/*z* for C_99_H_103_N_9_O_6_Sb_1_Zn_3_ [**2·**ZnSal-^*t*^Bu + H]^+^ calculated: 1832.4917. Found: 1832.4905 (−1.2 ppm error). **FT-IR** (ATR, ν/cm^–1^): 2944 (w), 1604 (s), 1581 (s), 1529 (s), 1429 (m), 1385 (m), 1179 (s), 1144 (m), 749 (vs), 531 (m), 501 (w).

#### Synthesis of [N­(3-py)_3_·(ZnSal-*^t^* Bu)_3_] (3·ZnSal-^*t*^Bu)

A narrow Schlenk tube was charged with N­(3-py)_3_ (5 mg, 0.020 mmol) and ZnSal-^*t*^Bu (29.5 mg, 0.060 mmol), and 1.5 mL of CH_2_Cl_2_ was then added. The resulting orange solution was stirred for 10 min at room temperature. Slow diffusion of *n-*hexane (10 mL) at room temperature yielded **3**·ZnSal-^*t*^Bu as orange blocks suitable for X-ray diffraction analysis, which were dried under vacuum. Yield (calculated as [N­(3-py)_3_·(ZnSal-^t^Bu)_3_] based on elemental analysis that indicates complete solvent loss from the crystal): 31.3 mg (0.018 mmol, 90%).^**1**^ **H NMR (298 K, CDCl**_**3**_, **500 MHz):** δ 8.60 (s, 6H, H_10_), δ 7.92 (d, ^3^ *J* = 5.0 Hz, 3H, H_6_), δ 7.71 (d, ^4^ *J* = 2.7 Hz, 3H, H_2_), δ 7.45 (m, 6H, H_8_), δ 7.23 (m, 12H, H_7_ + H_14_), δ 6.94 (d, ^3^ *J* = 7.4 Hz, 6H, H_12_), δ 6.91 (d, ^3^ *J* = 5.0 Hz, 3H, H_4_), δ 6.80 (m, 3H, H_5_), δ 6.39 (t, ^3^ *J* = 7.4 Hz, 6H, H_13_), δ 1.30 (s, 54H, H_18_). ^**13**^ **C­{** ^**1**^ **H} NMR (298 K, CDCl**_**3**_, **126 MHz):** δ 172.90 (s, C_16_), δ 162.39 (s, C_10_), δ 144.20 (s, C_6_py), δ 143.32 (s, C_2_py), δ 142.78 (s, C_15_), δ 142.01 (s, C_3_py), δ 140.02 (s, C_9_), δ 134.26 (s, C_12_), δ 132.20 (s, C_4_py), δ 131.29 (s, C_14_), δ 127.08 (s, C_7_), δ 125.79 (s, C_5_py), δ 119.36 (s, C_11_), δ 115.62 (s, C_8_), δ 113.02 (s, C_13_), δ 35.45 (s, C_17_), δ 29.54 (s, C_18_). **Elemental analysis** (%) calculated for {N­(3-py)_3_·(ZnSal-^*t*^Bu)_3_} (C_99_H_102_N_10_O_6_Zn_3_): C, 69.0; H, 6.0; N, 8.1. Found: C, 68.5; H, 6.1; N, 8.1. **HR-MS** (ESI, positive ion mode ESI-QTOF): *m*/*z* for C_99_H_103_N_10_O_6_Zn_3_ [**3·**ZnSal-^*t*^Bu + H]^+^ calculated: 1723.5913. Found: 1723.5984 (7.1 ppm error). **FT-IR** (ATR, ν/cm^–1^): 2956 (w), 1600 (s), 1579 (s), 1526 (s), 1426 (m), 1382 (m), 1260 (s), 1176 (m), 1083 (s), 798 (s), 744 (vs), 528 (m), 492 (w).

#### Synthesis of ZnSal-*^t^* Bu-Br

A solution of 4,5-dibromo-1,2-phenylenediamine (0.41 g, 1.6 mmol), 3-*tert*-butyl-2-hydroxybenzaldehyde (0.5 mL, 3.1 mmol), Zn­(OAc)_2_·2H_2_O (0.68 g, 3.1 mmol) and neat triethylamine (3 mL, 21.5 mmol) in 40 mL of MeOH (40 mL) was stirred for 20 min at room temperature. The desired compound was isolated by filtration, washed with cold MeOH (3 × 20 mL) and dried in vacuo to yield a yellow solid. Yield: 0.79 g (1.2 mmol, 78%).^**1**^ **H NMR (298 K, DMSO-*d***_**6**_, **500 MHz):** δ 9.08 (s, 2H, H_4_), δ 8.34 (s, 2H, H_2_), δ 7.31 (d, ^3^J= 7.5 Hz, 2H, H_6_), δ 7.24 (d, ^3^ *J* = 7.5 Hz, 2H, H_8_), δ 6.46 (t, ^3^ *J* = 7.5 Hz, 2H, H_7_), δ 1.46 (s, 18H, H_12_). ^**13**^ **C­{** ^**1**^ **H} NMR (298 K, DMSO-*d***_**6**_, **126 MHz):** δ 172.61 (s, C_10_), δ 163.90 (s, C_4_), δ 141.65 (s, C_9_), δ 139.89 (s, C_3_), δ 134.74 (s, C_6_), δ 130.95 (s, C_8_), δ 121.41 (s, C_1_), δ 121.20 (s, C_2_), δ 119.40 (s, C_5_), δ 112.57 (s, C_7_), δ 35.04 (s, C_11_), δ 29.48 (s, C_12_). **HR-MS** (ESI, positive ion mode ESI-QTOF): *m*/*z* for C_28_H_29_Br_2_N_2_O_2_Zn [ZnSal-*^t^* Bu-Br + H]^+^ calculated: 650.9840. Found: 650.9828 (−1.2 ppm error); *m*/*z* for C_28_H_28_Br_2_N_2_O_2_ZnNa [ZnSal-*^t^* Bu-Br + Na]^+^ calculated: 672.9660. Found: 672.9671 (1.1 ppm error).

#### Synthesis of [Bi­(3-py)_3_·(ZnSal-*^t^* Bu-Br)_3_] (1·ZnSal-*^t^* Bu-Br)

A Schlenk tube was charged with Bi­(3-py)_3_ (9.0 mg, 0.020 mmol) and ZnSal-*^t^* Bu-Br (40 mg, 0.061 mmol), and 4 mL of toluene was then added. The resulting brown suspension was stirred for 10 min at room temperature, and it was then filtered with a syringe filter into a narrow Schlenk flask to yield an orange solution. Slow diffusion of *n*-hexane (18 mL) at room temperature yielded **1·**ZnSal-*^t^* Bu-Br as an orange crystalline solid suitable for X-ray diffraction analysis, which was dried under vacuum. Yield (calculated as [Bi­(3-py)_3_·(ZnSal-*^t^* Bu-Br)_3_] based on elemental analysis that indicates complete solvent loss from the crystal): 31.9 mg (0.013 mmol, 65%). ^**1**^ **H NMR (298 K, CDCl**_**3**_, **500 MHz):** δ 8.59 (s, 6H, H_10_), δ 8.35 (s, 3H, H_2_), δ 8.25 (d, ^3^ *J* = 4.55 Hz, 3H, H_6_), δ 7.73 (s, 6H, H_8_), δ 7.58 (d, ^3^ *J* = 7.71 Hz, 3H, H_4_), δ 7.29 (dd, *J* = 7.29/1.72 Hz, 6H, H_14_), δ 7.04 (m, 9H, H_5_+H_12_), δ 6.46 (t, ^3^ *J* = 7.29 Hz, 6H, H_13_), δ 1.35 (s, 54H, H_18_). ^**13**^ **C­{** ^**1**^ **H} NMR (298 K, CDCl**_**3**_, **126 MHz):** δ 173.69 (s, C_16_), δ 162.89 (s, C_10_), δ 154.74 (s, C_2_py), δ 149.90­(s, C_3_py), δ 148.25 (s, C_6_py), δ 147.12 (s, C_4_py), δ 143.14 (s, C_15_), δ 140.26 (s, C_9_), δ 134.42 (s, C_12_), δ 132.09 (s, C_14_), δ 128.04 (s, C_5_py), δ 122.29 (s, C_7_), δ 120.56 (s, C_8_), δ 119.24 (s, C_11_), δ 113.61 (s, C_13_), δ 35.54 (s, C_17_), δ 29.61 (s, C_18_). **Elemental analysis** (%) calculated for **1·**ZnSal-^t^Bu-Br (C_99_H_96_Br_6_N_9_O_6_BiZn_3_): C, 49.7; H, 4.1; N, 5.3. Found: C, 49.5; H, 4.2; N, 5.2. **HR-MS** (ESI, positive ion mode ESI-QTOF): *m*/*z* for C_99_H_97_Br_6_N_9_O_6_BiZn_3_ [**1·**ZnSal-^t^Bu-Br + H]^+^ calculated: 2394.0244. Found: 2394.0299 (5.5 ppm error). **FT-IR** (ATR, ν/cm^–1^): 2948 (w), 1594 (vs), 1521 (s), 1464 (m), 1427 (s), 1381 (m), 1178 (s), 1141 (s), 864 (m), 745 (m), 699 (w), 354 (w), 255 (m).

#### Synthesis of [Sb­(3-py)_3_·(ZnSal-*^t^* Bu-Br)_3_] (2·ZnSal-*^t^* Bu-Br)

A Schlenk tube was charged with Sb­(3-py)_3_ (9.2 mg, 0.026 mmol) and ZnSal-*^t^* Bu-Br (50 mg, 0.077 mmol), and 2.5 mL of CH_2_Cl_2_ was then added. The resulting brown suspension was stirred for 10 min at room temperature, and it was then filtered with a syringe filter into a narrow Schlenk flask to yield an orange solution. Slow diffusion of *n-*hexane (15 mL) at room temperature yielded **2·**ZnSal-*^t^* Bu-Br as an orange crystalline solid suitable for X-ray diffraction analysis, which was dried under vacuum. Yield (calculated as [Sb­(3-py)_3_·(ZnSal-*^t^* Bu-Br)_3_] based on elemental analysis that indicates complete solvent loss from the crystal): 36.7 mg (0.016 mmol, 78%). ^**1**^ **H NMR (298 K, CDCl**_**3**_, **500 MHz):** δ 8.58 (s, 6H, H_10_), δ 8.19 (s, 3H, H_2_), δ 8.16 (d, ^3^ *J* = 4.83 Hz, 3H, H_6_), δ 7.73 (s, 6H, H_8_), δ 7.27 (m, 6H, H_14_), δ 7.17 (d, ^3^ *J* = 7.56 Hz, 3H, H_4_), δ 7.01 (d, ^3^ *J* = 7.64 Hz, 6H, H_12_), δ 6.95 (m, 3H, H5), δ 6.46 (t, ^3^ *J* = 7.64 Hz, 6H, H_13_), δ 1.32 (s, 54H, H_18_). ^**13**^ **C­{** ^**1**^ **H} NMR (298 K, CDCl**_**3**_, **126 MHz):** δ 173.66 (s, C_16_), δ 162.89 (s, C_10_), δ 153.85 (s, C_2_py), δ 149.07 (s, C_6_py), δ 145.29 (s, C_4_py), δ 143.15 (s, C_15_), δ 140.19 (s, C_9_), δ 134.42 (s, C_12_), δ 132.62 (s, C_3_py), δ 132.13 (s, C_14_), δ 126.20 (s, C_5_py), δ 122.33 (s, C_7_), δ 120.55 (s, C_8_), δ 119.22 (s, C_11_), δ 113.66 (s, C_13_), δ 35.52 (s, C_17_), δ 29.59 (s, C_18_). **Elemental analysis** (%) calculated for **2·**ZnSal-^t^Bu-Br (C_99_H_96_Br_6_N_9_O_6_SbZn_3_): C, 51.6; H, 4.2; N, 5.5. Found: C, 51.9; H, 4.4; N, 5.2. **HR-MS** (ESI, positive ion mode ESI-QTOF): *m*/*z* for C_99_H_96_Br_6_N_9_O_6_SbZn_3_Na [**2·**ZnSal-^t^Bu-Br + Na]^+^ calculated: 2327.9305. Found: 2327.9302 (−0.3 ppm error). **FT-IR** (ATR, ν/cm^–1^): 2954 (w), 1595 (vs), 1525 (vs), 1468 (m), 1425 (s), 1355 (m), 1179 (s), 1142 (vs), 866 (m), 749 (s), 699 (m), 547 (w), 446 (w), 354 (w).

#### Synthesis of [N­(3-py)_3_·(ZnSal-*^t^* Bu-Br)_3_] (3·ZnSal-*^t^* Bu-Br)

A Schlenk tube was charged with N­(3-py)_3_ (6.5 mg, 0.026 mmol) and ZnSal-*^t^* Bu-Br (50 mg, 0.077 mmol), and 2 mL of CH_2_Cl_2_ was then added. The resulting brown suspension was stirred for 10 min at room temperature, and it was then filtered with a syringe filter into a narrow Schlenk flask to yield an orange solution. Slow diffusion of *n*-hexane (15 mL) at room temperature yielded **3·**ZnSal-*^t^* Bu-Br as an orange crystalline solid suitable for X-ray diffraction analysis, which was dried under vacuum. Yield (calculated as [N­(3-py)_3_·(ZnSal-*^t^* Bu-Br)_3_] based on elemental analysis that indicates complete solvent loss from the crystal): 22.6 mg (0.010 mmol, 39%). ^**1**^ **H NMR (298 K, CDCl**_**3**_, **500 MHz):** δ 8.54 (s, 6H, H_10_), δ 7.91 (s, 3H, H_6_), δ 7.79 (s, 3H, H_2_), δ 7.74 (s, 6H, H_8_), δ 7.27 (m, 6H, H_14_), δ 6.96 (m, 9H, H_4_ + H_12_), δ 6.89 (m, 3H, H_5_), δ 6.44 (t, ^3^ *J* = 7.56 Hz, 6H, H_13_), δ 1.29 (s, 54H, H_18_). ^**13**^ **C­{** ^**1**^ **H} NMR (298 K, CDCl**_**3**_, **126 MHz):** δ 173.62 (s, C_16_), δ 162.83 (s, C_10_), δ 144.34 (s, C_6_py), δ 143.64 (s, C_2_py), δ 143.02 (s, C_15_), δ 142.20 (s, C_3_py), δ 140.14 (s, C_9_), δ 134.47 (s, C_12_), δ 132.15 (s, C_14_), δ 132.01 (s, C_4_py), δ 125.75 (s, C_5_py), δ 122.38 (s, C_7_), δ 120.51 (s, C_8_), δ 119.13 (s, C_11_), δ 113.60 (s, C_13_), δ 35.48 (s, C_17_), δ 29.53 (s, C_18_). **Elemental analysis** (%) calculated for **3·**ZnSal-*^t^* Bu-Br (C_99_H_96_Br_6_N_10_O_6_Zn_3_): C, 54.1; H, 4.4; N, 6.4. Found: C, 55.0; H, 4.6; N, 6.8. **HR-MS** (ESI, positive ion mode ESI-QTOF): *m*/*z* for C_99_H_97_Br_6_N_10_O_6_Zn_3_ [**3·**ZnSal-^t^Bu-Br + H]^+^ calculated: 2199.0471. Found: 2199.0506 (3.5 ppm error). **FT-IR** (ATR, ν/cm^–1^): 2957 (w), 1596 (vs), 1526 (vs), 1428 (s), 1356 (m), 1180 (s), 1144 (s), 866 (m), 866 (m), 802 (m), 751 (s), 410 (w).

#### Synthesis of {[Bi­(3-py)_3_]_6_·(MgSal-*^t^* Bu)_10_} (1·MgSal-^*t*^Bu)

Mg­(*^n^* Bu)_2_ 0.5 M (180 μL, 0.09 mmol) was added dropwise to a yellowish solution of salphen 2H-Sal-*^t^* Bu (40 mg, 0.09 mmol) in dry CH_2_Cl_2_ (3 mL) under a nitrogen atmosphere. The resulting mixture was stirred for 30 min, then added to solid Bi­(3-py)_3_ (22 mg, 0.05 mmol) and stirred for 10 min. Slow diffusion of dry *n*-hexane (15 mL) at room temperature yielded **1**·MgSal-^*t*^Bu as orange crystals suitable for X-ray diffraction analysis, which were dried under vacuum. Yield (calculated as {[Bi­(3-py)_3_]_6_·(MgSal-*^t^* Bu)_10_}): 19 mg (0.0027 mmol, 32%). δ 8.67 (s, 20H, H_10_), δ 8.46 (s, 18H, H_2_), δ 8.43 (d, ^3^ *J* = 4.00 Hz, 18H, H_6_), δ 7.74 (d, ^3^ *J* = 7.50 Hz, 18H, H_4_), δ 7.49 (s, 20H, H_7_), δ 7.33 (d, ^3^ *J* = 7.55 Hz, 20H, H_14_), δ 7.22 (m, 20H, H_8_), δ 7.13 (m, 38H, H_5_ + H_12_), δ 6.50 (t, ^3^ *J* = 7.55 Hz, 20H, H_13_), δ 1.42 (s, 180H, H_18_). Satisfactory elemental analysis could not be obtained.

#### Synthesis of {[Sb­(3-py)_3_]_6_·(MgSal-*^t^* Bu)_10_} (2·MgSal-^*t*^Bu)

Mg­(*^n^* Bu)_2_ 0.5 M (180 μL, 0.09 mmol) was added dropwise to a yellowish solution of salphen 2H-Sal-*^t^* Bu (40 mg, 0.09 mmol) in dry CH_2_Cl_2_ (3 mL) under a nitrogen atmosphere. The resulting mixture was stirred for 30 min, then added to solid Sb­(3-py)_3_ (18 mg, 0.05 mmol) and stirred for 10 min. Slow diffusion of dry *n*-hexane (15 mL) at room temperature yielded **2**·MgSal-^*t*^Bu as orange crystals suitable for X-ray diffraction analysis, which were dried under vacuum. Yield (calculated as {[Sb­(3-py)_3_]_6_·(MgSal-*^t^* Bu)_10_}): 23 mg (0.0035 mmol, 42%). δ 8.65 (d, ^4^ *J* = 3.99 Hz, 20H, H_10_), δ 8.39 (d, ^3^ *J* = 4.90 Hz, 18H, H_6_), δ 8.29 (s, 18H, H_2_), δ 7.48 (s, 20H, H_7_), δ 7.38 (d, ^3^ *J* = 7.66 Hz, 18H, H_4_), δ 7.32 (d, ^3^ *J* = 7.55 Hz, 20H, H_14_), δ 7.21 (m, 20H, H_8_), δ 7.11 (d, ^3^ *J* = 7.55 Hz, 20H, H_12_), δ 7.06 (m, 18H, H_5_), δ 6.49 (t, ^3^ *J* = 7.55 Hz, 20H, H_13_), δ 1.41 (s, 180H, H_18_). Satisfactory elemental analysis could not be obtained.

#### Synthesis of {[N­(3-py)_3_]_4_·(MgSal-*^t^* Bu)_6_} (3·MgSal-^*t*^Bu)

Mg­(*^n^* Bu)_2_ 0.5 M (180 μL, 0.09 mmol) was added dropwise to a yellowish solution of salphen 2H-Sal-*^t^* Bu (40 mg, 0.09 mmol) in dry CH_2_Cl_2_ (3 mL) under a nitrogen atmosphere. The resulting mixture was stirred for 30 min, then added to solid N­(3-py)_3_ (14 mg, 0.06 mmol) and stirred for 10 min. Slow diffusion of dry *n*-hexane (15 mL) at room temperature yielded **3**·MgSal-^*t*^Bu as orange crystals suitable for X-ray diffraction analysis, which were dried under vacuum. Yield (calculated as {[N­(3-py)_3_]_4_·(MgSal-*^t^* Bu)_6_}): 12 mg (0.0032 mmol, 21%). ^**1**^ **H NMR (298 K, CDCl**_**3**_, **500 MHz):** δ 8.66 (s, 12H, H_10_), δ 8.19 (d, ^3^ *J* = 4.35 Hz, 12H, H_6_), δ 8.08 (d, ^4^ *J* = 2.43 Hz, 12H, H_2_), δ 7.51 (s, 12H, H_8_), δ 7.32 (d, ^3^ *J* = 7.52 Hz, 12H, H_14_), δ 7.23 (s, 24H, H_7_ + H_4_), δ 7.09 (m, 24H, H_5_ + H_12_), δ 6.48 (t, ^3^ *J* = 7.52 Hz, 12H, H_13_), δ 1.41 (s, 108H, H_18_). Satisfactory elemental analysis could not be obtained.

## Computational Details

### General Methods

All computations were carried out using the Gaussian16 package,^101^ in which the hybrid method of Austin, Petersson, and Frisch with spherical atom dispersion terms (APFD) was applied.^102^ The triple-ζ cc-pVTZ-PP basis set with effective core potentials was used for the heavy atoms (Zn and Bi),^103^ as found in the basis set exchange library, and 6–31G­(d,p) was used for the rest of the atoms (C, N, O, H).^104^

After a single point calculation, a Natural Bonding Orbital (NBO) analysis was performed with the NBO 7.0 program.^105^ The visualization of the calculation results was performed with GaussView 6.1,^106^ and Chemcraft.^107^

QTAIM calculations^108^ and all surface extrema were also evaluated after conducting single point calculations of the structures with Multiwfn 3.8.^109^ Key local maxima located on the extension of the E–C_py_ bond were detected as sigma holes, whereas key local minima were identified as lone pairs after iterative inspection using VMD 1.9.2.^110^

## Supplementary Material



## Notes

Detailed experimental procedures, synthetic protocols, NMR spectra, high-resolution mass data, binding data analysis, DOSY experiments, catalytic experiments, X-ray crystallographic details, and computational calculation details (PDF)
