# Supplementary material for: Sb- and Bi-Based Salphen Capsules: Pnictogen Bonding, Macrocycle Formation, and ON/OFF Catalysis
Source: Inorg Chem. 2026 Sep 29;65(40):23581–97. doi: 10.1021/acs.inorgchem.6c03201 (PMC13664082; doi:10.1021/acs.inorgchem.6c03201)
Supplement: Supplementary file 1 [file ic6c03201_si_001.pdf]

Supporting Information

# Sb- and Bi-Based Salphen Capsules: Pnictogen Bonding, Macrocycle Formation, and ON/OFF Catalysis

Lara Garriel-Muñoz [a], Héctor Barbero [a], Álvaro García-Romero*[a] and Raúl García-Rodríguez*[a]

[a] GIR MIOMeT-IU Cinquima-Química Inorgánica Facultad de Ciencias, Universidad de Valladolid; Campus Miguel Delibes, 47011 Valladolid (Spain). E-mail: raul.garcia.rodriguez@uva.es; alvaro.garcia.romero@uva.es.

# Contents


**NMR studies and spectra** .......... **3**

DOSY experiments .......... 27

Binding data analysis .......... 35

Method of continuous variation (Job plot) .......... 35

$^1$H NMR titrations .......... 39

Variable temperature experiments .......... 47

**Computational details** .......... **50**

NBO analysis .......... 50

QTAIM calculations .......... 51

Effect of coordination on pnictogen bonding .......... 52

**High resolution mass data** .......... **56**

**FT-IR selected spectra** .......... **60**

**X-Ray crystallographic studies** .......... **63**

Single crystal X-Ray diffraction analysis (SCXRD) .......... 63

X-Ray powder diffraction analysis (XRPD) .......... 78

**Proof-of-concept catalytic studies** .......... **79**

General procedure for Bi-catalyzed oxidative cleavage of 1,2-diols .......... 79

General procedure for Sb-catalyzed oxidations of α-hydroxyketones .......... 80

ON/OFF experiments .......... 81

**References** .......... **88**

# NMR studies and spectra

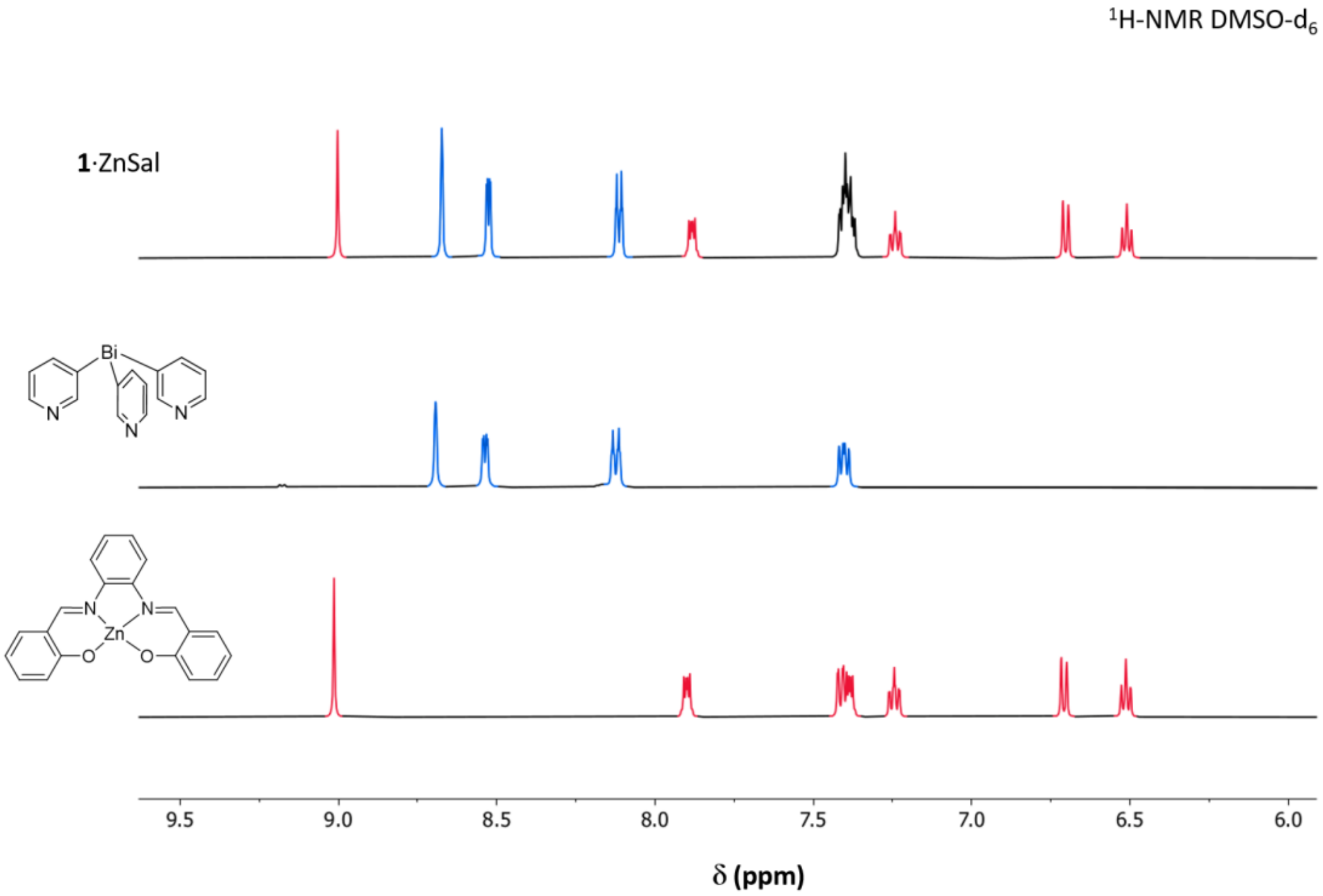


*Figure S1. Stacked $^1$H NMR (DMSO-d$_6$) spectra of **1**·ZnSal (top), Bi(3-py)$_3$ (middle) and ZnSal (bottom), showing coincident peaks between both free species. Note: Some signals are overlapped, and they are therefore not highlighted in red or blue.*

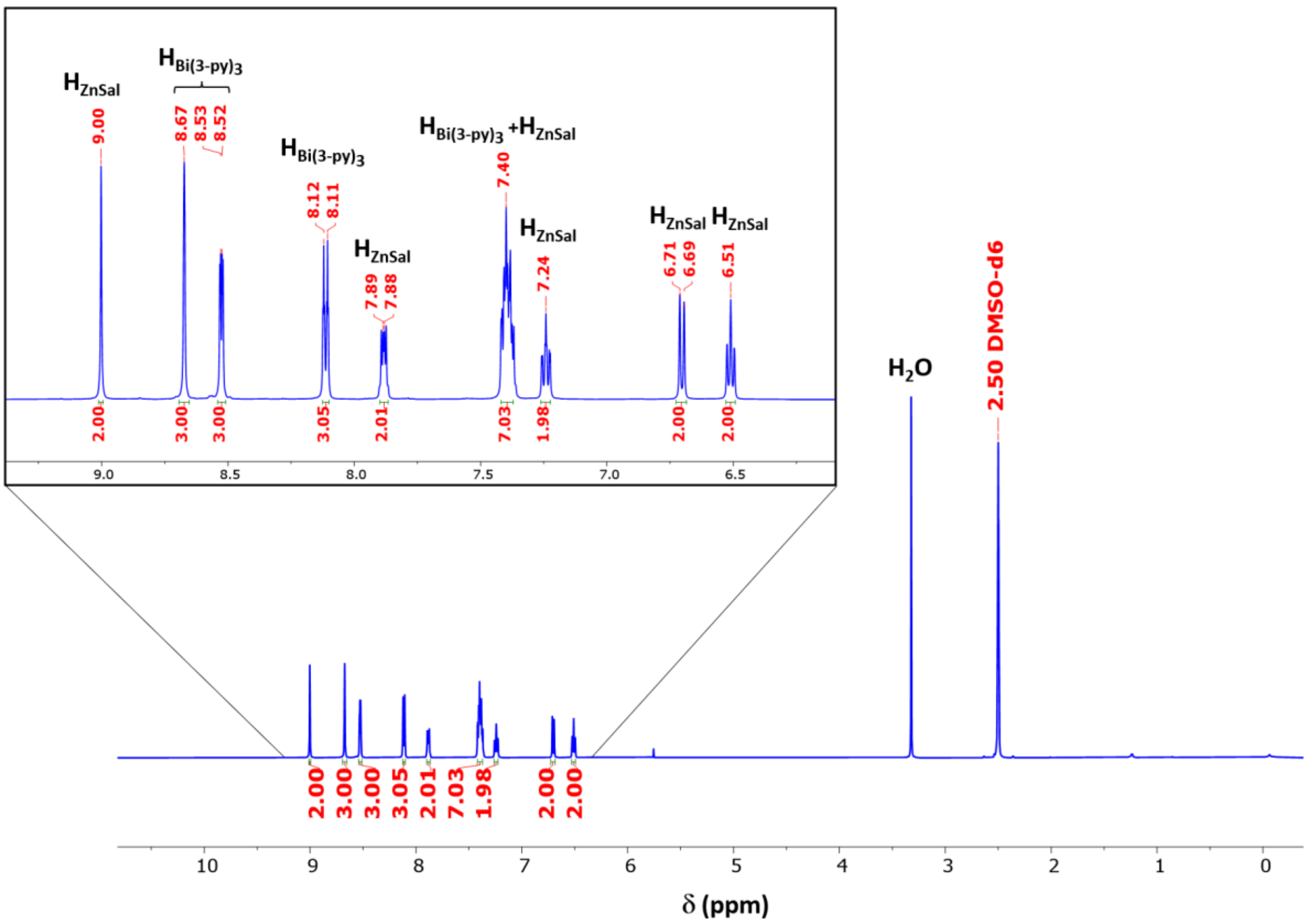


*Figure S2. $^1$H NMR (298 K, DMSO-d$_6$, 500 MHz) spectrum of **1**·ZnSal showing the expected 1:1 ratio.*

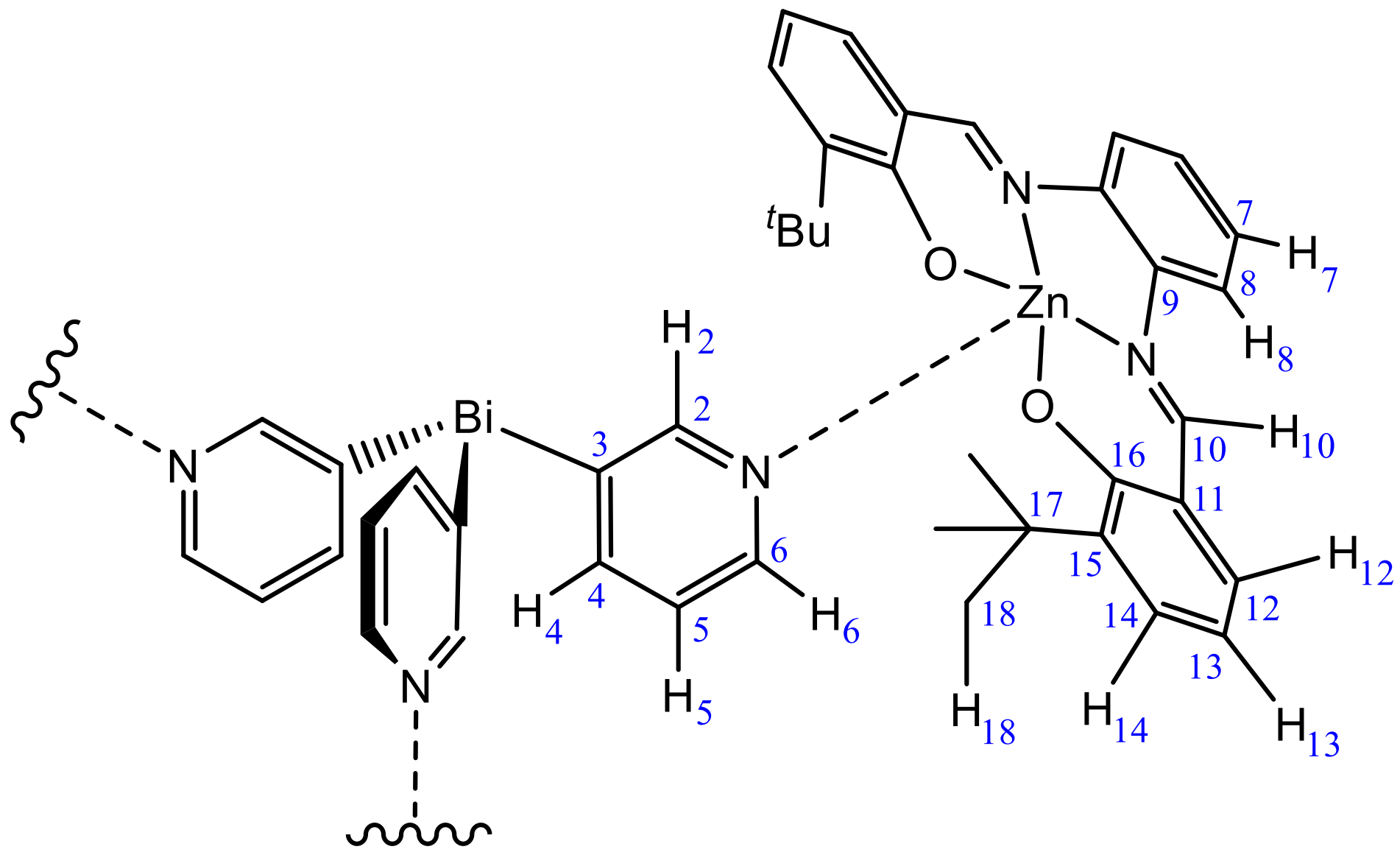

*Figure S3. Compound **1**·ZnSal-$^t$Bu with the atom labelling used in the NMR studies.*

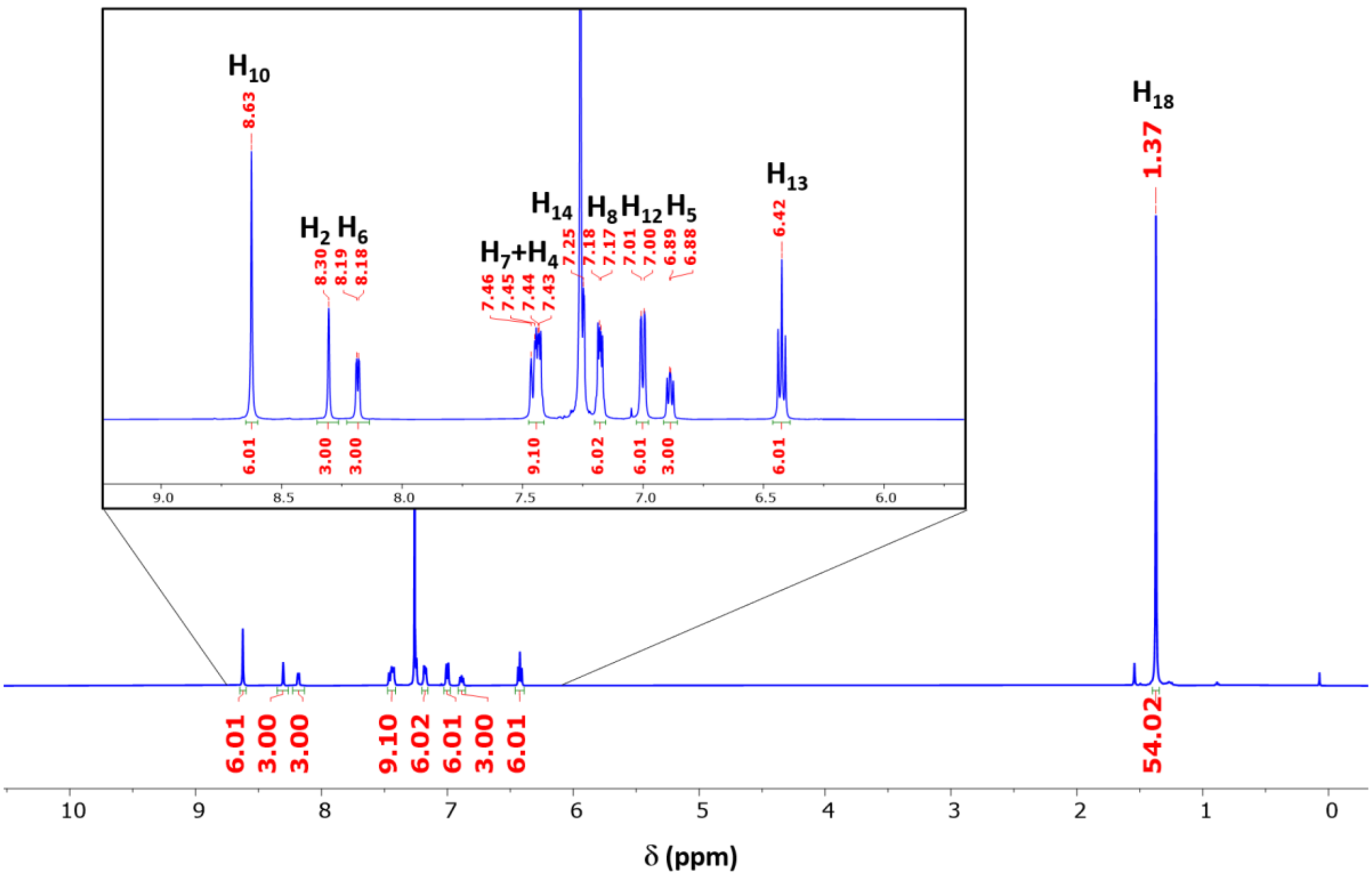

*Figure S4. $^1$H NMR (298 K, $CDCl_3$, 500 MHz) spectrum of **1**·ZnSal-$^t$Bu.*

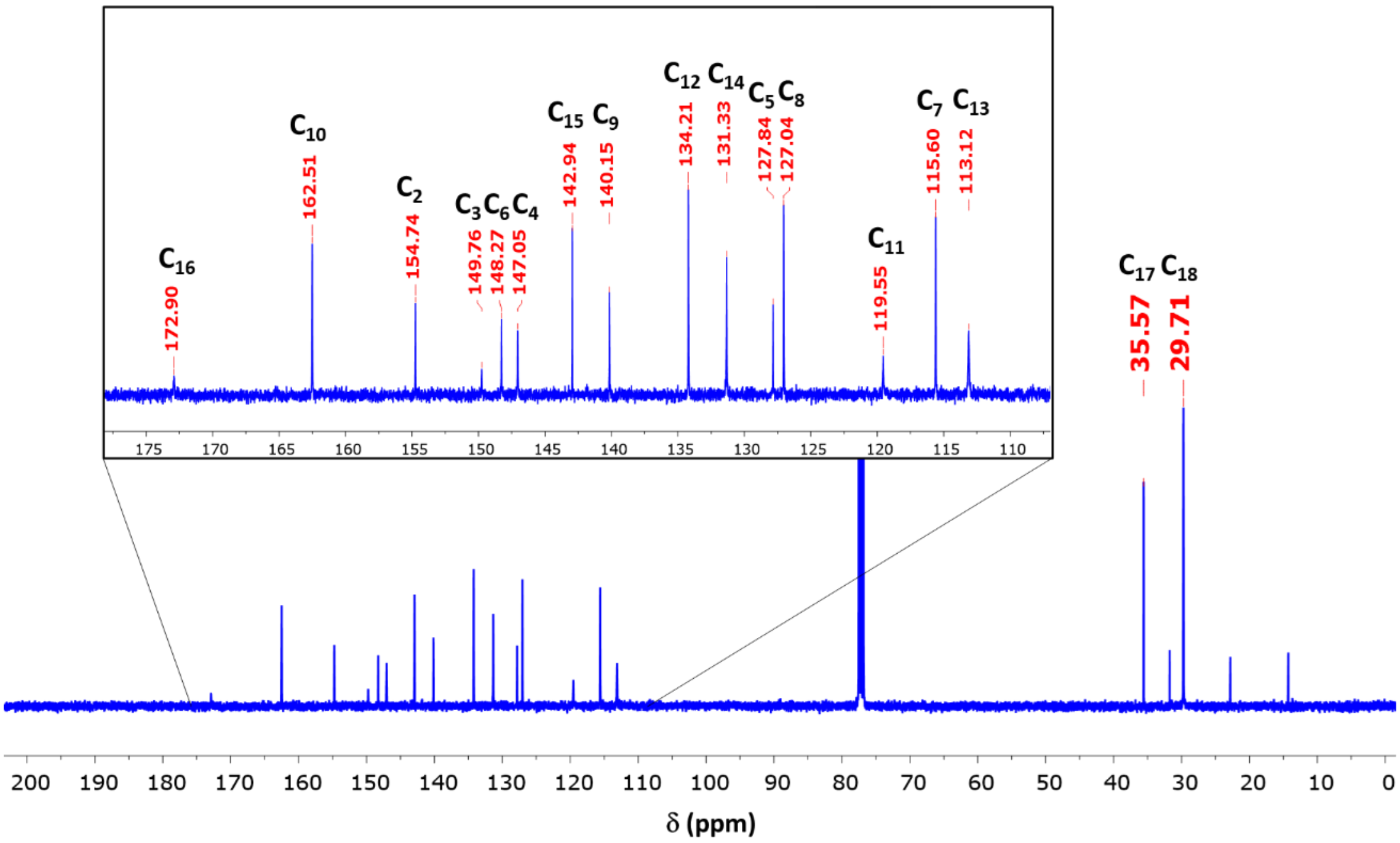


*Figure S5. $^{13}C\{^{1}H\}$-NMR (298 K, $CDCl_3$, 100.5 MHz) spectrum of **1**·ZnSal-$^{t}$Bu.*

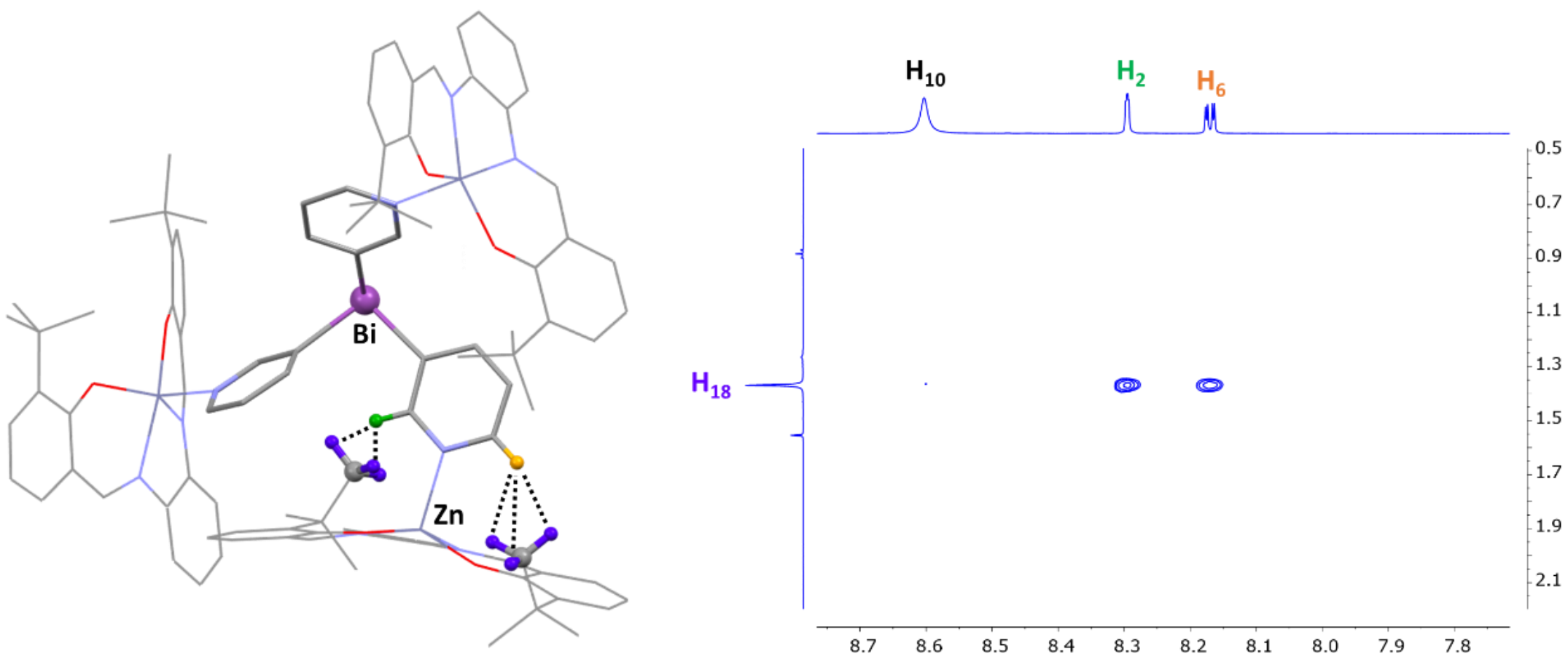


*Figure S6. Selected region of the $^{1}H$–$^{1}H$ ROESY (298 K, $CDCl_3$, mixing time of 400 ms) spectrum of **1**·ZnSal-$^{t}$Bu. Crosspeaks observed between $H_{18}$ and the pyridinic $H_2$, $H_6$ protons arise from intramolecular cross-relaxation of protons that are close to each other in space, confirming the presence of N–Zn linkage in solution.*

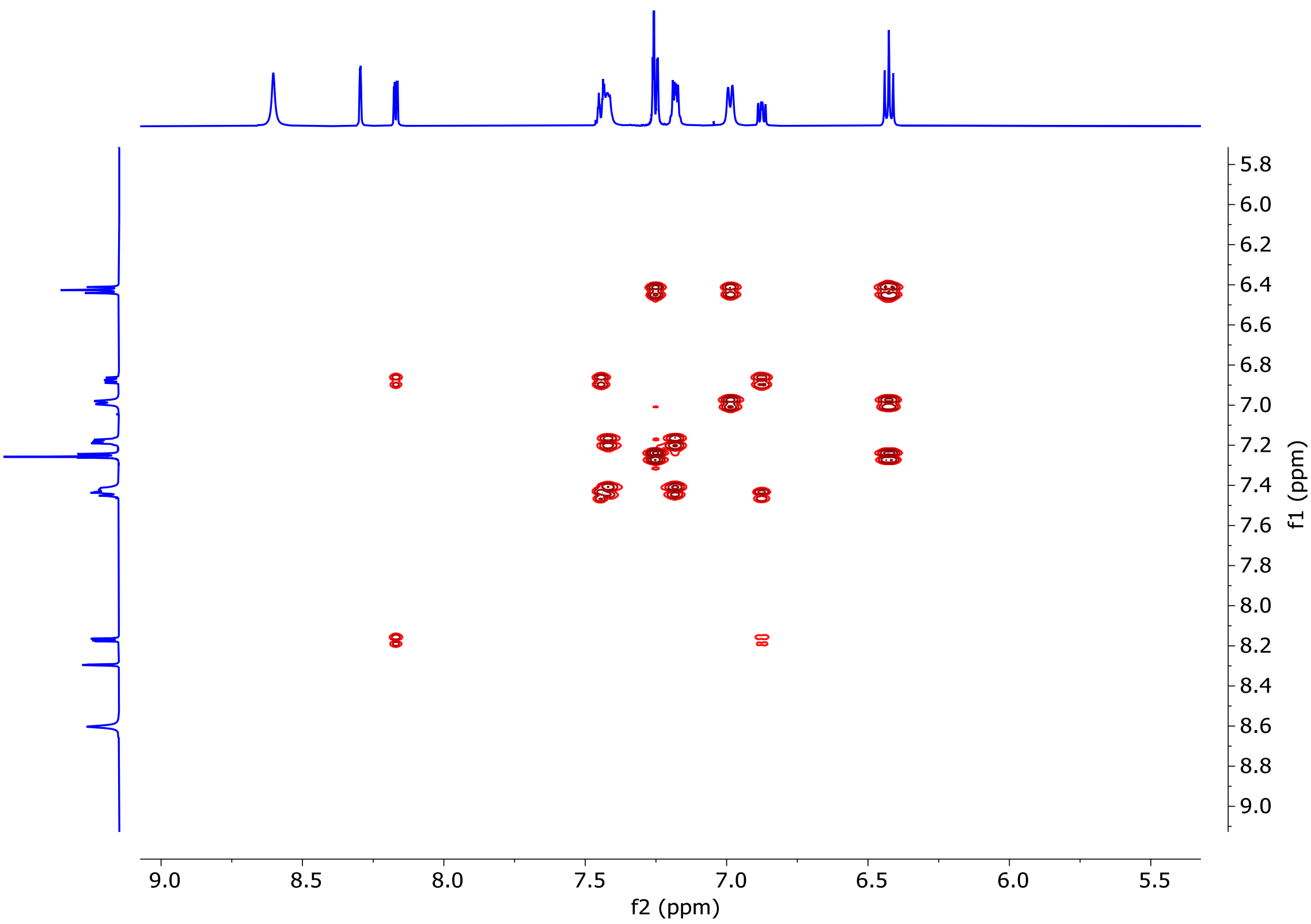


*Figure S7. $^1H$–$^1H$ gCOSY (298 K, $CDCl_3$, 500 MHz) spectrum of **1**·ZnSal-$^t$Bu.*

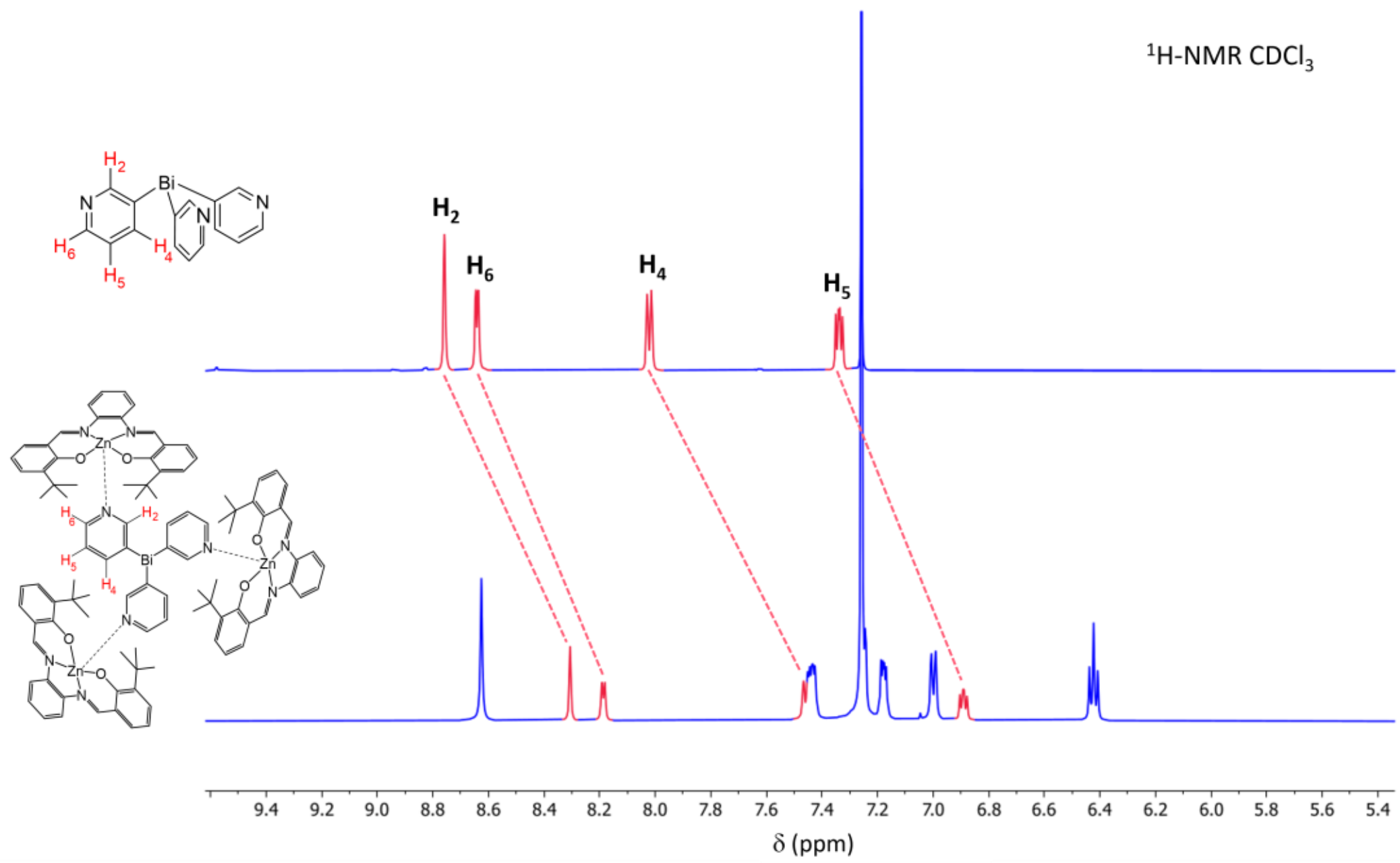


*Figure S8. $^1H$ NMR (298 K, $CDCl_3$, 500 MHz) spectrum of **1** (top) and **1**·ZnSal-$^t$Bu (bottom), showing an upfield shift of the pyridyl proton signals (ca. 0.4 ppm for the ortho protons).*

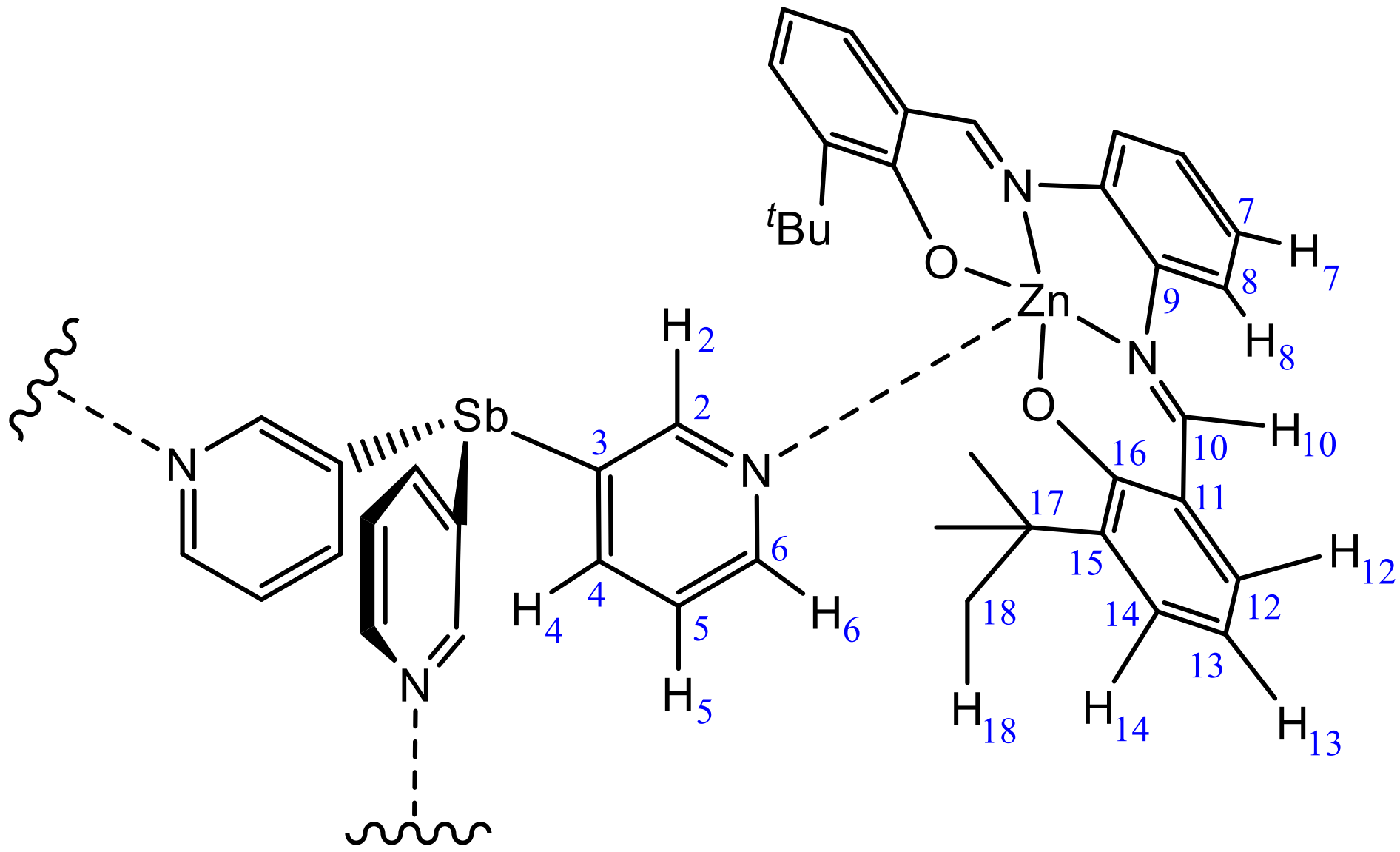


*Figure S9. Compound **2**·ZnSal-tBu with the atom labelling used in the NMR studies.*

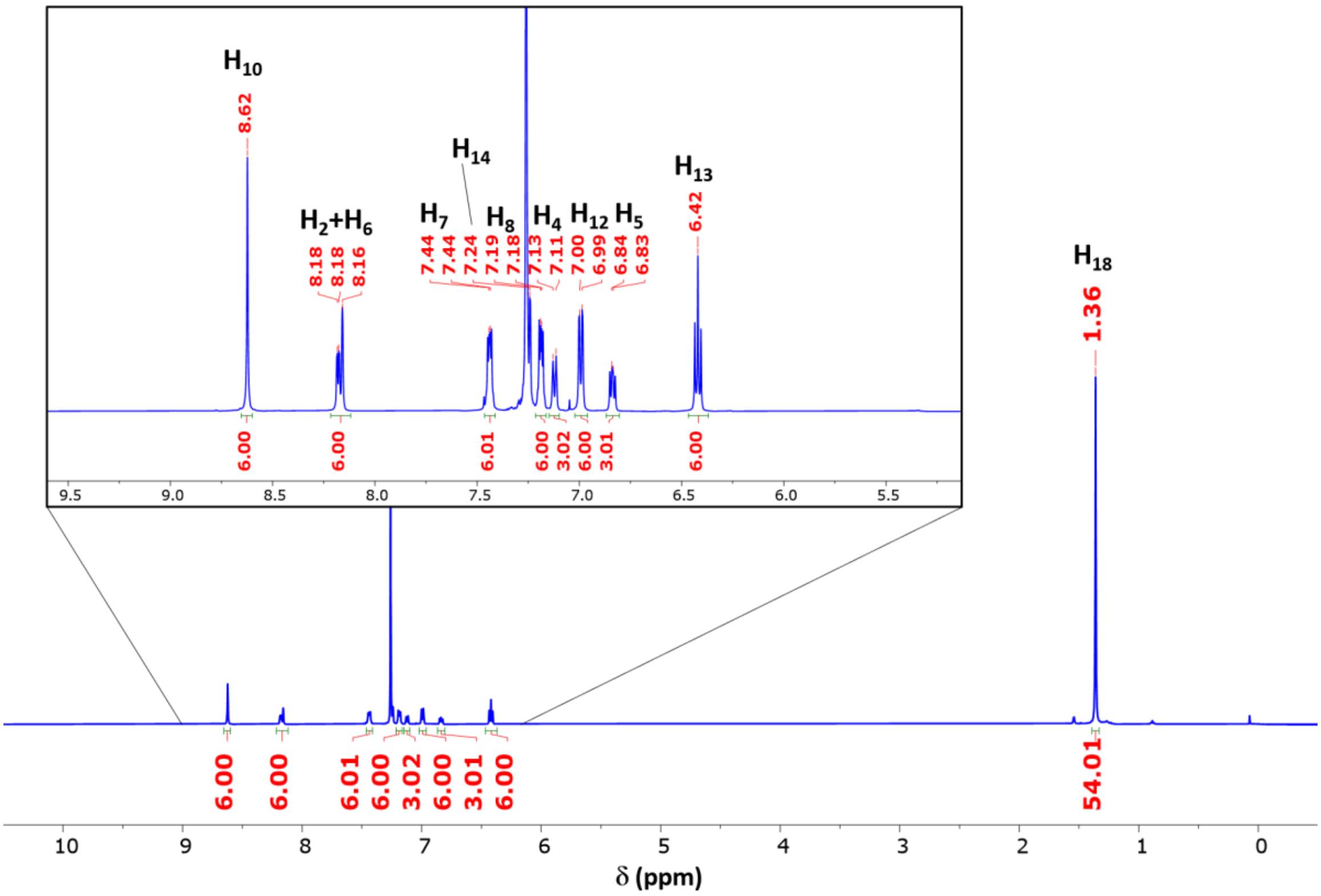


*Figure S10. $^1$H NMR (298 K, $CDCl_3$, 500 MHz) spectrum of **2**·ZnSal-tBu.*

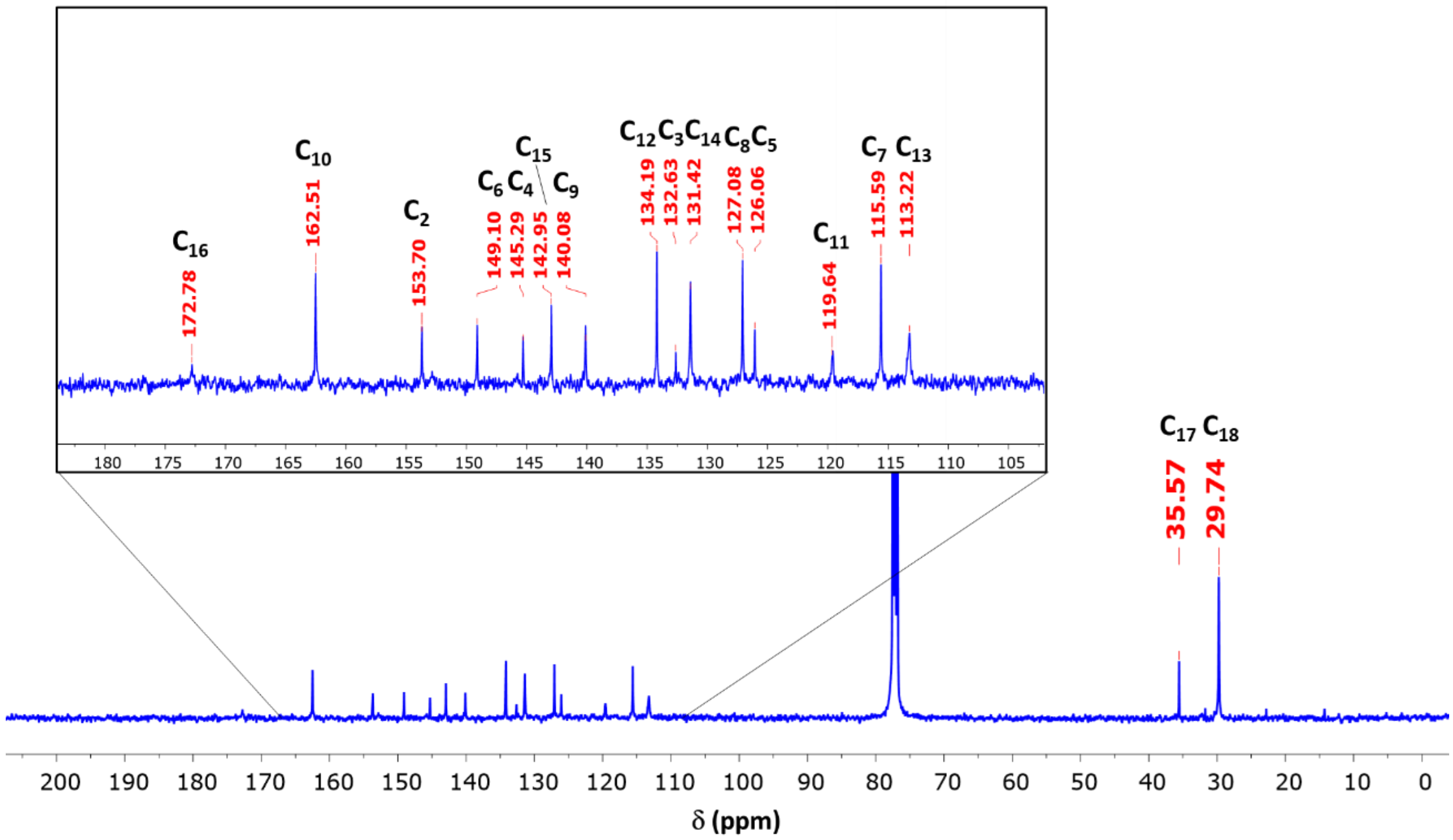


*Figure S11. $^{13}C\{^{1}H\}$-NMR (298 K, $CDCl_3$, 100.5 MHz) spectrum of **2**·ZnSal-$^{t}$Bu.*

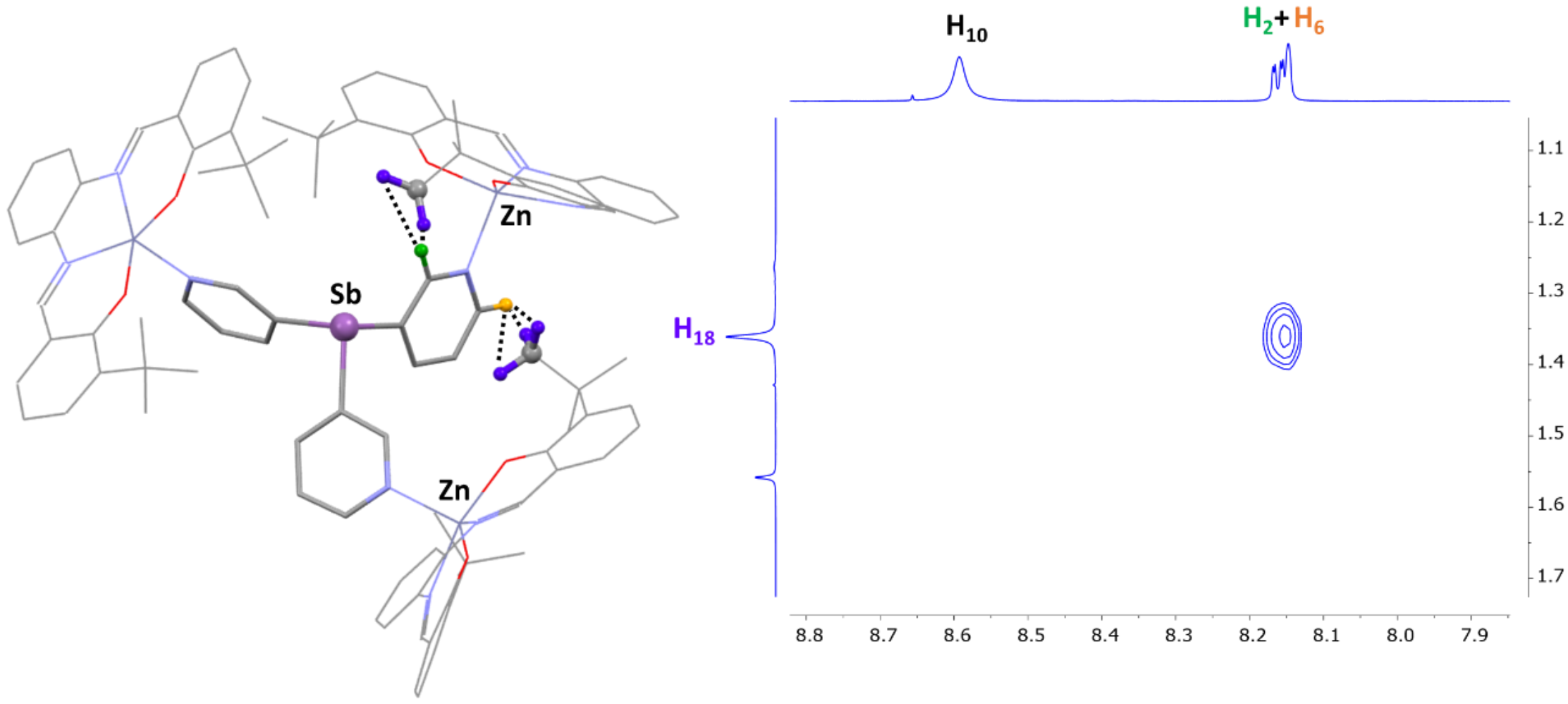


*Figure S12. Selected region of the $^{1}H$–$^{1}H$ ROESY (298 K, $CDCl_3$, mixing time of 400 ms) spectrum of **2**·ZnSal-$^{t}$Bu. Crosspeaks observed between $H_{18}$ and pyridinic $H_2$, $H_6$ protons arise from intramolecular cross-relaxation of protons that are close to each other in space, confirming the presence of N–Zn linkage in solution.*

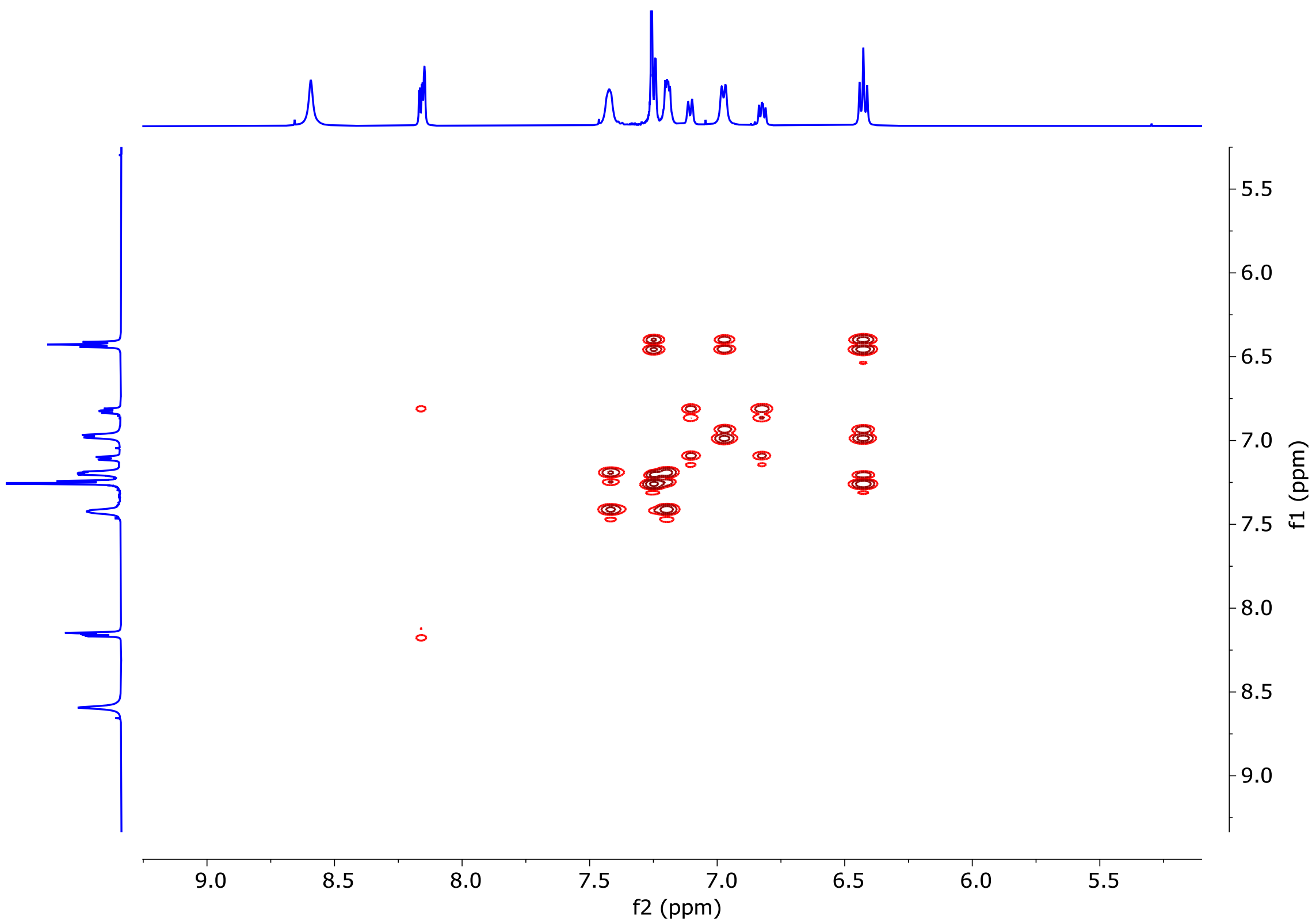


*Figure S13. $^{1}H$–$^{1}H$ gCOSY (298 K, $CDCl_3$, 500 MHz) spectrum of **2**·ZnSal-$^{t}$Bu.*

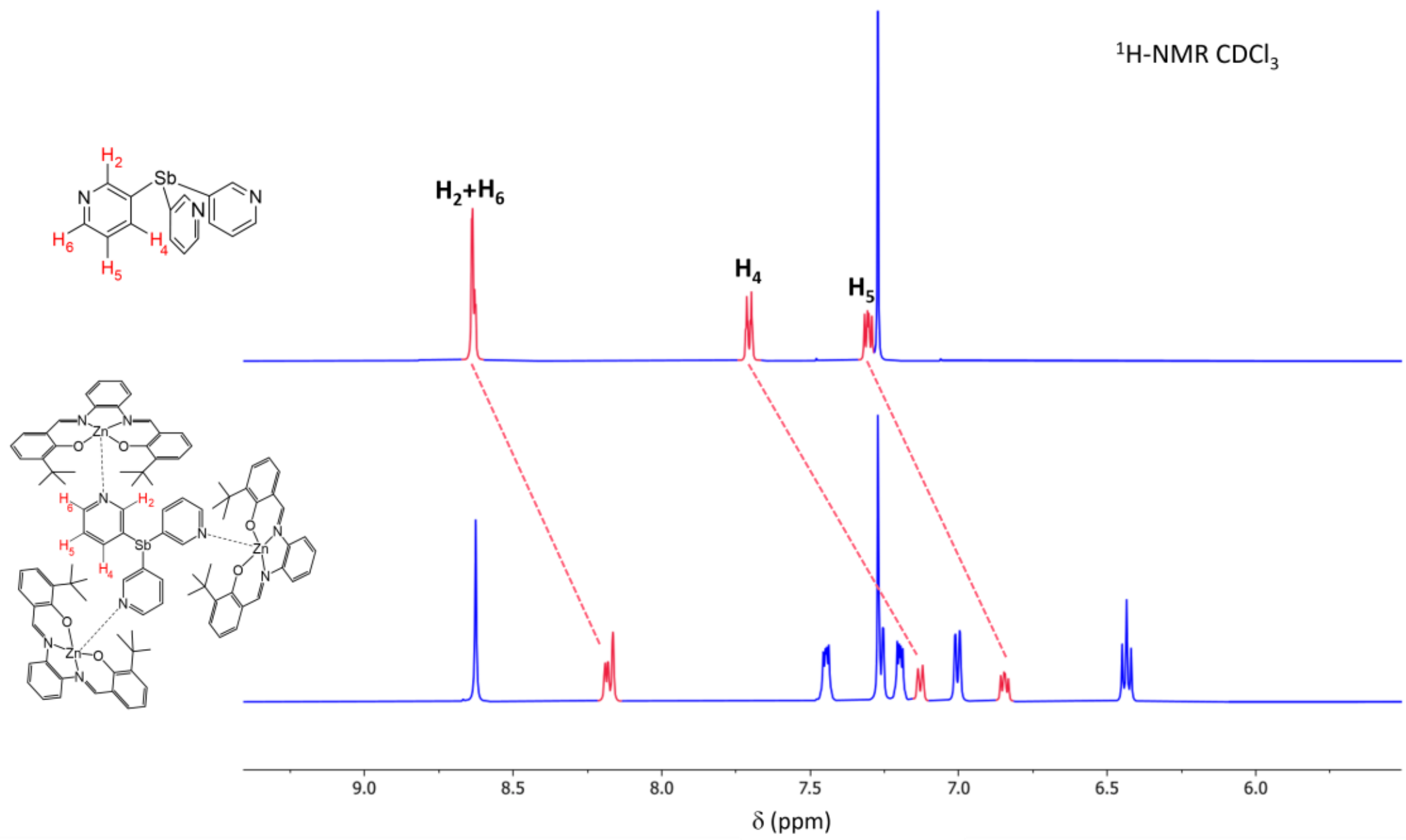


*Figure S14. $^{1}H$ NMR (298 K, $CDCl_3$, 500 MHz) spectrum of **2** (top) and **2**·ZnSal-$^{t}$Bu (bottom), showing an upfield shift of the pyridyl proton signals (ca. 0.4 ppm for the ortho protons).*

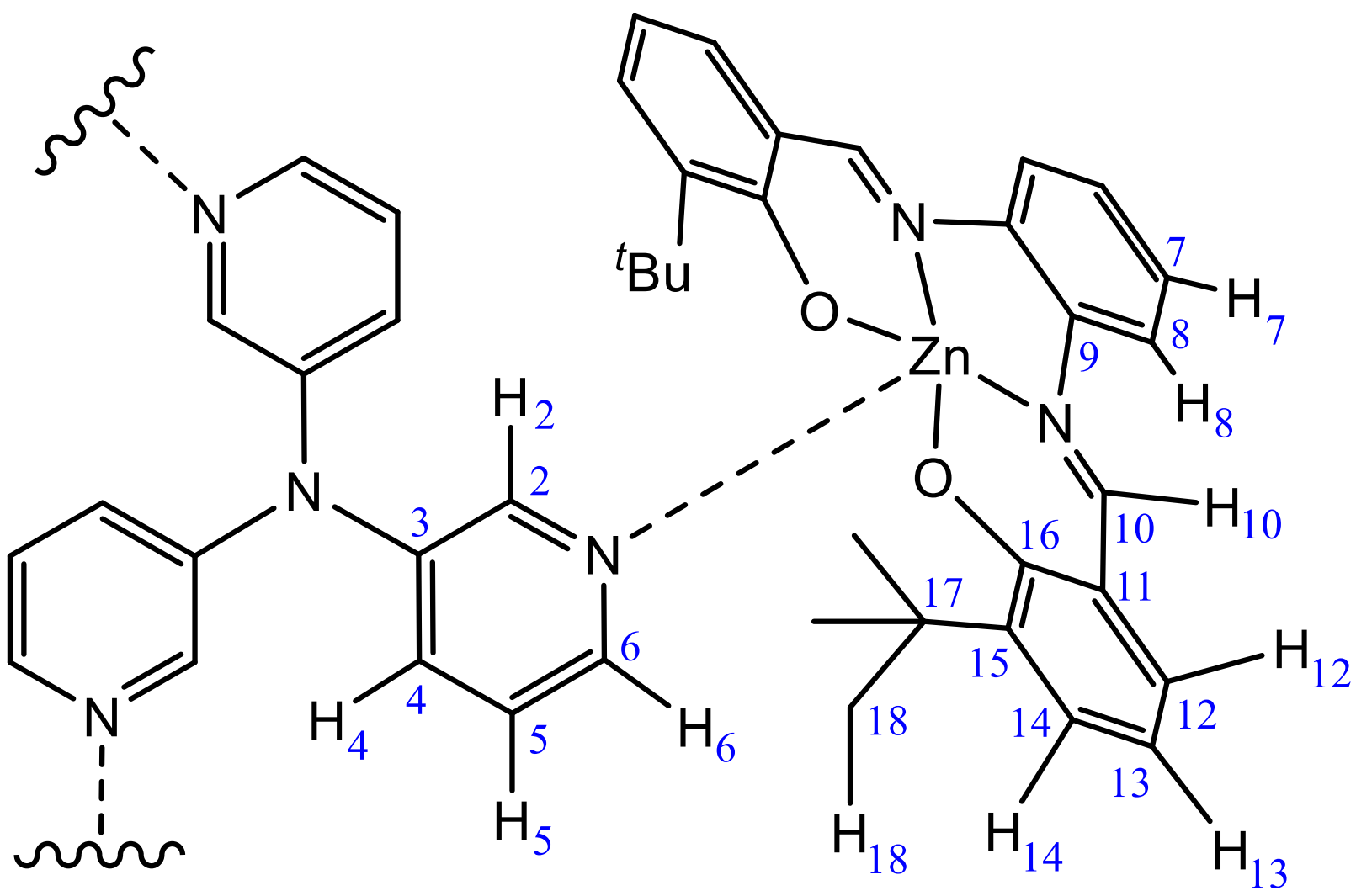

*Figure S15. Compound **3**·ZnSal-$^t$Bu with the atom labelling used in the NMR studies.*

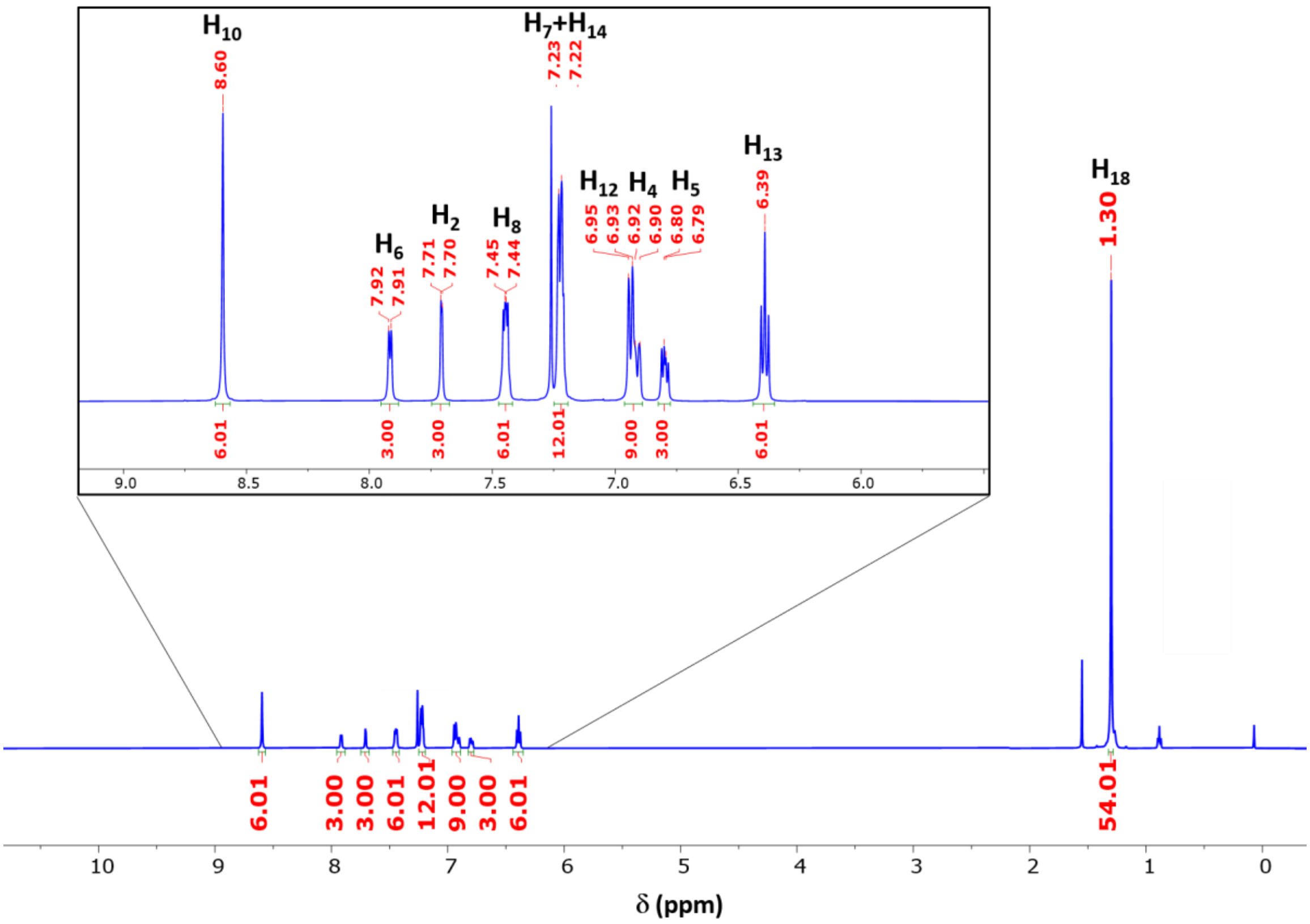


*Figure S16. $^1$H NMR (298 K, $CDCl_3$, 500 MHz) spectrum of **3**·ZnSal-$^t$Bu.*

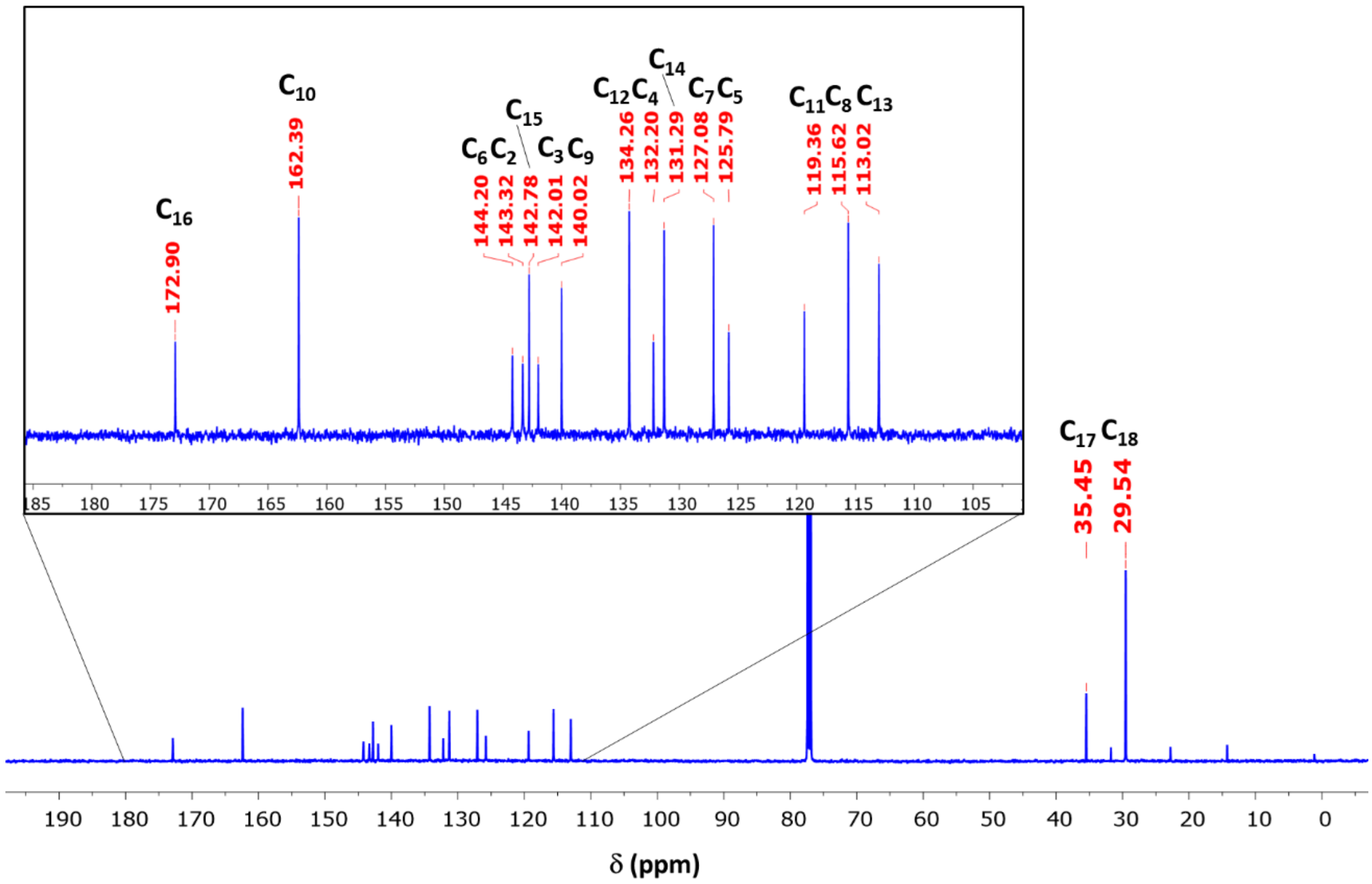


*Figure S17. $^{13}C\{^{1}H\}$-NMR (298 K, $CDCl_3$, 100.5 MHz) spectrum of **3**·ZnSal-$^{t}$Bu.*

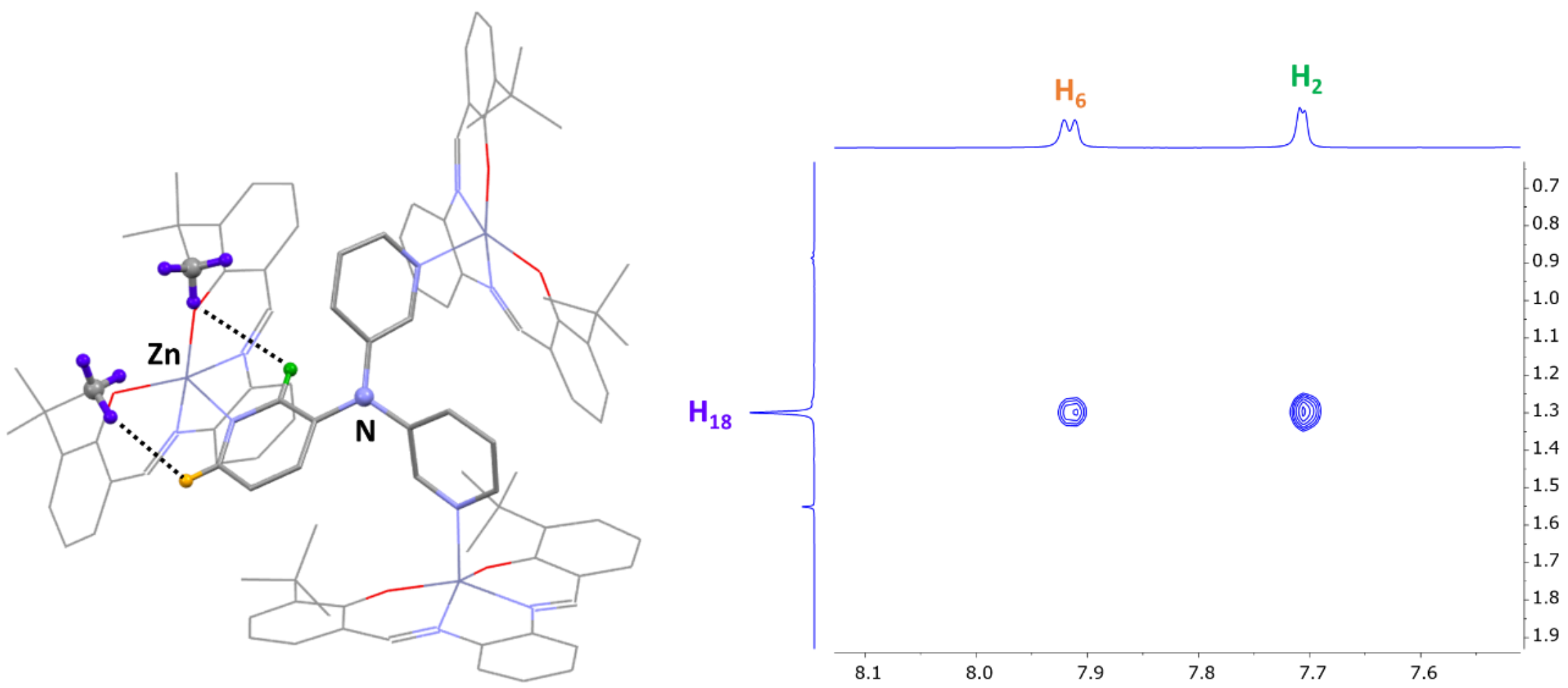


*Figure S18. Selected region of the $^{1}H$–$^{1}H$ NOESY (298 K, $CDCl_3$, mixing time of 800 ms) spectrum of **3**·ZnSal-$^{t}$Bu. Crosspeaks observed between $H_{18}$ and pyridinic $H_2$, $H_6$ protons arise from intramolecular cross-relaxation of protons that are close to each other in space, confirming the presence of N–Zn linkage in solution.*

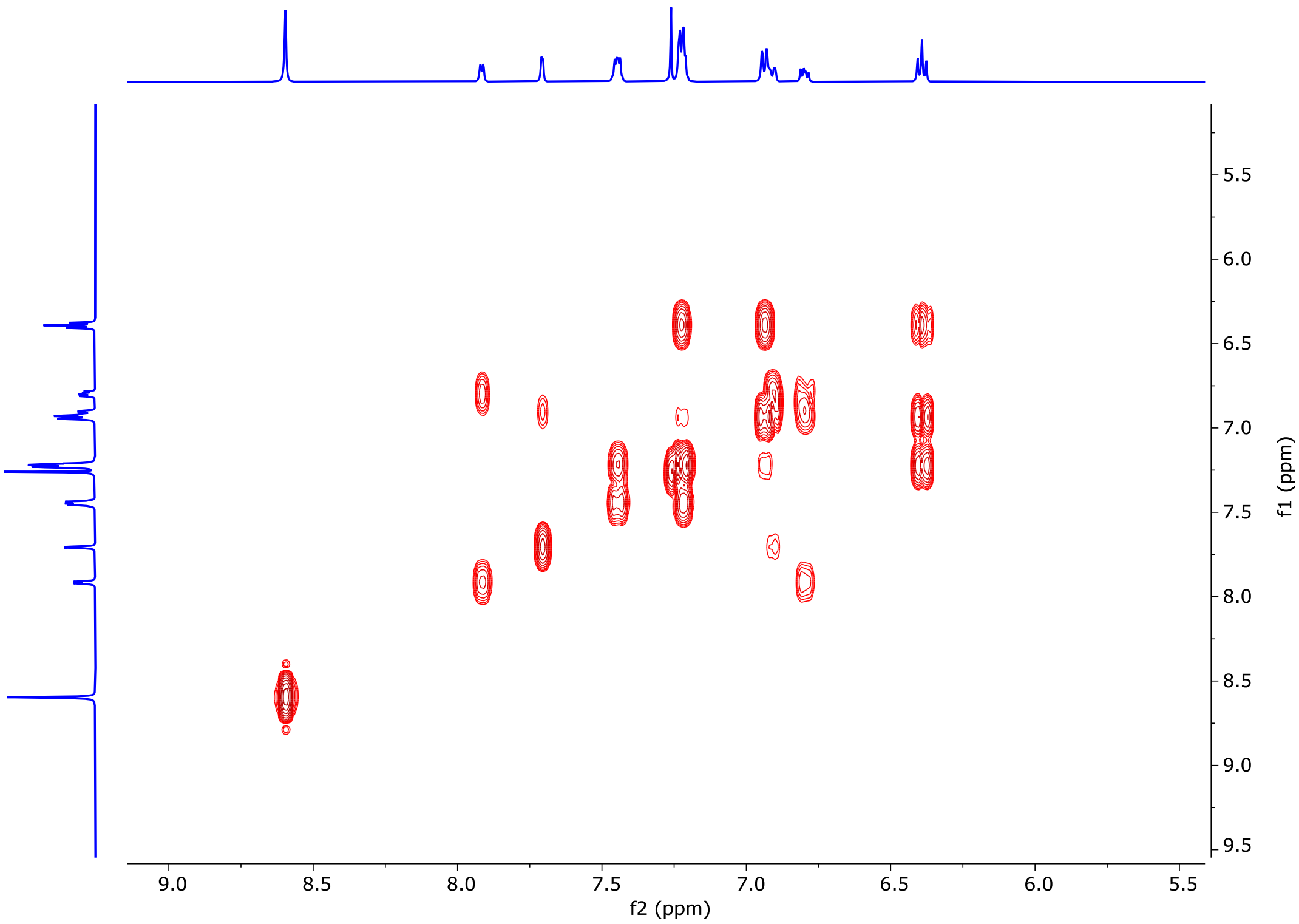


*Figure S19. $^1H$–$^1H$ gCOSY (298 K, $CDCl_3$, 500 MHz) spectrum of **3**·ZnSal-$^t$Bu.*

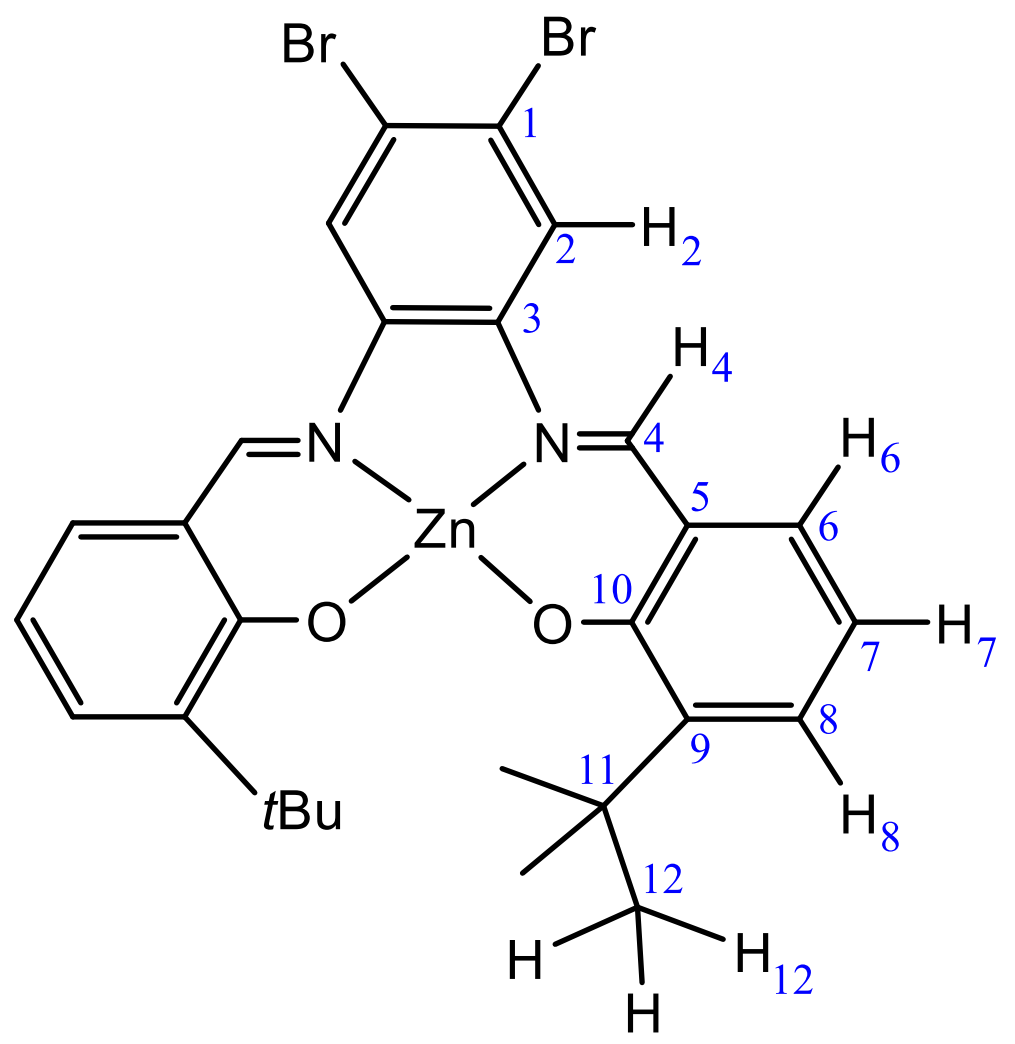


*Figure S20. Compound **ZnSal-$^t$Bu-Br** with the atom labelling used in the NMR studies.*

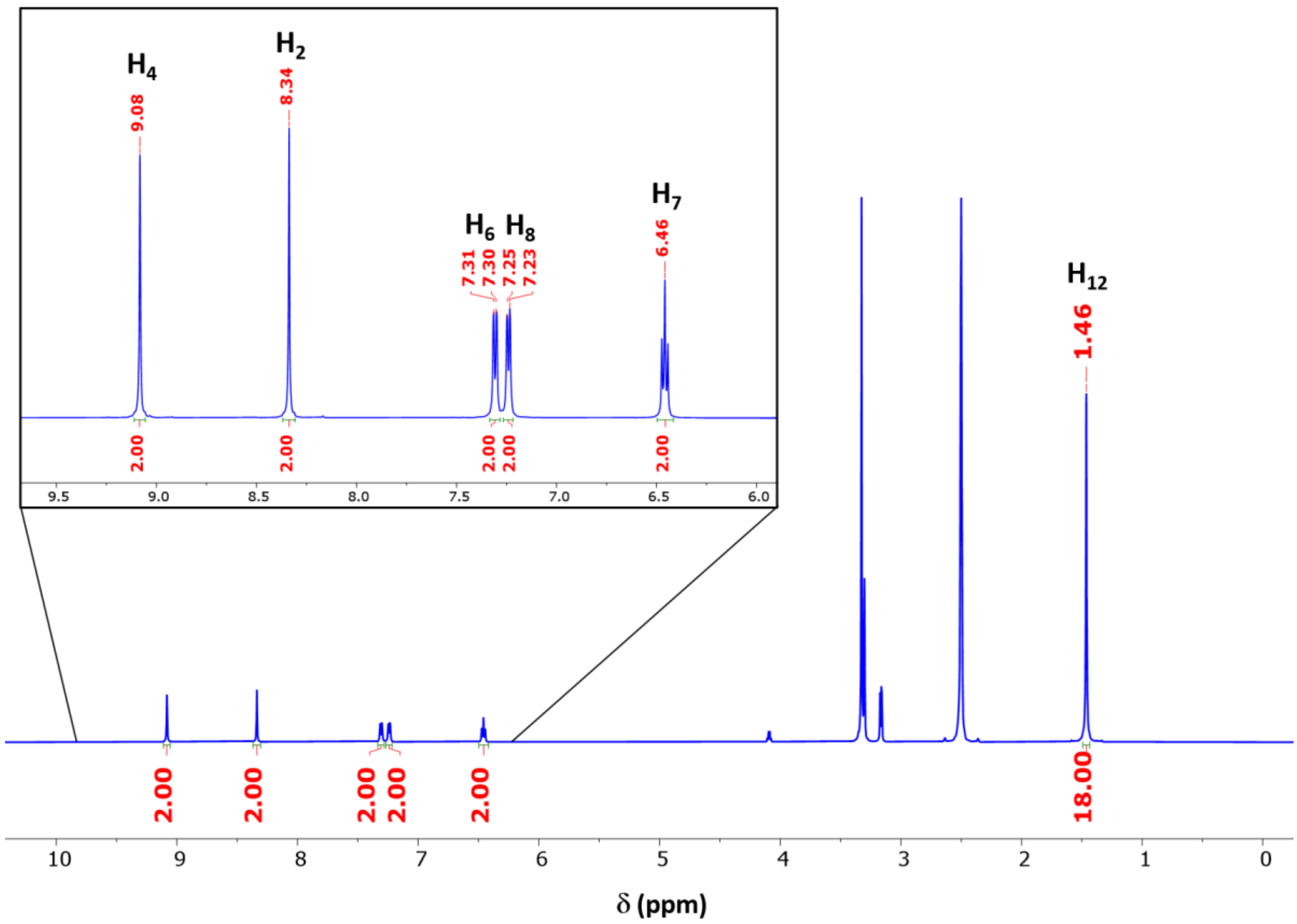


*Figure S21. $^1$H NMR (298 K, DMSO-d$_6$, 500 MHz) spectrum of **ZnSal-$^t$Bu-Br**.*

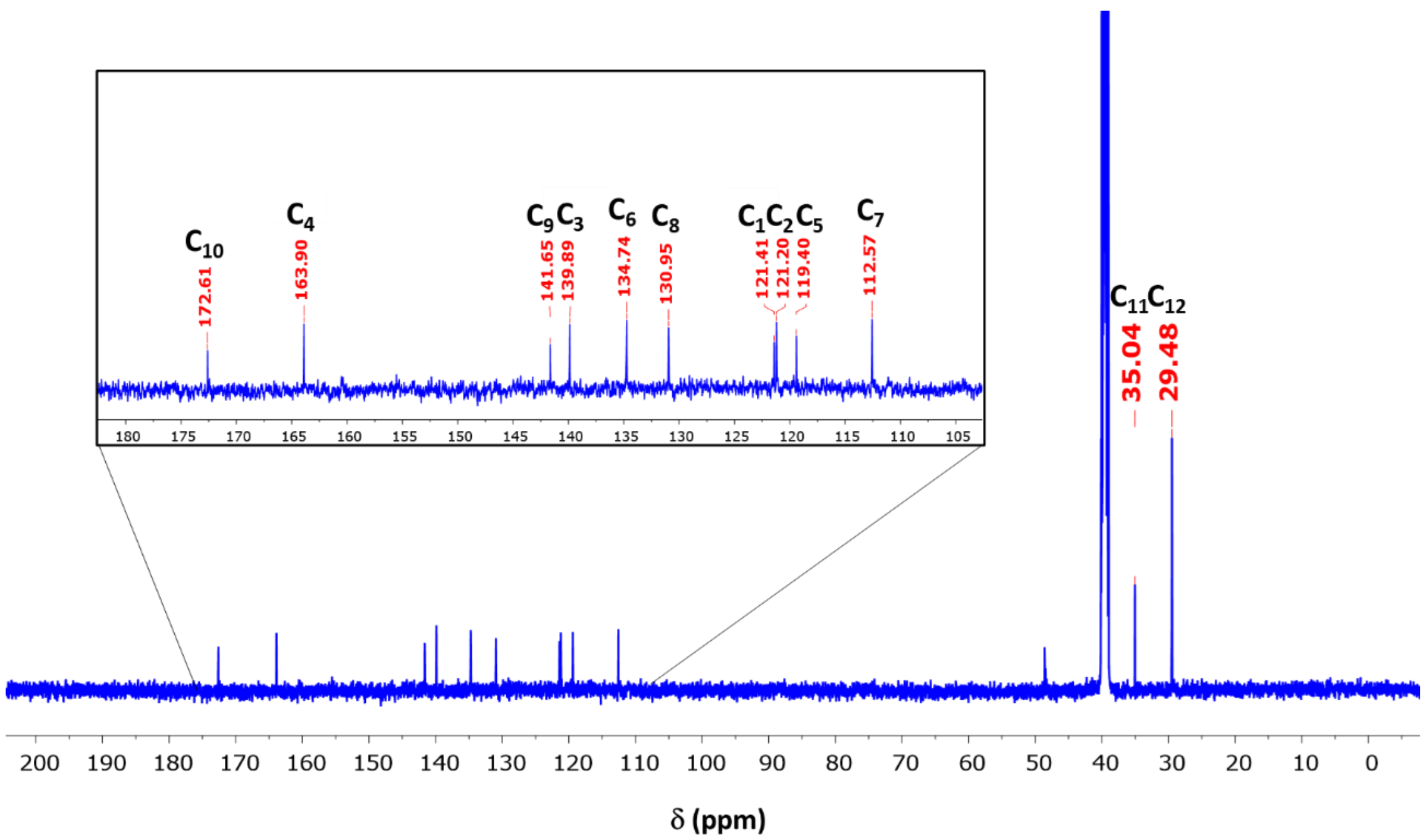


*Figure S22. $^{13}C\{^{1}H\}$-NMR (298 K, DMSO-$d_6$, 100.5 MHz) spectrum of **ZnSal-$^{t}$Bu-Br**.*

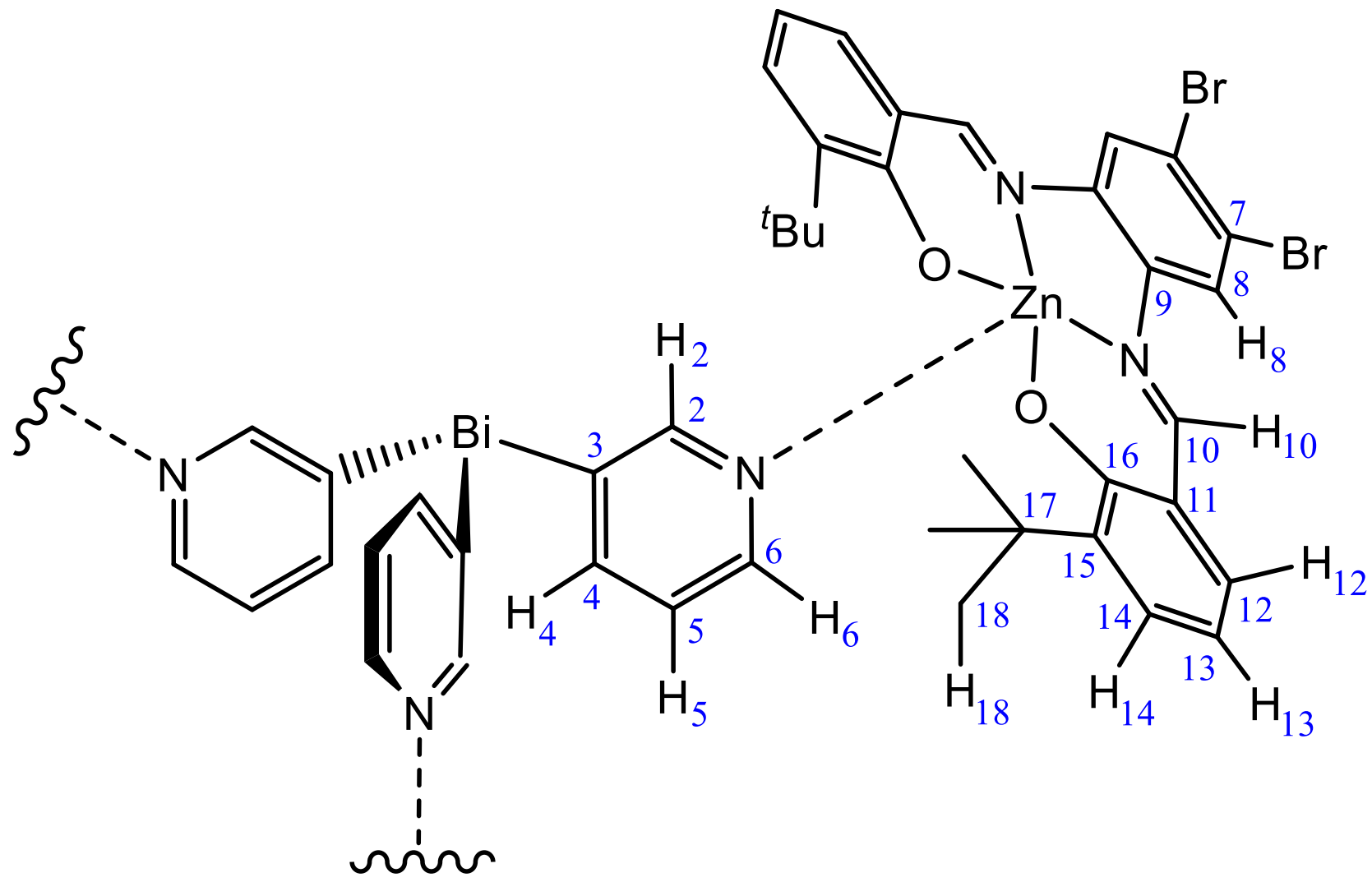


*Figure S23. Compound **1**·ZnSal-$^t$Bu-Br with the atom labelling used in the NMR studies.*

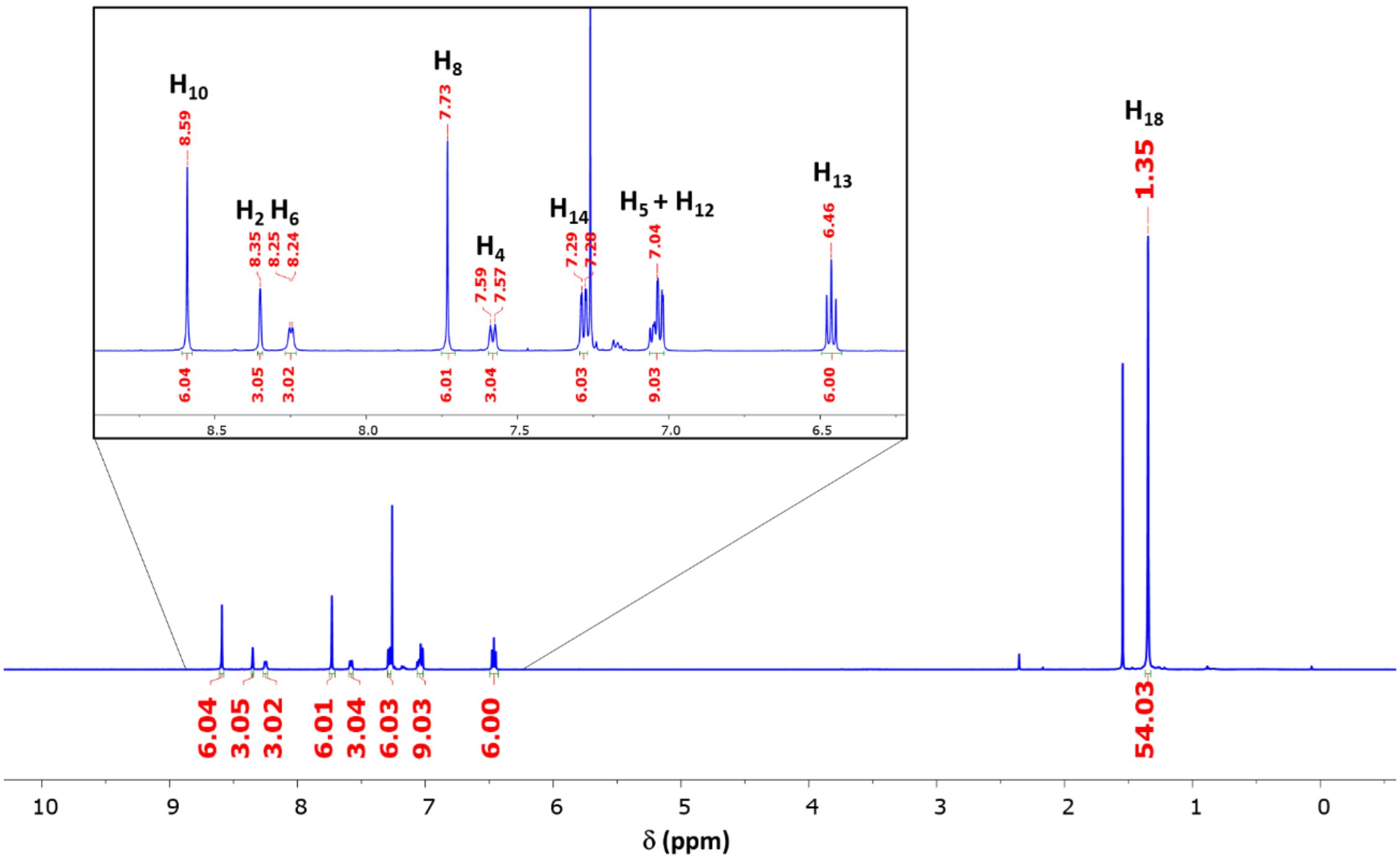


*Figure S24. $^1$H NMR (298 K, $CDCl_3$, 500 MHz) spectrum of **1**·ZnSal-$^t$Bu-Br.*

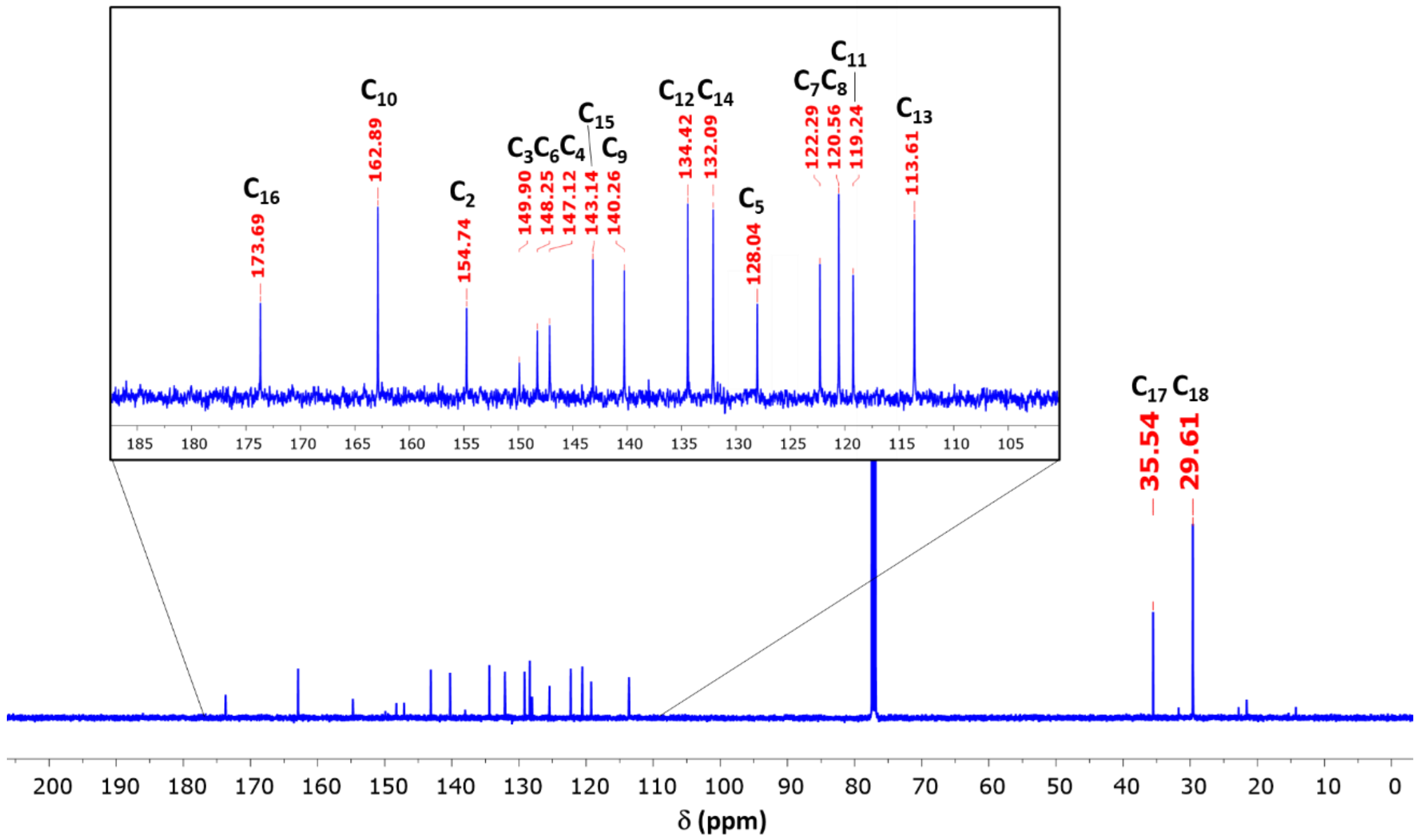


Figure S25. $^{13}C\{^{1}H\}$-NMR (298 K, $CDCl_3$, 100.5 MHz) spectrum of ***1*·ZnSal-$^{t}$Bu-Br.**

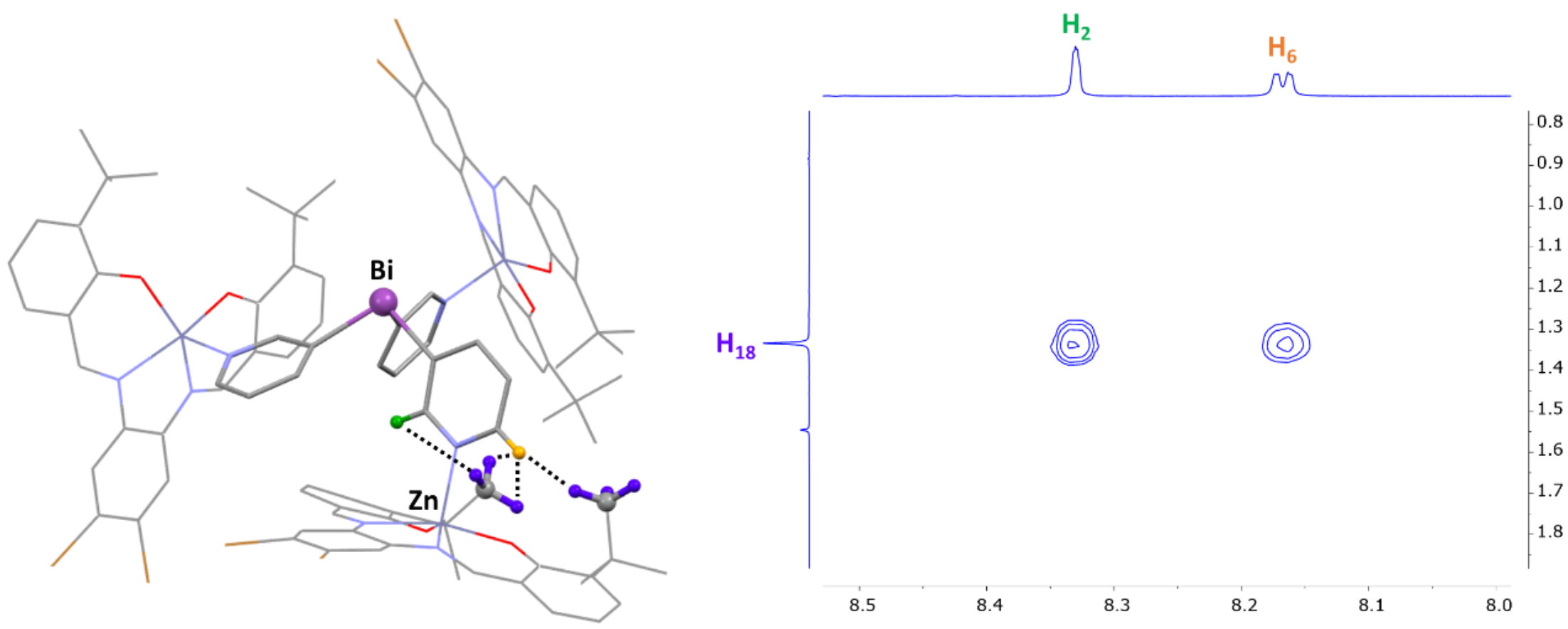


*Figure S26. Selected region of the $^{1}H$–$^{1}H$ NOESY (298 K, $CDCl_3$, mixing time of 800 ms) spectrum of **1**·ZnSal-$^{t}$Bu-Br. Crosspeaks observed between $H_{18}$ and pyridinic $H_2$, $H_6$ protons arise from intramolecular cross-relaxation of protons that are close to each other in space, confirming the presence of N–Zn linkage in solution.*

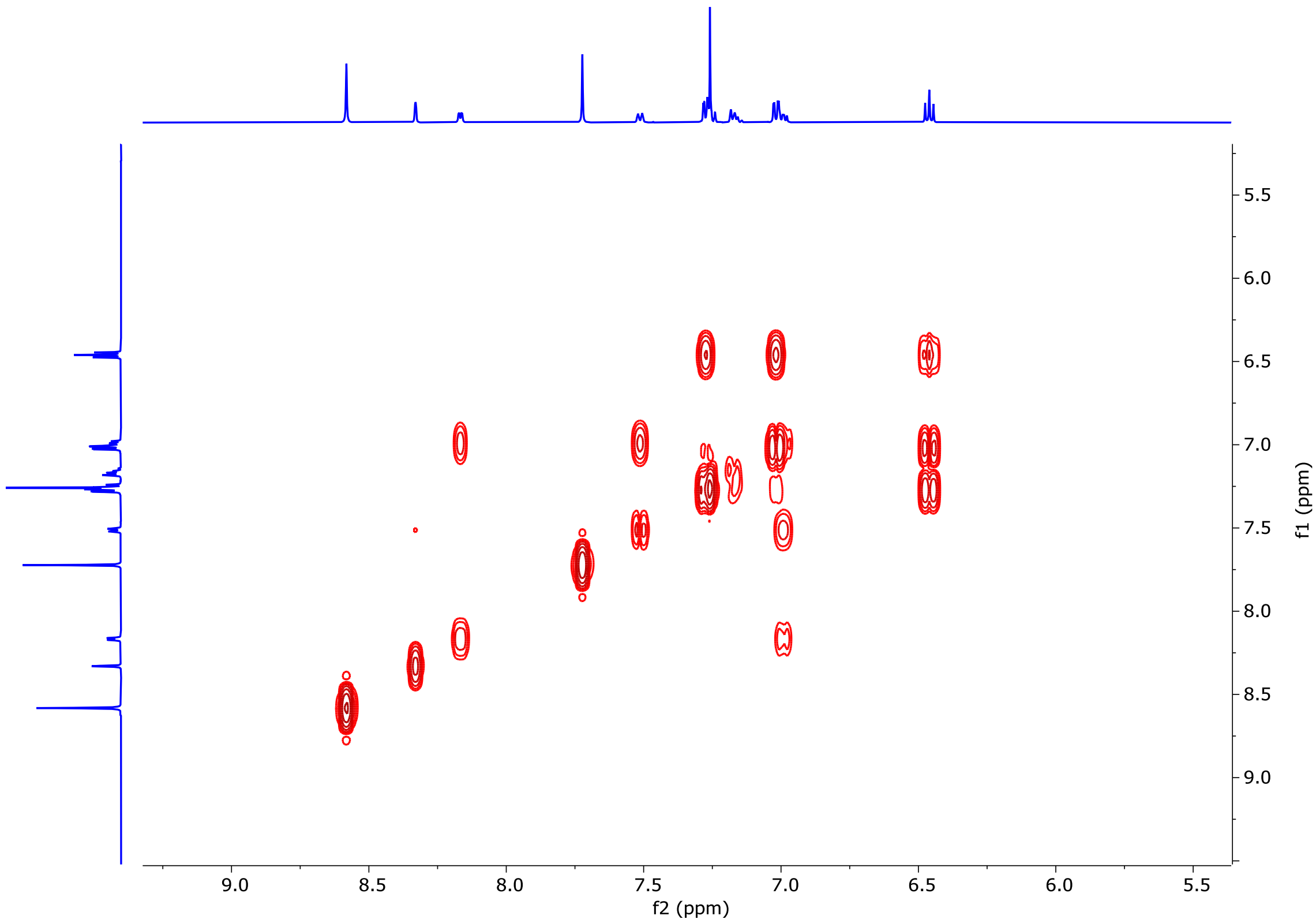


*Figure S27. $^1$H–$^1$H gCOSY (298 K, $CDCl_3$, 500 MHz) spectrum of **1**·ZnSal-$^t$Bu-Br.*

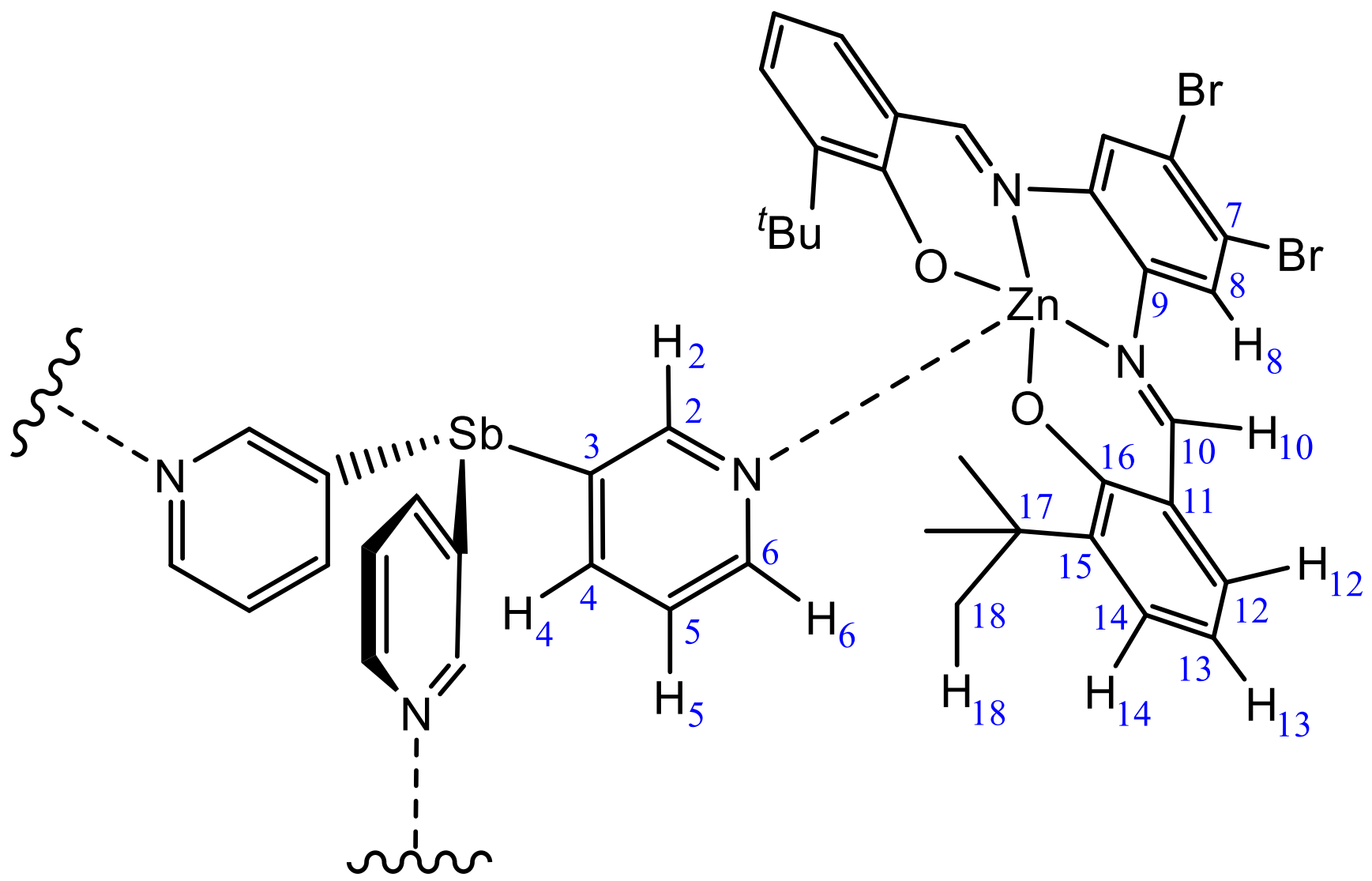


*Figure S28. Compound **2**·ZnSal-$^t$Bu-Br with the atom labelling used in the NMR studies.*

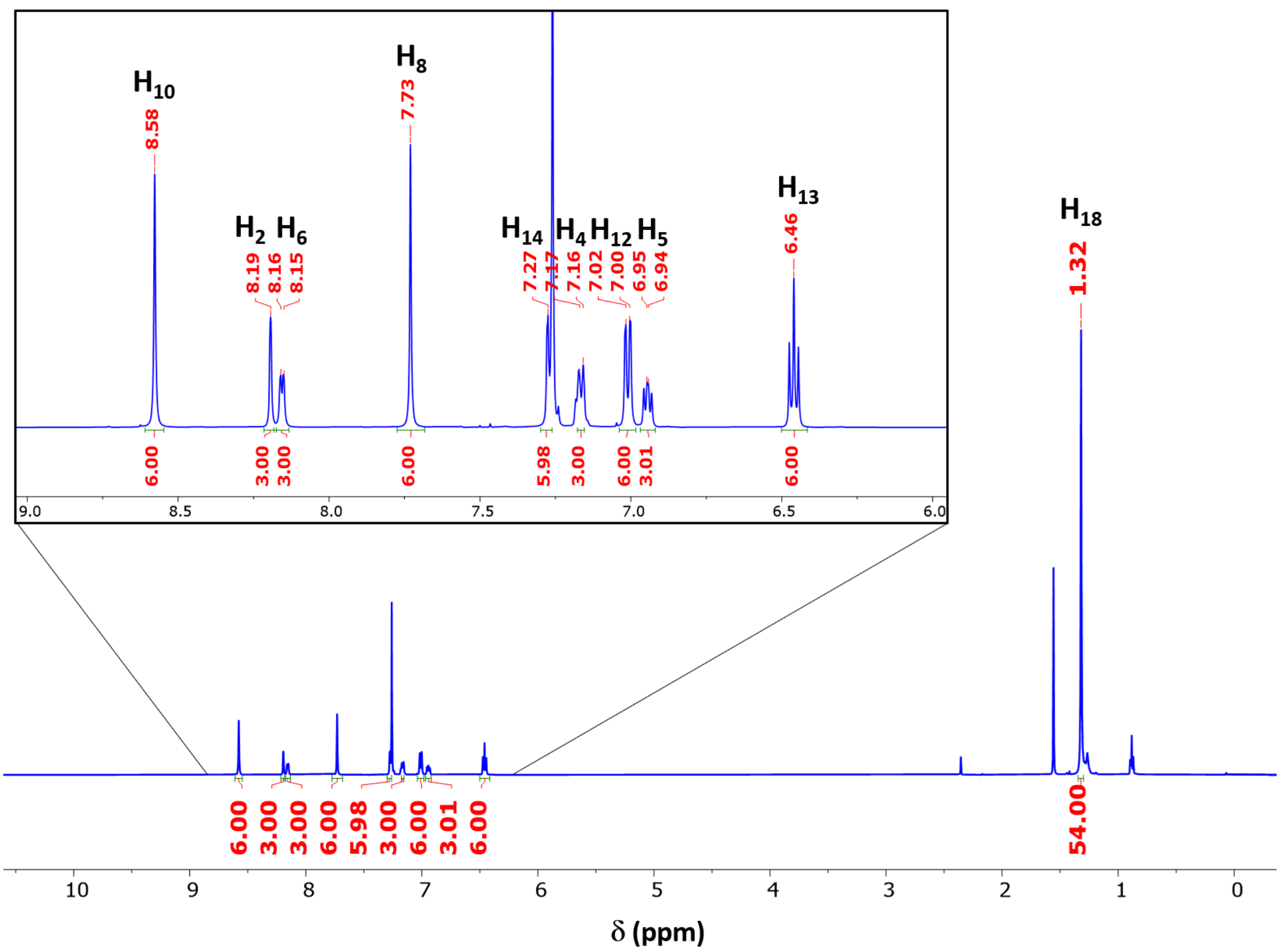


*Figure S29. $^1$H NMR (298 K, $CDCl_3$, 500 MHz) spectrum of **2**·ZnSal-$^t$Bu-Br.*

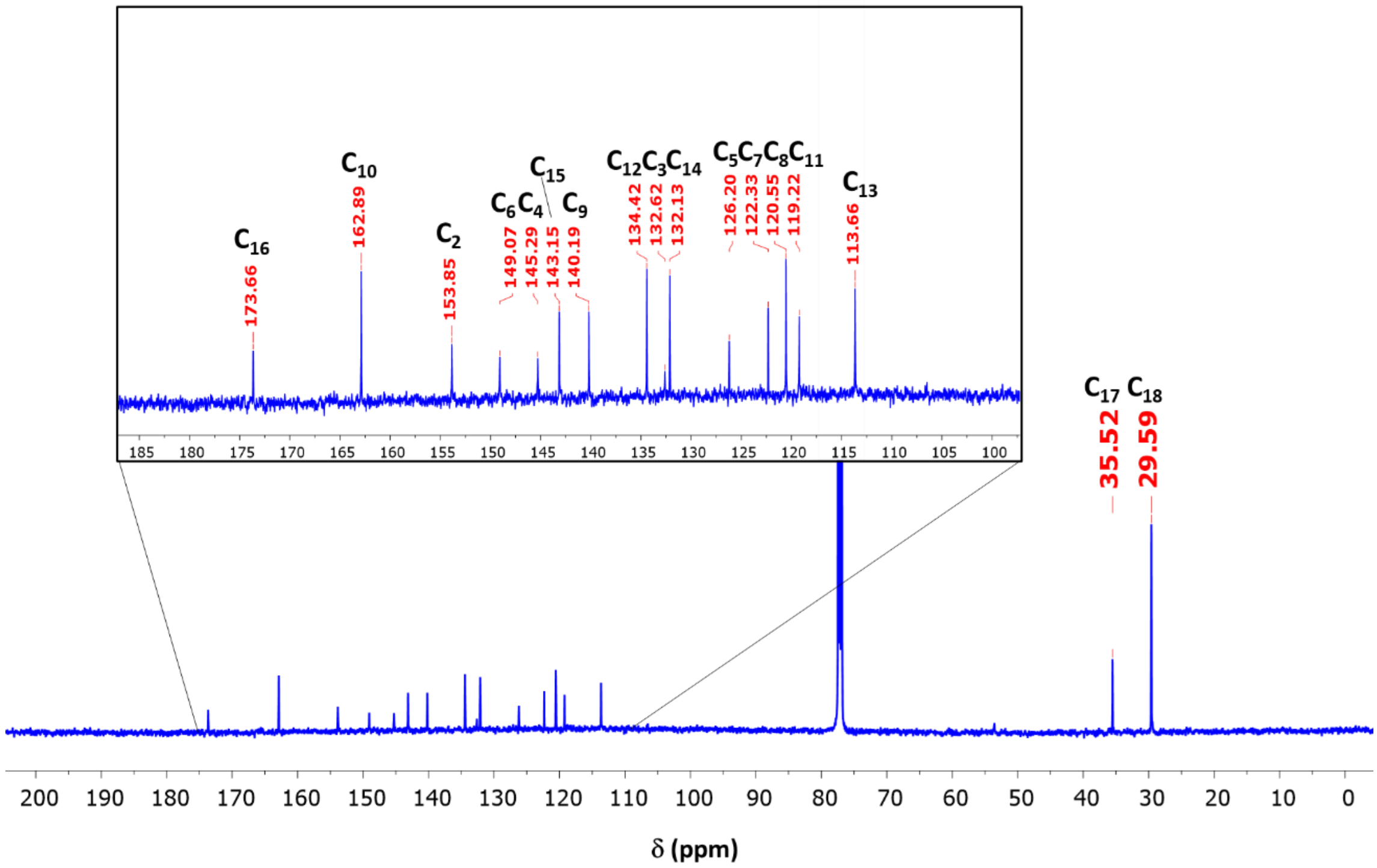


*Figure S30. $^{13}C\{^1H\}$-NMR (298 K, $CDCl_3$, 100.5 MHz) spectrum of **2**·ZnSal-$^t$Bu-Br.*

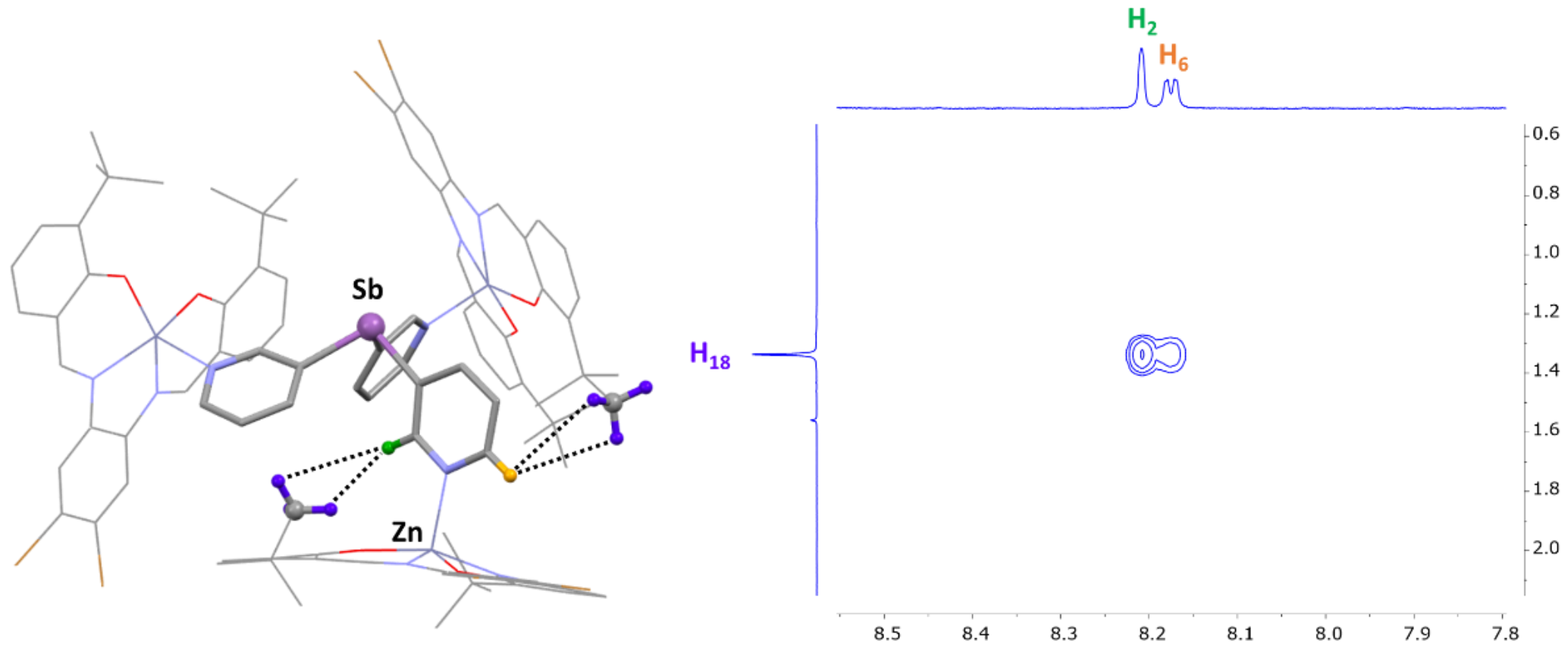


*Figure S31. Selected region of the $^1H$–$^1H$ ROESY (298 K, $CDCl_3$, mixing time of 400 ms) spectrum of **2**·ZnSal-$^t$Bu-Br. Crosspeaks observed between $H_{18}$ and pyridinic $H_2$, $H_6$ protons arise from intramolecular cross-relaxation of protons that are close to each other in space, confirming the presence of N–Zn linkage in solution.*

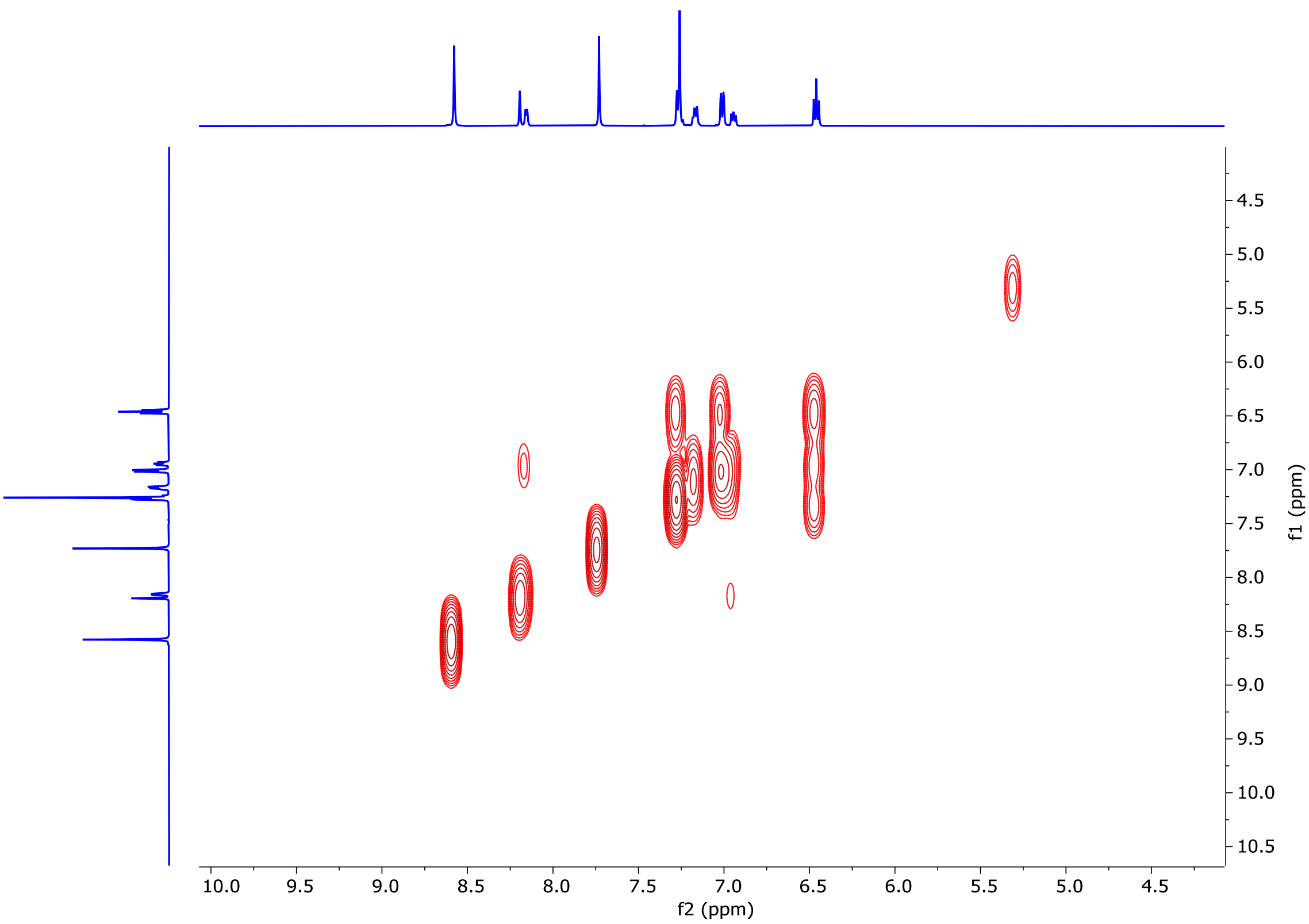


*Figure S32. $^{1}H$–$^{1}H$ gCOSY (298 K, $CDCl_3$, 500 MHz) spectrum of **2**·ZnSal-$^{t}$Bu-Br.*

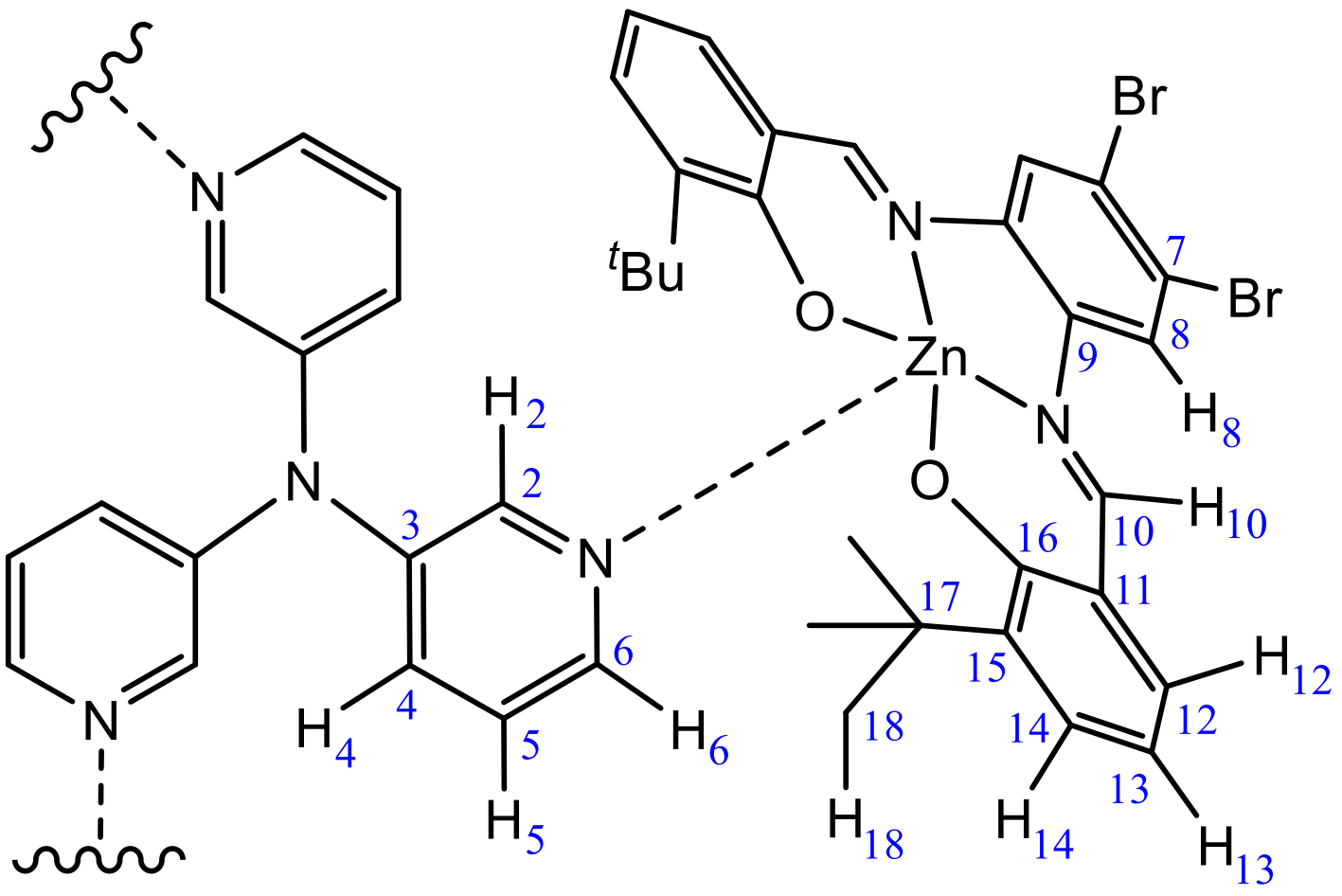


*Figure S33. Compound **3**·ZnSal-tBu-Br with the atom labelling used in the NMR studies.*

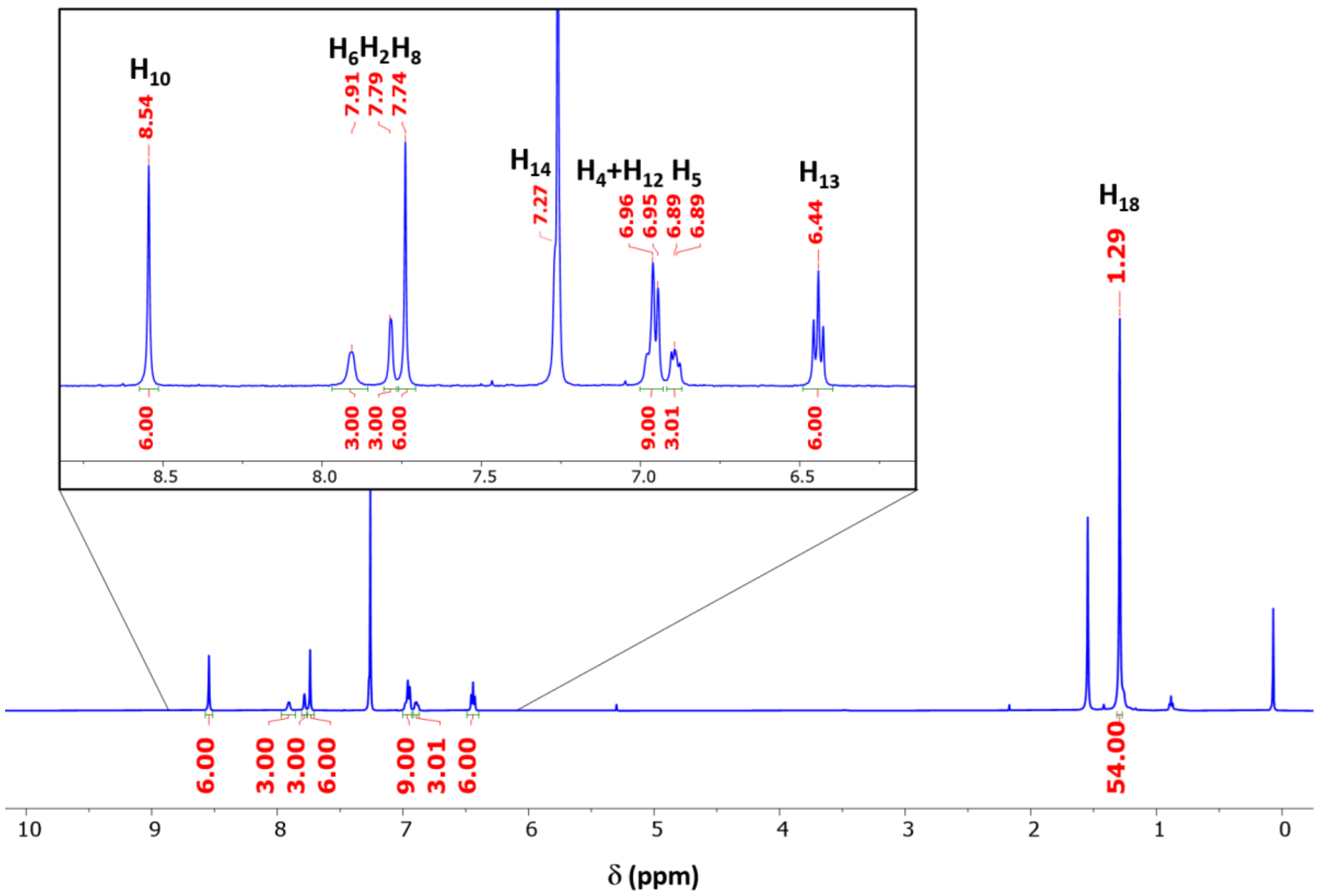


*Figure S34. $^1$H NMR (298 K, $CDCl_3$, 500 MHz) spectrum of **3**·ZnSal-tBu-Br.*

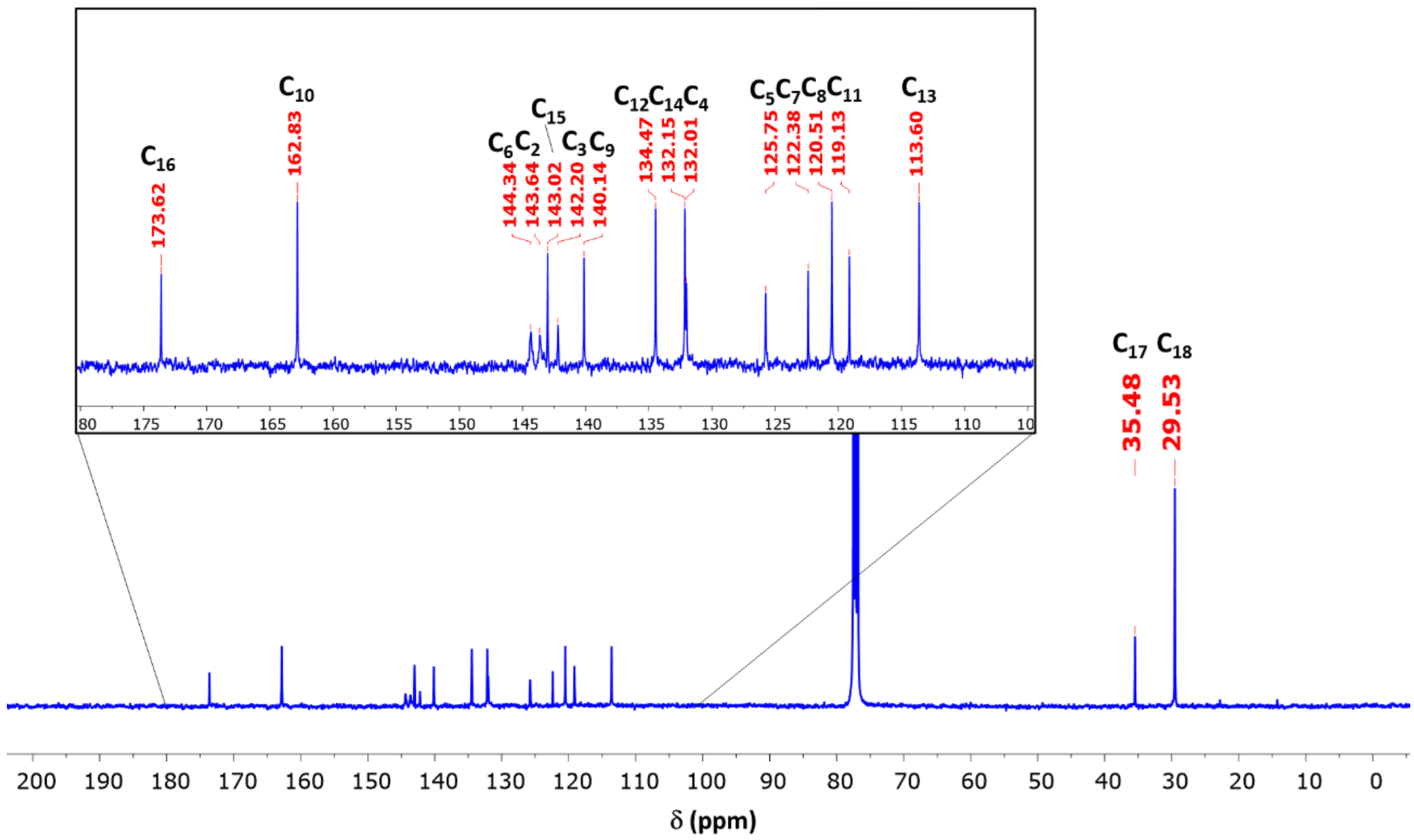


*Figure S35. $^{13}C\{^{1}H\}$-NMR (298 K, $CDCl_3$, 100.5 MHz) spectrum of **3**·ZnSal-$^{t}$Bu-Br.*

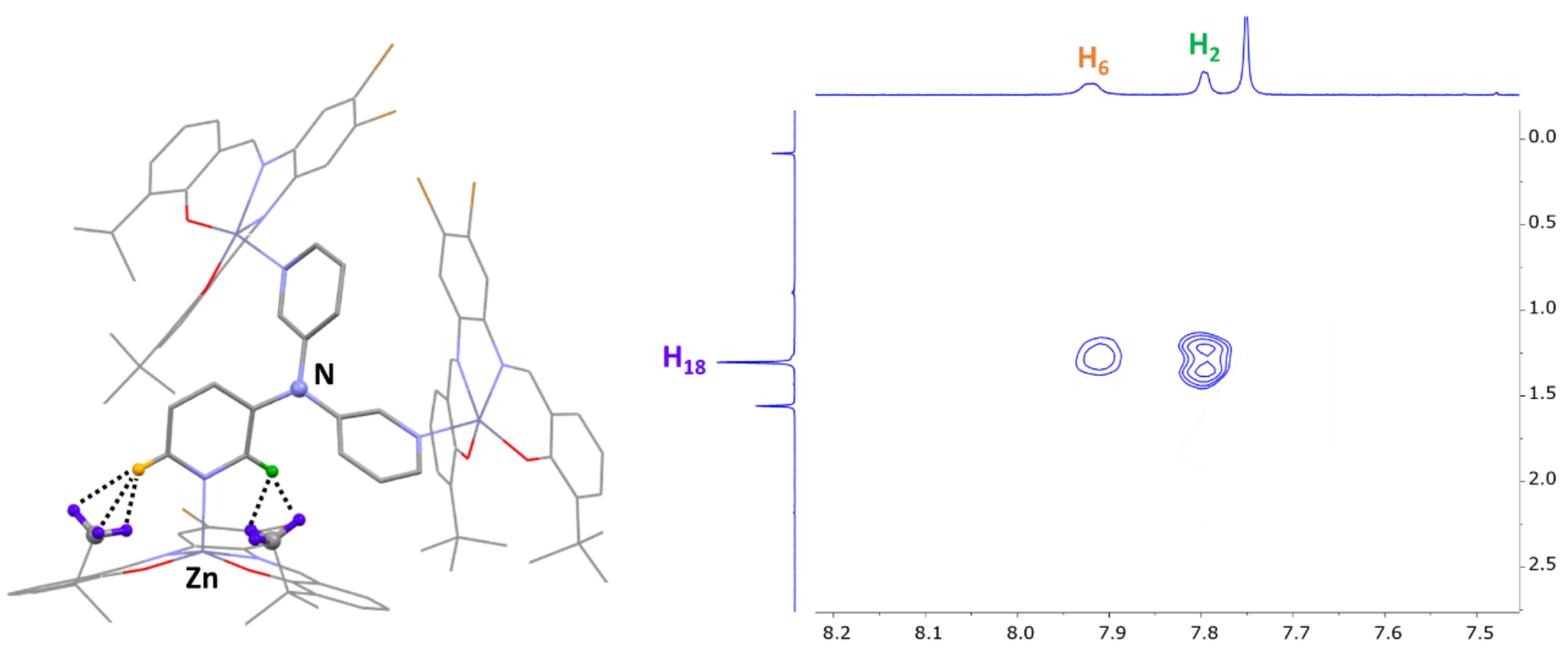


*Figure S36. Selected region of the $^{1}H$–$^{1}H$ NOESY (298 K, $CDCl_3$, mixing time of 800 ms) spectrum of **3**·ZnSal-$^{t}$Bu-Br. Crosspeaks observed between $H_{18}$ and pyridinic $H_2$, $H_6$ protons arise from intramolecular cross-relaxation of protons that are close to each other in space, confirming the presence of N–Zn linkage in solution.*

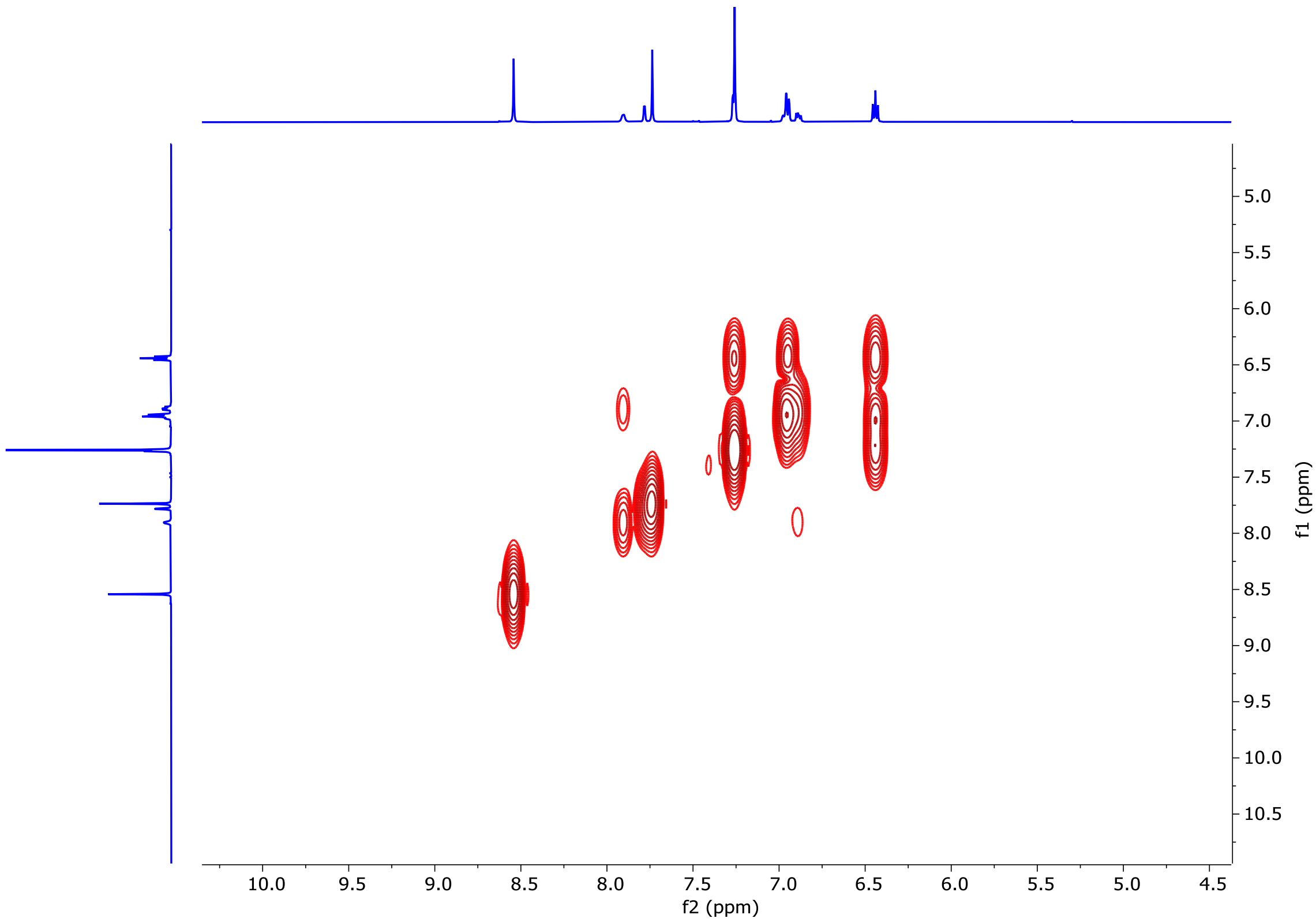


*Figure S37.* $^{1}$*H–*$^{1}$*H gCOSY (298 K,* $CDCl_3$*, 500 MHz) spectrum of* ***3***·*ZnSal-*$^{t}$*Bu-Br.*

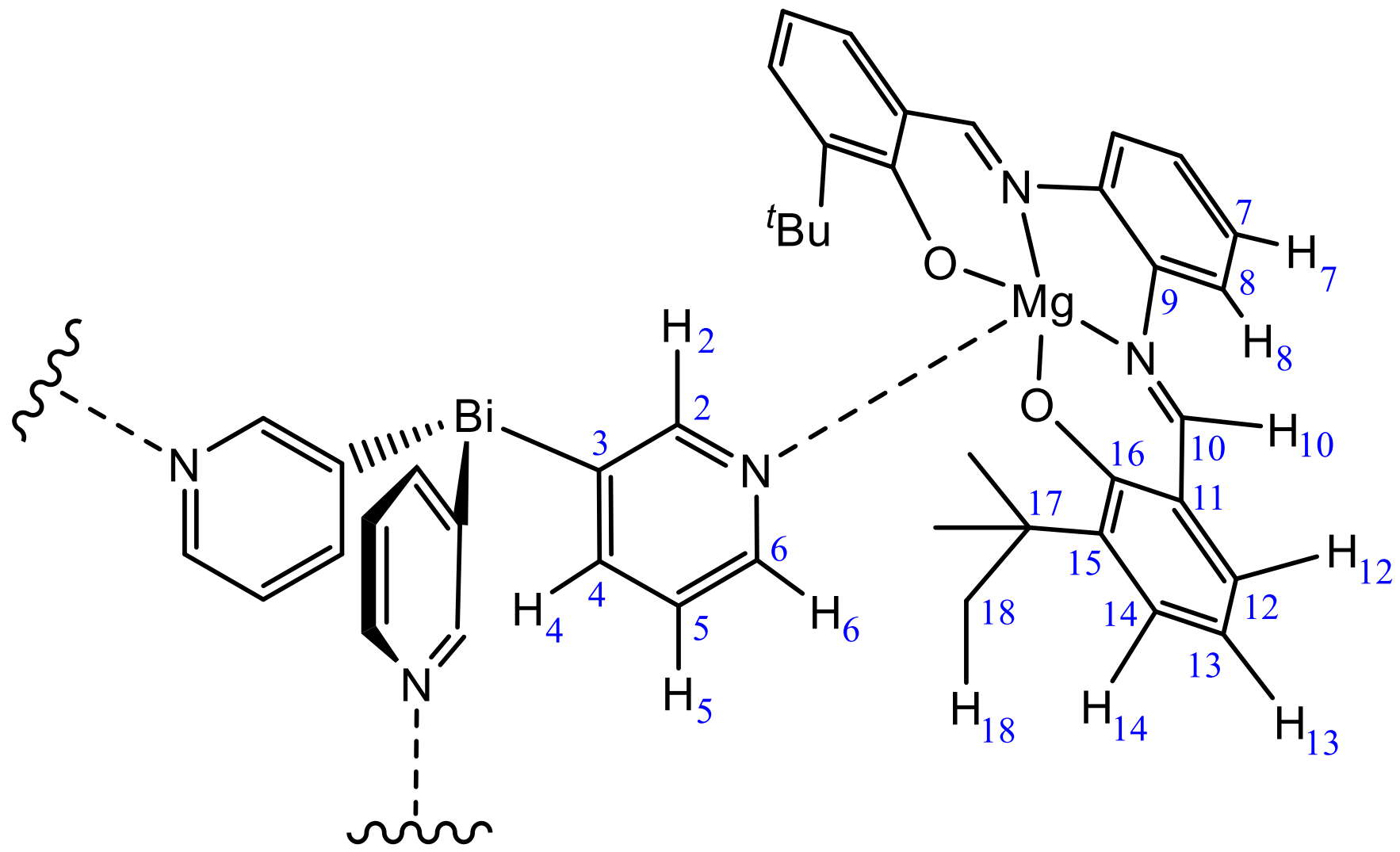


*Figure S38. Compound **1**·MgSal-$^t$Bu with the atom labelling used in the NMR studies.*

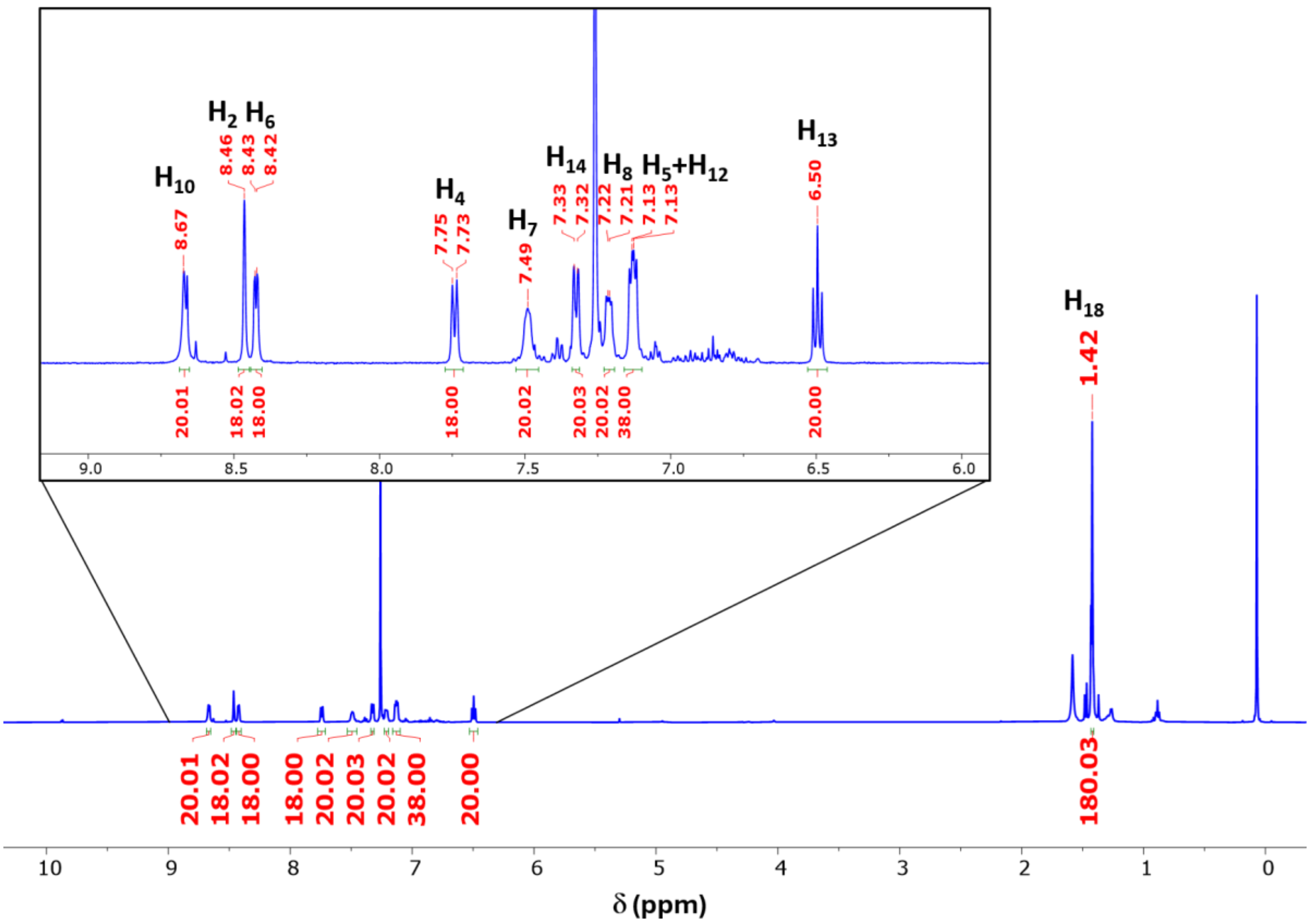


*Figure S39. $^1$H NMR (298 K, CDCl$_3$, 500 MHz) spectrum of **1**·MgSal-$^t$Bu.*

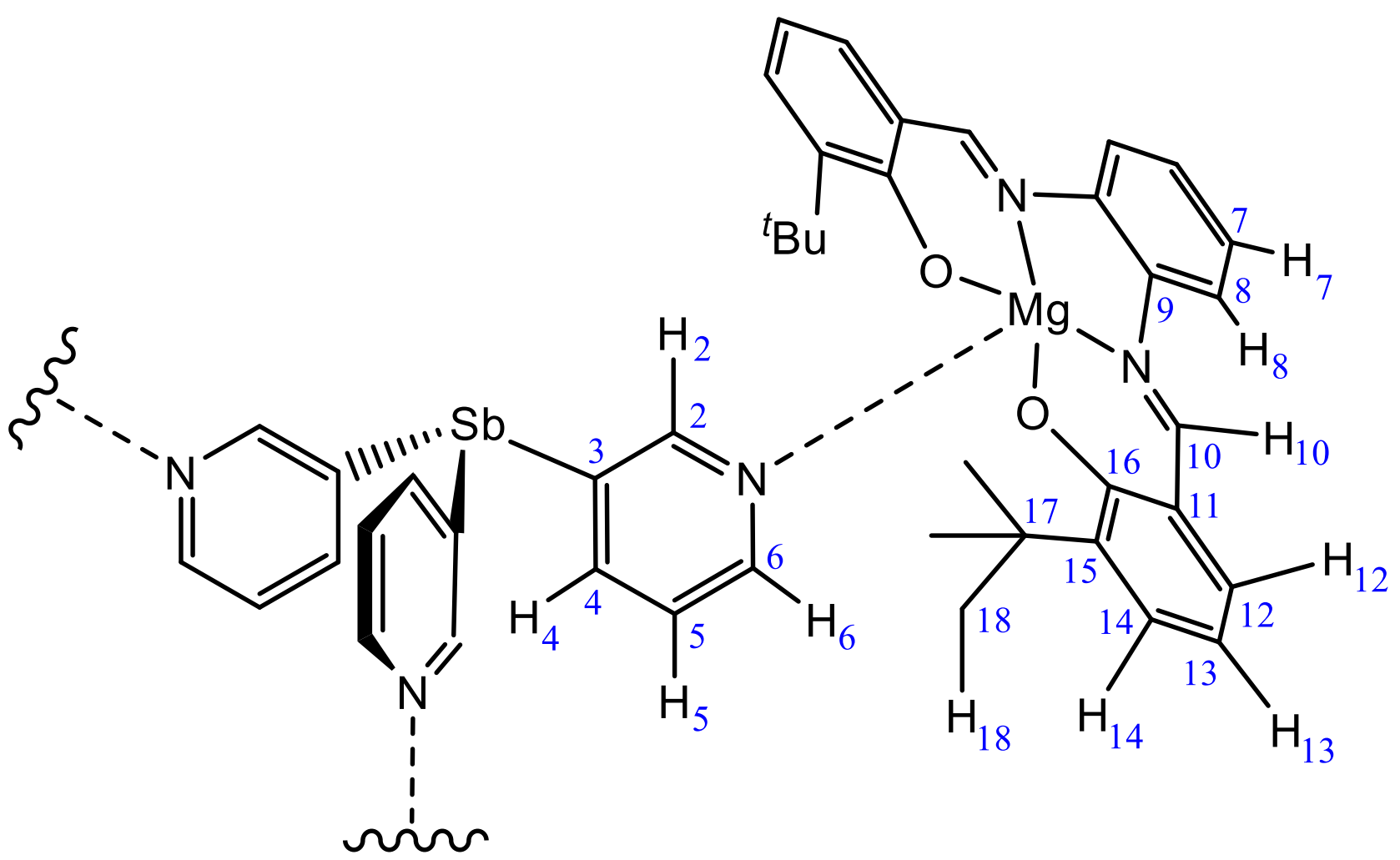


*Figure S40. Compound **2**·MgSal-$^{t}$Bu with the atom labelling used in the NMR studies.*

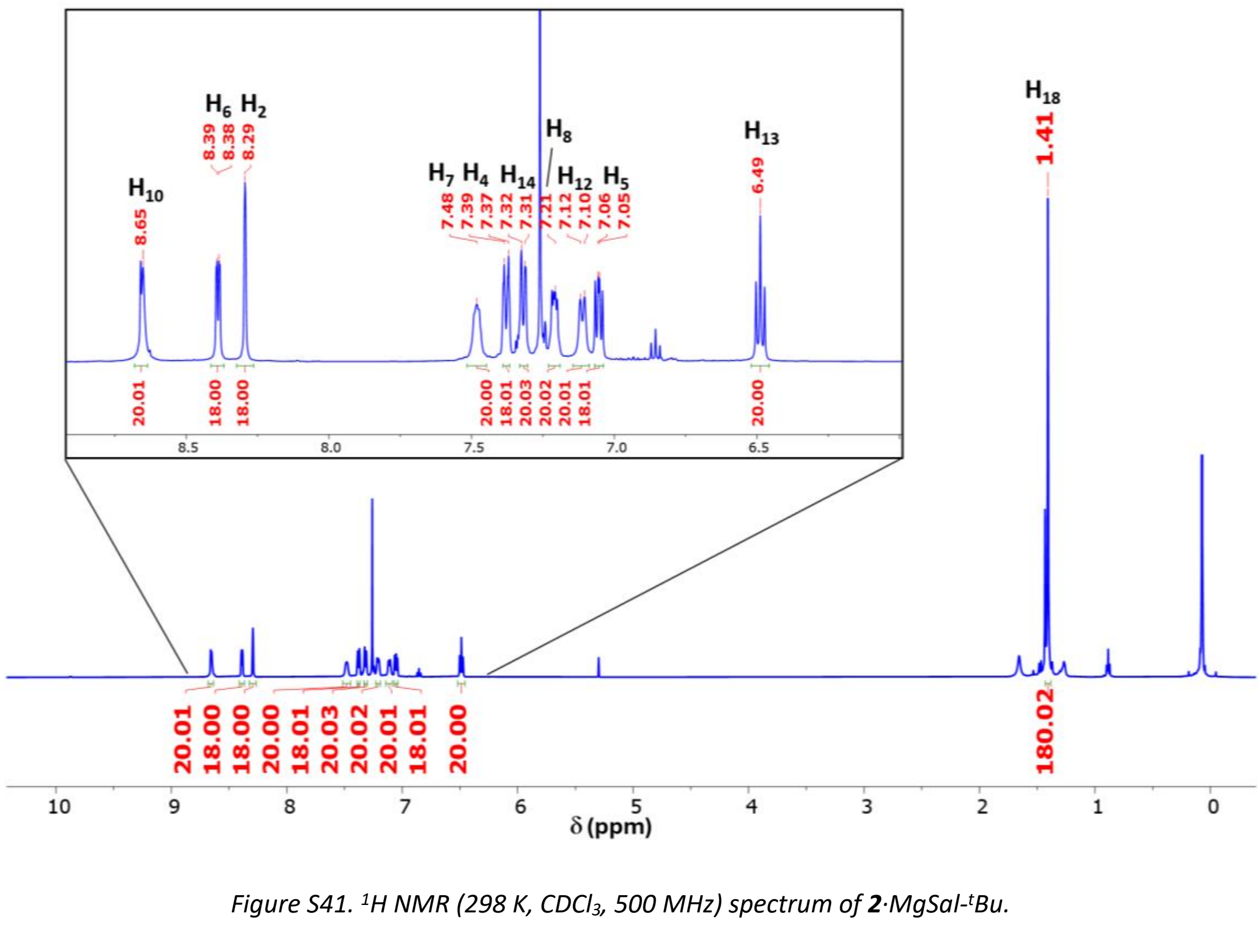


*Figure S41. $^{1}$H NMR (298 K, $CDCl_3$, 500 MHz) spectrum of **2**·MgSal-$^{t}$Bu.*

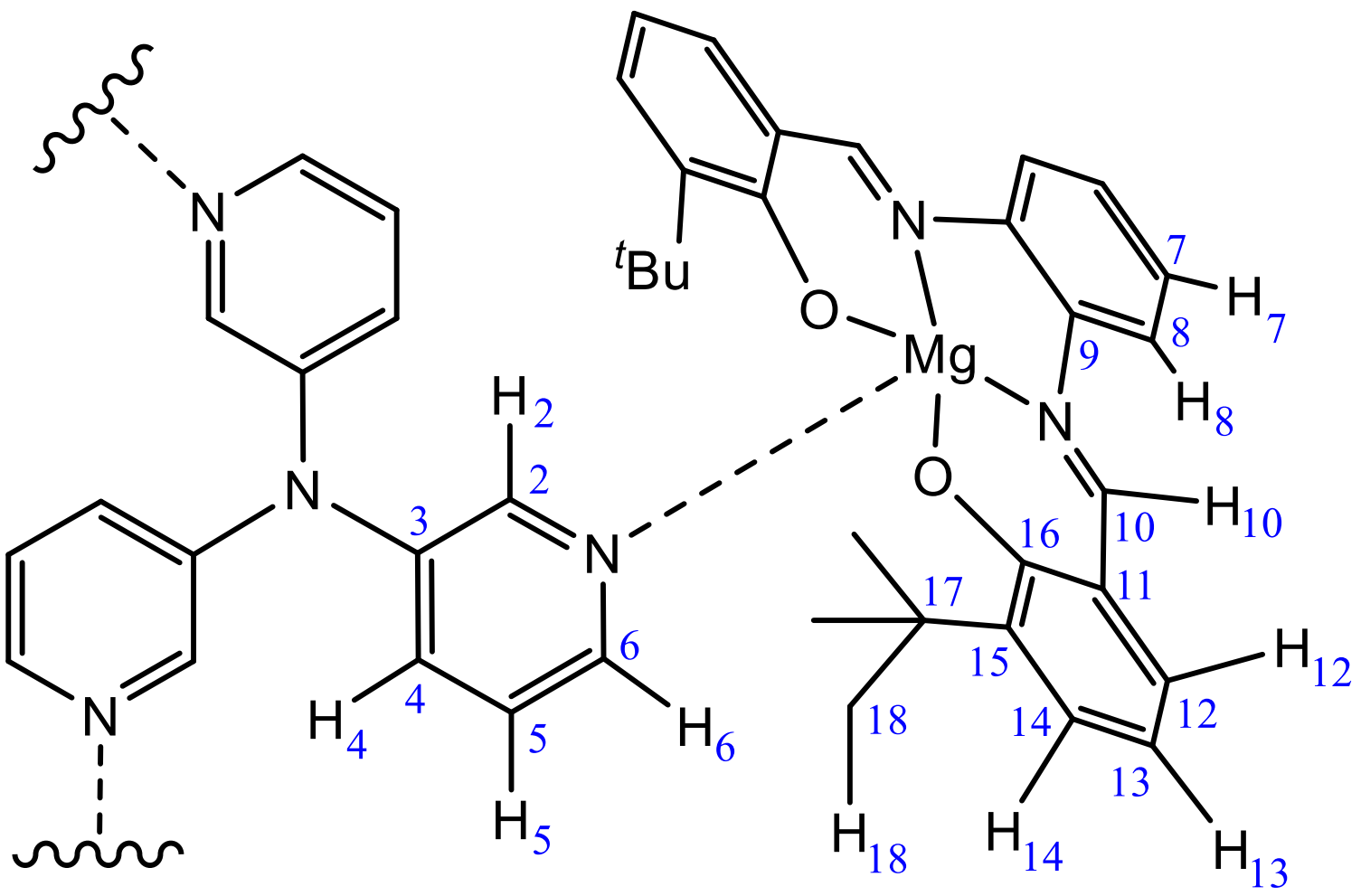

*Figure S42. Compound **3**·MgSal-ᵗBu with the atom labelling used in the NMR studies.*

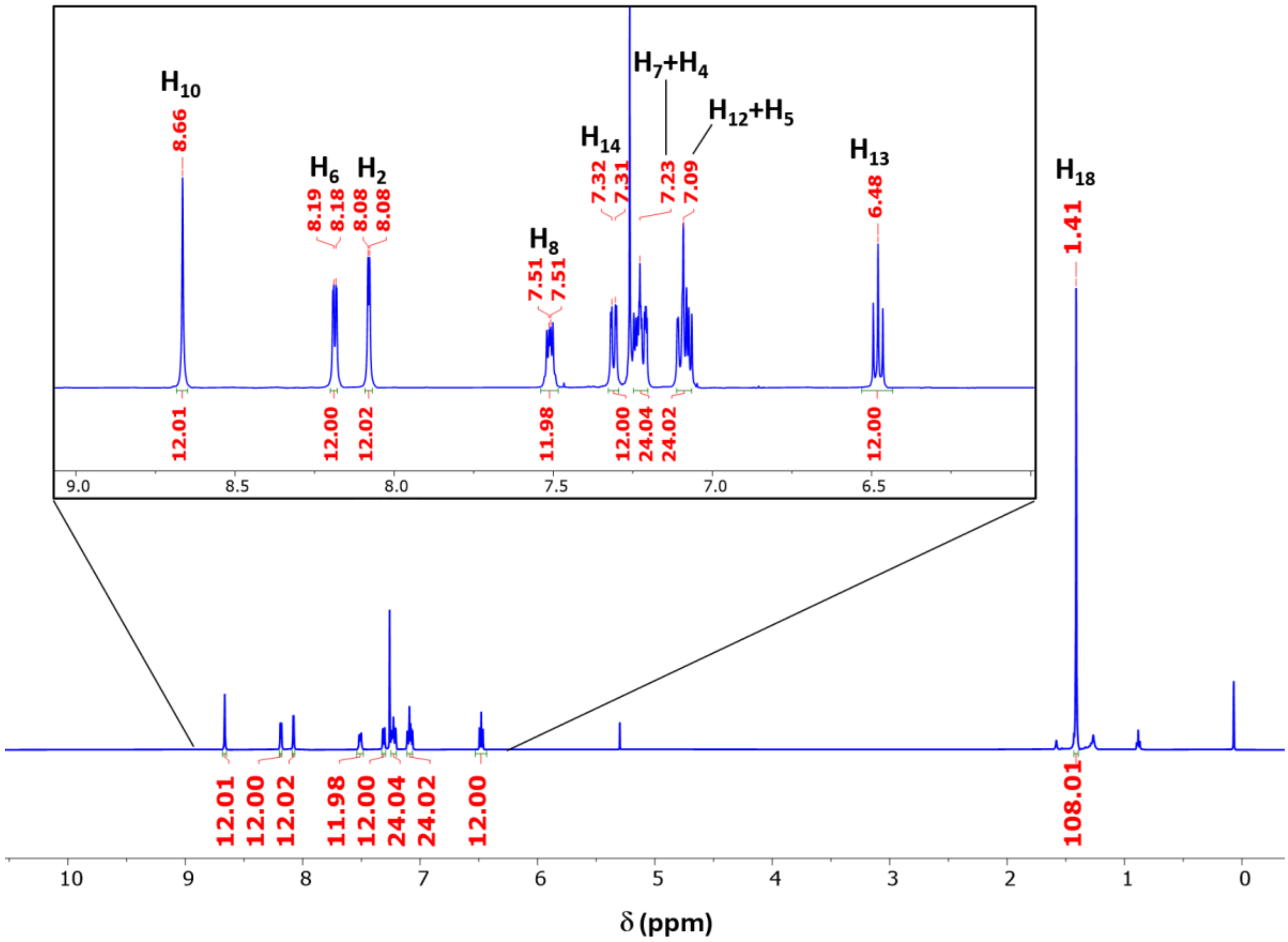


*Figure S43. $^{1}H$ NMR (298 K, $CDCl_3$, 500 MHz) spectrum of compound **3**·MgSal-ᵗBu.*

## DOSY experiments

$^{1}$H-DOSY experiments were performed to estimate the hydrodynamic radii of the molecular capsules in $CDCl_3$ solution.

For the capsules **1**·ZnSal-$^{t}$Bu, **2**·ZnSal-$^{t}$Bu and **3**·ZnSal-$^{t}$Bu, the DOSY experiments indicate that the structures remain intact in solution as all signals corresponding to the salphen and the ligand fragments present similar diffusion coefficients. Additionally, the calculated hydrodynamic radii derived from the diffusion coefficient values ($r_{\text{H-DOSY}}$) are comparable with those expected based on the crystallographic radii ($r_{\text{X-Ray}}$) (Figures S44 and S45).

In contrast, for the macrocycles **1**·MgSal-$^{t}$Bu, **2**·MgSal-$^{t}$Bu and **3**·MgSal-$^{t}$Bu the signals of the ligand and the salphen present different diffusion coefficients, indicating that the structure observed in solid state does not remain intact in solution. In order to estimate their aggregation state in solution, the DOSY NMR data were analysed using the Molecular Weight (MW) Estimation Software developed by Stalke ($ECC_{DSE}^{CDCl_3}$).[1,2,3] $^{1}$H-DOSY-ECC-MW estimation of **1**·MgSal-$^{t}$Bu, **2**·MgSal-$^{t}$Bu and **3**·MgSal-$^{t}$Bu in $CDCl_3$ was carried out at 298 K. Adamantane was used as an internal reference. The molecular weights estimated from the diffusion coefficients using the Stalke method ($MW_{det}$) suggest the formation of (on average) monomeric species of the type {[E(3-py)$_3$]·(MgSal-$^{t}$Bu)} (see below).

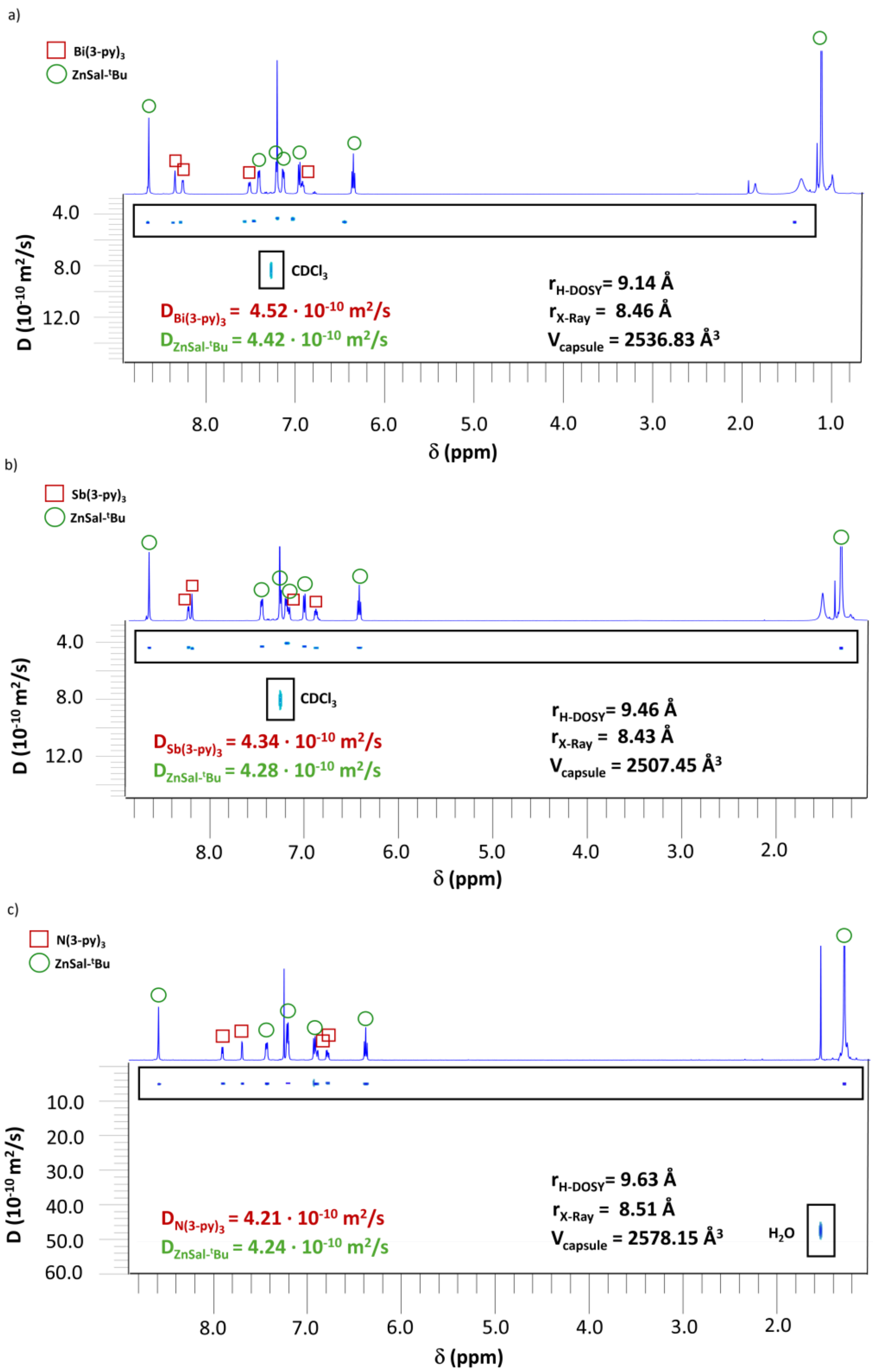


*Figure S44. $^{1}$H DOSY NMR spectra of (a) **1**·ZnSal-$^t$Bu, (b) **2**·ZnSal-$^t$Bu and (c) **3**·ZnSal-$^t$Bu in $CDCl_3$ at 298 K. In all three cases, the signals corresponding to the salphen and ligand fragments exhibit similar diffusion coefficients, indicating that the capsule arrangement remains intact in solution.*

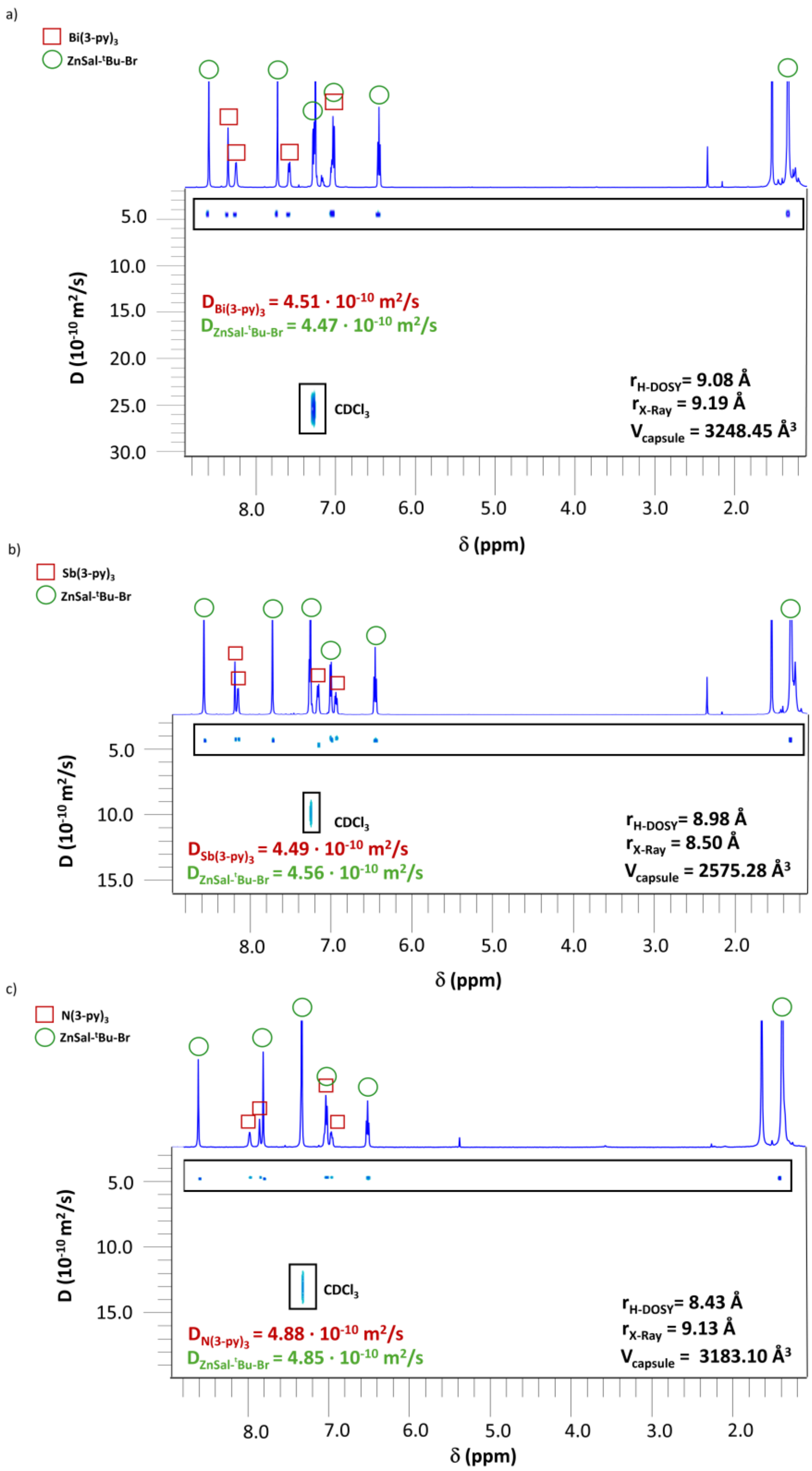


*Figure S45. $^{1}H$ DOSY NMR spectra of (a) **1** ZnSal-$^{t}$Bu-Br, (b) **2** ZnSal-$^{t}$Bu-Br and (c) **3** ZnSal-$^{t}$Bu-Br in $CDCl_3$ at 298 K. In all three cases, the signals corresponding to the salphen and ligand fragments exhibit similar diffusion coefficients, indicating that the capsule arrangement remains intact in solution.*

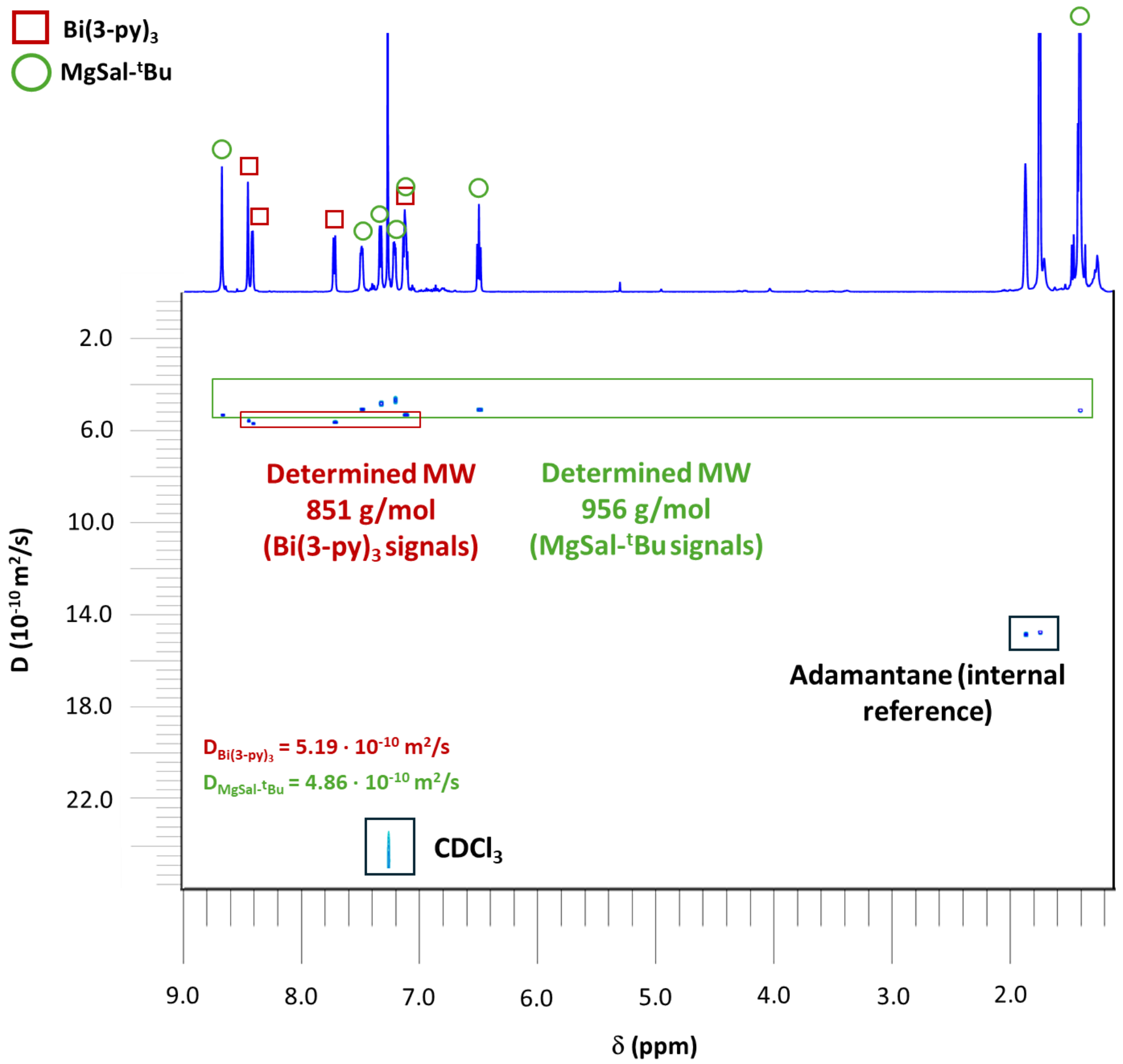


*Figure S46. $^{1}$H DOSY NMR spectrum of* ***1****·MgSal-[t]Bu and adamantane (internal reference) at 298 K in $CDCl_3$. The diffusion coefficients (D) corresponding to the $Bi(3\text{-}py)_3$ and MgSal-[t]Bu signals are highlighted in red and green, respectively.*

| Compound | *D* ($m^2/s$) | log *D* | log $D_{x,norm}$ | MW (g/mol) |
|---|---|---|---|---|
| **Adamantane** | 1.465E-09 | -8.8343 | - | 136.23[a] |
| **MgSal-[t]Bu** | 4.859E-10 | -9.3135 | -9.2947 | 956[b] |
| **$Bi(3\text{-}py)_3$** | 5.192E-10 | -9.2847 | -9.2659 | 851[b] |

*Table S1. Molecular weight (MW) analysis using the $^{1}$H DOSY NMR data obtained for the mixture of* ***1****·MgSal-[t]Bu and adamantane (internal reference) at 298 K in $CDCl_3$. [a]Real MW. [b]$MW_{det}$.*

| Aggregate | $MW_{det}$ (MgSal-$^{t}$Bu signals) | $MW_{det}$ (Bi(3-py)$_3$ signals) | $MW_{cal}$ (theoretical) | $MW_{Diff}$ (%) |
|---|---|---|---|---|
| MgSal-$^{t}$Bu | 956 | - | 451 | -53 |
| Bi(3-py)$_3$ | - | 851 | 443 | -48 |
| **{Bi(3-py)$_3$·(MgSal-$^{t}$Bu)}** | 956 | 851 | 894 | **-7 and 5** |
| {Bi(3-py)$_3$·(MgSal-$^{t}$Bu)$_2$} | 956 | 851 | 1345 | 41 and 58 |
| {[Bi(3-py)$_3$]$_2$·(MgSal-$^{t}$Bu)$_2$} | 956 | 851 | 1788 | 87 and 110 |

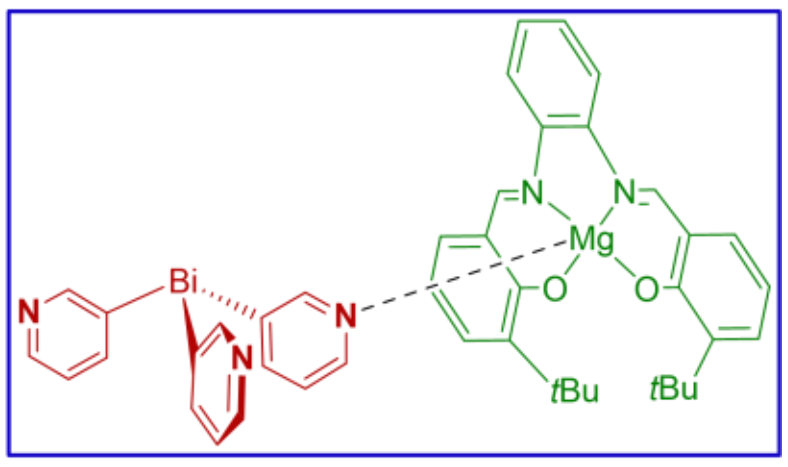


*Table S2. $ECC_{DSE}^{CDCl_3}$ was used to determine the $MW_{det}$ of **1**·MgSal-$^{t}$Bu in $CDCl_3$ at 298 K and the $MW_{Diff}$ for the proposed species. Note: The deviation is calculated as $M_{Diff} = [(MW_{cal}-MW_{det})/MW_{det}]\times100\%$ where $MW_{det}$ is the experimentally determined value and $MW_{cal}$ is the calculated molecular weight for the hypothetical species.*

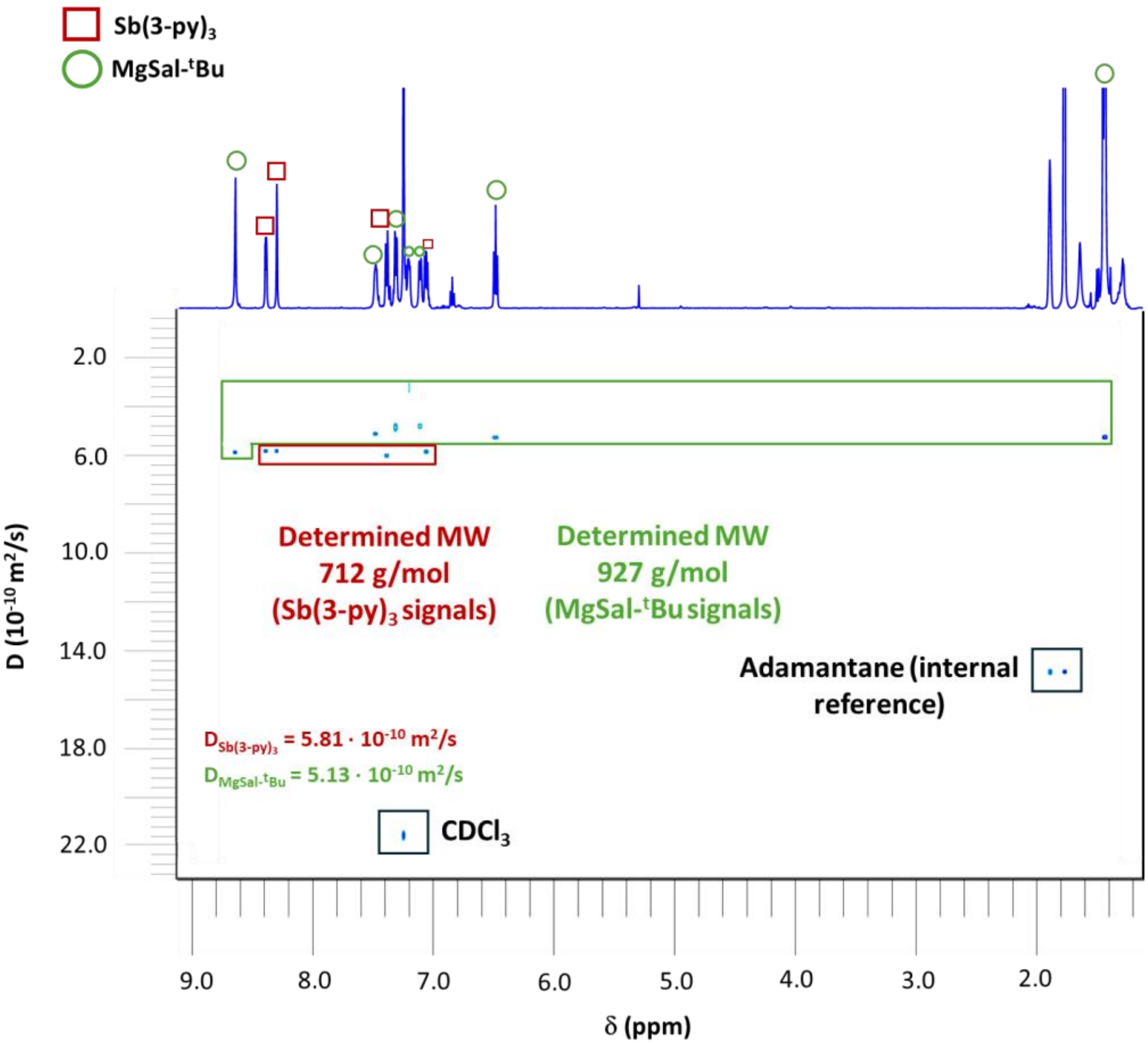


*Figure S47. $^{1}$H DOSY NMR spectrum of **2**·MgSal-$^{t}$Bu and adamantane (internal reference) at 298 K in $CDCl_3$. The diffusion coefficients (D) corresponding to the Sb(3-py)$_3$ and MgSal-$^{t}$Bu signals are highlighted in red and green, respectively.*

| Compound | *D* ($m^2$/s) | log *D* | log $D_{x,norm}$ | MW (g/mol) |
|---|---|---|---|---|
| **Adamantane** | 1.479E-09 | -8.8300 | - | 136.23[a] |
| **Sb(3-py)$_3$** | 5.808E-10 | -9.2360 | -9.2215 | 712[b] |
| **MgSal-$^t$Bu** | 4.993E-10 | -9.3017 | -9.2872 | 927[b] |

*Table S3. Molecular weight (MW) analysis using the $^1$H DOSY NMR data obtained for the mixture of **2**·MgSal-$^t$Bu and adamantane (internal reference) at 298 K in $CDCl_3$. [a]Real MW. [b]$MW_{det}$.*

| Aggregate | $MW_{det}$ (MgSal-$^t$Bu signals) | $MW_{det}$ (Sb(3-py)$_3$ signals) | $MW_{cal}$ (theoretical) | $MW_{Diff}$ (%) |
|---|---|---|---|---|
| MgSal-$^t$Bu | 927 | - | 451 | -51 |
| Sb(3-py)$_3$ | - | 712 | 356 | -50 |
| **{Sb(3-py)$_3$·(MgSal-$^t$Bu)}** | 927 | 712 | 807 | **-13 and 13** |
| {Sb(3-py)$_3$·(MgSal-$^t$Bu)$_2$} | 927 | 712 | 1258 | 36 and 77 |
| {[Sb(3-py)$_3$]$_2$·(MgSal-$^t$Bu)$_2$} | 927 | 712 | 1614 | 74 and127 |

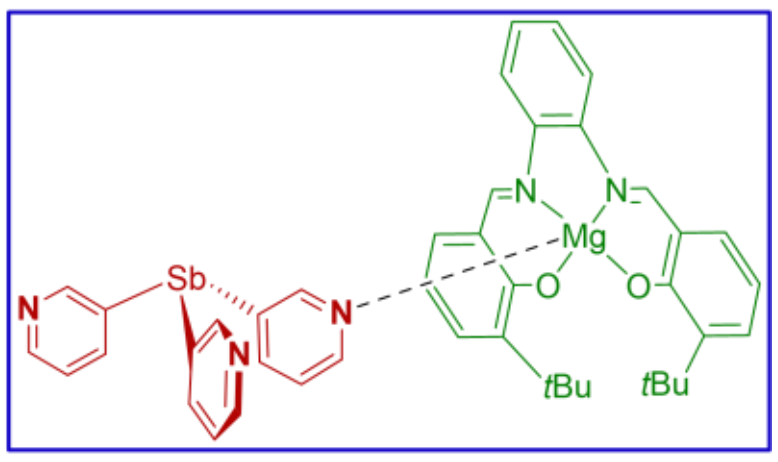


*Table S4. $ECC_{DSE}^{CDCl_3}$ was used to determine the $MW_{det}$ of **2**·MgSal-$^t$Bu in $CDCl_3$ at 298 K and the $MW_{Diff}$ for the proposed species. Note: The deviation is calculated as $M_{Diff} = [(MW_{cal}-MW_{det})/MW_{det}]\times100\%$, where $W_{det}$ is the experimentally determined value and $MW_{cal}$ is the calculated molecular weight for the hypothetical species.*

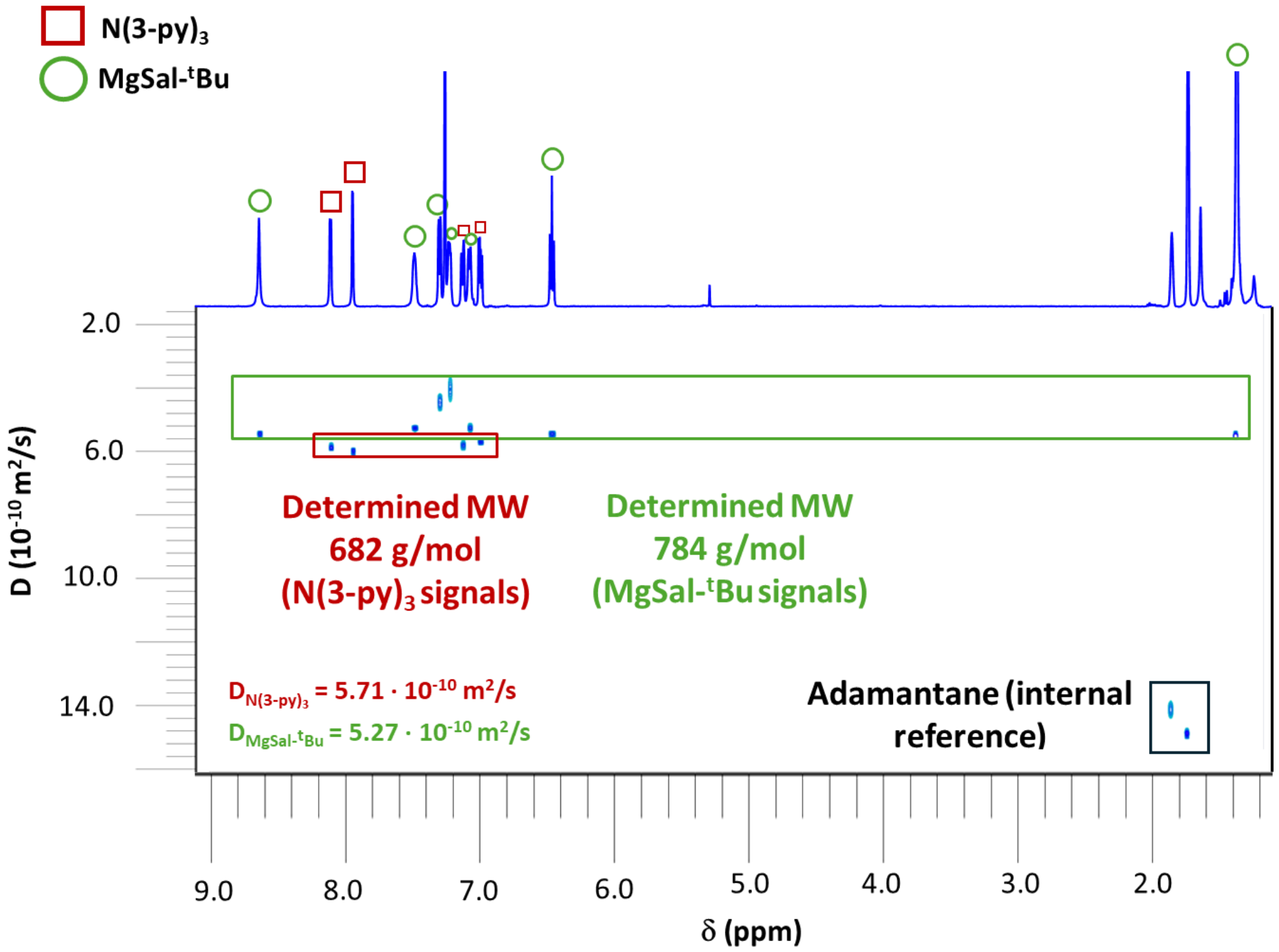


*Figure S48. $^{1}$H DOSY NMR spectrum of **3**·MgSal-$^{t}$Bu and adamantane (internal reference) at 298 K in $CDCl_3$. The diffusion coefficients (D) corresponding to the N(3-py)$_3$ and MgSal-$^{t}$Bu signals are highlighted in red and green, respectively.*

| Compound | *D* ($m^2/s$) | log *D* | log $D_{x,norm}$ | MW (g/mol) |
|---|---|---|---|---|
| **Adamantane** | 1.418E-09 | -8.8484 | - | 136.23[a] |
| **N(3-py)$_3$** | 5.705E-10 | -9.2437 | -9.2108 | 682[b] |
| **MgSal-$^{t}$Bu** | 5.265E-10 | -9.2786 | -9.2457 | 784[b] |

*Table S5. Molecular weight (MW) analysis using the $^{1}$H DOSY NMR data obtained for the mixture of **3**·MgSal-$^{t}$Bu and adamantane (internal reference) at 298 K in $CDCl_3$. [a]Real MW. [b]$MW_{det}$.*

| Aggregate | $MW_{det}$ (MgSal-$^{t}$Bu signals) | $MW_{det}$ (N(3-py)$_3$ signals) | $MW_{cal}$ (theoretical) | $MW_{Diff}$ (%) |
|---|---|---|---|---|
| MgSal-$^{t}$Bu | 784 | - | 451 | -42 |
| N(3-py)$_3$ | - | 682 | 248 | -64 |
| **{N(3-py)$_3$·(MgSal-$^{t}$Bu)}** | 784 | 682 | 699 | **-11 and 3** |
| {N(3-py)$_3$·(MgSal-$^{t}$Bu)$_2$} | 784 | 682 | 1150 | 47 and 69 |
| {[N(3-py)$_3$]$_2$·(MgSal-$^{t}$Bu)$_2$} | 784 | 682 | 1398 | 78 and 105 |

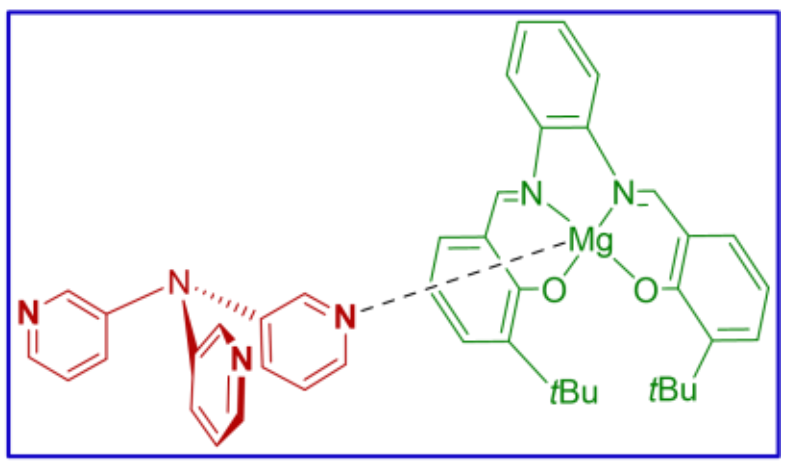


*Table S 6. $ECC_{DSE}^{CDCl_3}$ was used to determine the $MW_{det}$ of **3**·MgSal-$^{t}$Bu in $CDCl_3$ at 298 K and the $MW_{Diff}$ for the proposed species. Note: The deviation is calculated as $M_{Diff}$ = [($MW_{cal}$-$MW_{det}$)/$MW_{det}$]×100%, where $MW_{det}$ is the experimentally determined value and $MW_{cal}$ is the calculated molecular weight for the hypothetical species.*

## Binding data analysis

In order to study the aggregation state of the molecular capsules in solution, Job plots and titration studies were performed.

### Method of continuous variation (Job plot)

#### General procedure

The method of continuous variation was used to estimate the stoichiometry of the adducts **[E(3-py)$_3$·(ZnSal-$^t$Bu)$_3$]** and **[E(3-py)$_3$·(ZnSal-$^t$Bu-Br)$_3$] (E = N, Sb, Bi)**. 1 mM solutions of each ligand and salphen were prepared in $CDCl_3$. For each compound, the corresponding ligand and salphen were combined in 12 different molar fractions (Table S7). The resulting solutions (500 µL each one) were transferred to NMR tubes and then analyzed using $^1$H NMR spectroscopy.

| | $V_{E(3\text{-py})_3\text{ ligand}}$ / µL | $V_{salphen}$ / µL | $\chi_{E(3\text{-py})_3\text{ ligand}}$ | $\chi_{salphen}$ |
|---|---|---|---|---|
| **1** | 25 | 475 | 0.05 | 0.95 |
| **2** | 50 | 450 | 0.10 | 0.90 |
| **3** | 75 | 425 | 0.15 | 0.85 |
| **4** | 100 | 400 | 0.20 | 0.80 |
| **5** | 125 | 375 | 0.25 | 0.75 |
| **6** | 150 | 350 | 0.30 | 0.70 |
| **7** | 175 | 325 | 0.35 | 0.65 |
| **8** | 200 | 300 | 0.40 | 0.60 |
| **9** | 225 | 275 | 0.45 | 0.55 |
| **10** | 250 | 250 | 0.50 | 0.50 |
| **11** | 400 | 100 | 0.80 | 0.20 |
| **12** | 500 | 0 | 1.00 | 0.00 |

*Table S 7. Volume and molar fraction of the E(3-py)$_3$ (E = N, Sb, Bi) ligands and salphen complexes in the prepared solutions.*

The Job plots showed maxima at $\chi_{ligand}$ = 0.75 for the adducts of **1**, **2** and **3** with the salphens ZnSal-$^t$Bu and ZnSal-$^t$Bu-Br, confirming the expected 1:3 stoichiometry in $CDCl_3$ solution (Figures S49-S54).

## Job plot studies

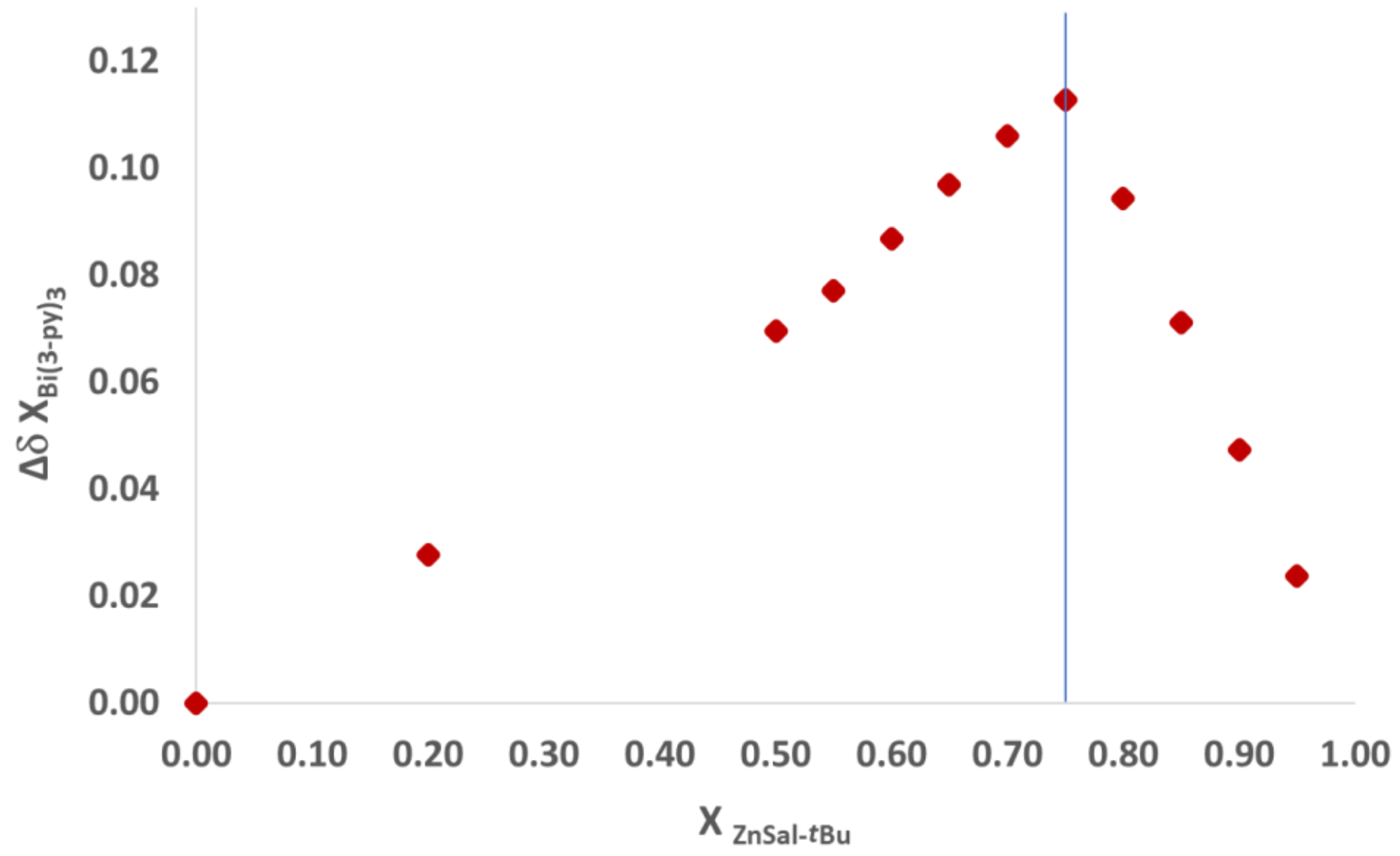


*Figure S49. Job plot study of **1**·ZnSal-[t]Bu showing a maximum at $\chi_{salphen}$ = 0.75. This corresponds to the formation of a complex with 1:3 stoichiometry.*

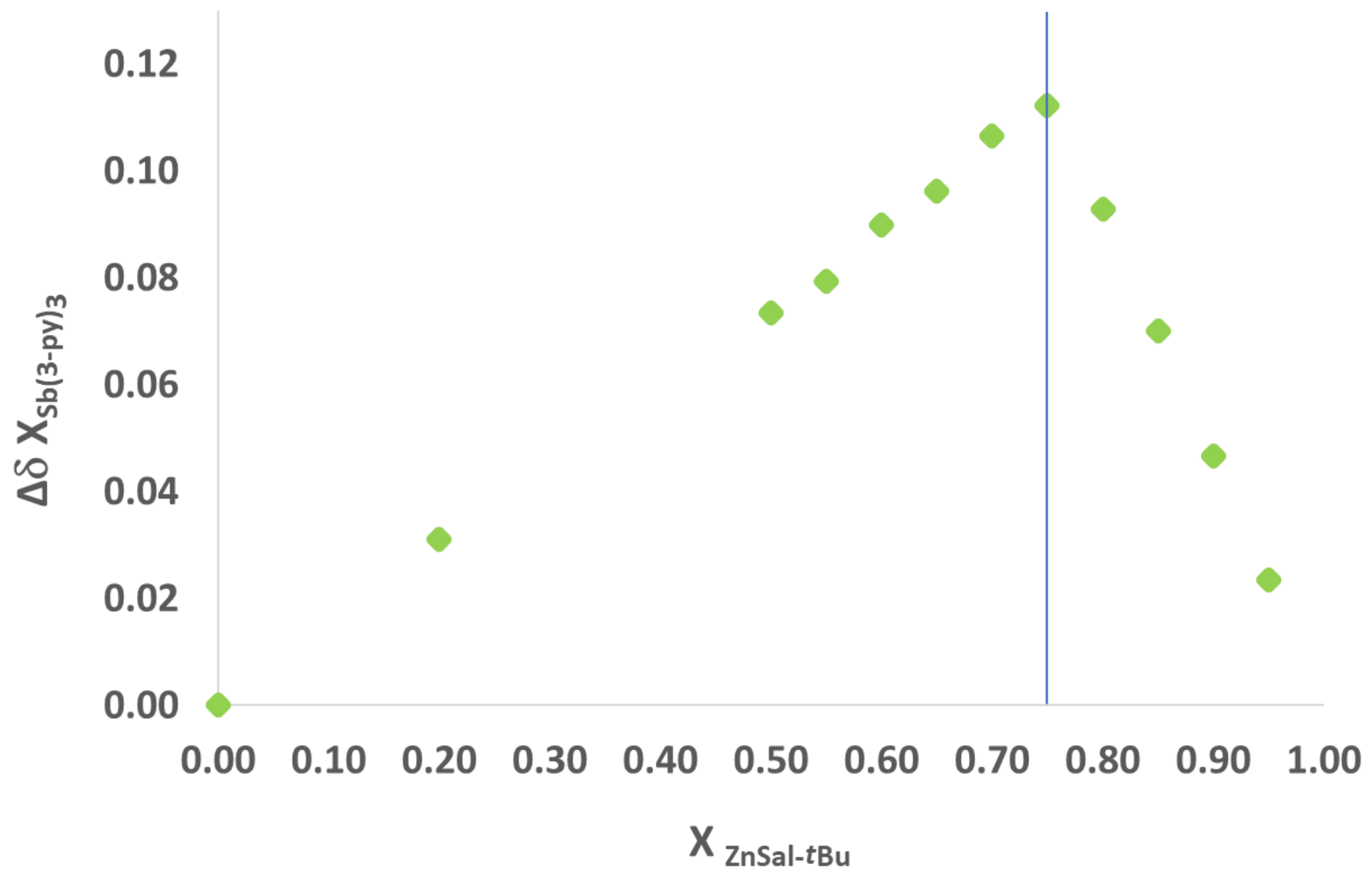


*Figure S50. Job plot study of **2**·ZnSal-[t]Bu, showing a maximum at $\chi_{salphen}$ = 0.75. This corresponds to the formation of a complex with 1:3 stoichiometry.*

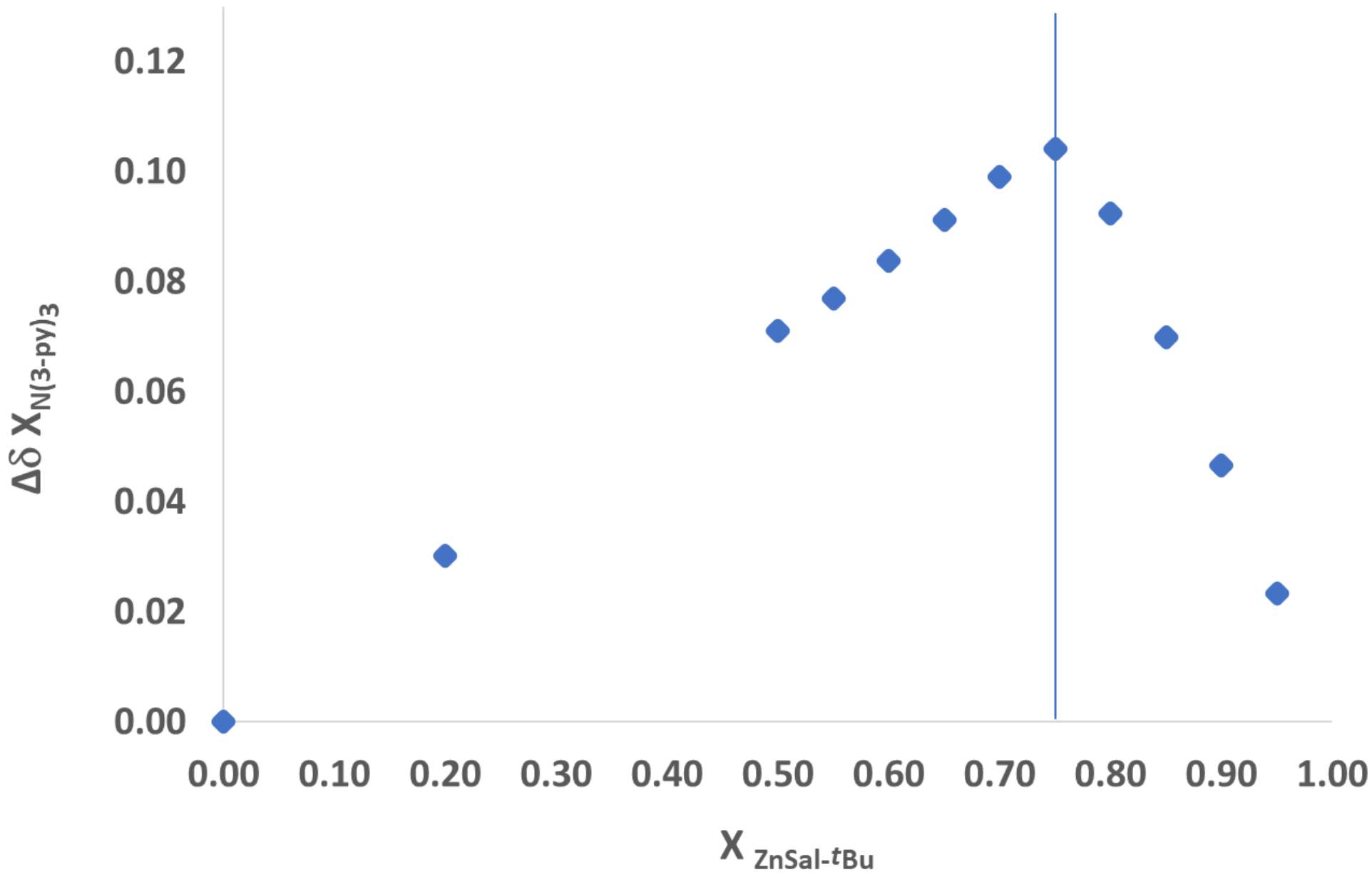


*Figure S51. Job plot study of **3**·ZnSal-[t]Bu, showing a maximum at $\chi_{salphen} = 0.75$. This corresponds to the formation of a complex with 1:3 stoichiometry.*

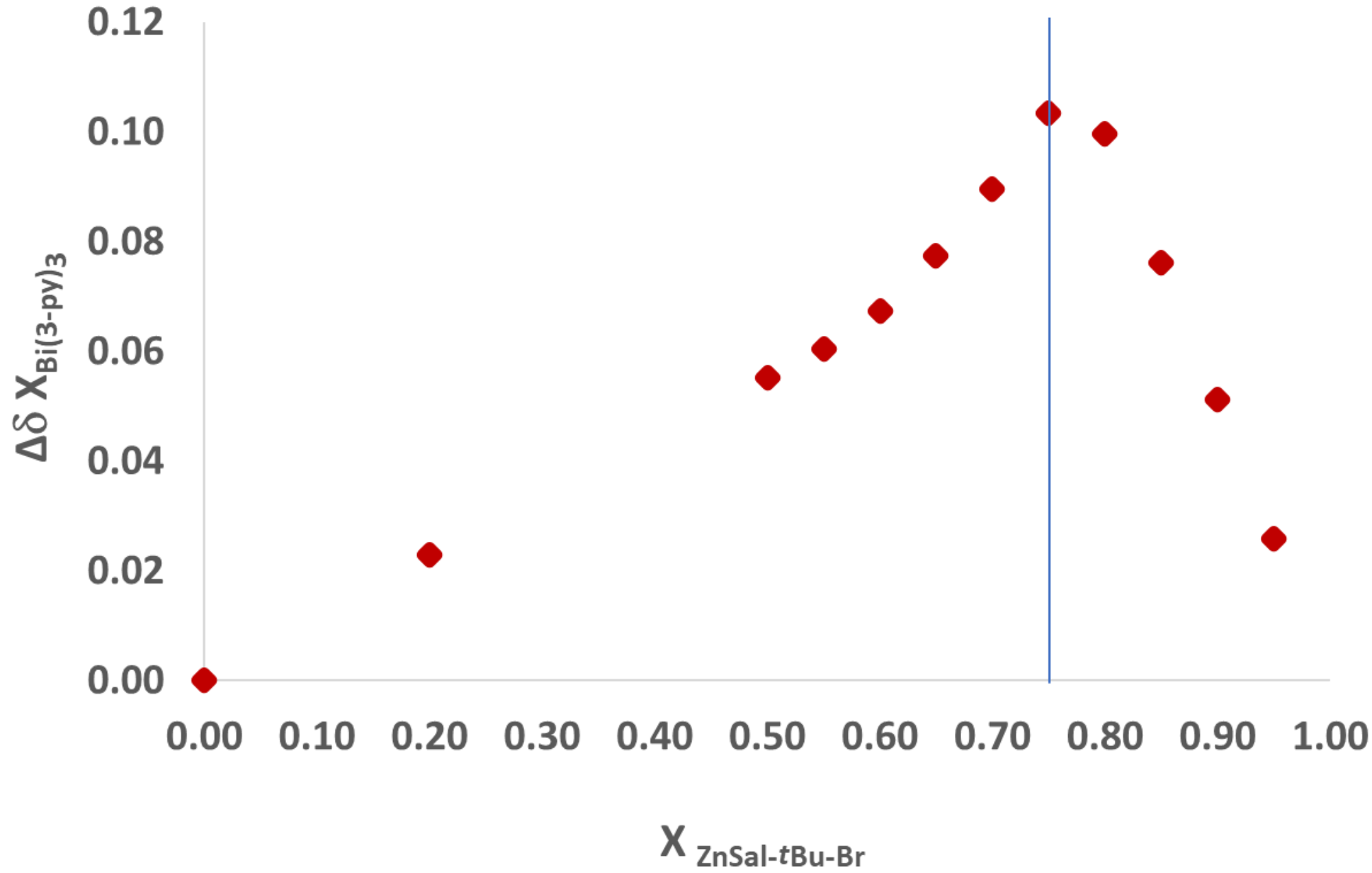


*Figure S52. Job plot study of **1**·ZnSal-[t]Bu-Br, showing a maximum at $\chi_{salphen} = 0.75$. This corresponds to the formation of a complex with 1:3 stoichiometry.*

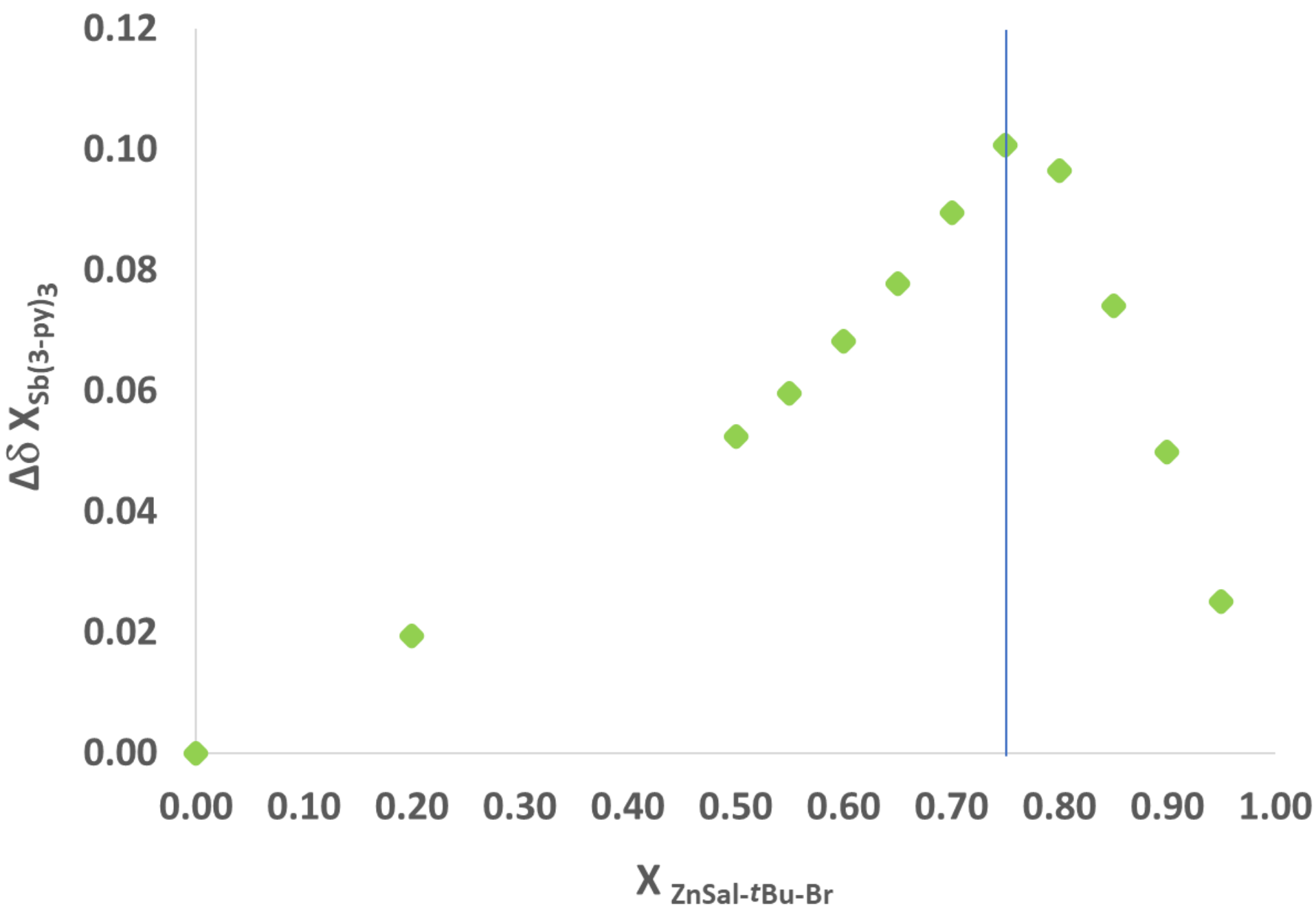


*Figure S53. Job plot study of **2**·ZnSal-$^t$Bu-Br, showing a maximum at $\chi_{salphen}$ = 0.75. This corresponds to the formation of a complex with 1:3 stoichiometry.*

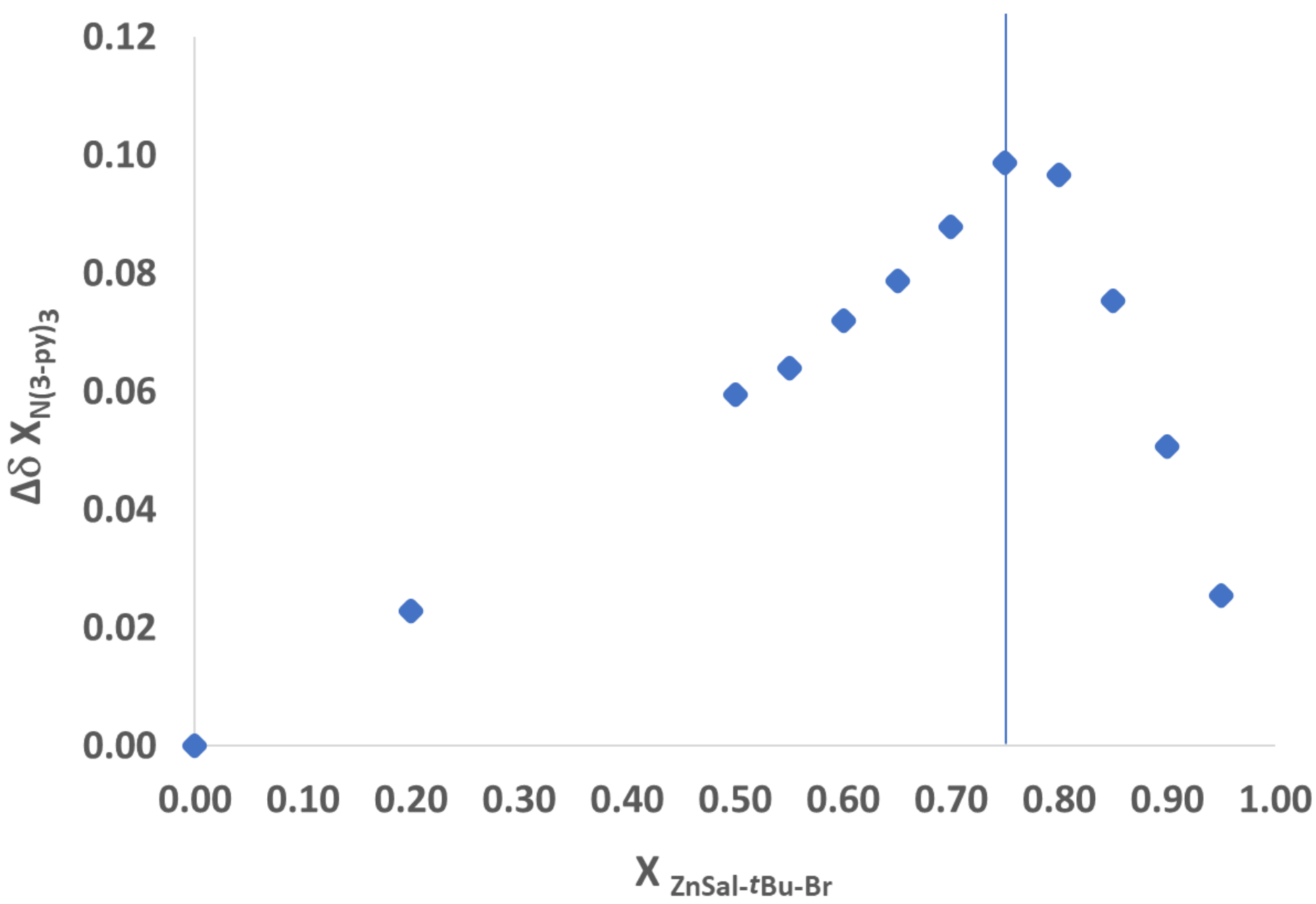


*Figure S54. Job plot study of **3**·ZnSal-$^t$Bu-Br, showing a maximum at $\chi_{salphen}$ = 0.75. This corresponds to the formation of a complex with 1:3 stoichiometry.*

## $^{1}$H NMR titrations

Association constants ($K_a$) of the ligands **1, 2** and **3** with the salphen ZnSal-$^t$Bu were estimated via $^{1}$H NMR titrations. Association constants for the adducts with the salphen ZnSal-$^t$Bu-Br could not be estimated due to solubility issues.

$10^{-3}$ M stock solutions of the salphen ZnSal-$^t$Bu and $10^{-4}$ M stock solutions of each ligand were prepared in $CD_3CN$ or $CDCl_3$. However, we were unable to obtain reliable association constants in $CDCl_3$, as the association constants obtained were quite large, reaching or exceeding the upper limit generally associated with this technique (*ca*. $10^5$ $M^{-1}$),[4,5] and gave unstable fittings with large uncertainties. These results are consistent with greater robustness of the Zn–salphen assemblies relative to their porphyrin-based counterparts. The estimation of $K_a$ in $CD_3CN$ is described below.

For each titration, 500 µL of the ligand solution was transferred to an NMR tube, and portions of the salphen stock solution were added to cover a wide range of molar ratios. After each addition, the $^{1}$H NMR spectrum was recorded at 298 K. The chemical shifts of selected protons were then plotted as a function of the molar fraction of the guest (salphen), and the resulting curve was fitted by a nonlinear method using the global analysis approach described below.

Fitting to 1:1 and 1:2 models

The NMR titration data was fitted to 1:1 and 1:2 binding models using Bindfit (supramolecular.org). The equations for these models have been detailed previously[4,5] and will not be repeated here. Links to all the fittings are provided below.

***Titration study of Bi(3-py)$_3$ vs ZnSal-$^t$Bu***

- 1:1 model:

https://app.supramolecular.org/bindfit/view/92642f84-0348-4876-95d5-2f2d3eff1a70

- 1:2 full model:

https://app.supramolecular.org/bindfit/view/89149980-bfe1-482a-abf3-37c747694625

- 1:2 additive model:

https://app.supramolecular.org/bindfit/view/79af9c2c-7c93-459b-9af0-452111a07ebf

- 1:2 non-cooperative model:

https://app.supramolecular.org/bindfit/view/c83bb836-d58b-468f-8c2f-c754039374aa

- 1:2 statistic model:

https://app.supramolecular.org/bindfit/view/ede9651d-843a-4f39-a35a-1b5c4c235e80

***Titration study of Sb(3-py)$_3$ vs ZnSal-$^t$Bu***

- 1:1 model:

https://app.supramolecular.org/bindfit/view/a725b802-5211-4cd4-8361-74b4df791e96

- 1:2 full model:

https://app.supramolecular.org/bindfit/view/be319684-4134-43d7-9212-9e2df9e3a715

- 1:2 additive model:

https://app.supramolecular.org/bindfit/view/4f0eaa74-81eb-4d89-8cc5-75630943246b

- 1:2 non-cooperative model:

https://app.supramolecular.org/bindfit/view/dbb2842b-0bf1-4005-b40e-7a9d6f816c48

- 1:2 statistic model:

https://app.supramolecular.org/bindfit/view/a472cedd-246e-4d09-bf6e-79f3ba32e0a6

***Titration study of $N(3\text{-py})_3$ vs ZnSal-$^t$Bu***

- 1:1 model:

https://app.supramolecular.org/bindfit/view/09ae42b5-0428-4824-85a0-37e86e4afd80

- 1:2 full model:

https://app.supramolecular.org/bindfit/view/539acd30-a1c3-481d-bcf1-7f3c62673c91

- 1:2 additive model:

https://app.supramolecular.org/bindfit/view/4535001d-bd5c-484a-be96-3aadef57095a

- 1:2 non-cooperative model:

https://app.supramolecular.org/bindfit/view/8b353e36-298d-442e-8b4f-4dcae91f724e

- 1:2 statistic model:

https://app.supramolecular.org/bindfit/view/942ab05f-156d-4757-802a-0a37fd033af5

Fitting to 1:3 models

The data was fitted to a 1:3 model using the approach previously described by Bownam–James, Thordarson, and co-workers in their $^1$H NMR study of the 1:3 association of a phosphate macrocycle adduct.[6,7] The association equilibrium constants and mass balance equations are as follows:

$$K_1 = \frac{[\mathrm{HG}]}{[\mathrm{H}][\mathrm{G}]} \quad \text{eq 1;} \qquad K_2 = \frac{[\mathrm{HG_2}]}{[\mathrm{HG}][\mathrm{G}]} \quad \text{eq 2;} \qquad K_1 = \frac{[\mathrm{HG_3}]}{[\mathrm{HG_2}][\mathrm{G}]} \quad \text{eq 3}$$

$$[\mathrm{H}]_0 = [\mathrm{H}] + [\mathrm{HG}] + [\mathrm{HG_2}] + [\mathrm{HG_3}] \quad \text{eq 4}$$

$$[\mathrm{G}]_0 = [\mathrm{G}] + [\mathrm{HG}] + 2[\mathrm{HG_2}] + 3[\mathrm{HG_3}] \quad \text{eq 5}$$

For the 1:2 and 1:3 stoichiometry models, four 'flavors' were considered. Each of these flavors is described below using the 1:3 stoichiometry as an example.

- Stepwise (non-degenerate) full 1:3 binding model. In this model, no assumptions are made and all the parameters ($K_1$, $K_2$, $K_3$, $\Delta\delta_{\Delta HG}$, $\Delta\delta_{\Delta HG2}$, $\Delta\delta_{\Delta HG3}$) are evaluated independently.

- Stepwise (non-degenerate), additive 1:3 binding model. In this case, it is assumed that $\Delta\delta_{\Delta HG}$ = 1/2 $\Delta\delta_{\Delta HG2}$ = 1/3 $\Delta\delta_{\Delta HG3}$. Therefore, the analysis is simplified, and only four parameters are fitted ($K_1$, $K_2$, $K_3$, $\Delta\delta_{\Delta HG}$).

- Stepwise non-cooperative 1:3 binding model. In this case, it is assumed that $K_1 = 3K_2 = 9K_3$. Defining $K_{1n}$ as $K_{1n} = K_1 = 3K_2 = 9K_3$, this model also furnishes four parameters to be fitted ($\Delta\delta_{\Delta HG}$, $\Delta\delta_{\Delta HG2}$, $\Delta\delta_{\Delta HG3}$, $K_{1n}$).

- Statistical 1:3 model. In this case, both assumptions are made, i.e., $K_1 = 3K_2 = 9K_3$ and $\Delta\delta_{\Delta HG}$ = 1/2 $\Delta\delta_{\Delta HG2}$ = 1/3 $\Delta\delta_{\Delta HG3}$. This provides the simplest fit, as only two parameters are evaluated ($\Delta\delta_{\Delta HG}$, $K_{1n}$).

All binding isotherms were systematically fit to all models and flavors, and the results are presented in Tables S8, S9, S10.

| Model | $K_1$ | $K_2$ | $K_3$ | SSR[a] | $SE_y$[b] | $cov_{fit}$ ratio[c] |
|---|---|---|---|---|---|---|
| **1-to-1** | 2.83E+03 | - | - | 9.59E-03 | 1.40E-02 | 1.00 |
| **1-to-2 full** | 1.26E+04 | 4.44E+03 | - | 4.00E-04 | 2.95E-03 | 22.64 |
| **1-to-2 additive** | 6.95E+03 | 4.90E+03 | - | 5.45E-03 | 1.07E-02 | 1.70 |
| **1-to-2 non-coop** | 1.70E+04 | 4.26E+03 | - | 4.08E-04 | 2.95E-03 | 22.13 |
| **1-to-2 statistical** | 8.22E+03 | 2.06E+03 | - | 7.48E-03 | 1.24E-02 | 1.29 |
| **1-to-3 full** | 1.59E+06 | 5.55E+03 | 1.78E+03 | 1.32E-04 | 1.75E-03 | 69.21 |
| **1-to-3 additive** | 9.90E+03 | 8.55E+03 | 7.37E+02 | 5.36E-03 | 1.07E-02 | 1.72 |
| **1-to-3 non-coop** | 1.93E+04 | 6.45E+03 | 2.15E+03 | 3.75E-04 | 2.89E-03 | 24.47 |
| **1-to-3 statistical** | 2.01E+04 | 6.70E+03 | 2.23E+03 | 5.74E-03 | 1.08E-02 | 1.65 |

*Table S8. Association constants ($M^{-1}$) of host **1** with guest ZnSal-$^t$Bu obtained using the different binding models.*

| Model | $K_1$ | $K_2$ | $K_3$ | SSR[a] | $SE_y$[b] | $cov_{fit}$ ratio[c] |
|---|---|---|---|---|---|---|
| **1-to-1** | 2.28E+03 | - | - | 5.66E-03 | 1.07E-02 | 1.00 |
| **1-to-2 full** | 4.36E+03 | 2.81E+03 | - | 1.86E-04 | 2.01E-03 | 29.34 |
| **1-to-2 additive** | 6.20E+03 | 2.83E+03 | - | 4.21E-03 | 9.36E-03 | 1.30 |
| **1-to-2 non-coop** | 1.06E+04 | 2.66E+03 | - | 2.41E-04 | 2.26E-03 | 22.40 |
| **1-to-2 statistical** | 6.28E+03 | 1.57E+03 | - | 4.81E-03 | 9.90E-03 | 1.17 |
| **1-to-3 full** | 3.92E+06 | 3.52E+03 | 1.57E+03 | 6.63E-05 | 1.24E-03 | 81.49 |
| **1-to-3 additive** | 6.62E+03 | 3.16E+03 | 6.79E+01 | 4.22E-03 | 9.47E-03 | 1.30 |
| **1-to-3 non-coop** | 1.31E+04 | 4.38E+03 | 1.46E+03 | 1.45E-04 | 1.80E-03 | 37.57 |
| **1-to-3 statistical** | 1.41E+04 | 4.71E+03 | 1.57E+03 | 4.24E-03 | 9.31E-03 | 1.30 |

*Table S9. Association constants ($M^{-1}$) of host **2** with guest ZnSal-$^t$Bu obtained using the different binding models.*

| Model | $K_1$ | $K_2$ | $K_3$ | SSR[a] | $SE_y$[b] | $cov_{fit}$ ratio[c] |
|---|---|---|---|---|---|---|
| **1-to-1** | 1.73E+03 | - | - | 3.18E-03 | 8.05E-03 | 1.00 |
| **1-to-2 full** | 1.87E+03 | 3.22E+03 | - | 3.62E-04 | 2.80E-03 | 8.36 |
| **1-to-2 additive** | 4.70E+03 | 2.77E+03 | - | 1.03E-03 | 4.63E-03 | 2.95 |
| **1-to-2 non-coop** | 9.93E+03 | 2.48E+03 | - | 4.19E-04 | 2.99E-03 | 7.15 |
| **1-to-2 statistical** | 4.60E+03 | 1.15E+03 | - | 2.32E-03 | 6.88E-03 | 1.38 |
| **1-to-3 full** | 3.47E+05 | 5.89E+02 | 1.84E-06 | 1.94E-04 | 2.12E-03 | 15.48 |
| **1-to-3 additive** | 4.70E+03 | 2.78E+03 | 4.21E-07 | 1.03E-03 | 4.68E-03 | 2.95 |
| **1-to-3 non-coop** | 5.41E+03 | 1.80E+03 | 6.01E+02 | 3.36E-04 | 2.73E-03 | 9.00 |
| **1-to-3 statistical** | 9.83E+03 | 3.28E+03 | 1.09E+03 | 1.43E-03 | 5.40E-03 | 2.23 |

*Table S10. Association constants ($M^{-1}$) of host **3** with guest ZnSal-$^t$Bu obtained using the different binding models.*

[a] SSR is the squared sum of residuals:

$$SSR = \sum (y_{\text{data}} - y_{\text{calc}})^2 \quad \text{eq 6}$$

[b] $SE_y$ is the error in the estimation of *y*:

$$SE_y = \sqrt{\frac{\sum (y_{\text{data}} - y_{\text{calc}})^2}{N - k}} \quad \text{eq 7}$$

[c]The $cov_{fit}$ ratio was calculated as:

$$\text{cov}_{\text{fit}} \text{ ratio} = \frac{\text{cov}_{\text{fit}}(\text{simplest model})}{\text{cov}_{\text{fit}}(\text{complex model})} \quad \text{eq 8}$$

The most significant model was chosen based on two important factors, namely, chemically meaningful values and statistical parameters.

Regarding the first criterion, only models providing chemically meaningful association constants were considered. Therefore, the magnitude of all *K* values was also carefully examined. Models yielding negative *K* values, unrealistic variations between consecutive stepwise constants, or unrealistic association constants were discarded. A representative example is the full 1:3 model for host 3 (Table S10), in which the stepwise constants decrease by three orders of magnitude from $K_1$ to $K_2$, followed by an unrealistic drop of eight orders of magnitude to give a negligible $K_3$. Such inconsistent variations are not chemically meaningful for describing the binding behavior of the species under study, despite the apparently good statistical descriptors obtained for this model.

Regarding the second factor, the best models were chosen based on the lowest fitting errors (SSR and SEy) and, more importantly, on the highest covariance fit ratios (i.e., the ratio between the covariance of fit for a complex model with respect to a model of reference, which is usually the simplest 1:1 stoichiometry model). Typically, a ratio of 3 to 5 or greater justifies the selection of the complex model/flavor over the simpler one, as this indicates that the complex model describes the data significantly better.[8] This statistical comparison was applied only to the chemically meaningful models retained after the initial screening described above.

In light of the above rationale, the best fit for *all* hosts (ligands **1, 2** and **3**) was found to be the 1:3 non-cooperative model. This indicates that binding of one ZnSal-$^t$Bu unit does not significantly affect coordination at the remaining sites, so that the process can be seen as three independent events.

| **Ligand / Salphen** | **ZnSal-$^t$Bu** |
|---|---|
| **Bi(3-py)$_3$** | $K_1 = (1.93 \pm 0.01) \cdot 10^4$ |
| | $K_2 = (6.45 \pm 0.04) \cdot 10^3$ |
| | $K_3 = (2.15 \pm 0.01) \cdot 10^3$ |
| **Sb(3-py)$_3$** | $K_1 = (1.31 \pm 0.01) \cdot 10^4$ |
| | $K_2 = (4.38 \pm 0.03) \cdot 10^3$ |
| | $K_3 = (1.46 \pm 0.01) \cdot 10^3$ |
| **N(3-py)$_3$** | $K_1 = (5.41 \pm 0.04) \cdot 10^3$ |
| | $K_2 = (1.80 \pm 0.02) \cdot 10^3$ |
| | $K_3 = (6.01 \pm 0.05) \cdot 10^2$ |

*Table S11. Binding constants [$M^{-1}$] for the chosen most significant binding models (1:3 non-cooperative). The reported uncertainties correspond to fitting uncertainties estimated by Monte Carlo simulations and not to standard deviations from replicate measurements.*[8,9]

### *Titration study of Bi(3-py)$_3$ vs ZnSal-$^t$Bu*

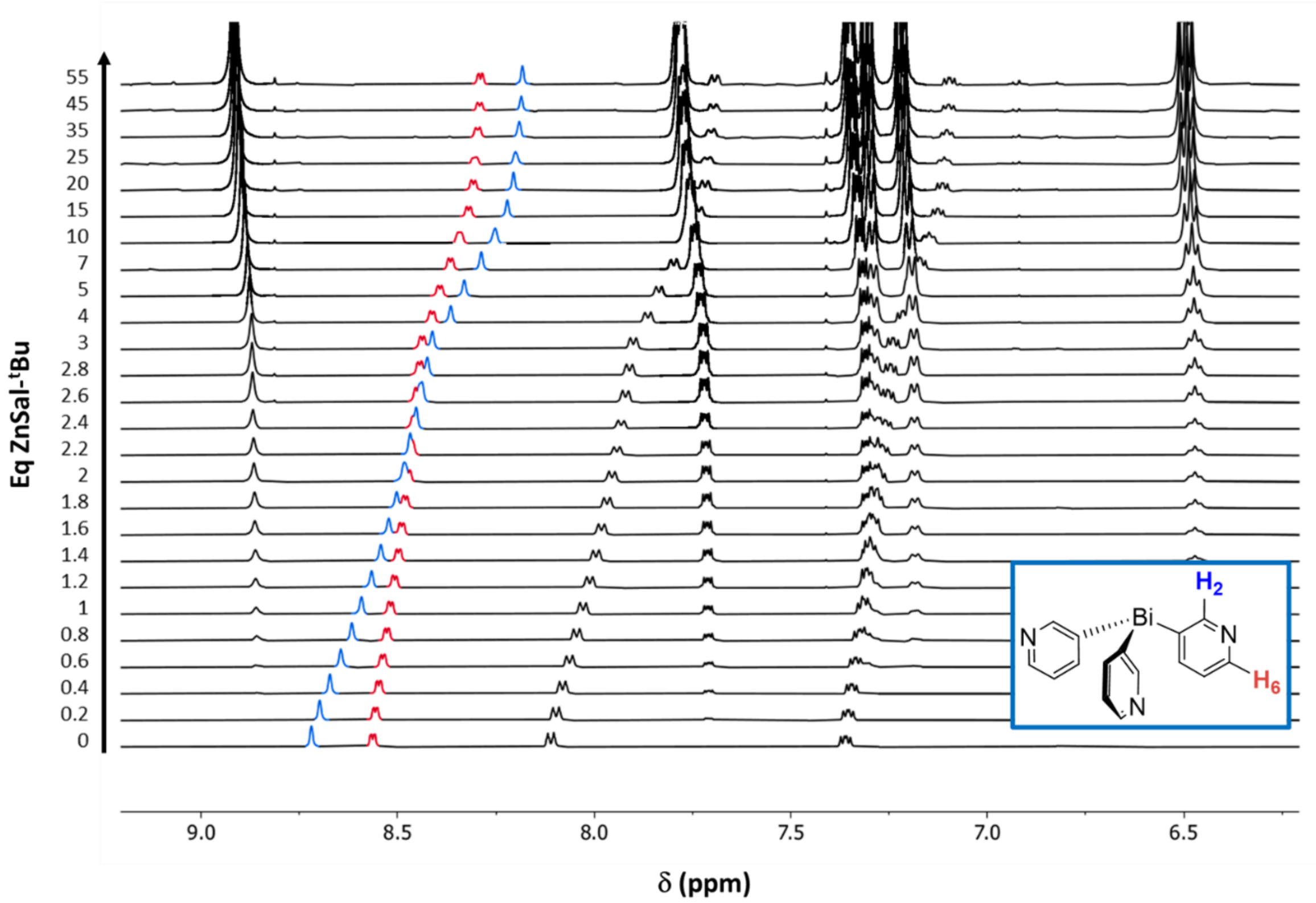


*Figure S55. Stacked $^1H$ NMR spectra of the titration of ligand **1** with up to 55 equivalents of ZnSal-$^t$Bu in $CD_3CN$. The signals of the ligands that were followed are indicated in blue ($H_2$) and red ($H_6$), respectively.*

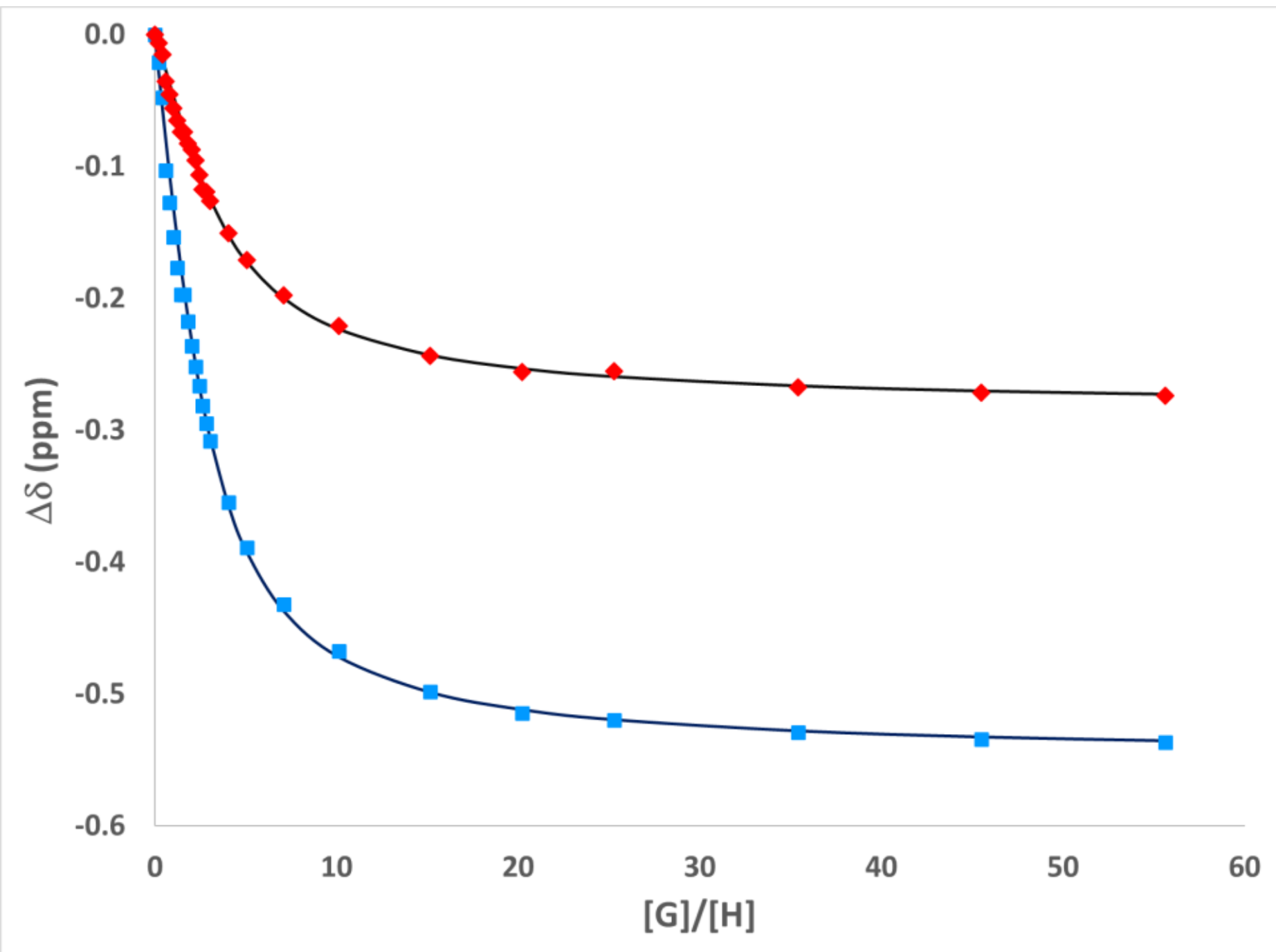


*Figure S56. Changes in the chemical shifts of the Bi(3-py)$_3$ signals ($H_2$ in blue and $H_6$ in red) vs [G]/[H], and theoretical values obtained from the non-linear regression to a 1:3 binding isotherm non-cooperative model (black lines).*

### *Titration study of Sb(3-py)$_3$ vs ZnSal-$^t$Bu*

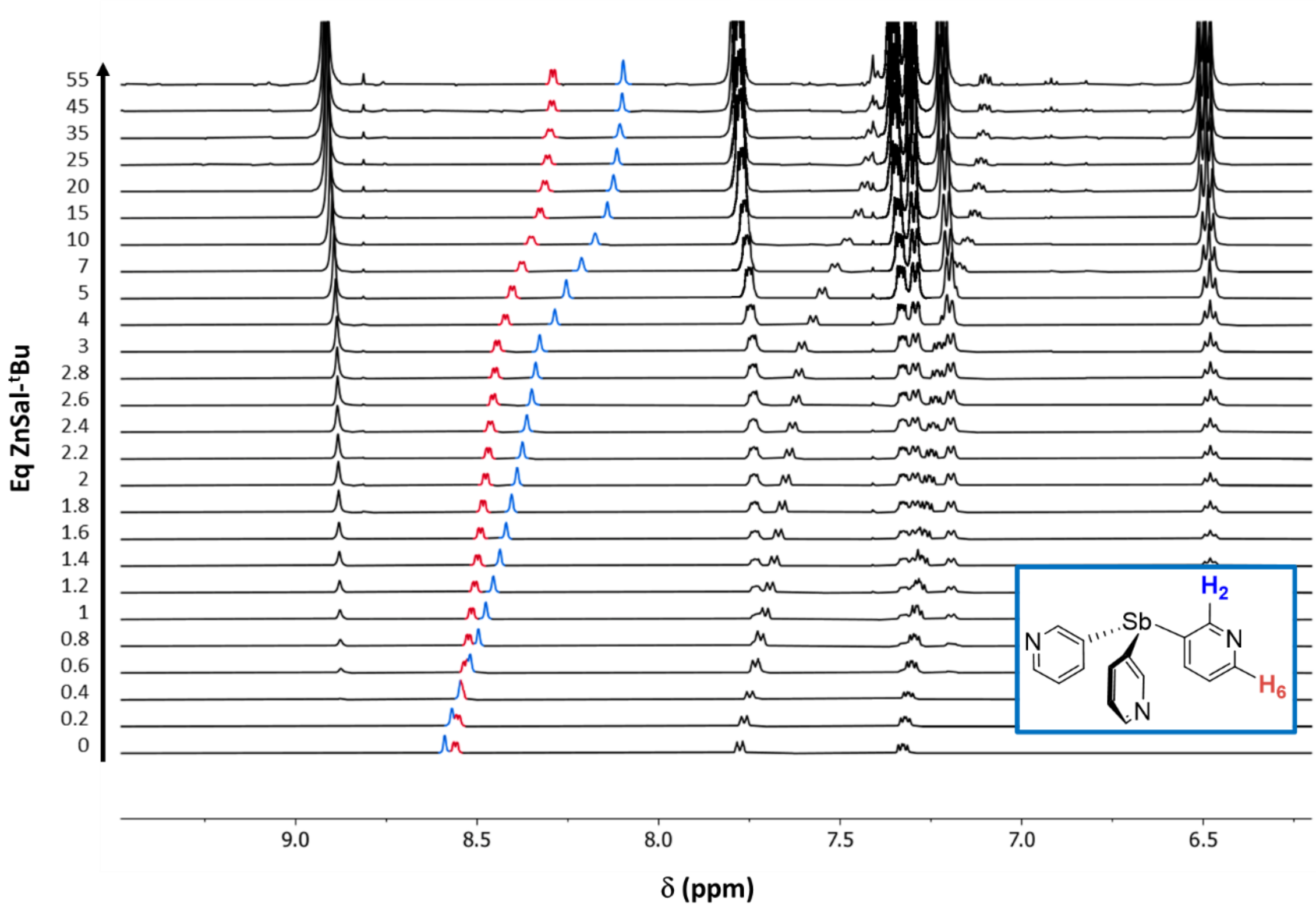


*Figure S57. Stacked $^1H$ NMR spectra of the titration of ligand **2** with up to 55 equivalents of ZnSal-$^t$Bu in $CD_3CN$. The signals of the ligands that were followed are indicated in blue ($H_2$) and red ($H_6$), respectively.*

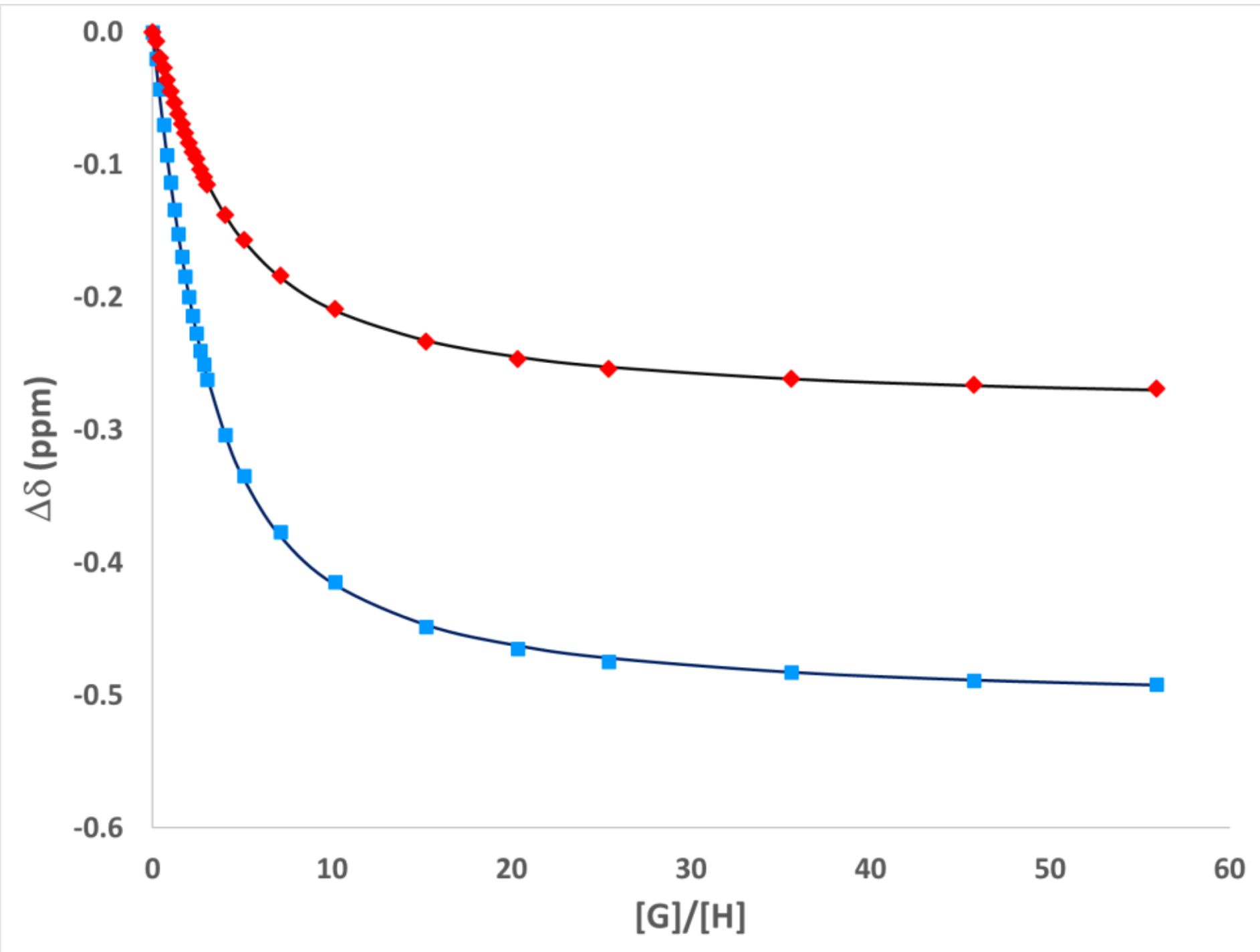


*Figure S58. Changes in the chemical shifts of the Sb(3-py)$_3$ signals ($H_2$ in blue and $H_6$ in red) vs [G]/[H], and theoretical values obtained from the non-linear regression to a 1:3 binding isotherm non-cooperative model (black lines).*

### *Titration study of N(3-py)$_3$ vs ZnSal-$^t$Bu*

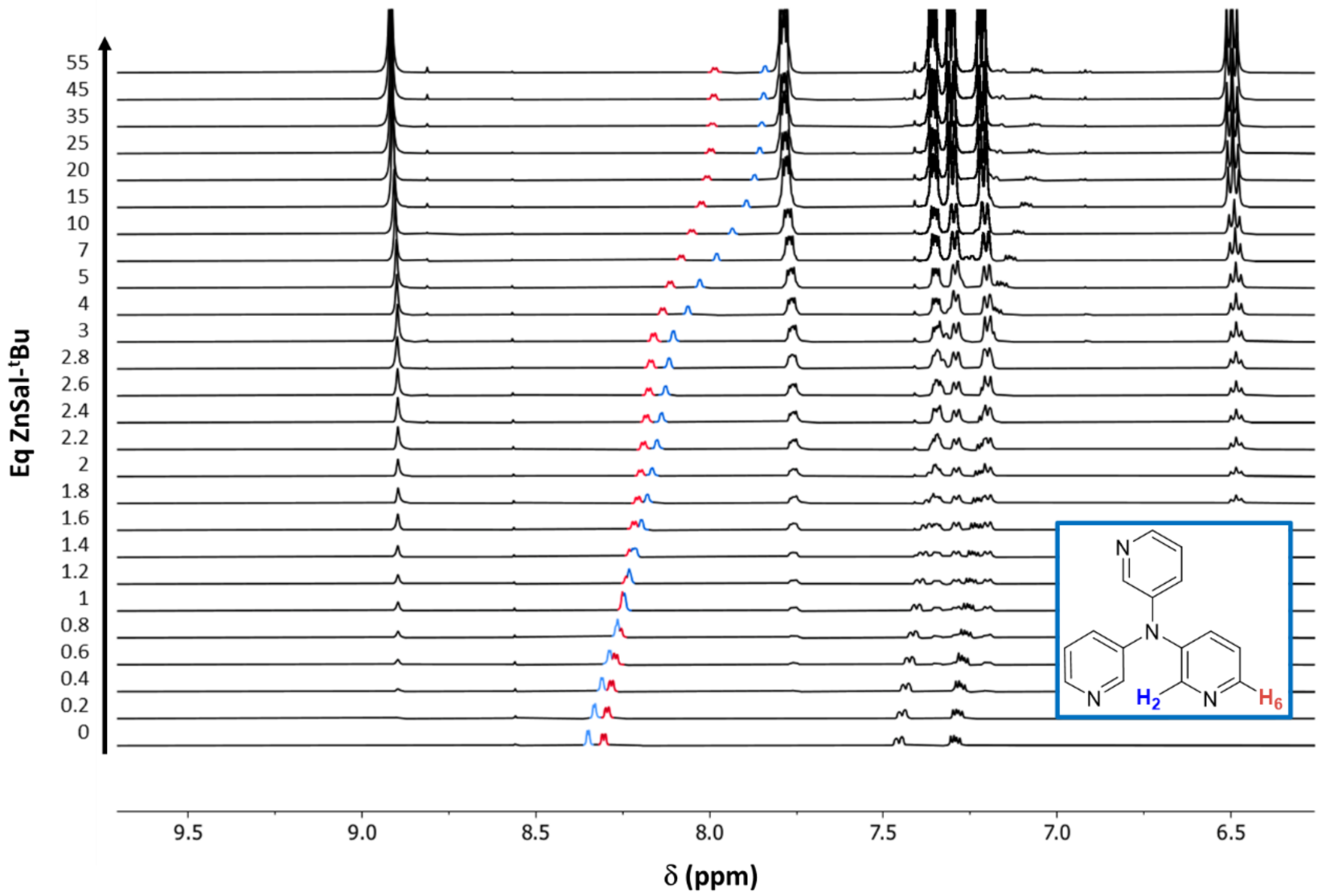


*Figure S59. Stacked $^1$H NMR spectra of the titration of ligand **3** with up to 55 equivalents of ZnSal-$^t$Bu in $CD_3CN$. The signals of the ligands that were followed are indicated in blue ($H_2$) and red ($H_6$), respectively.*

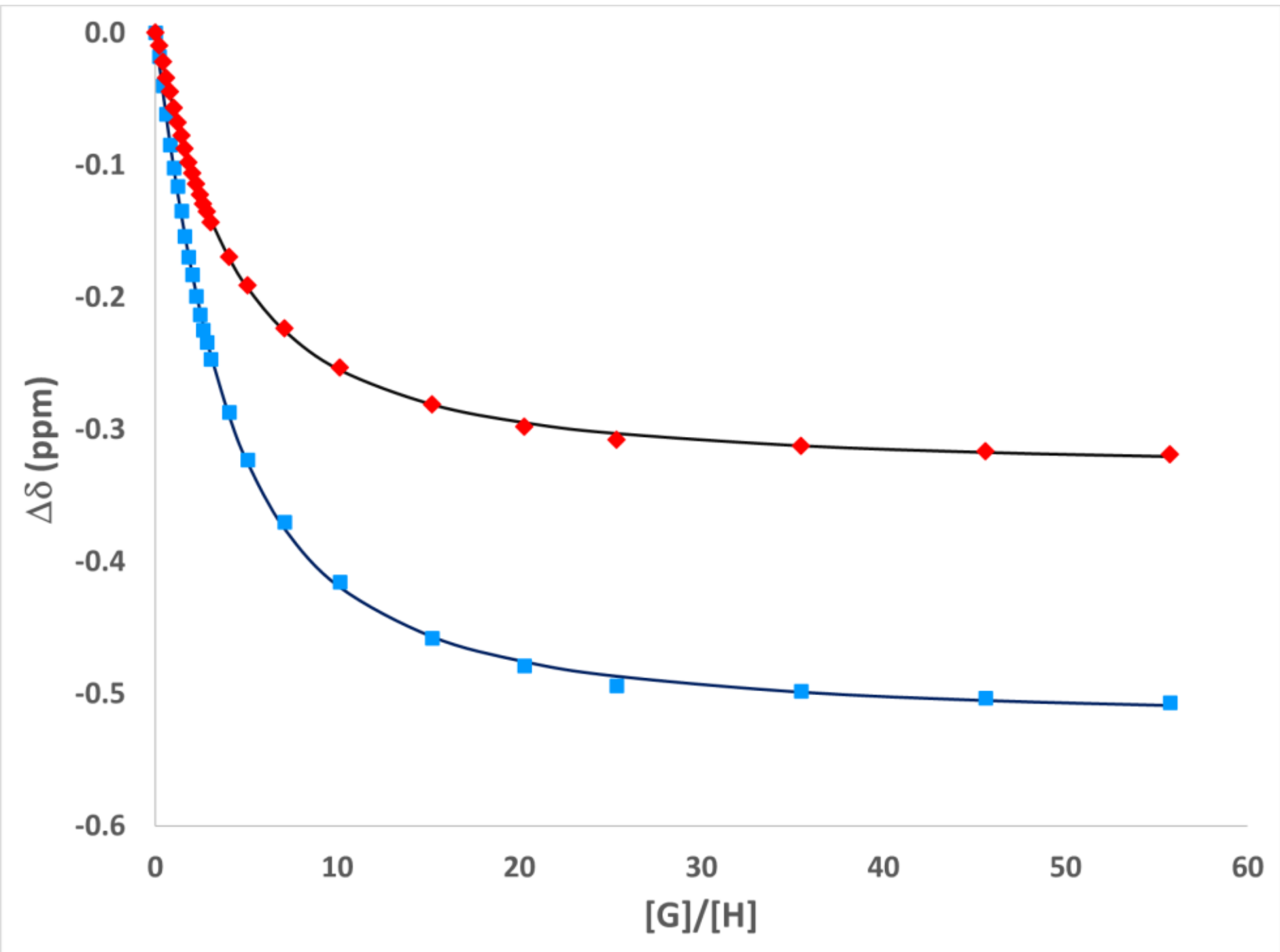


*Figure S60. Changes in the chemical shifts of the N(3-py)$_3$ signals ($H_2$ in blue and $H_6$ in red) vs [G]/[H], and the theoretical values obtained from the non-linear regression to a 1:3 binding isotherm non-cooperative model (black lines).*

## Variable temperature experiments

The room-temperature $^1H$ NMR spectra of capsules **1**·ZnSal-$^t$Bu and **2**·ZnSal-$^t$Bu both display a remarkably simple pattern, consisting of a single set of signals for ligand **1** or **2** and one set for the Zn-salphen units. This indicates that the observed solid-state conformations are averaged in solution, resulting in effective $C_{3v}$ and $C_{2v}$ symmetries for the tris(3-pyridyl) ligand and the Zn-salphen unit. Variable-temperature (VT) $^1H$ NMR experiments further reveal that the interconversion between conformations remains fast on the NMR timescale, even at 218 K in $CDCl_3$ (Figure S61 and Figure S62). This dynamic behaviour may proceed through intra and/or intermolecular pathways. VT NMR studies of a mixture of **1**·ZnSal-$^t$Bu or **2**·ZnSal-$^t$Bu and ZnSal-$^t$Bu (3 equivalents) in $CDCl_3$ showed that pyridine coordination and decoordination becomes slow on the NMR timescale at 218 K, giving rise to the expected resonances for the intact capsule and free ZnSal-$^t$Bu (Figure S63 and Figure S64). These observations suggest that the capsules **1**·ZnSal-$^t$Bu and **2**·ZnSal-$^t$Bu retain a significant degree of dynamicity in solution, which arises primarily from intramolecular processes (*e.g.* rotation around the Zn–N(py) and C–E bonds) rather than from pyridine dissociation. Consequently, the solid-state conformations appear to be averaged in solution without requiring ligand decoordination events.

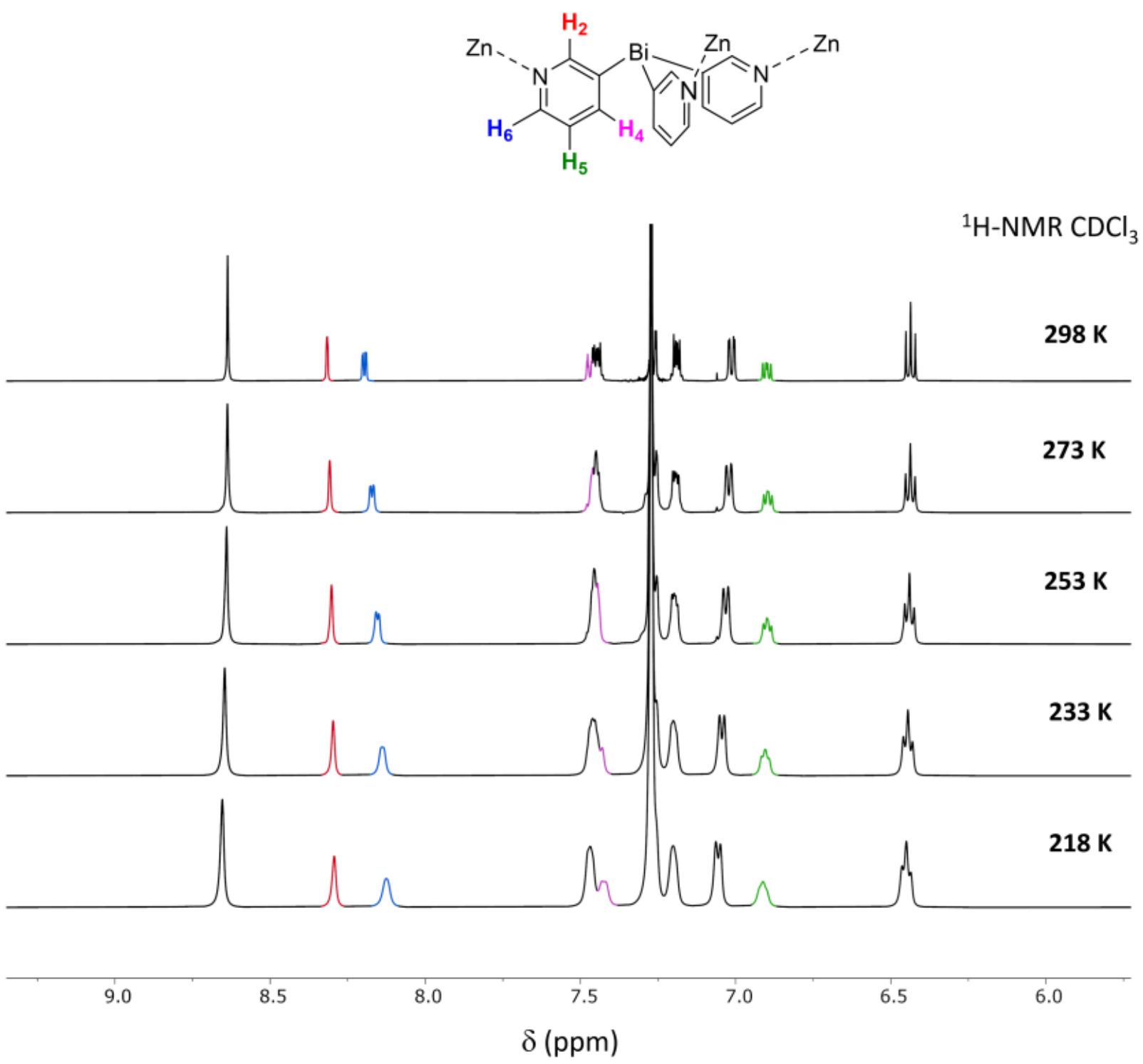


*Figure S61. $^1H$ NMR ($CDCl_3$, 500 MHz) spectra of **1**·ZnSal-$^t$Bu at 298 K, 273 K, 253 K, 233 K and 218 K. As the temperature decreases, the peaks broaden, but no conformers were identified due to a rapid equilibrium between them in solution.*

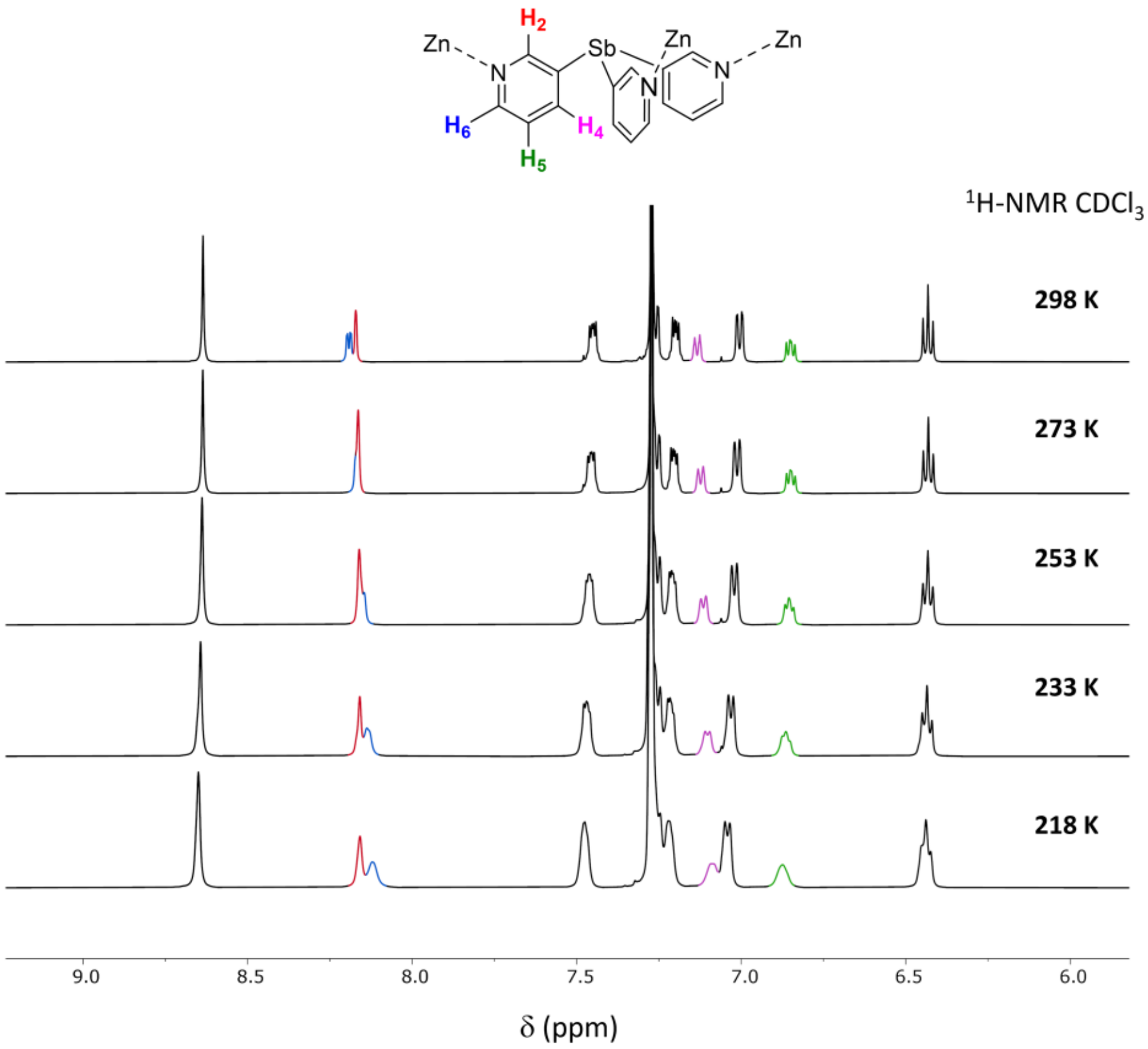


*Figure S62. $^{1}$H NMR (CDCl$_3$, 500 MHz) spectra of **2**·ZnSal-$^t$Bu at 298 K, 273 K, 253 K, 233 K and 218 K. As the temperature decreases, the peaks broaden, but no conformers were identified due to a rapid equilibrium between them in solution.*

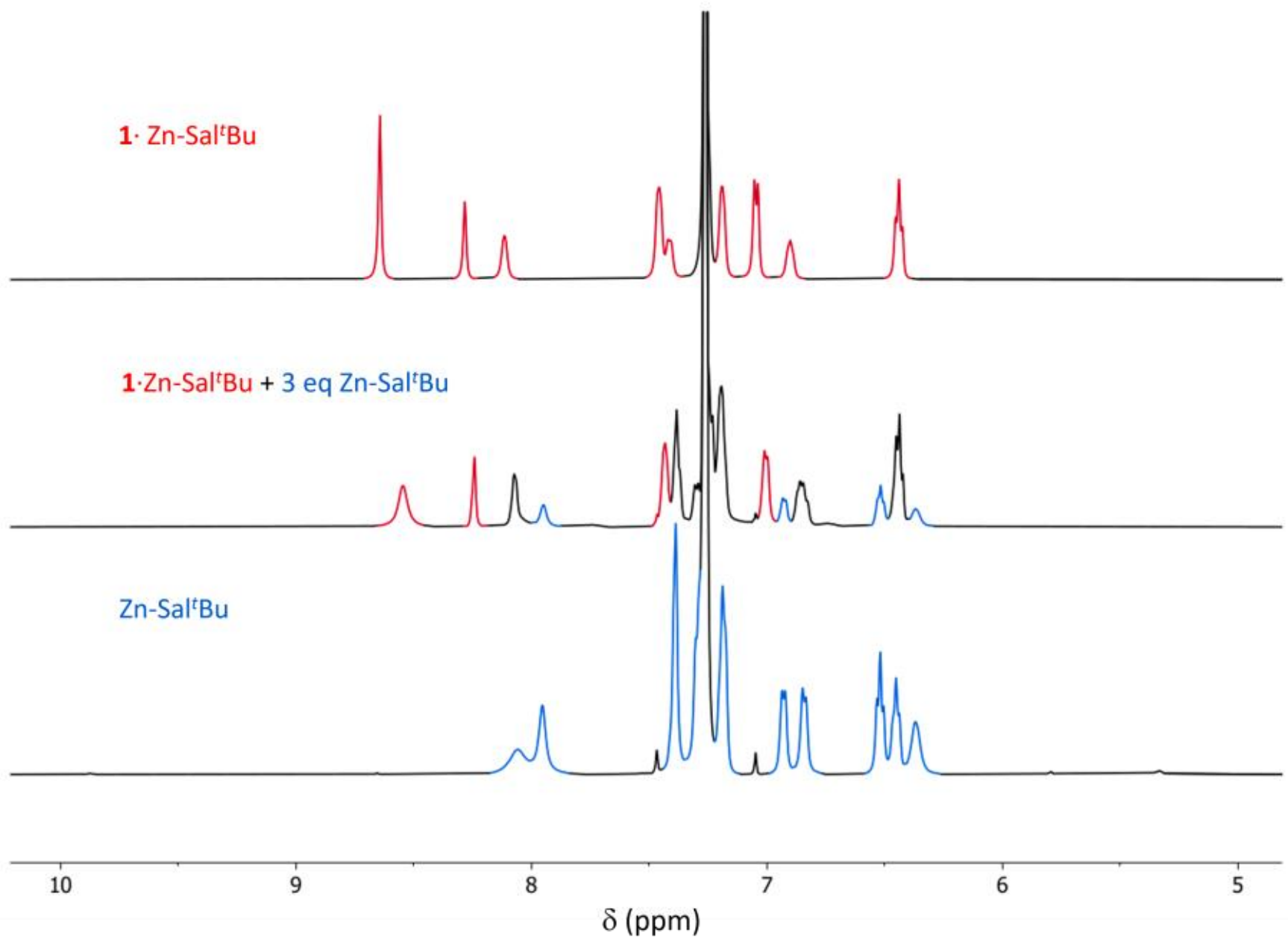


*Figure S63. $^{1}$H NMR spectra at 218 K in CDCl$_3$ of capsule **1**·ZnSal-$^t$Bu (top), a mixture of **1**·ZnSal-$^t$Bu and 3 equivalents of ZnSal-$^t$Bu (middle) and ZnSal-$^t$Bu (bottom). Although exchange between bound and free ZnSal-$^t$Bu becomes slow on the NMR timescale at 218 K, as evidenced by the presence of distinct resonances for each species, the capsule signals do not show any additional splitting. Instead, the spectra retain one set of resonances for ligand **1** and one set for the Zn-salphen unit, indicating that conformational averaging remains operative in solution. Note: Some signals are overlapped, and they are therefore not highlighted in red or blue.*

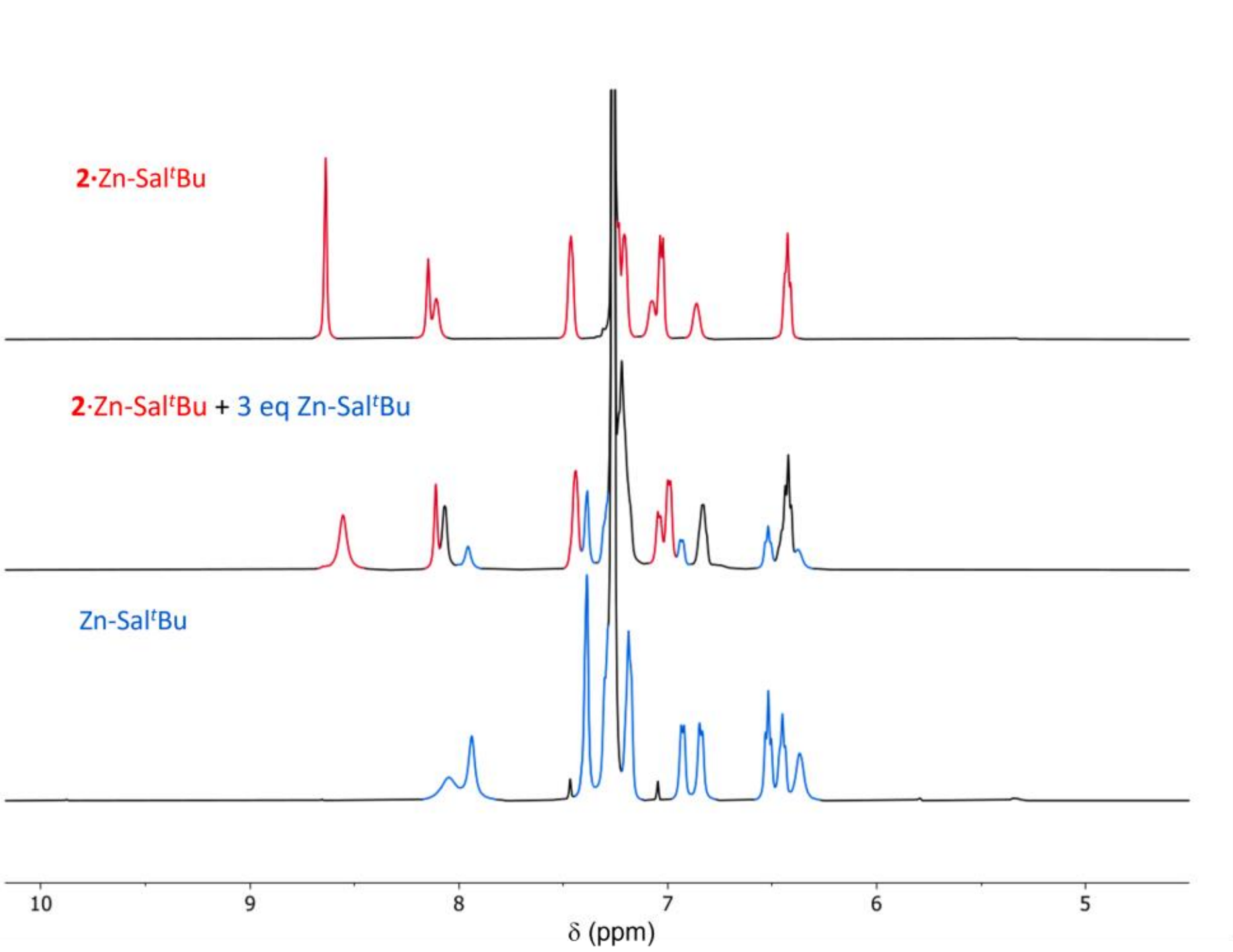


*Figure S 64. $^1$H NMR spectra at 218 K in $CDCl_3$ of capsule **2**·ZnSal-$^t$Bu (top), a mixture of **2**·ZnSal-$^t$Bu and 3 equivalents of ZnSal-$^t$Bu (middle) and ZnSal-$^t$Bu (bottom). Although exchange between bound and free ZnSal-$^t$Bu becomes slow on the NMR timescale at 218 K, as evidenced by the presence of distinct resonances for each species, the capsule signals do not show any additional splitting. Instead, the spectra retain one set of resonances for ligand **2** and one set for the Zn-salphen unit, indicating that conformational averaging remains operative in solution. Note: Some signals are overlapped, and they are therefore not highlighted in red or blue.*

# Computational details

## NBO analysis

A dimeric fragment of the **1**·ZnSal polymer, comprising two adjacent {Bi(3-py)$_3$·(ZnSal)} monomeric units extracted from the X-ray structure, was used as a structural model to analyse the Bi···O interactions (Figure S65). The Bi···O distances within this fragment are significantly shorter than the sum of the van der Waals radii of Bi and O $\sum_{VDW}(Bi-O)$= 3.59 Å,[10] indicating the presence of intermolecular Bi···O contacts (Table S12).

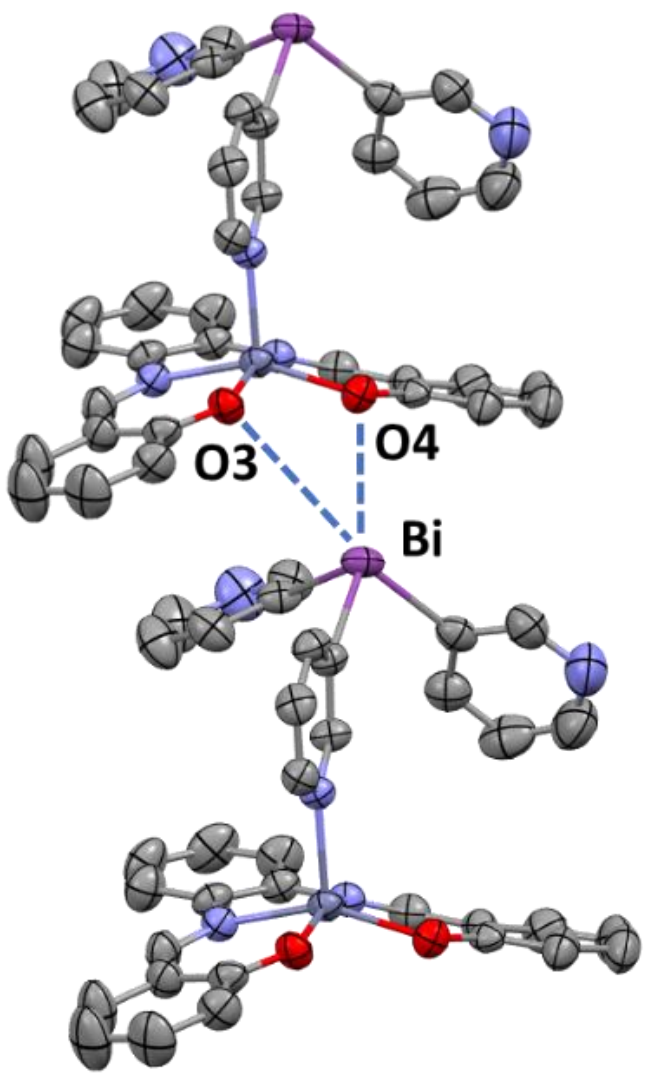


*Figure S65. Dimeric fragment of the **1**·ZnSal polymer, [Bi(3-py)$_3$·(ZnSal)]$_2$, extracted from the X-ray structure and used to analyse the intermolecular Bi···O interactions. Displacement ellipsoids at 50% probability. H atoms are omitted for clarity. Colour key: C (grey), N (lilac), O (red), Bi (dark purple) and Zn (greyish blue).*

| Bi–O3 distance (Å) | Bi–O4 distance (Å) |
|---|---|
| 3.157 | 3.073 |

*Table S12. Bi–O distances of [Bi(3-py)$_3$·(ZnSal)]$_2$ in the X-ray structure.*

Charge transfer energies between oxygen and bismuth are shown in Table S13. NBO analysis of the **1**·ZnSal dimer shows a significant overlap between unoccupied σ*(Bi–C$_{py}$) orbital and the lone pair of a single oxygen from an adjacent salphen unit (Figure S66).

| Donor NBO | Acceptor NBO | *E* (kcal / mol) |
|---|---|---|
| LP (1) O3 | BD*(1)Bi 71 – C78 | 0.48 |
| LP (1) O3 | BD*(1)Bi 71 – C83 | 0.19 |
| **LP (1) O3** | **BD*(1)Bi 71 – C89** | **2.30** |
| LP (3) O3 | BD*(1)Bi 71 – C78 | 0.27 |
| LP (3) O3 | BD*(1)Bi 71 –C83 | 0.48 |
| **LP (3) O3** | **BD*(1)Bi 71 – C89** | **1.19** |
| **LP (1) O4** | **BD*(1)Bi 71 – C78** | **4.15** |
| LP (1) O4 | BD*(1)Bi 71 – C83 | 0.08 |
| LP (1) O4 | BD*(1)Bi 71 – C89 | 0.05 |
| LP (2) O4 | BD*(1)Bi 71 – C83 | 0.07 |
| **LP (3) O4** | **BD*(1)Bi 71 – C78** | **2.20** |
| LP (3) O4 | BD*(1)Bi 71 – C83 | 0.54 |
| **Sum (O3)** | | **4.91** |
| **Sum (O4)** | | **7.09** |

*Table S13. Charge transfer energies between donor and acceptor NBOs that involve Bi and O. Rydberg contributions have been omitted.*

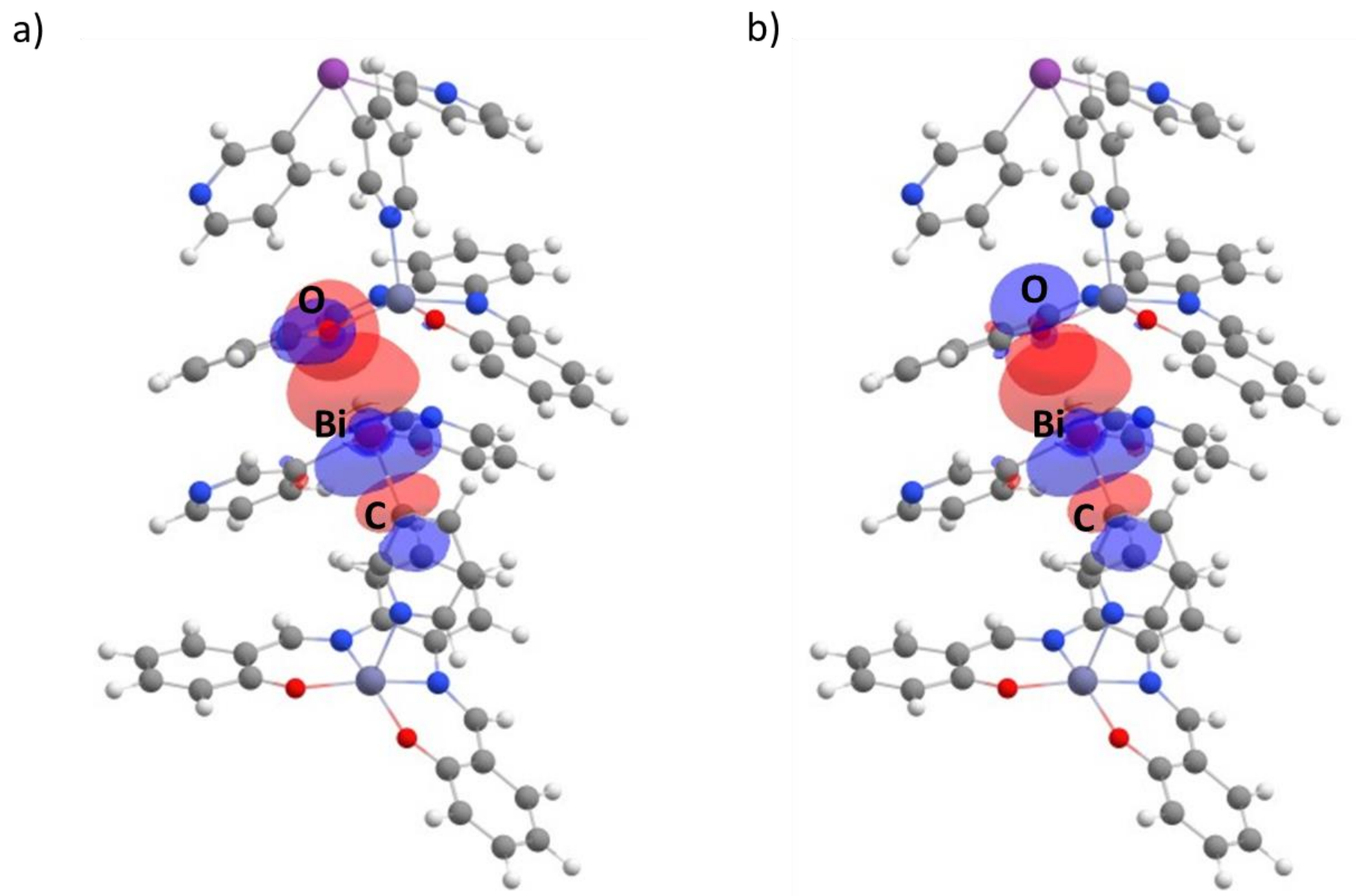


*Figure S66. NBOs with greatest contribution in the interaction Bi–O4. a) Interaction between donor NBO LP(1)O4 to acceptor NBO BD*(1)Bi 71 - C78. b) Interaction between donor NBO LP(3)O4 to acceptor NBO BD*(1)Bi 71 - C78. Only the orbitals between Oxygen and Bismuth with the shortest distance are shown for clarity.*

Calculation of the total attractive interaction energy based on the NBO approach gave values of 4.91 and 7.09 kcal/mol for the pnictogen bonds showing the longest and shortest Bi–O distances, respectively. The latter (shown in Figure S66) is located approximately along the extension of the Bi–$C_{py}$ bond of the Zn-coordinated pyridyl arm, indicating a deepened sigma hole on the pnictogen atom.

## QTAIM calculations

Quantum Theory of Atoms in Molecules (QTAIM) analysis was carried out on **1**·ZnSal dimer and revealed two bond critical point (BCPs) along the Bi - - - O axis, supporting the presence of pnictogen bonds.

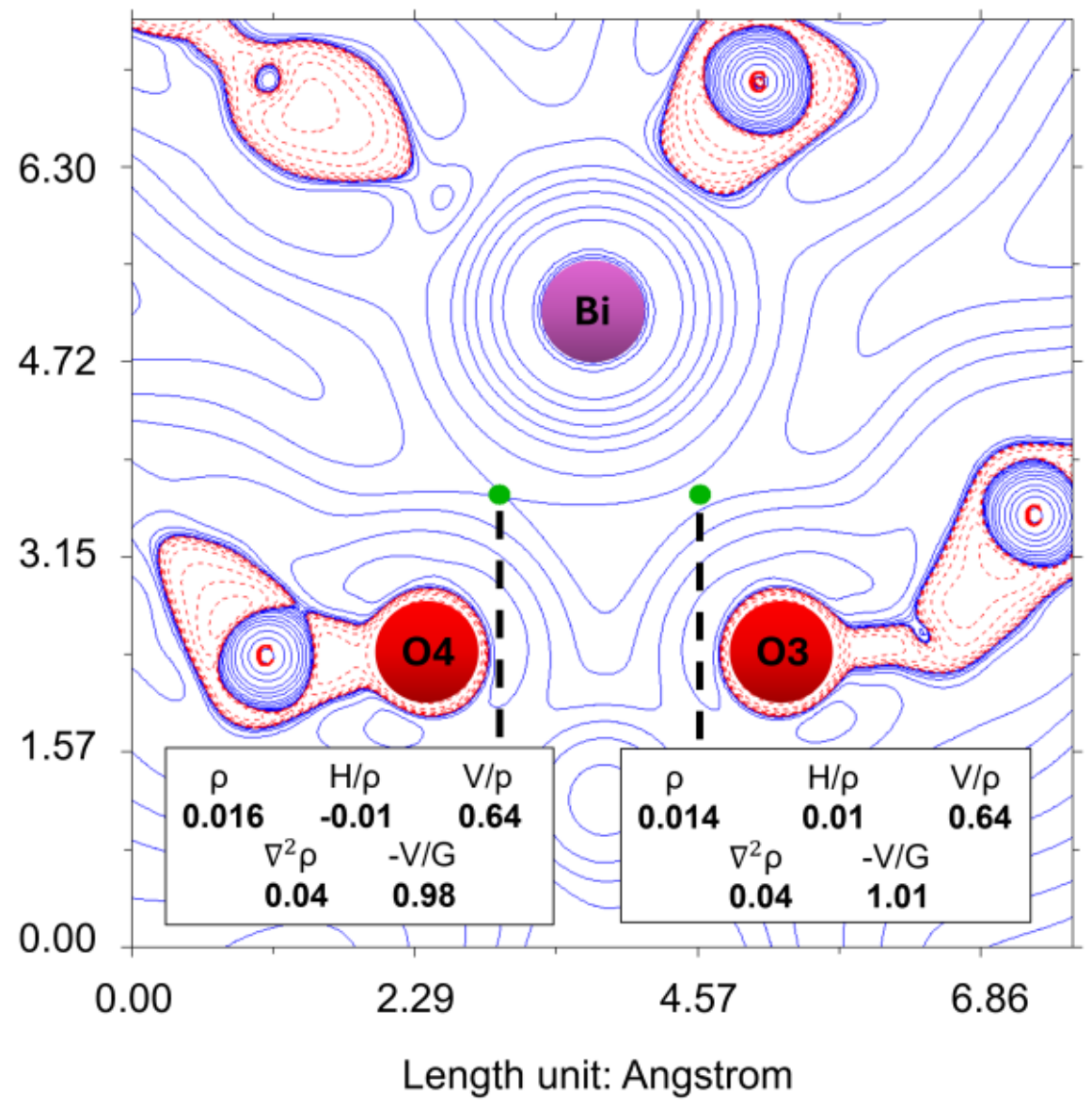


*Figure S67. 2D Quantum Theory of Atoms in Molecules (QTAIM) analysis of **1**·ZnSal. Contour lines show the Laplacian of the electron density ($\nabla^2\rho$) (blue $\nabla^2\rho > 0$; red $\nabla^2\rho < 0$). Atomic critical points (3, +3) are displayed as atom labels. Bond critical points (3, −1) are displayed as green dots. The values of the electron density (ρ), the Laplacian of the electron density ($\nabla^2\rho$), the relative energy density (H/ρ), the relative kinetic energy density (V/ρ) and the ratio of kinetic and potential energy densities (−V/G) at the bond critical point are given in atomic units (a.u.).*

## Effect of coordination on pnictogen bonding

The key local maximum ($V_{max}$) located opposite the Bi–$C_{py}$ bond (sigma hole) in the electrostatic surface potential (ESP) was followed as alternating salphen and Bi(3-py)$_3$ fragments to build up an assembly of three {Bi(3-py)$_3$·ZnSal} monomeric units, as shown in Figure S68.

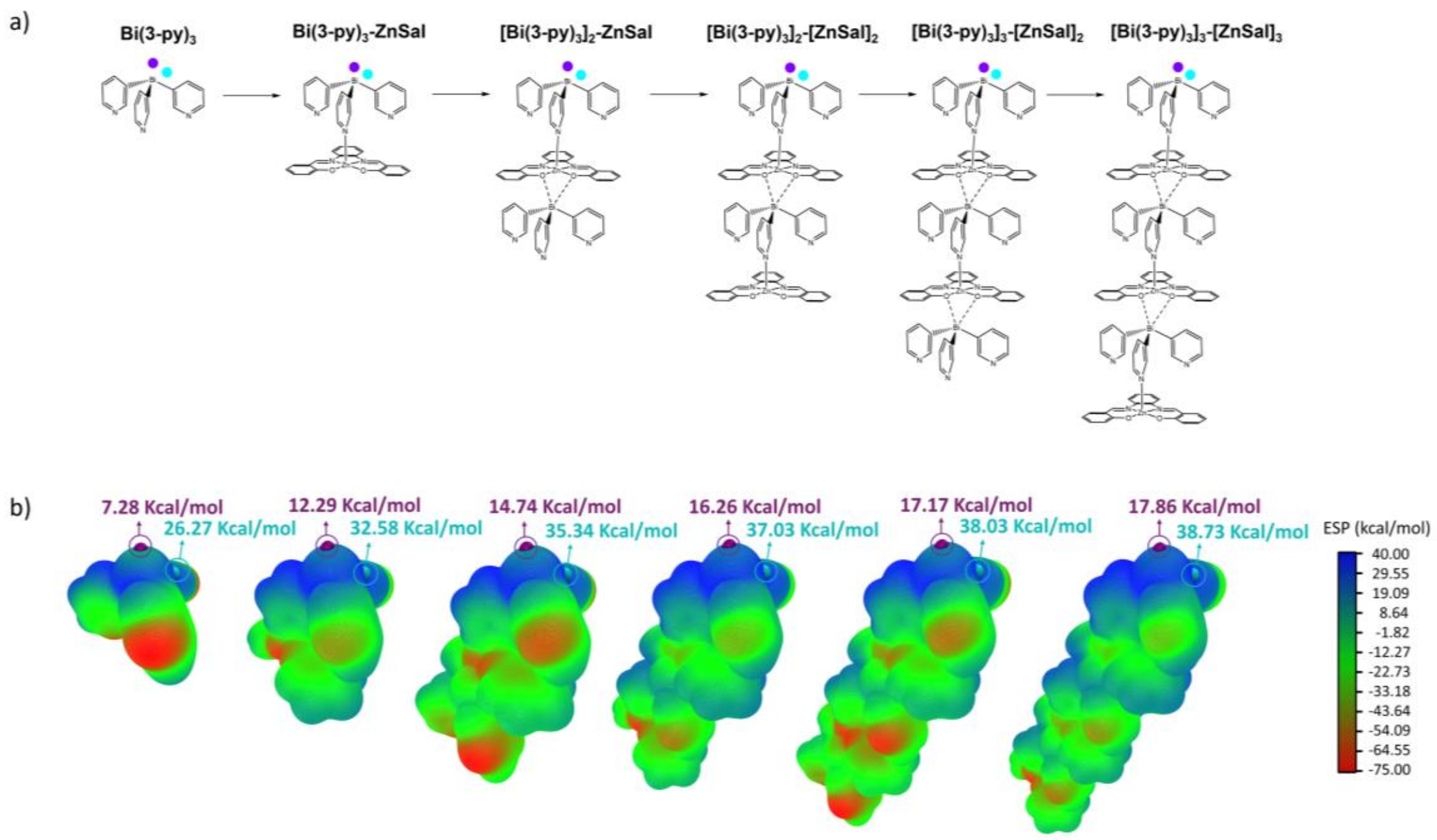


*Figure S68. Schemes (a) and ESP maps (b) of the columnar growth model for the Bi(3-py)$_3$/salphen assembly. The blue dot corresponds to the key local maximum ($V_{max}$), whereas the purple dot corresponds to the key local minimum ($V_{min}$) corresponding to the Bi lone pair.*

The sigma hole is deepened by 6.31 kcal/mol after coordination of the pyridyl arm of **1** to a single Zn-salphen unit, consistent with an increase in Lewis acidity at Bi. Interestingly, the subsequent Bi···O PnB interaction between the ZnSal unit and the next Bi(3-py)$_3$ unit further deepens the Bi sigma hole, albeit modestly (by 2.76 kcal/mol). This effect becomes progressively weaker as the columnar assembly grows, reflecting the increasingly remote influence of each newly added building block, and reaches a plateau. The available data (up to six moieties were modeled) were fitted by nonlinear regression according to the exponential equation 9, which is typically used to describe saturation behaviors. This analysis gave an extrapolated plateau value of 39.09 kcal/mol, corresponding to an overall sigma hole deepening of 12.82 kcal/mol.

$$E = E_0 + (E_{\max} - E_0)(1 - e^{-K \cdot sg}) \qquad eq\ 9$$

Where:
$E_0$ is the $V_{\max}$ for the Bi(3-py)$_3$ ligand.
$E_{max}$ is the energy plateau of the key local maximum.
$K$ is a rate constant (reciprocal of $x$ axis units, dimensionless in this case).
$sg$ is the stepwise growth of the columnar assembly (length in number of building blocks).

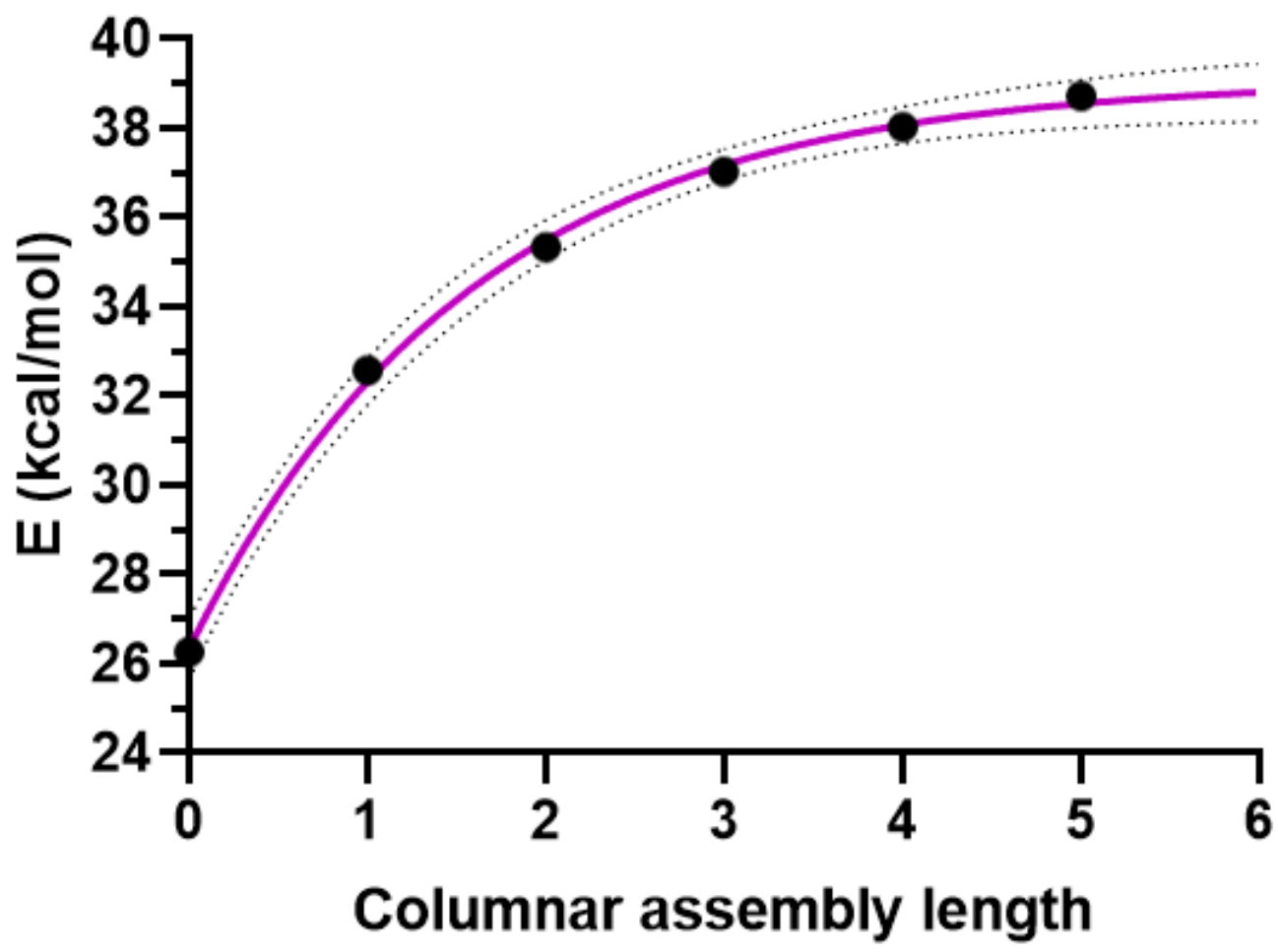


*Figure S69. Key local maximum ($V_{max}$) values corresponding to the observed sigma hole in the Bi(3-py)$_3$/salphen assemblies as a function of the columnar length, i.e., the number of added building blocks (dimensionless), as shown in Figure S68. Purple line: nonlinear regression of the data according to Equation 9. Dotted lines: error bars of the fitting at a 95% confidence interval.*

The same protocol was applied to key local minimum ($V_{\min}$) located atop the Bi atom, which is associated with the charge concentration of its lone pair (see Figure S68). The observed trend was similar to that of $V_{\max}$. Pyridyl coordination of **1** to the first ZnSal unit decreases the charge concentration in this region by 5.01 kcal/mol. Further extension of the columnar assembly leads to additional, but progressively attenuated, charge depletion at the Bi lone-pair region, reaching an extrapolated plateau value of 18.38 kcal/mol (Figure S70), as calculated using the nonlinear regression shown in Equation 9. This suggests increased stereochemical inactivity of Bi lone pair as the columnar assembly grows, and may further contribute to Bi···O pnictogen-bond formation on electrostatic grounds.

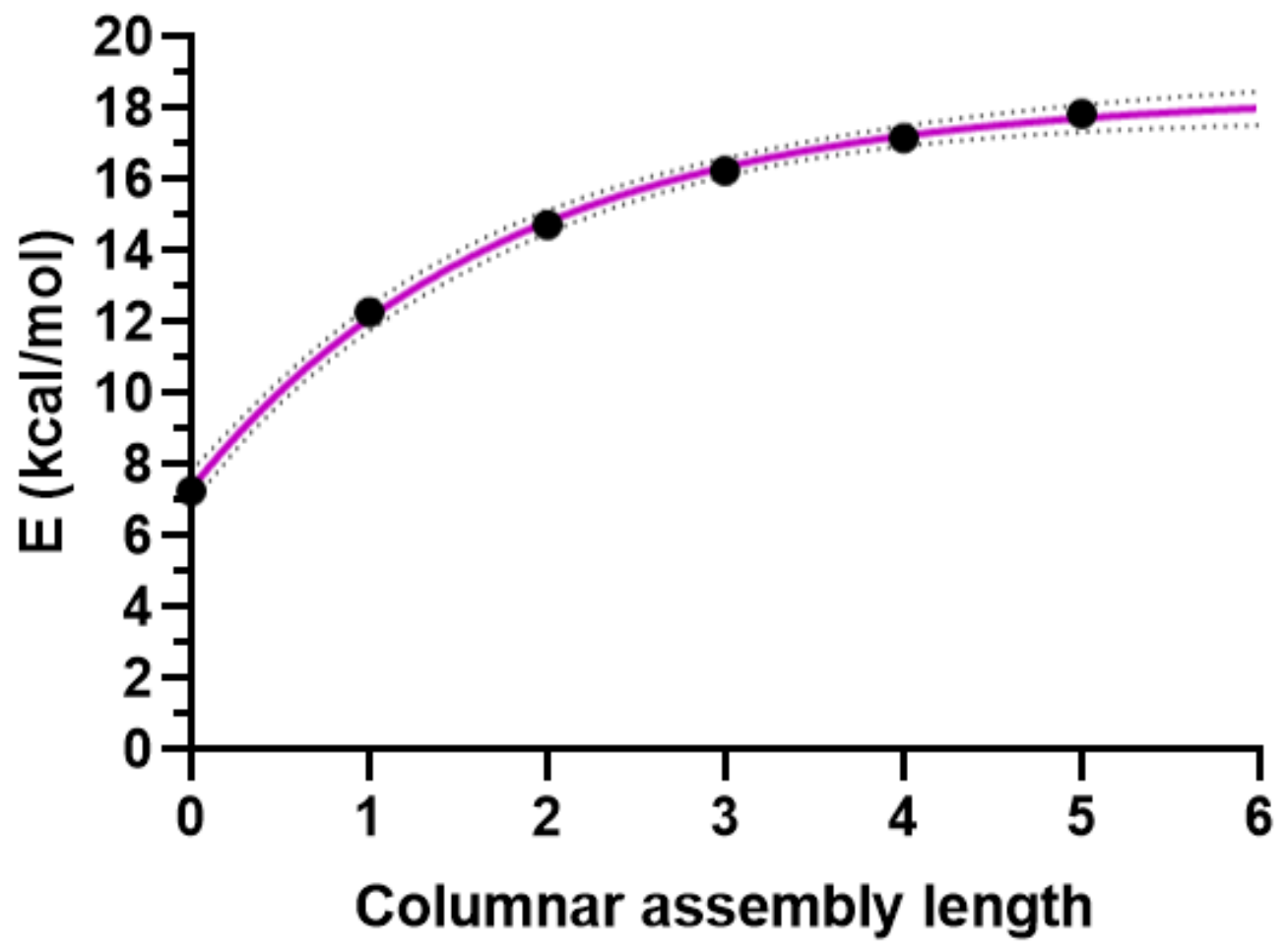


*Figure S70. Values of the key local minimum ($V_{min}$) representing charge concentration attributed to the Bi lone pair in Bi(3-py)$_3$/salphen assemblies as a function of the column length (i.e., number of added building blocks, dimensionless), as shown in Figure S68. Purple line: nonlinear regression of the data using Equation 9. Dotted lines: error bars of the fitting at a 95% confidence interval.*

Additionally, a local minimum ($V_{\text{min}}$) located near the oxo groups of the salphen moiety (ZnSal) was followed during the same modeling of columnar growth as described above. The assembly was constructed in a bottom-up fashion (Figure S71), allowing the electrostatic trend of the studied region to be tracked upon interaction with multiple Bi(3-py)$_3$/salphen moieties.

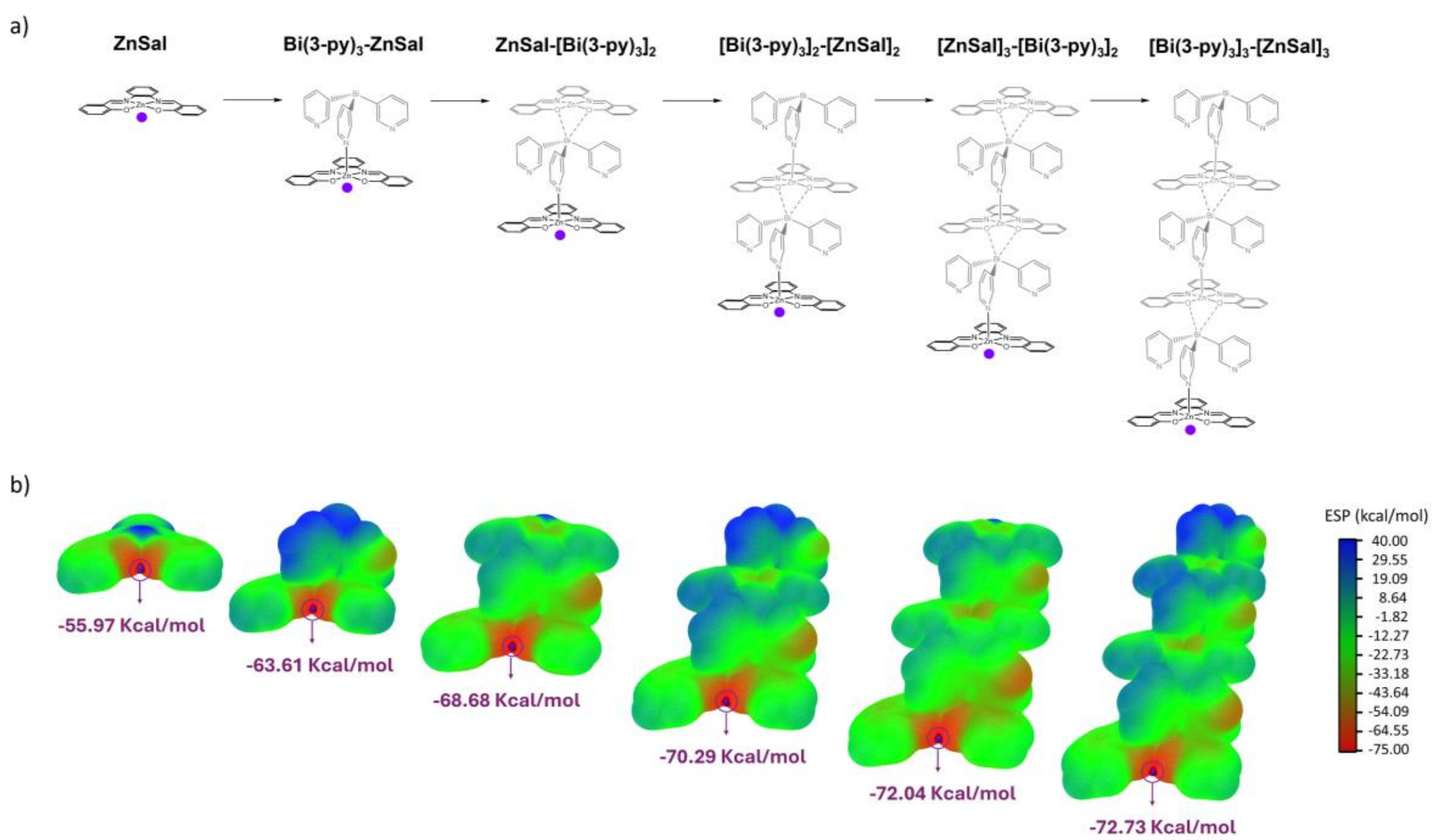


*Figure S71. Schemes (a) and ESP maps (b) of columnar growth model for Bi(3-py)$_3$/salphen assembly. Purple dot corresponds to key local minimum ($V_{min}$) accounting for oxo group donating strength.*

Charge concentration is increased by 7.64 kcal/mol after pyridyl coordination of a single Bi(3-py)$_3$ (**1**) to Zn (II). Interestingly, this charge concentration is reinforced, albeit to a lesser extent (by

2.07 kcal/mol), when a second ZnSal unit interacts with **1** via Bi···O interaction. As expected, the impact of further coordination/interaction events becomes progressively attenuated as the columnar assembly grows due to the increasing distance from the monitored local minimum (Figure S72). This behavior follows a similar trend as observed for the Bi(3-py)$_3$ sigma hole and lone pair (see Figure S69 and Figure S70), reaching a plateau of -73.58 kcal/mol according to the nonlinear fitting.

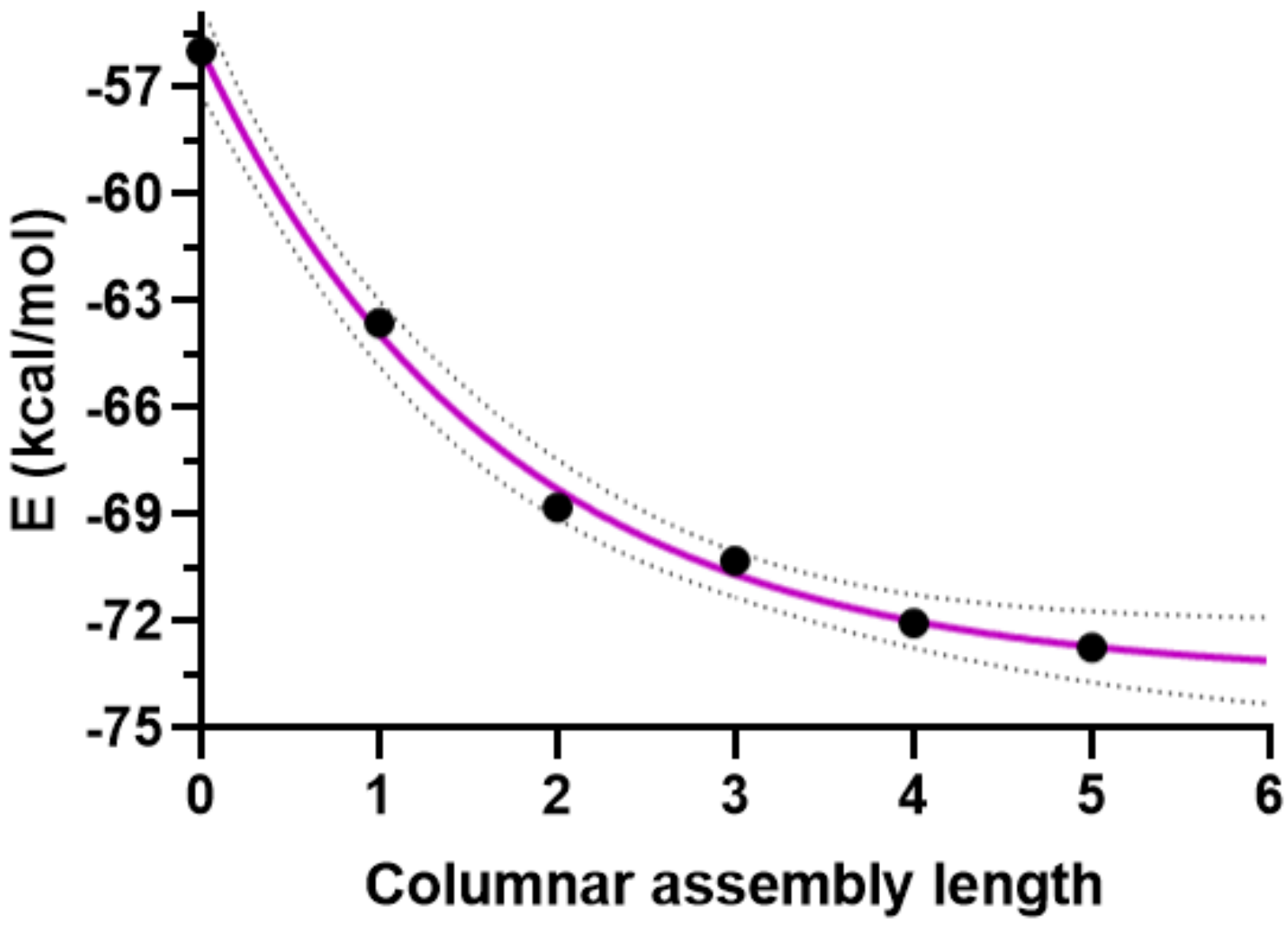


*Figure S72. Values of the key local minimum ($V_{min}$) representing the charge concentration near salphen oxo groups in Bi(3-py)$_3$/salphen assemblies as a function of the columnar length (i.e., number of added building blocks, dimensionless), as shown in Figure S71. Purple line: nonlinear regression of the data according to Equation 9. Dotted lines: error bars of the fitting at a 95% confidence interval.*

# High resolution mass data

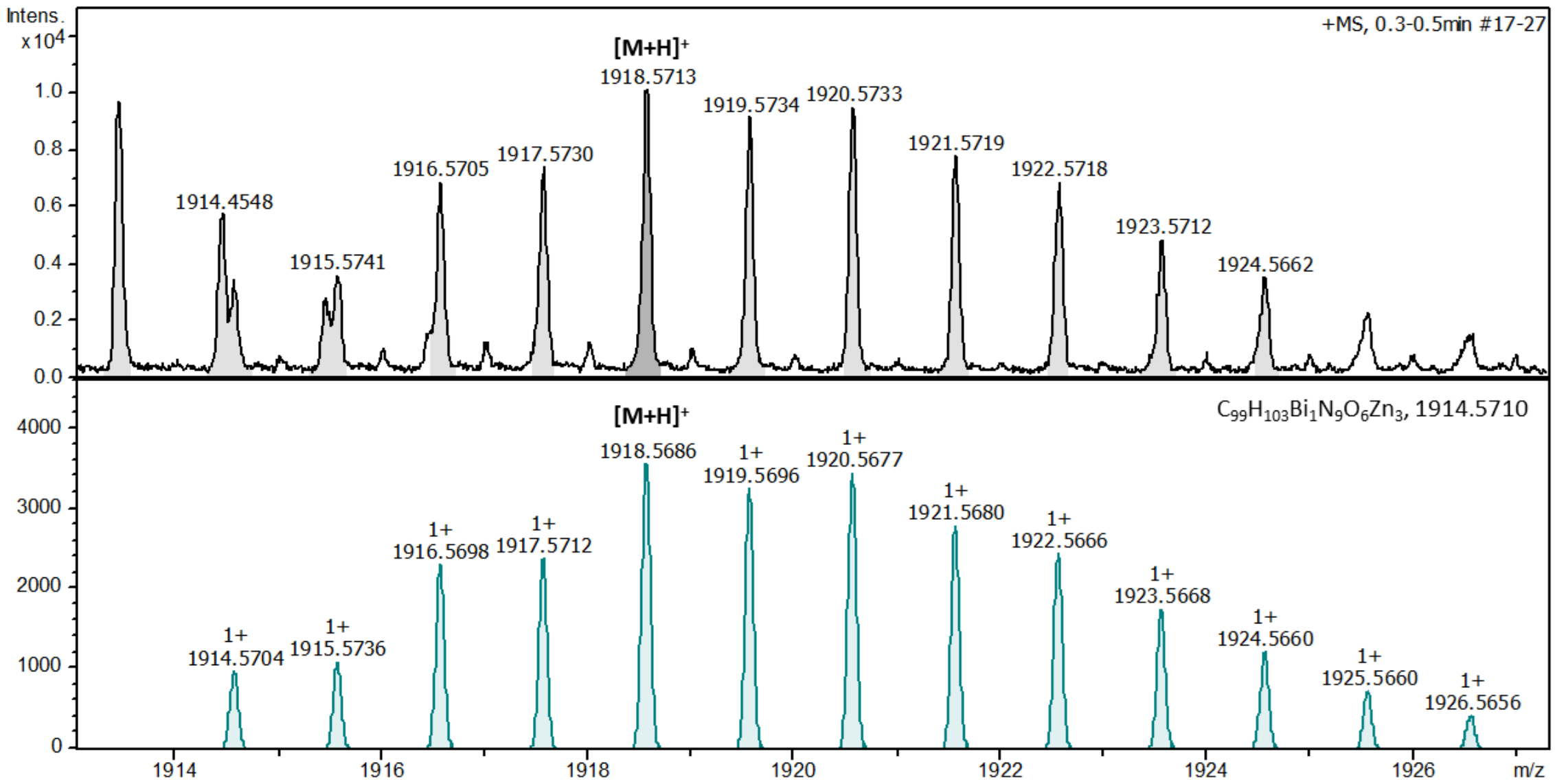


| Meas. m/z | m/z | err [ppm] | Ion Formula |
|---|---|---|---|
| 1918.5713 | 1918.5686 | 0.3 | $C_{99}H_{103}BiN_9O_6Zn_3$ |

*Figure S73. HR-MS (ESI-QTOF) (positive mode) of **1**·ZnSal-ᵗBu showing the expected [M+H]⁺ peak at m/z 1918.5713 (top) and simulation (m/z calculated: 1918.5686) (bottom) (0.3 ppm error).*

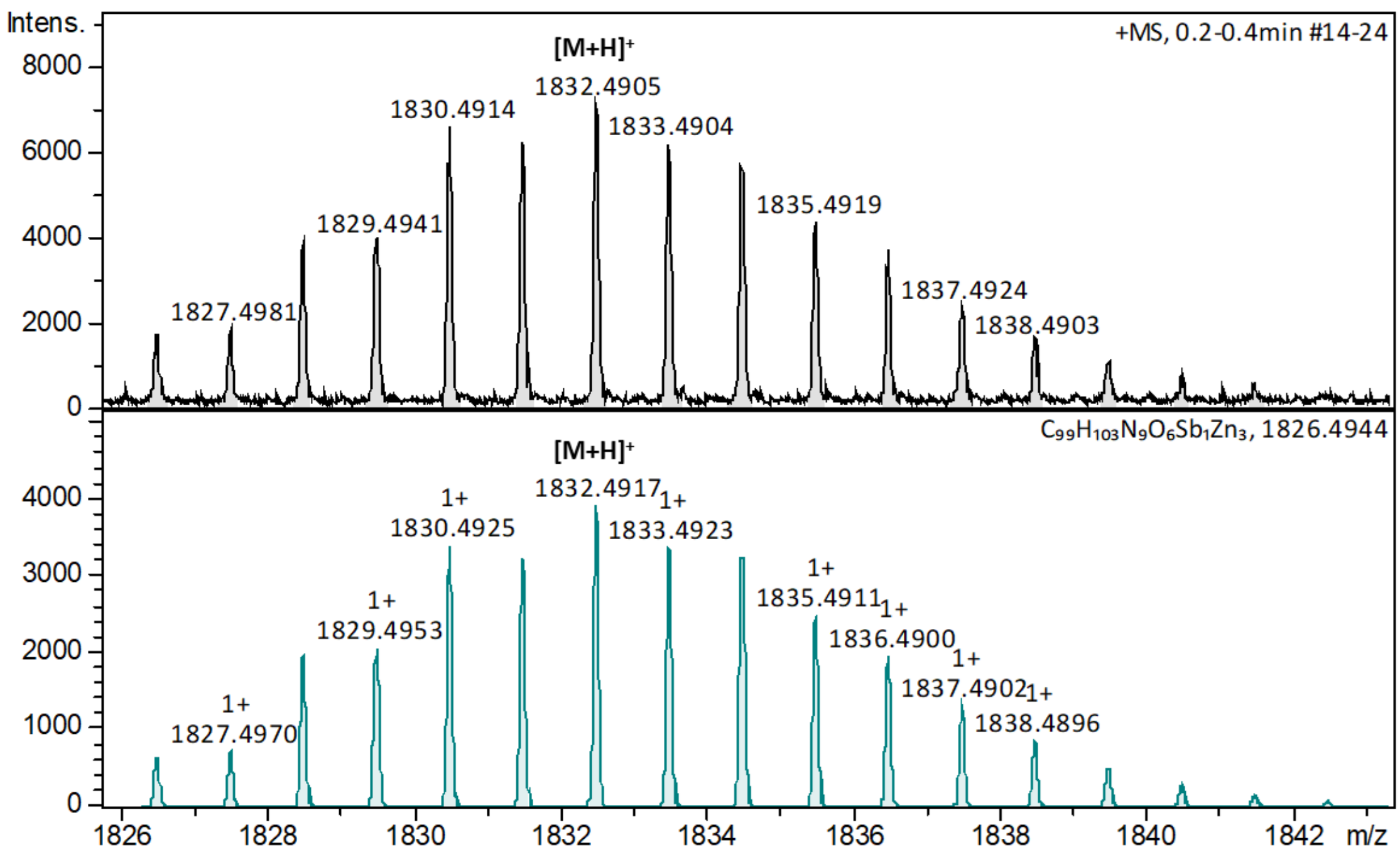


| Meas. m/z | m/z | err [ppm] | Ion Formula |
|---|---|---|---|
| 1832.4905 | 1832.4917 | -1.2 | $C_{99}H_{103}N_9O_6SbZn_3$ |

*Figure S74. HR-MS (ESI-QTOF) (positive mode) of **2**·ZnSal-ᵗBu showing the expected [M+H]⁺ peak at m/z 1832.4905 (top) and simulation (m/z calculated: 1832.4917) (bottom) (-1.2 ppm error).*

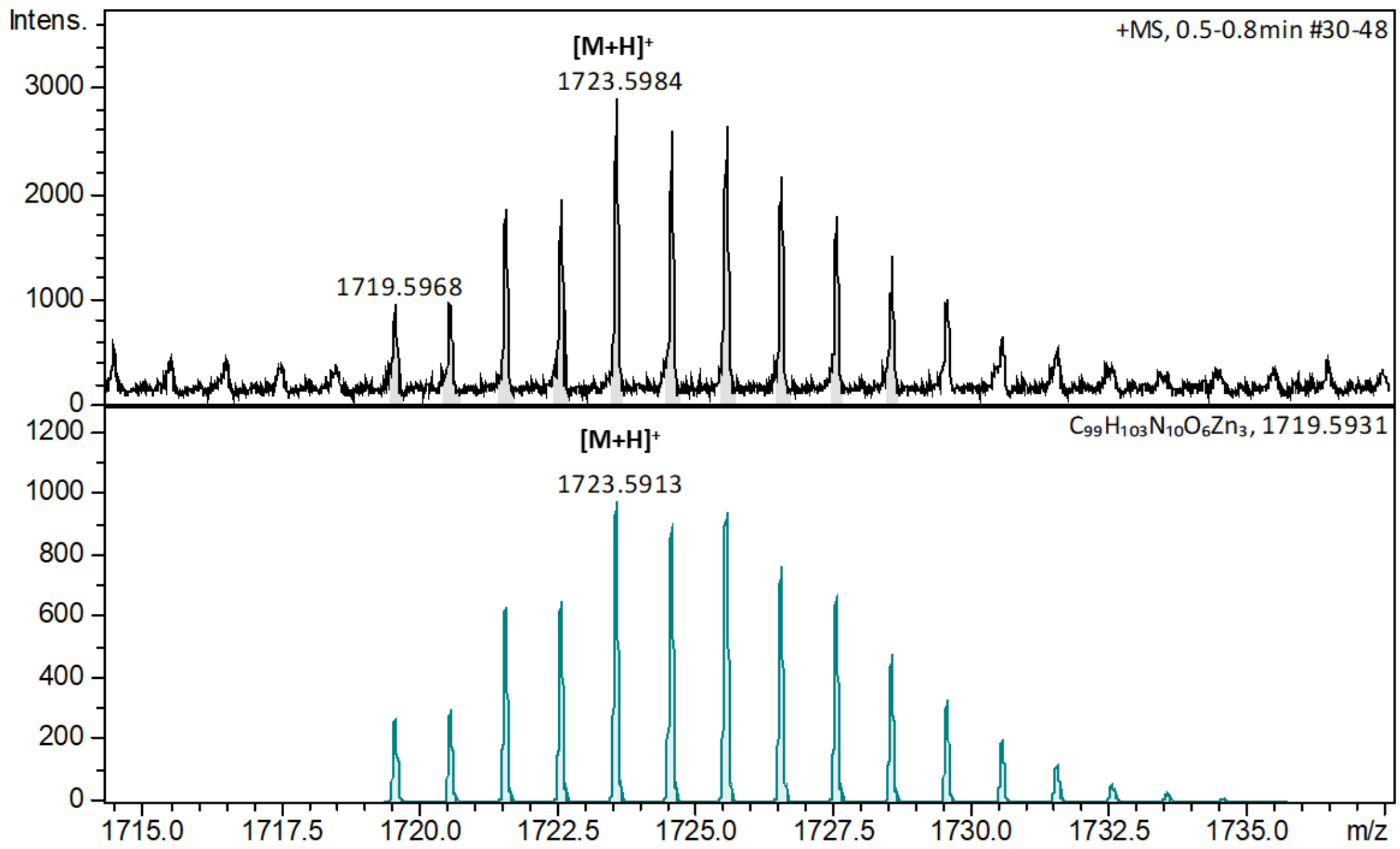


| Meas. m/z | m/z | err [ppm] | Ion Formula |
|---|---|---|---|
| 1723.5984 | 1723.5913 | 7.1 | $C_{99}H_{103}N_{10}O_6Zn_3$ |

*Figure S75. HR-MS (ESI-QTOF) (positive mode) of **3·**ZnSal-ᵗBu showing the expected [M+H]⁺ peak at m/z 1723.5984 (top) and simulation (m/z calculated: 1723.5913) (bottom) (7.1 ppm error).*

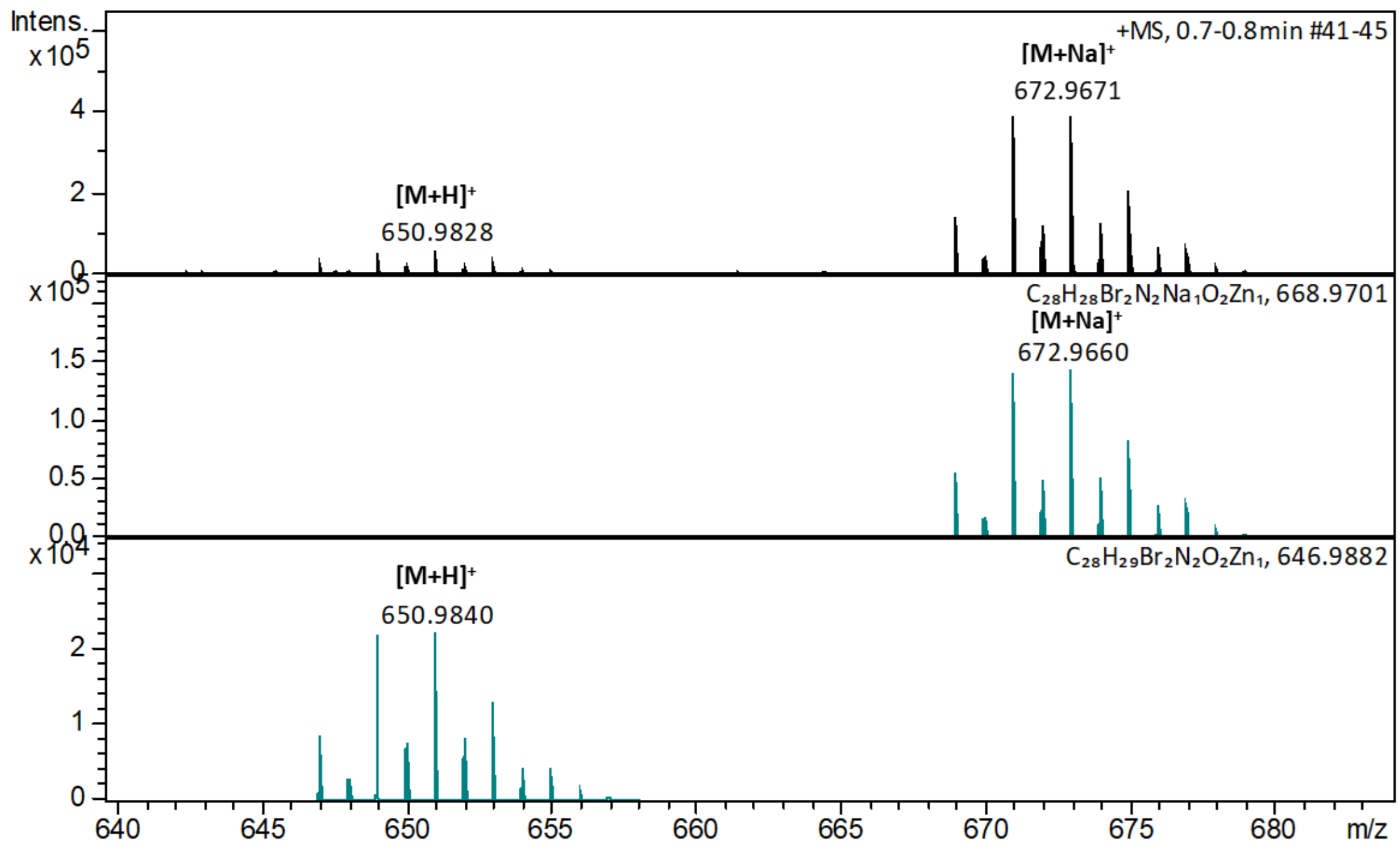


| Meas. m/z | m/z | err [ppm] | Ion Formula |
|---|---|---|---|
| 650.9828 | 650.9840 | -1.2 | $C_{28}H_{29}Br_2N_2O_2Zn$ |
| 672.9671 | 672.9660 | 1.1 | $C_{28}H_{28}Br_2N_2O_2ZnNa$ |

*Figure S76. HR-MS (ESI-QTOF) (positive mode) of **ZnSal-ᵗBu-Br** showing the expected [M+H]⁺ peak at m/z 650.9828 (top) and simulation (m/z calculated: 650.9840) (bottom) (-1.2 ppm error), and also the [M+Na]⁺ peak at m/z 672.9671 (top) and simulation (m/z calculated: 672.9660) (middle) (1.1 ppm error).*

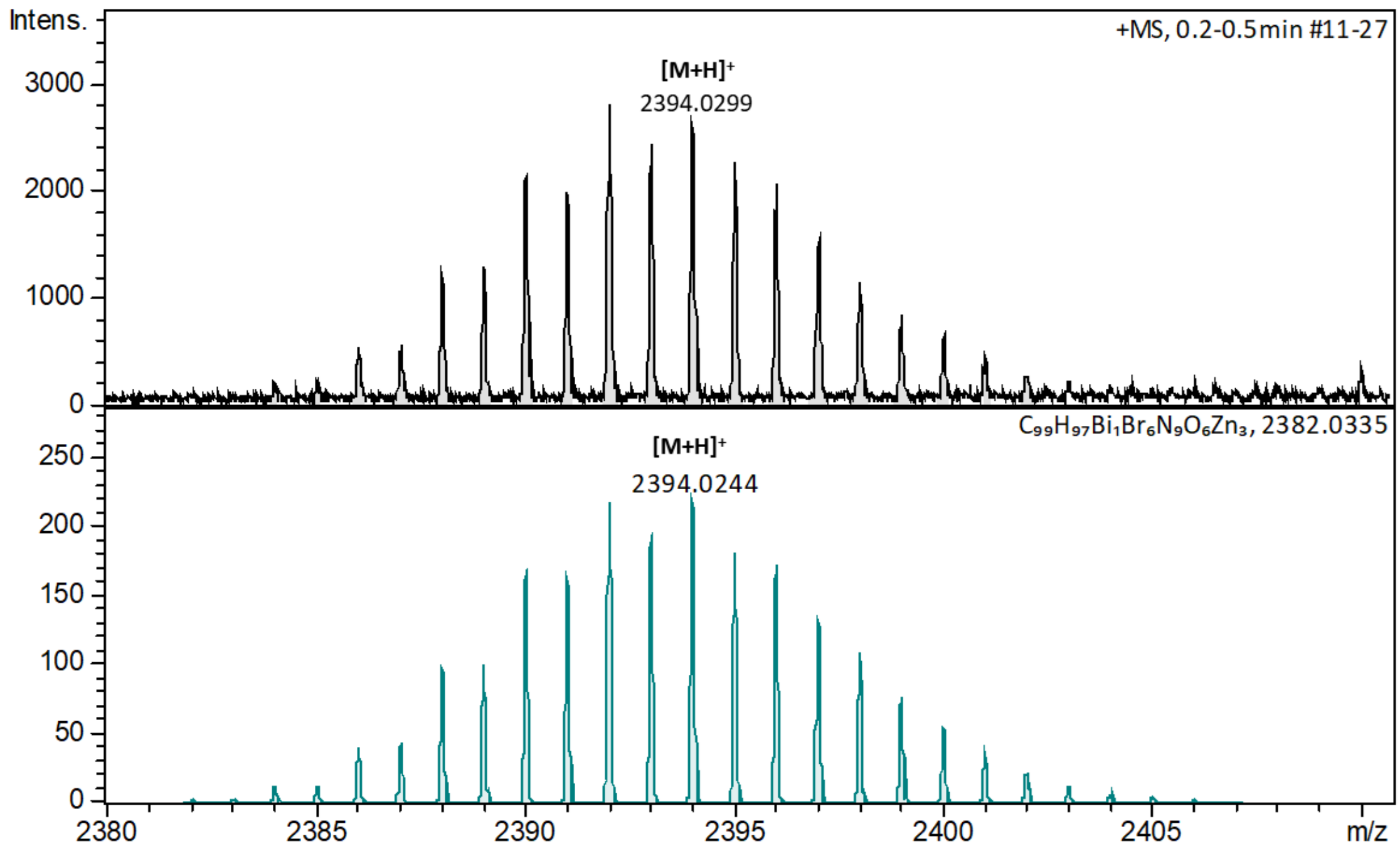


| Meas. m/z | m/z | err [ppm] | Ion Formula |
|---|---|---|---|
| 2394.0299 | 2394.0244 | 5.5 | $C_{99}H_{97}BiBr_6N_9O_6Zn_3$ |

*Figure S77. HR-MS (ESI-QTOF) (positive mode) of **1**·ZnSal-tBu-Br showing the expected [M+H]+ peak at m/z 2394.0299 (top) and simulation (m/z calculated: 2394.0244) (bottom) (5.5 ppm error).*

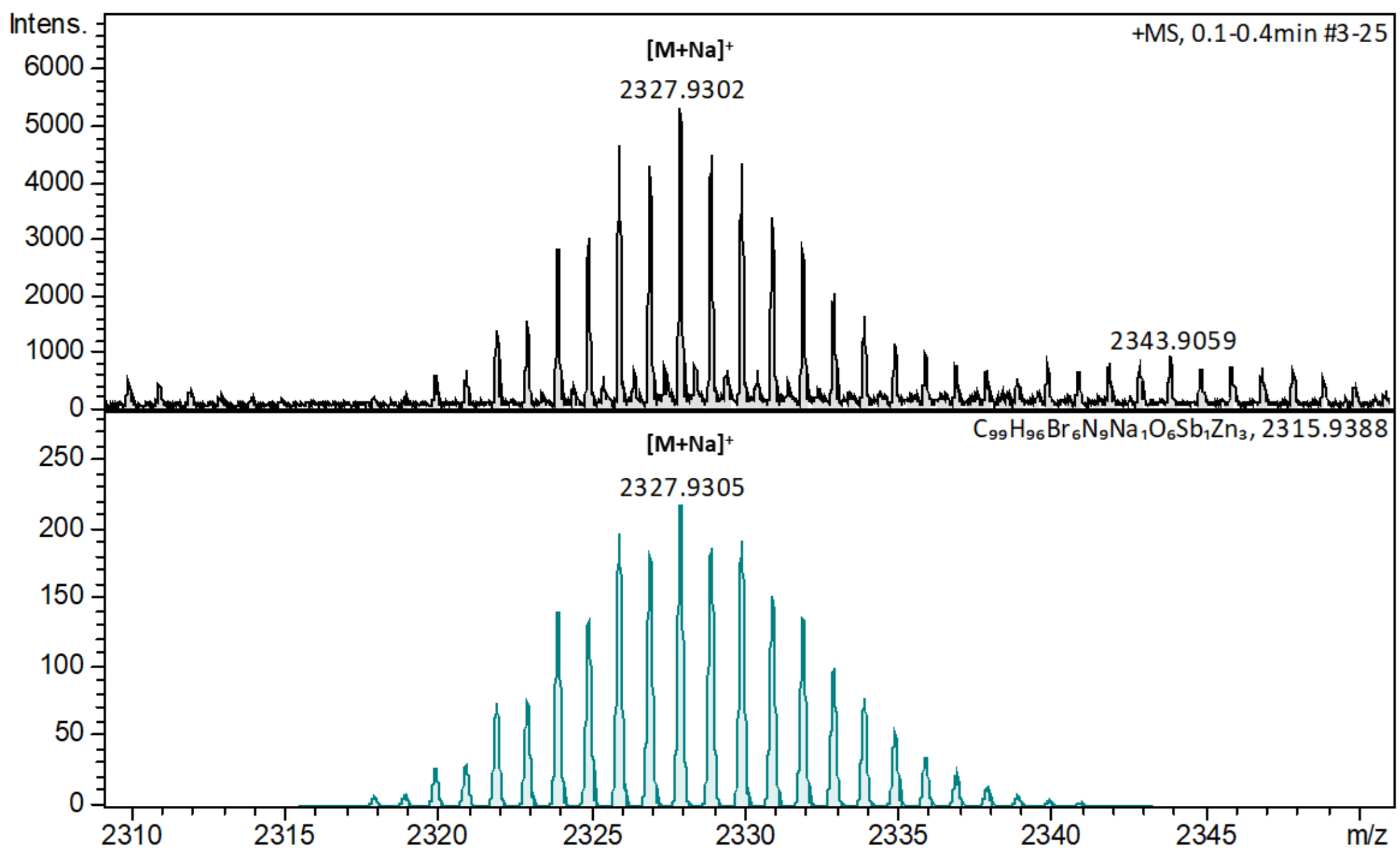


| Meas. m/z | m/z | err [ppm] | Ion Formula |
|---|---|---|---|
| 2327.9302 | 2327.9305 | -0.3 | $C_{99}H_{96}Br_6N_9O_6SbZn_3Na$ |

*Figure S78. HR-MS (ESI-QTOF) (positive mode) of **2**·ZnSal-tBu-Br showing the expected [M+Na]+ peak at m/z 2327.9302 (top) and simulation (m/z calculated: 2327.9305) (bottom) (-0.3 ppm error).*

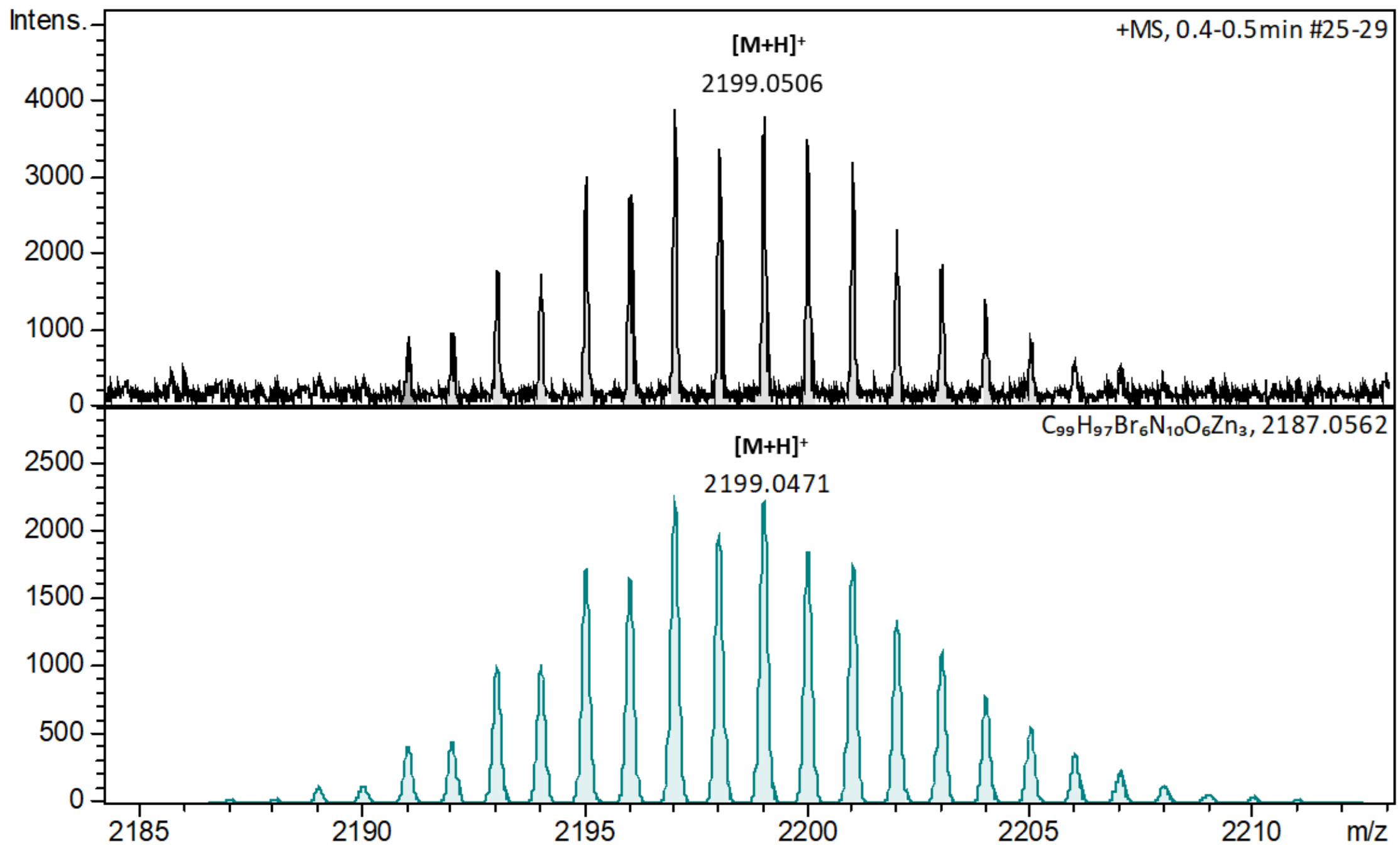


| Meas. m/z | m/z | err [ppm] | Ion Formula |
|---|---|---|---|
| 2199.0506 | 2199.0471 | 3.5 | $C_{99}H_{97}Br_6N_{10}O_6Zn_3$ |

*Figure S79. HR-MS (ESI-QTOF) (positive mode) of **3**·ZnSal-$^t$Bu-Br showing the expected $[M+H]^+$ peak at m/z 2199.0506 (top) and simulation (m/z calculated: 2199.0471) (bottom) (3.1 ppm error).*

# FT-IR selected spectra

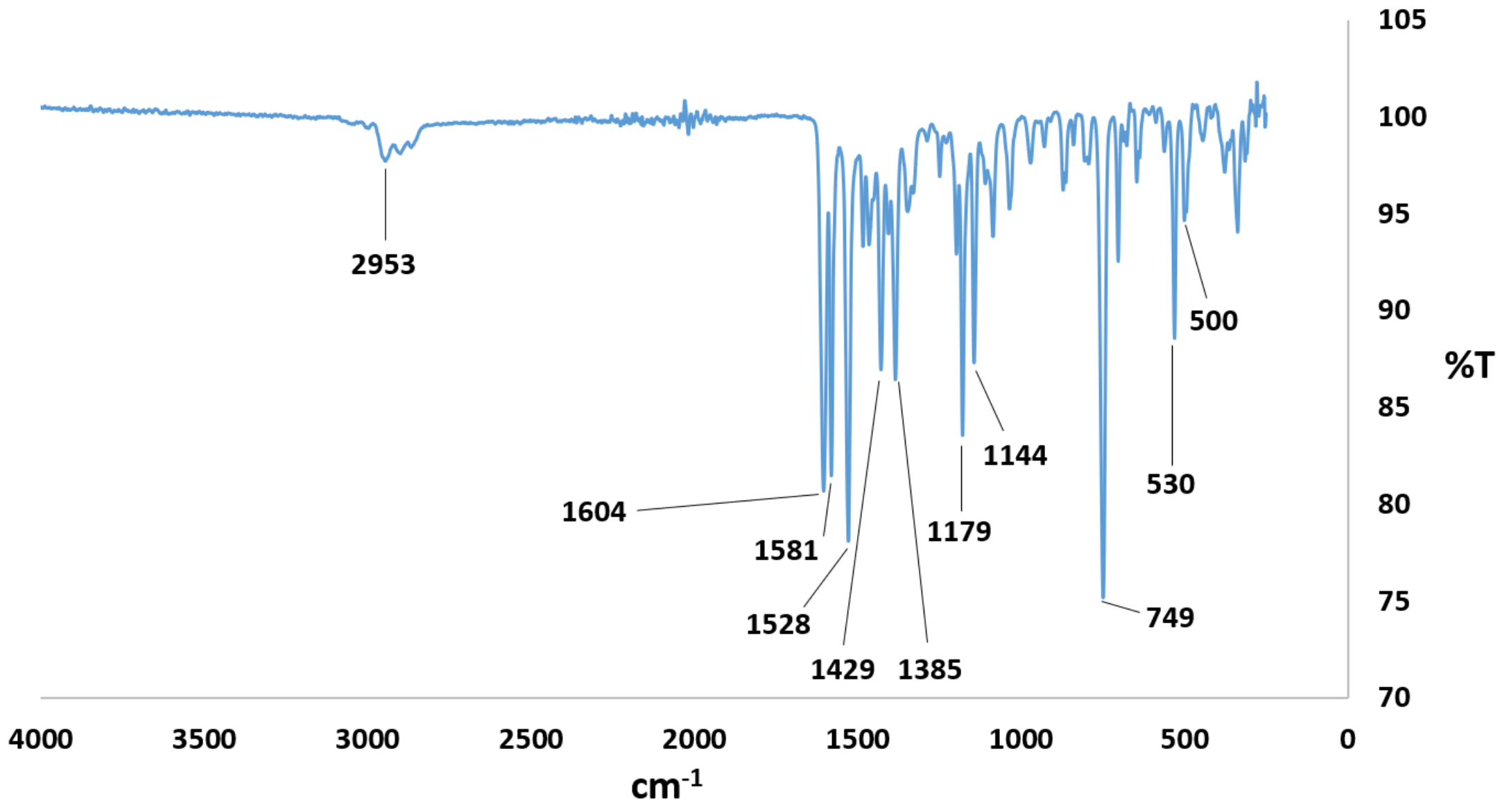


*Figure S80. FT-IR (ATR) spectrum of **1**·ZnSal-$^{t}$Bu.*

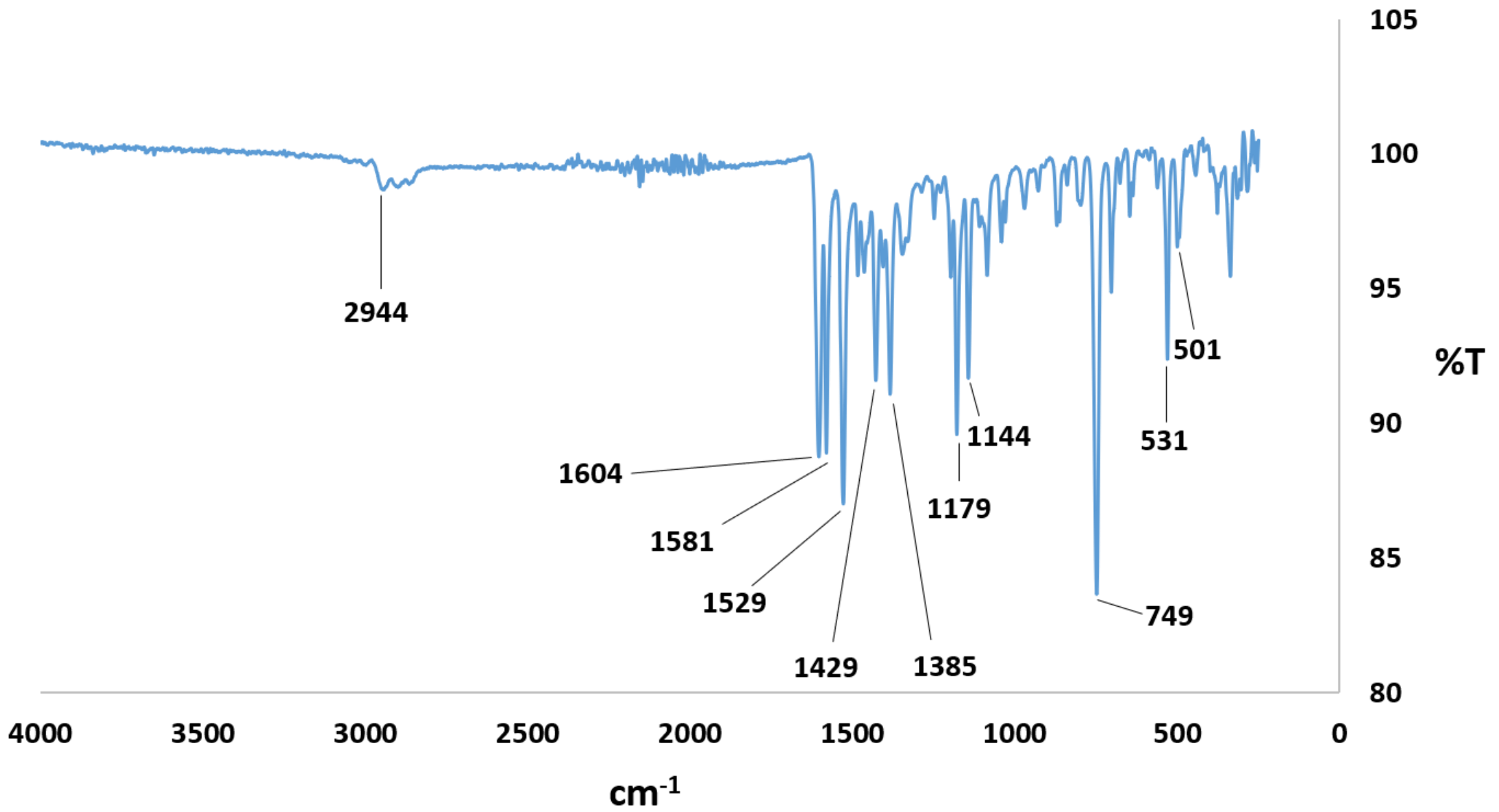


*Figure S81. FT-IR (ATR) spectrum of **2**·ZnSal-$^{t}$Bu.*

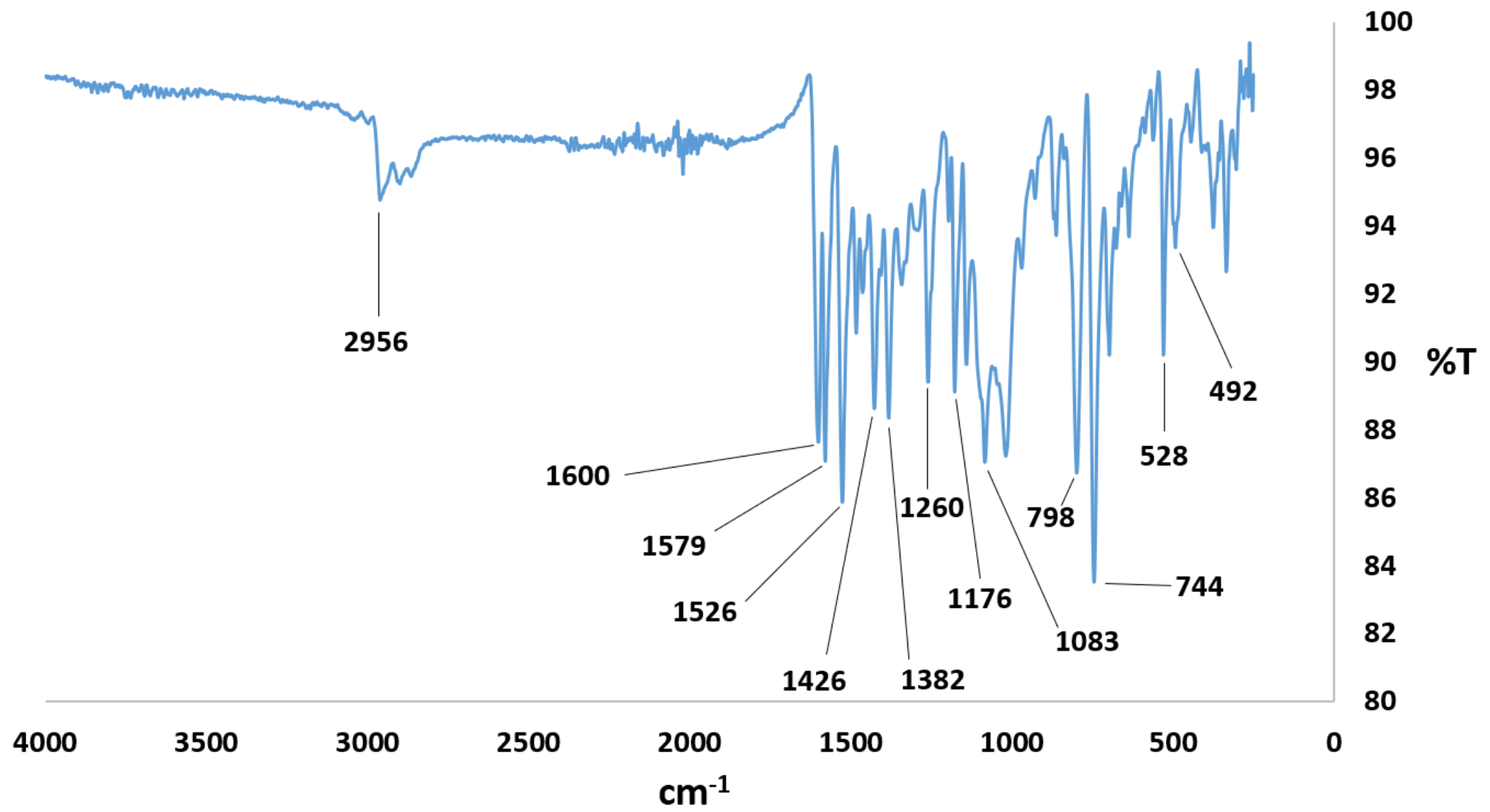


*Figure S82. FT-IR (ATR) spectrum of **3**·ZnSal-$^{t}$Bu.*

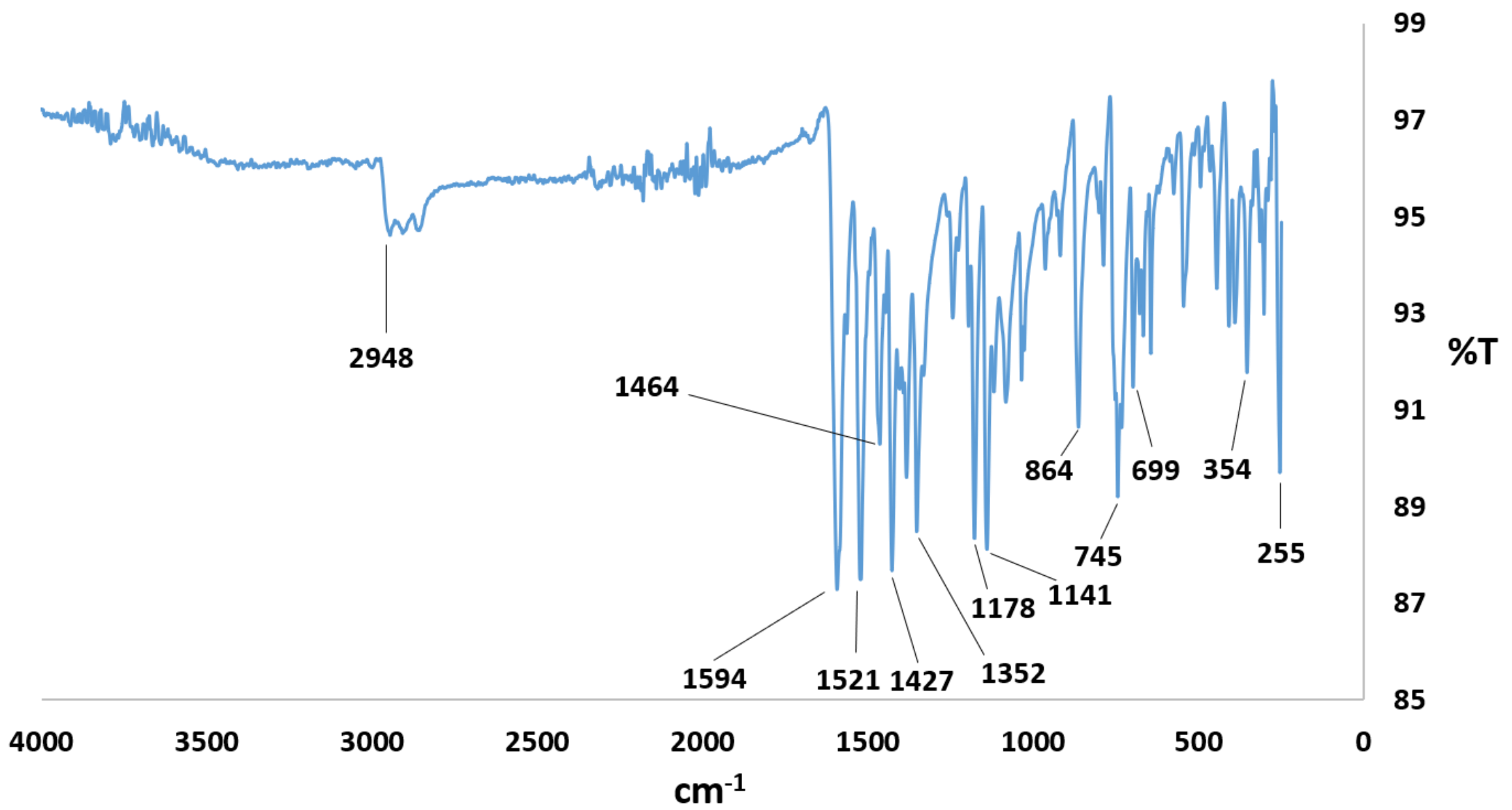


*Figure S83. FT-IR (ATR) spectrum of **1**·ZnSal-$^{t}$Bu-Br.*

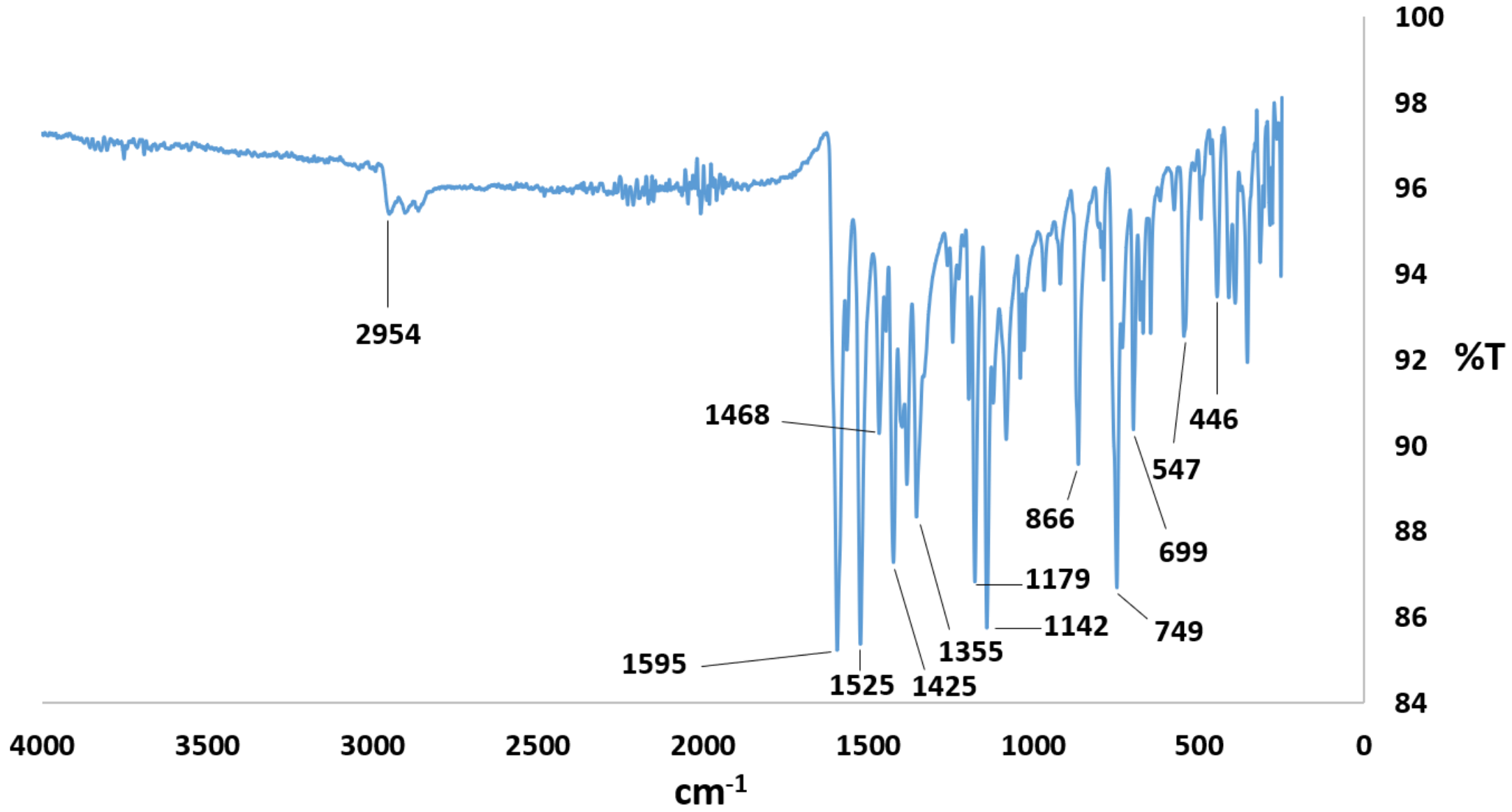


*Figure S84. FT-IR (ATR) spectrum of **2**·ZnSal-$^{t}$Bu-Br.*

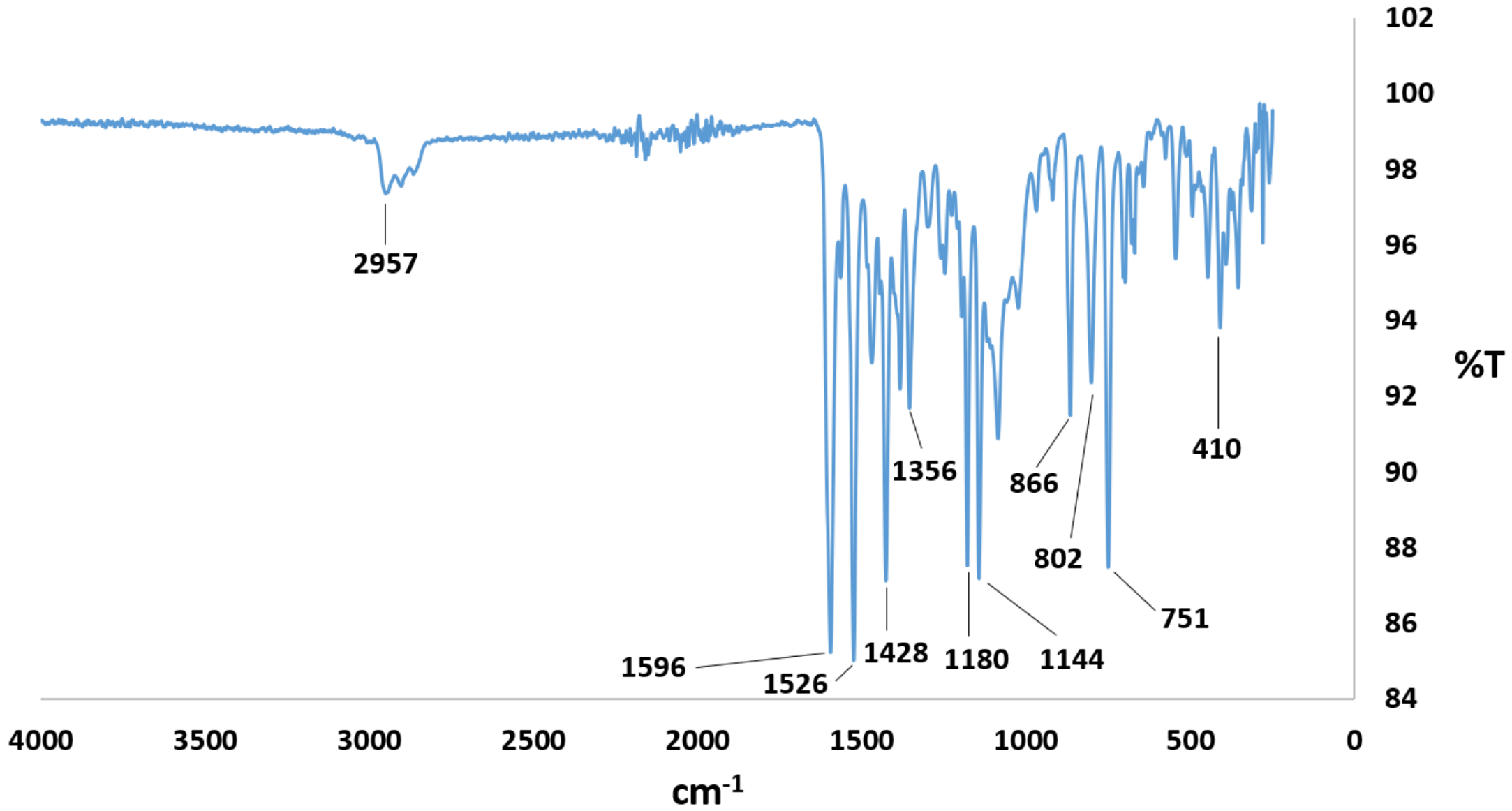


*Figure S85. FT-IR (ATR) spectrum of **3**·ZnSal-$^{t}$Bu-Br.*

# X-Ray crystallographic studies

## Single crystal X-Ray diffraction analysis (SCXRD)

Details of the data collections and structural refinements are given in Table S14.

Further details of the methods of refinement of the structures are as follows.

**1**·ZnSal

The refinement was straightforward.

**1**·ZnSal-$^t$Bu

One molecule of MeOH and three molecules of $CH_2Cl_2$ were present in the asymmetric unit. The C–O distance of the methanol molecule was restrained using DFIX. Two of the $CH_2Cl_2$ were not well resolved and were refined using geometrical restraints together with ADP restraints.

**2**·ZnSal-$^t$Bu

Four molecules of $CH_2Cl_2$ were present in the asymmetric unit. One of these solvent molecules is disordered over two positions and lies close to an inversion centre. Restraints were applied to maintain reasonable anisotropic displacement parameters.

**3**·ZnSal-$^t$Bu

Two molecules of $CH_2Cl_2$ were clearly identified. One of them was refined as a rigid body and thermal parameter restraints (RIGU) were applied. In addition to this, the residual electron density shows poorly resolved solvent molecules, and in some cases, it appears that it could comprise $CH_2Cl_2$ molecules with overlaid hexane molecules (which could suggest that they occupy the site when the not fully occupied $CH_2Cl_2$ is not present). A solvent mask was therefore applied. In the calculated solvent mask, 684 electrons were found in a total volume of 4040 cubic angstroms per unit cell volume. The crystallization solvent was $CH_2Cl_2$/hexane, and this could be consistent with the presence of roughly eight $CH_2Cl_2$ and seven hexane molecules per unit cell, which account for 686 electrons per unit cell. However, the exact quantity of the solvent remains effectively unknown.

**1**·ZnSal-$^t$Bu-Br

The crystals diffracted relatively weakly, and the refinement reported is the best out of numerous crystals examined. The molecular capsule is well resolved, and restraints on the displacement parameters (SIMU and DELU) were applied to two aryl groups of salphen moieties. Conventional refinement identified two molecules of toluene in the asymmetric unit. One was refined using geometrical restraints (DFIX and SADI) and ADP restraints. The second molecule of toluene, however, was very poorly defined with large displacement ellipsoids, and could not be reasonably refined. After identifying these molecules, Q-peaks from additional solvent molecules that were poorly defined were evident in the difference Fourier map. All these poorly

defined molecules were eventually omitted, and the Olex2 solvent mask was applied (*i.e.*, leaving only one toluene molecule in the reported model). A solvent mask was calculated, and 564 electrons were found in a void with a volume of 3978 cubic angstroms. The crystallization solvent was toluene/hexane. This could be consistent with the presence of roughly three toluene molecules per asymmetric unit (*i.e.*, 12 toluene molecules per unit cell), which accounts for 600 electrons per unit cell. Even though the electron count matches very well with the presence of three toluene molecules per asymmetric unit, the exact identity and quantity of the solvent is not completely clear, and a mixture of both solvents (toluene and hexane) could be present.

**2**·ZnSal-$^{t}$Bu-Br

Two $CH_2Cl_2$ molecules were present in the asymmetric unit and were modelled as disordered over two positions using geometrical restraints (DFIX, SADI) and restraints on displacement parameters (ISOR).

**3**·ZnSal-$^{t}$Bu-Br

Conventional refinement identified two molecules of $CHCl_3$ in the asymmetric unit that were well defined. In addition to this, several Q-peaks were observed in the Fourier map from poorly defined solvent. After defining two $CHCl_3$ molecules (which seem evident but not well resolved), additional peaks appeared in the Fourier map suggesting superimposed hexane molecules (i.e., it is possible that these $CHCl_3$ molecules could be partially occupied, and hexane present when $CHCl_3$ is not present). Attempts to refine these additional highly disordered solvent molecules were unsuccessful, and they were therefore omitted from the model and treated using the Olex2 solvent mask. The final reported model thus retains only two explicit $CHCl_3$. A solvent mask was calculated, and 380 electrons were found in a void with a volume of 1699 cubic angstroms. The crystallization solvent was $CHCl_3$/hexane. This could be consistent with the presence of roughly three $CHCl_3$ molecules per asymmetric unit (*i.e.*, six $CHCl_3$ molecules per unit cell), which accounts for 348 electrons per unit cell. These $CHCl_3$ molecules are very poorly defined, and the exact identity and quantity of the solvent is not completely clear (*i.e.*, a mixture of $CHCl_3$ and hexane could be present).

**1**·MgSal-$^{t}$Bu

Four molecules of $CH_2Cl_2$ were identified in the asymmetric unit. Although the molecules were relatively well defined, the use of geometrical restraints (DFIX) and ADP restraints was necessary in some cases to maintain more sensible geometries and ADPs, and one of the $CH_2Cl_2$ molecules was treated as a rigid body for the final refinement cycles. Another of the $CH_2Cl_2$ molecules was disordered and was modelled over two positions. In this case, ISOR restraints were used and anisotropic ADPs were applied to all the disordered atoms, and the disordered $CH_2Cl_2$ molecule was treated as a rigid body for the final refinement cycles.

Additional apparent $CH_2Cl_2$ molecules were also present, along with additional electron density that was difficult to resolve into any meaningful molecular model. Thus, a solvent mask was applied to account for this residual solvent, which may be $CH_2Cl_2$ and/or hexane. A solvent mask was calculated, and 692 electrons were found in a total volume of 3214 cubic angstroms. The

crystallization solvent was $CH_2Cl_2$/hexane, and this could be consistent with the presence of roughly four $CH_2Cl_2$ molecules per asymmetric unit, which accounts for 672 electrons per unit cell.

**2**·MgSal-$^t$Bu

Four molecules of $CH_2Cl_2$ were identified in the asymmetric unit. Although the molecules were relatively well defined, the use of geometrical restraints (DFIX) and ADP restraints was necessary in some cases to maintain more sensible ADPs and geometries. Two apparent $CH_2Cl_2$ molecules and additional electron density that was difficult to resolve into any meaningful molecular model were also present, so a solvent mask was applied to account for residual solvent, which may be $CH_2Cl_2$ and/or hexane. A solvent mask was calculated, and 690 electrons were found in a total volume of 3168 cubic angstroms. The crystallization solvent was $CH_2Cl_2$/hexane, and this could be consistent with the presence of roughly four $CH_2Cl_2$ molecules per asymmetric unit, which accounts for 672 electrons per unit cell.

**3**·MgSal-$^t$Bu

Conventional refinement identified two well-defined molecules of $CH_2Cl_2$ in the asymmetric unit. An additional molecule of $CH_2Cl_2$ was identified, but it was very poorly resolved. Attempts to refine the molecule as disordered over two positions were not satisfactory, so it was omitted and treated using the Olex2 solvent mask. A solvent mask was calculated, and 99 electrons were found in a volume of 636 cubic angstroms. The crystallization solvent was $CH_2Cl_2$/hexane, and this is consistent with the presence of roughly two $CH_2Cl_2$ molecules per unit cell (i.e., one $CH_2Cl_2$ molecule per asymmetric unit), which accounts for 84 electrons per unit cell.

*Table S14. Crystallographic data*

| Identification code | **1**·ZnSal | **1**·ZnSal-$^t$Bu | **2**·ZnSal-$^t$Bu |
|---|---|---|---|
| **CCDC Number** | 2556710 | 2556711 | 2556712 |
| **Empirical formula** | $C_{35}H_{26}BiN_5O_2Zn$ | $C_{103}H_{112}BiCl_6N_9O_7Zn_3$ | $C_{102.5}H_{109}Cl_7N_9O_6SbZn_3$ |
| **Formula weight** | 822.96 | 2205.8 | 2128.99 |
| **Temperature/K** | 293(2) | 293(2) | 180(2) |
| **Crystal system** | triclinic | monoclinic | triclinic |
| **Space group** | P-1 | $P2_1/n$ | P-1 |
| **a/Å** | 9.7758(5) | 16.2801(6) | 13.9632(10) |
| **b/Å** | 12.1579(5) | 35.9585(8) | 16.9128(12) |
| **c/Å** | 15.1011(9) | 18.2422(6) | 23.3931(18) |
| **α/°** | 70.548(4) | 90 | 70.121(7) |
| **β/°** | 72.150(5) | 108.159(4) | 83.156(6) |
| **γ/°** | 68.954(4) | 90 | 74.984(6) |
| **Volume/Å$^3$** | 1543.43(15) | 10147.3(6) | 5014.9(7) |
| **Z** | 2 | 4 | 2 |
| **$\rho_{calc}$g/cm$^3$** | 1.771 | 1.444 | 1.41 |
| **μ/mm$^{-1}$** | 6.51 | 2.645 | 1.219 |
| **F(000)** | 800 | 4496 | 2190 |
| **Crystal size/mm$^3$** | 0.301 × 0.155 × 0.078 | 0.47 × 0.38 × 0.1 | 0.189 × 0.152 × 0.141 |
| **Radiation** | Mo Kα (λ = 0.71073) | Mo Kα (λ = 0.71073) | Mo Kα (λ = 0.71073) |
| **2Θ range for data collection/°** | 5.928 to 55.01 | 3.706 to 50.078 | 3.792 to 50.096 |
| **Index ranges** | -9 ≤ h ≤ 12, -15 ≤ k ≤ 15, -19 ≤ l ≤ 19 | -19 ≤ h ≤ 13, -42 ≤ k ≤ 34, -21 ≤ l ≤ 21 | -16 ≤ h ≤ 14, -18 ≤ k ≤ 20, -25 ≤ l ≤ 27 |
| **Reflections collected** | 13510 | 45732 | 42686 |
| **Independent reflections** | 6844 [$R_{int}$ = 0.0299, $R_{sigma}$ = 0.0504] | 17896 [$R_{int}$ = 0.0444, $R_{sigma}$ = 0.0631] | 17732 [$R_{int}$ = 0.1427, $R_{sigma}$ = 0.2524] |
| **Data/restraints/parameters** | 6844/0/397 | 17896/51/1181 | 17732/37/1186 |
| **Goodness-of-fit on F$^2$** | 1.026 | 1.034 | 0.978 |
| **Final R indexes [I>=2σ (I)]** | $R_1$ = 0.0333, $wR_2$ = 0.0559 | $R_1$ = 0.0497, $wR_2$ = 0.1094 | $R_1$ = 0.0805, $wR_2$ = 0.1611 |
| **Final R indexes [all data]** | $R_1$ = 0.0476, $wR_2$ = 0.0619 | $R_1$ = 0.0839, $wR_2$ = 0.1262 | $R_1$ = 0.2018, $wR_2$ = 0.1990 |
| **Largest diff. peak/hole / e Å$^{-3}$** | 0.86/-0.81 | 0.99/-0.73 | 0.89/-0.72 |
| **Flack parameter** | - | - | - |

| Identification code | **3**·ZnSal-$^t$Bu | **1**·ZnSal-$^t$Bu-Br | **2**·ZnSal-$^t$Bu-Br |
|---|---|---|---|
| **CCDC Number** | 2556713 | 2556714 | 2556715 |
| **Empirical formula** | $C_{100}H_{104}Cl_2N_{10}O_6Zn_3$ | $C_{106}H_{104}BiBr_6N_9O_6Zn_3$ | $C_{101}H_{100}Br_6Cl_4N_9O_6SbZn_3$ |
| **Formula weight** | 1808.94 | 2484.53 | 2475.01 |
| **Temperature/K** | 220(2) | 220(2) | 293(2) |
| **Crystal system** | monoclinic | monoclinic | orthorhombic |
| **Space group** | $P2_1/c$ | $P2_1/c$ | $P2_12_12_1$ |
| **a/Å** | 18.9204(3) | 20.2431(10) | 14.7942(5) |
| **b/Å** | 31.2577(6) | 14.5869(4) | 19.5451(8) |
| **c/Å** | 35.0735(6) | 45.0121(18) | 35.6250(14) |
| **α/°** | 90 | 90 | 90 |
| **β/°** | 96.1015(15) | 102.147(4) | 90 |
| **γ/°** | 90 | 90 | 90 |
| **Volume/Å$^3$** | 20625.2(7) | 12993.8(9) | 10301.1(7) |
| **Z** | 8 | 4 | 4 |
| **$\rho_{calc}$g/cm$^3$** | 1.165 | 1.27 | 1.596 |
| **μ/mm$^{-1}$** | 0.797 | 3.787 | 3.437 |
| **F(000)** | 7568 | 4936 | 4944 |
| **Crystal size/mm$^3$** | 0.647 × 0.275 × 0.207 | 0.695 × 0.151 × 0.133 | 0.453 × 0.146 × 0.125 |
| **Radiation** | Mo Kα (λ = 0.71073) | Mo Kα (λ = 0.71073) | Mo Kα (λ = 0.71073) |
| **2Θ range for data collection/°** | 3.28 to 49.452 | 4.898 to 50.106 | 4.014 to 49.452 |
| **Index ranges** | -22 ≤ h ≤ 18, -36 ≤ k ≤ 29, -36 ≤ l ≤ 41 | -20 ≤ h ≤ 24, -17 ≤ k ≤ 17, -53 ≤ l ≤ 53 | -17 ≤ h ≤ 14, -22 ≤ k ≤ 18, -29 ≤ l ≤ 41 |
| **Reflections collected** | 95635 | 101634 | 32931 |
| **Independent reflections** | 35141 [$R_{int}$ = 0.0524, $R_{sigma}$ = 0.0776] | 22957 [$R_{int}$ = 0.1307, $R_{sigma}$ = 0.1347] | 17225 [$R_{int}$ = 0.0589, $R_{sigma}$ = 0.1201] |
| **Data/restraints/parameters** | 35141/37/2211 | 22957/210/1200 | 17225/87/1245 |
| **Goodness-of-fit on F$^2$** | 1.033 | 1.038 | 1.015 |
| **Final R indexes [I>=2σ (I)]** | $R_1$ = 0.0567, $wR_2$ = 0.1363 | $R_1$ = 0.1135, $wR_2$ = 0.2540 | $R_1$ = 0.0581, $wR_2$ = 0.0783 |
| **Final R indexes [all data]** | $R_1$ = 0.1055, $wR_2$ = 0.1591 | $R_1$ = 0.1622, $wR_2$ = 0.2789 | $R_1$ = 0.1335, $wR_2$ = 0.1010 |
| **Largest diff. peak/hole / e Å$^{-3}$** | 0.73/-0.98 | 1.25/-2.91 | 0.64/-0.35 |
| **Flack parameter** | - | - | -0.008(7) |

| Identification code | **3**·ZnSal-$^t$Bu-Br | **1**·MgSal-$^t$Bu |
|---|---|---|
| CCDC Number | 2556716 | 2556717 |
| Empirical formula | $C_{101}H_{98}Br_6Cl_6N_{10}O_6Zn_3$ | $C_{378}H_{388}Bi_6Cl_{16}Mg_{10}N_{38}O_{20}$ |
| Formula weight | 2436.16 | 7847.42 |
| Temperature/K | 220(2) | 220(2) |
| Crystal system | triclinic | monoclinic |
| Space group | P-1 | $P2_1/n$ |
| a/Å | 14.0400(3) | 19.0039(4) |
| b/Å | 20.8932(8) | 26.9834(4) |
| c/Å | 23.5126(6) | 39.7533(9) |
| α/° | 107.339(3) | 90 |
| β/° | 102.666(2) | 101.181(2) |
| γ/° | 93.372(2) | 90 |
| Volume/Å$^3$ | 6366.2(3) | 19998.2(7) |
| Z | 2 | 2 |
| $\rho_{calc}$g/cm$^3$ | 1.271 | 1.303 |
| μ/mm$^{-1}$ | 2.616 | 2.813 |
| F(000) | 2448 | 7944 |
| Crystal size/mm$^3$ | 0.73 × 0.627 × 0.382 | 0.382 × 0.237 × 0.19 |
| Radiation | Mo Kα (λ = 0.71073) | Mo Kα (λ = 0.71073) |
| 2Θ range for data collection/° | 3.844 to 50.314 | 3.478 to 50.07 |
| Index ranges | -16 ≤ h ≤ 16, -17 ≤ k ≤ 24, -28 ≤ l ≤ 27 | -22 ≤ h ≤ 19, -32 ≤ k ≤ 27, -47 ≤ l ≤ 41 |
| Reflections collected | 55216 | 83225 |
| Independent reflections | 22505 [$R_{int}$ = 0.0453, $R_{sigma}$ = 0.0710] | 35317 [$R_{int}$ = 0.0490, $R_{sigma}$ = 0.0813] |
| Data/restraints/parameters | 22505/0/1207 | 35317/44/2155 |
| Goodness-of-fit on F$^2$ | 1.032 | 1.019 |
| Final R indexes [I>=2σ (I)] | $R_1$ = 0.0511, $wR_2$ = 0.1029 | $R_1$ = 0.0500, $wR_2$ = 0.1061 |
| Final R indexes [all data] | $R_1$ = 0.0850, $wR_2$ = 0.1165 | $R_1$ = 0.0918, $wR_2$ = 0.1225 |
| Largest diff. peak/hole / e Å$^{-3}$ | 0.94/-0.92 | 1.95/-2.01 |
| Flack parameter | - | - |

| Identification code | **2**·MgSal-$^t$Bu | **3**·MgSal-$^t$Bu |
|---|---|---|
| **CCDC Number** | 2556718 | 2556719 |
| **Empirical formula** | $C_{378}H_{388}Cl_{16}Mg_{10}N_{38}O_{20}Sb_6$ | $C_{232}H_{236}Cl_8Mg_6N_{28}O_{12}$ |
| **Formula weight** | 7324.04 | 4037.93 |
| **Temperature/K** | 220(2) | 220(2) |
| **Crystal system** | monoclinic | triclinic |
| **Space group** | $P2_1/n$ | P-1 |
| **a/Å** | 18.9632(4) | 16.9084(5) |
| **b/Å** | 27.0554(5) | 19.1709(6) |
| **c/Å** | 39.6694(7) | 20.3234(7) |
| **α/°** | 90 | 106.088(3) |
| **β/°** | 101.0734(18) | 91.457(3) |
| **γ/°** | 90 | 114.490(3) |
| **Volume/Å$^3$** | 19973.7(7) | 5685.7(3) |
| **Z** | 2 | 1 |
| **$\rho_{calc}$g/cm$^3$** | 1.218 | 1.179 |
| **μ/mm$^{-1}$** | 0.583 | 0.179 |
| **F(000)** | 7560 | 2128 |
| **Crystal size/mm$^3$** | 0.532 × 0.343 × 0.221 | 0.382 × 0.288 × 0.151 |
| **Radiation** | Mo Kα (λ = 0.71073) | Mo Kα (λ = 0.71073) |
| **2Θ range for data collection/°** | 3.188 to 50.444 | 4.164 to 50.11 |
| **Index ranges** | -22 ≤ h ≤ 22, -32 ≤ k ≤ 27, -39 ≤ l ≤ 47 | -20 ≤ h ≤ 19, -20 ≤ k ≤ 22, -24 ≤ l ≤ 22 |
| **Reflections collected** | 92418 | 44764 |
| **Independent reflections** | 35970 [$R_{int}$ = 0.0359, $R_{sigma}$ = 0.0516] | 20078 [$R_{int}$ = 0.0522, $R_{sigma}$ = 0.1003] |
| **Data/restraints/parameters** | 35970/32/2134 | 20078/25/1306 |
| **Goodness-of-fit on F$^2$** | 1.016 | 1.021 |
| **Final R indexes [I>=2σ (I)]** | $R_1$ = 0.0540, $wR_2$ = 0.1358 | $R_1$ = 0.0739, $wR_2$ = 0.1778 |
| **Final R indexes [all data]** | $R_1$ = 0.0801, $wR_2$ = 0.1518 | $R_1$ = 0.1468, $wR_2$ = 0.2247 |
| **Largest diff. peak/hole / e Å$^{-3}$** | 1.17/-1.11 | 0.67/-0.62 |
| **Flack parameter** | - | - |

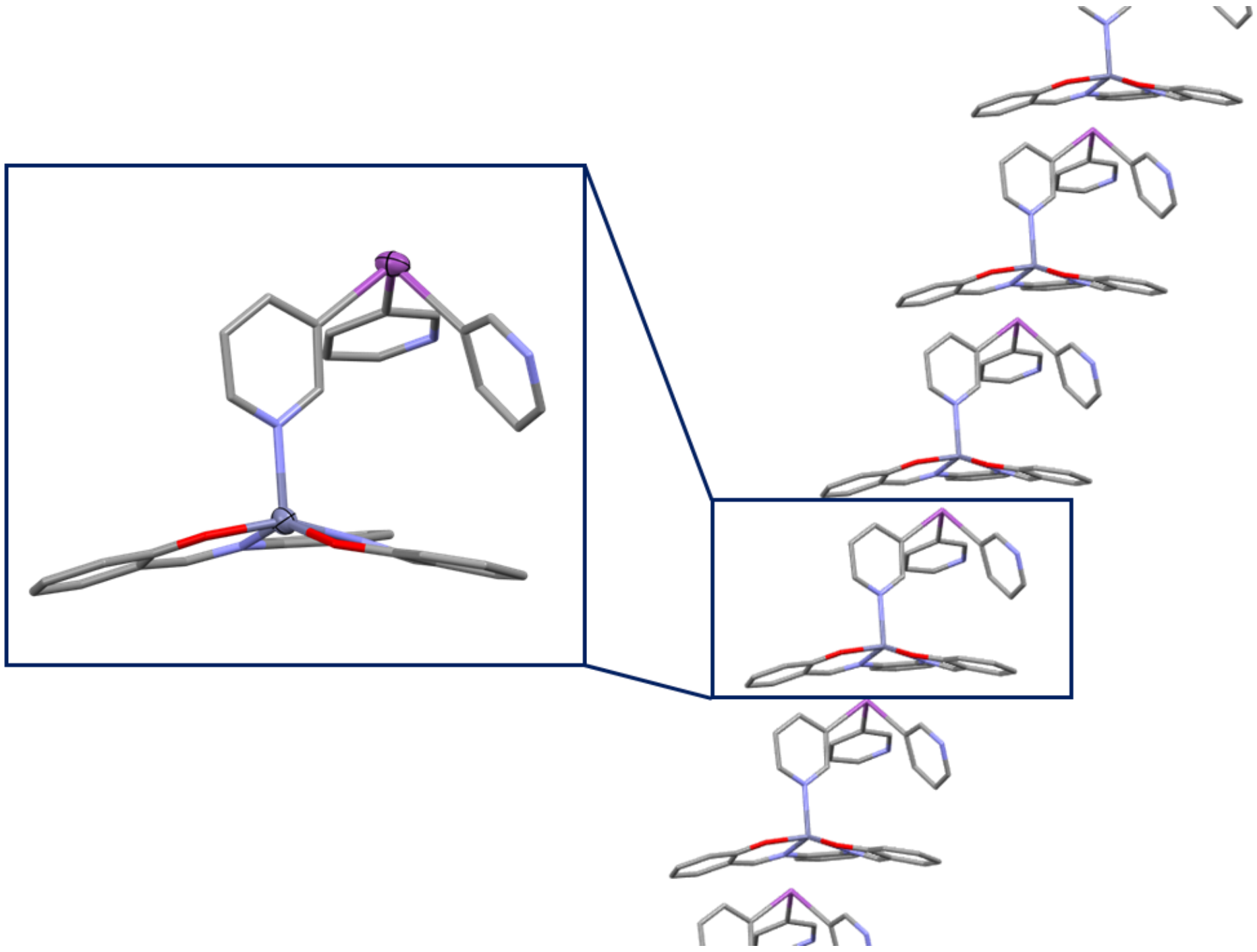

*Figure S86. X-ray structure of the polymer **1·**ZnSal formed through Bi---O interactions with the highlighted {Bi(3-py)$_3$·(ZnSal)} unit. Displacement ellipsoids at 50% probability. H atoms are omitted for clarity. Colour key: C (grey), N (lilac), O (red), Bi (dark purple) and Zn (greyish blue).*

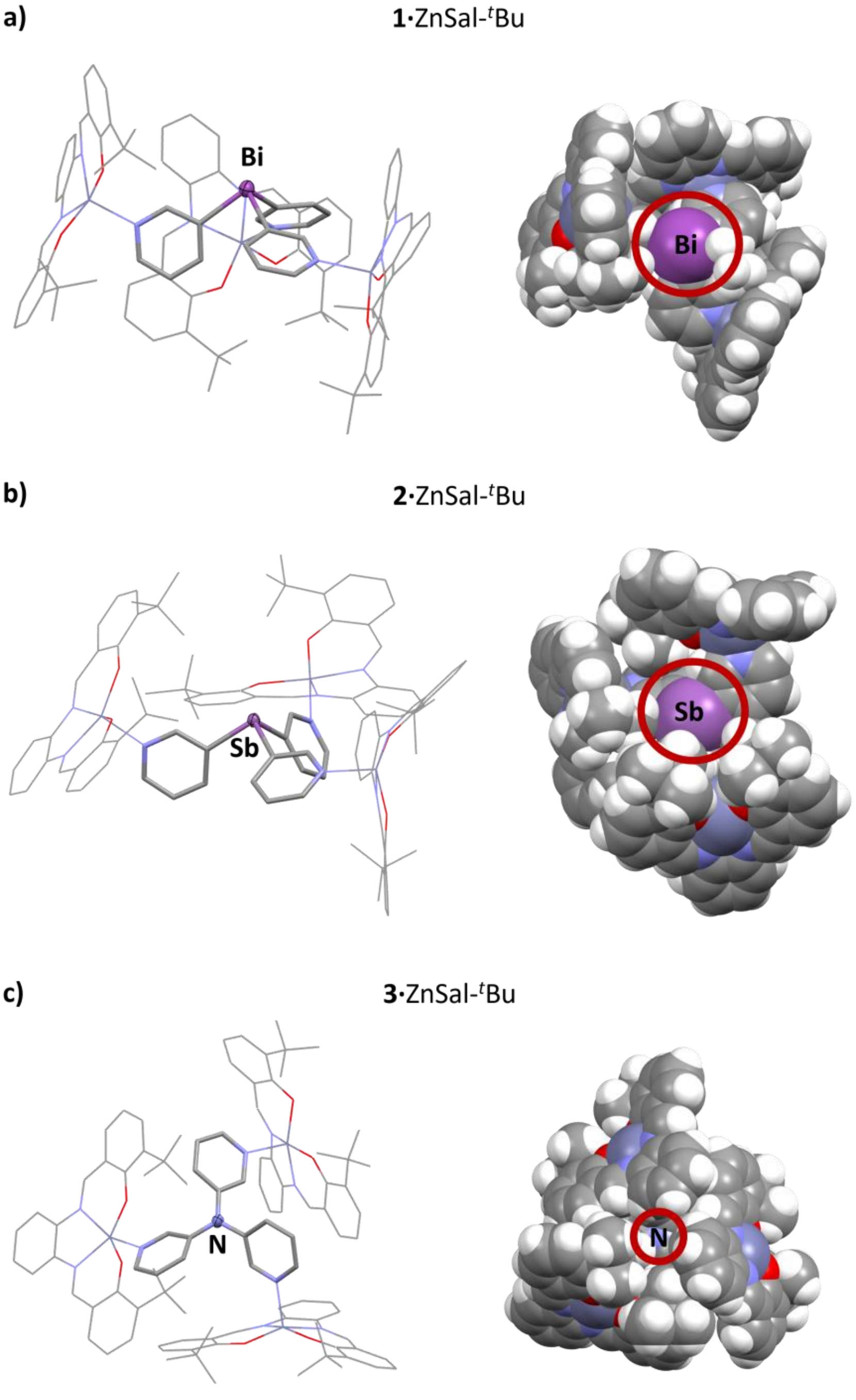


*Figure S87. X-ray structures of the family of molecular capsules **1**·ZnSal-ᵗBu (a), **2**·ZnSal-ᵗBu (b), and **3**·ZnSal-ᵗBu (c), showing their different conformations. Displacement ellipsoids at 50% probability. Colour key: C (grey), N (lilac), Sb (light purple), Bi (dark purple), Zn (greyish blue), O (red), H (white). In the wireframe structures at left, H atoms are omitted for clarity. Space filling views of the capsules (a, b, c, right) show the encapsulation of the bridgehead atom in each case.*

| | $D_{Zn\text{-}Zn}$ (Å) | $D_{E\text{-}C_{py}}$ (Å) | $C_{py}$-E-$C_{py}$ (º) |
|---|---|---|---|
| **1**·ZnSal-$^{t}$Bu | 9.72 | 2.27 | 93.45 |
| **2**·ZnSal-$^{t}$Bu | 10.04 | 2.15 | 96.76 |
| **3**·ZnSal-$^{t}$Bu | 8.75 | 1.41 | 120.00 |

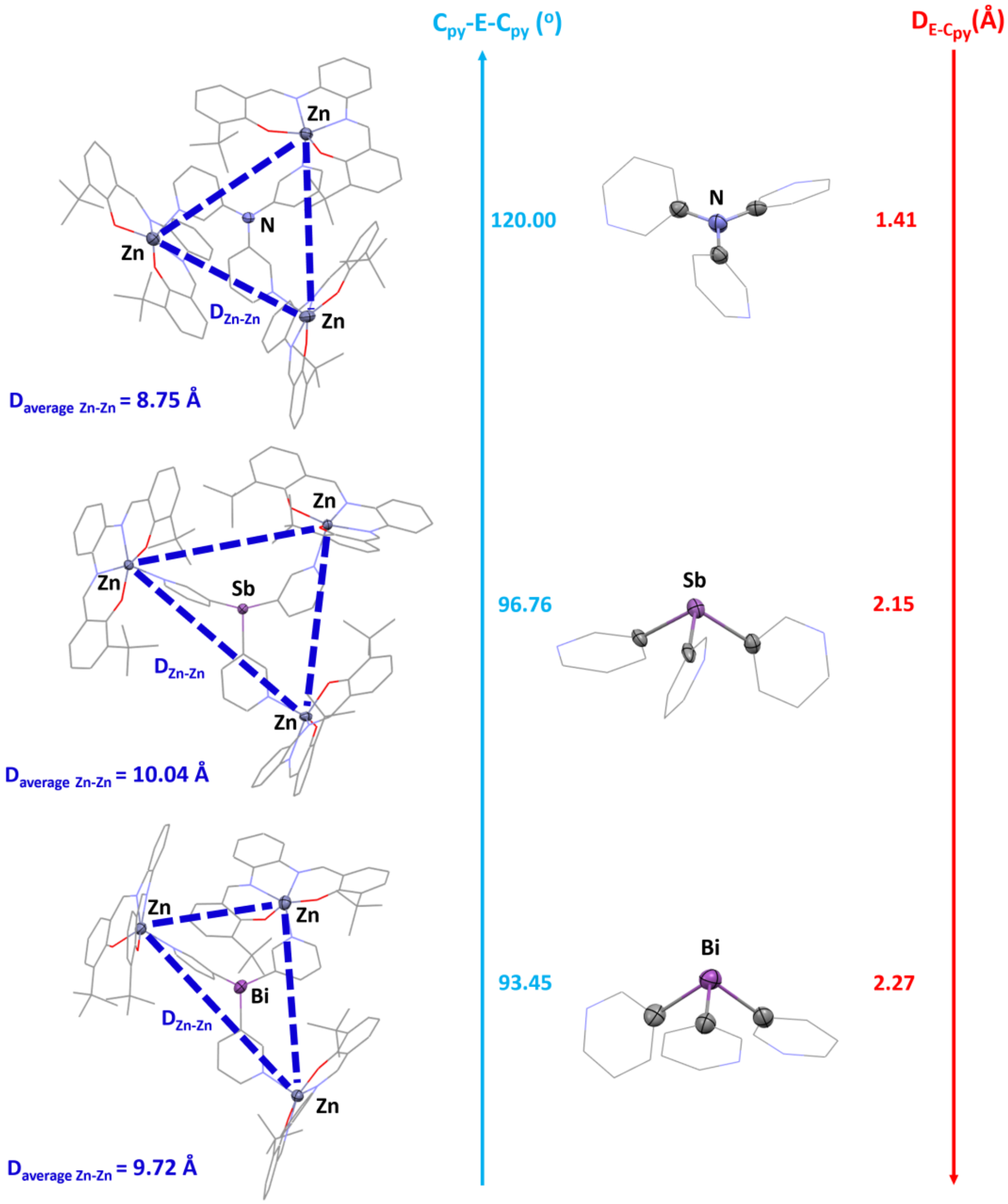


*Figure S88. Averages of selected bond length and angles in the family of **[E(3-py)$_3$·(ZnSal-$^{t}$Bu)$_3$]** capsules.*

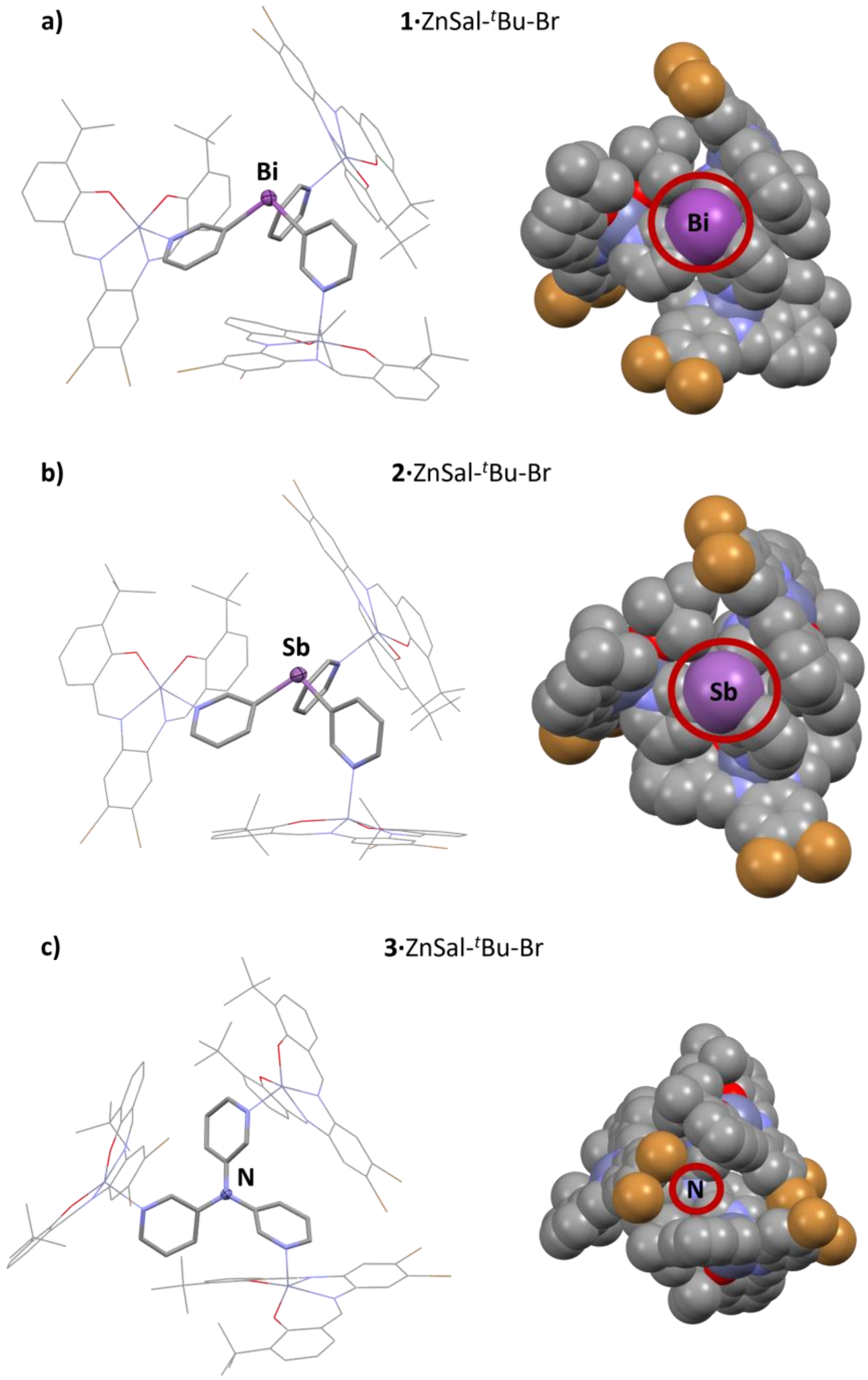


*Figure S89. X-ray structures of the family of molecular capsules **1**·ZnSal-$^t$Bu-Br (a), **2**·ZnSal-$^t$Bu-Br (b), and **3**·ZnSal-$^t$Bu-Br (c) showing their different conformations. Displacement ellipsoids at 50% probability. Colour key: C (grey), N (lilac), Sb (light purple), Bi (dark purple), Zn (greyish blue), O (red), Br (orange), H (white). In the wireframe structures at left, H atoms are omitted for clarity. Space filling views of the capsules (a, b, c, right) show the encapsulation of the bridgehead atom in each case.*

| | $D_{Zn-Zn}$ (Å) | $D_{E-C_{py}}$ (Å) | $C_{py}$-E-$C_{py}$ (º) |
|---|---|---|---|
| **1**·ZnSal-$^{t}$Bu-Br | 9.30 | 2.25 | 95.35 |
| **2**·ZnSal-$^{t}$Bu-Br | 9.34 | 2.14 | 96.51 |
| **3**·ZnSal-$^{t}$Bu-Br | 8.85 | 1.42 | 120.03 |

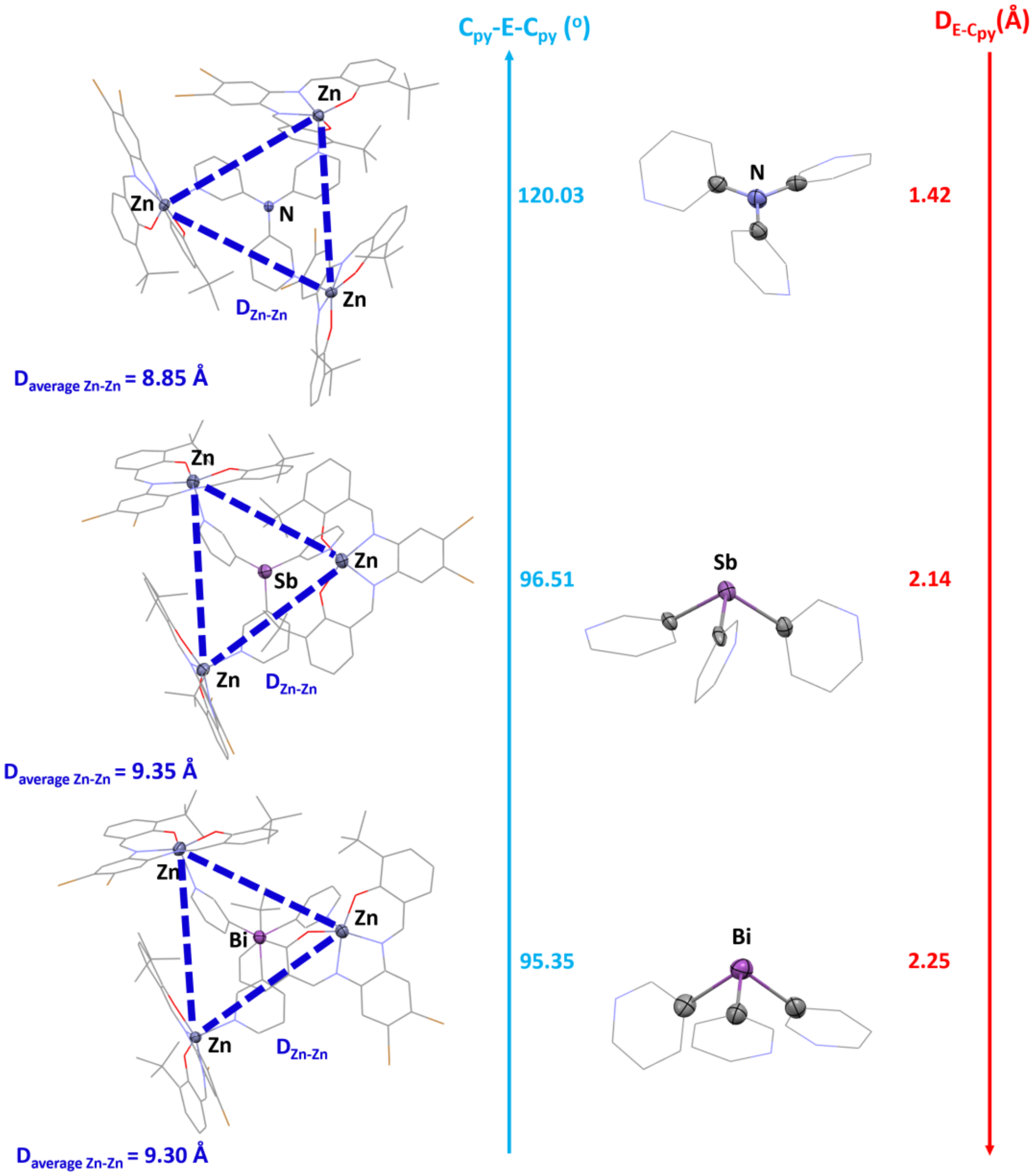


*Figure S90. Averages of selected bond length and angles in the family of **[E(3-py)$_3$·(ZnSal-$^{t}$Bu-Br)$_3$]** capsules.*

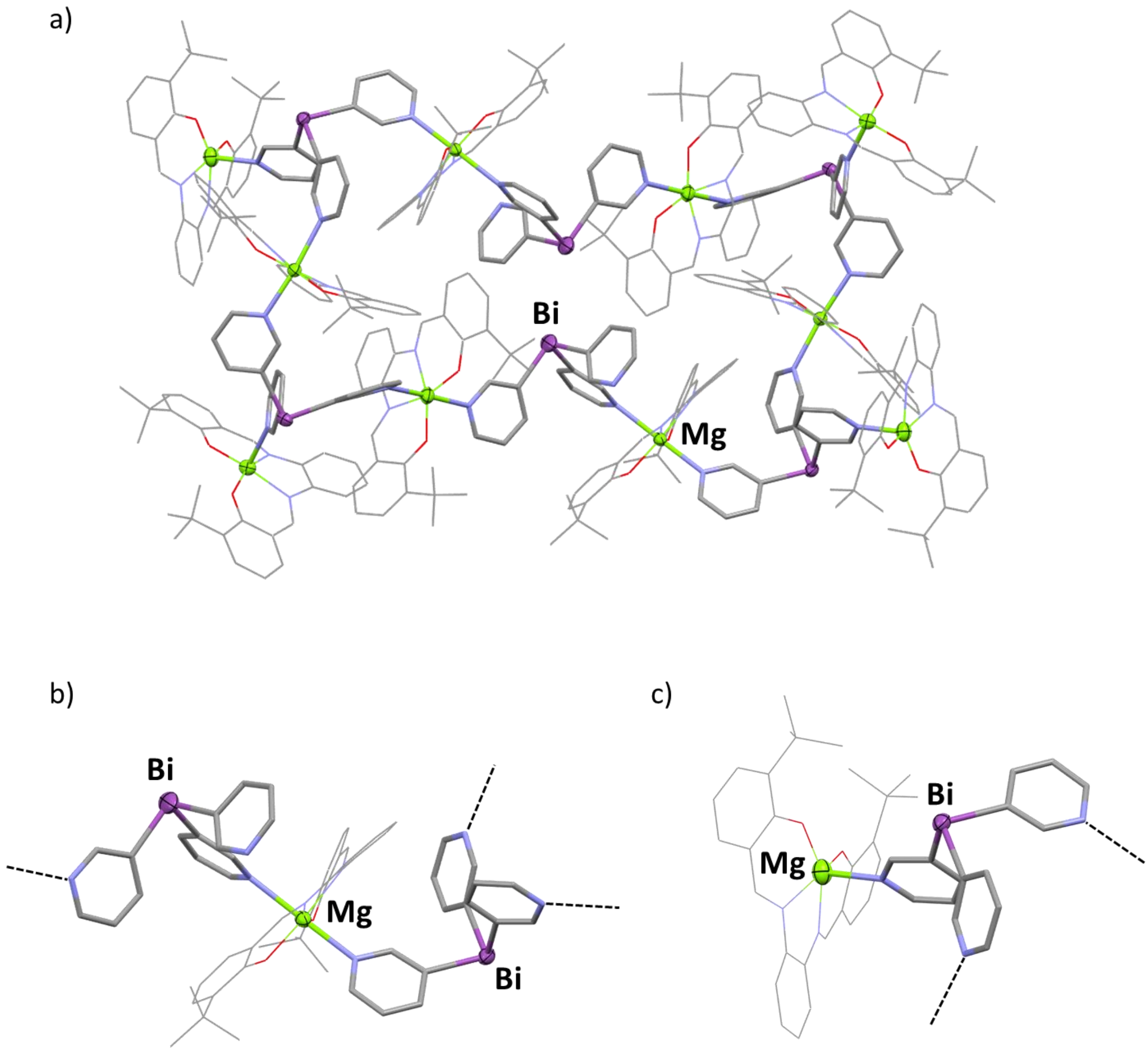


*Figure S91. a) X-ray structure of **1**·MgSal-$^t$Bu. Displacement ellipsoids at 50% probability. H atoms are omitted for clarity. Colour key: C (grey), N (lilac), Bi (dark purple), Mg (green), O (red). b) and c) Enlargements of the X-ray structure of **1**·MgSal-$^t$Bu showing Mg salphen units coordinated to the ligand at two (b) and at one (c) axial positions.*

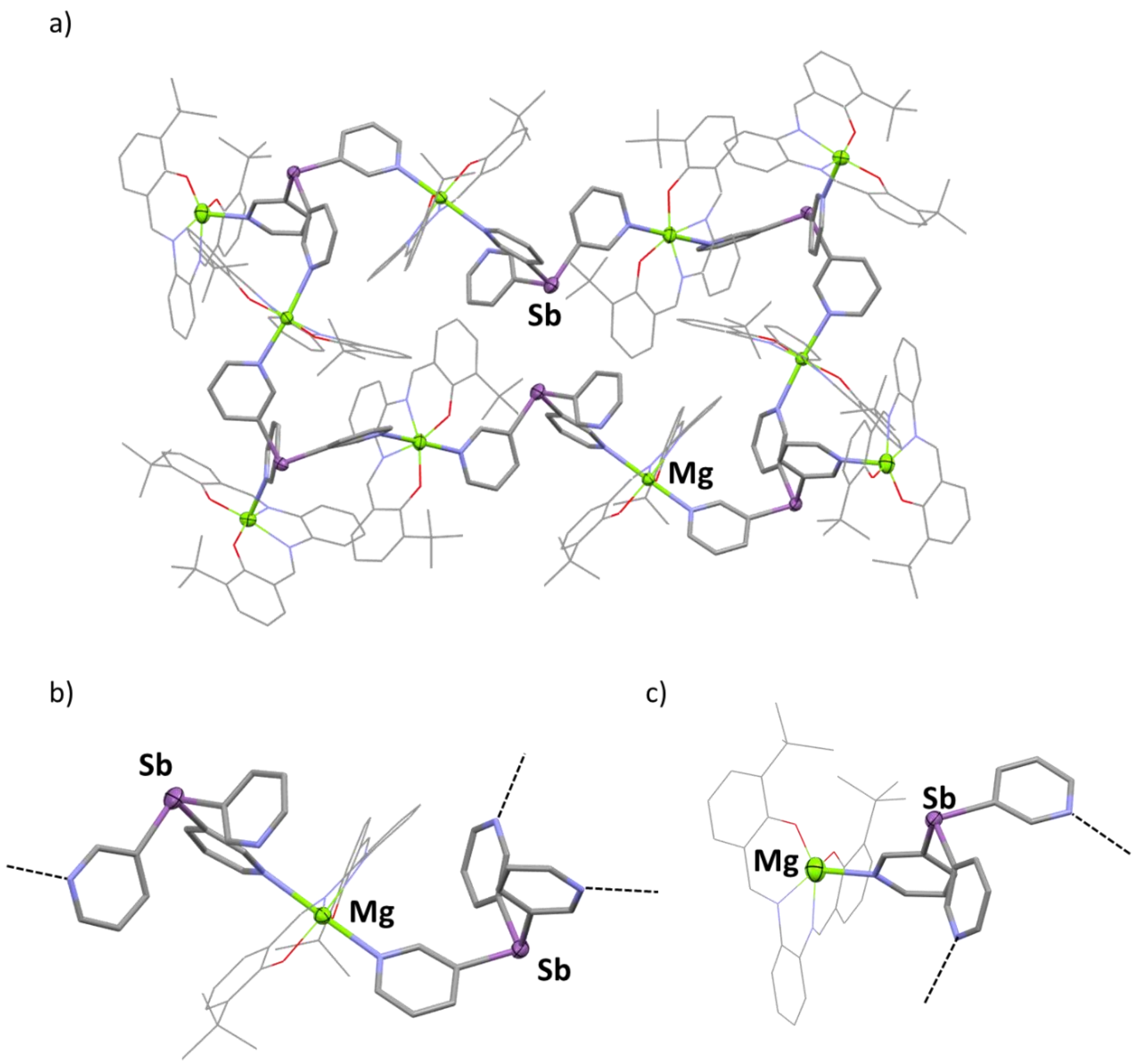


*Figure S92. a) X-ray structure of **2**·MgSal-$^t$Bu. Displacement ellipsoids at 50% probability. H atoms are omitted for clarity. Colour key: C (grey), N (lilac), Sb (light purple), Mg (green), O (red). b) and c) Enlargements of the X-ray structure of **2**·MgSal-$^t$Bu showing Mg salphen units coordinated to the ligand at two (b) and at one (c) axial positions.*

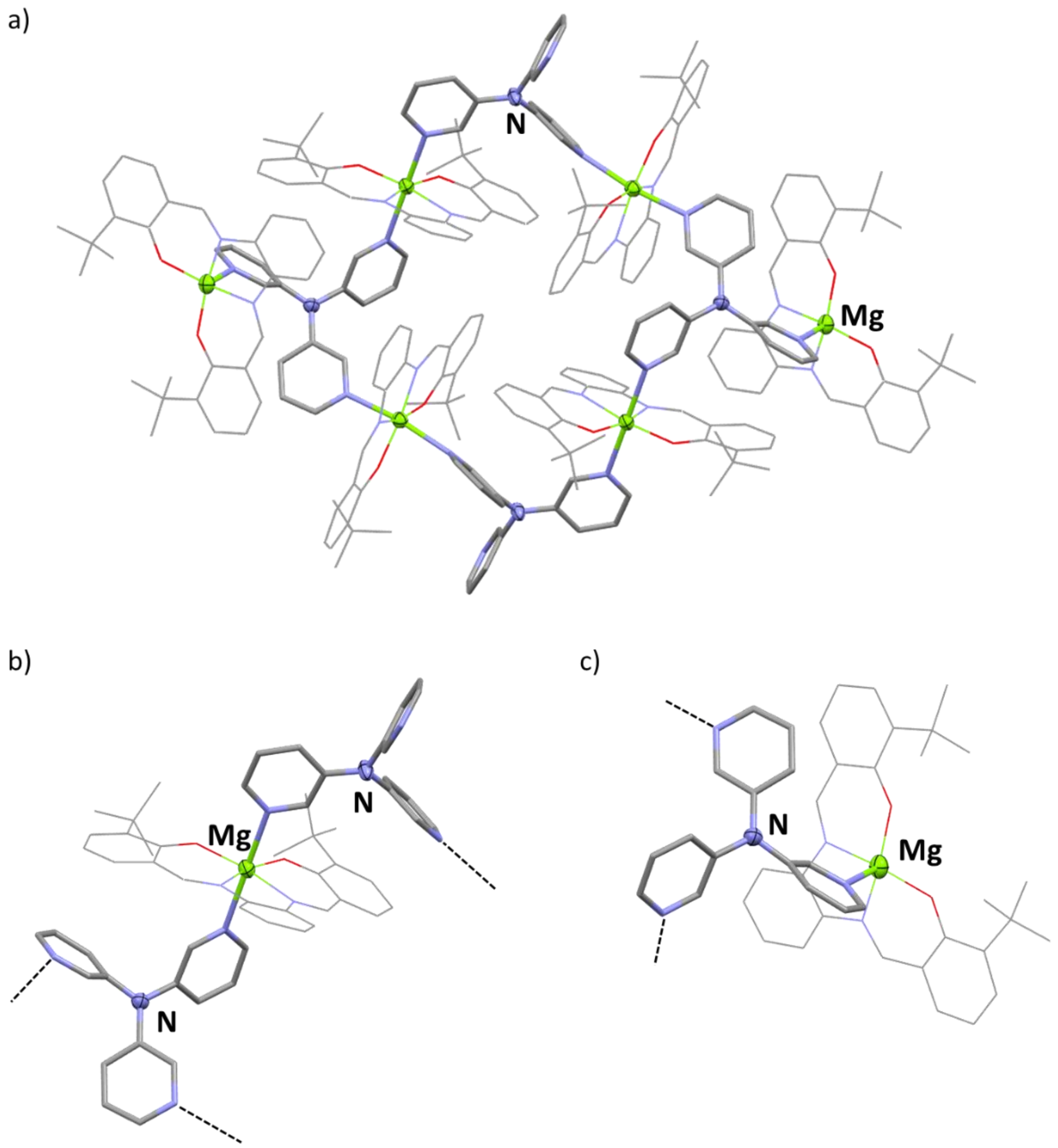


*Figure S93. a) X-Ray structure of **3**·MgSal-$^t$Bu. Displacement ellipsoids at 50% probability. H atoms are omitted for clarity. Colour key: C (grey), N (lilac), Mg (green), O (red). b) and c) Enlargements of the X-ray structure of **3**·MgSal-$^t$Bu showing the Mg salphen units coordinated to the ligand at two (b) and at one (c) axial positions.*

## X-Ray powder diffraction analysis (XRPD)

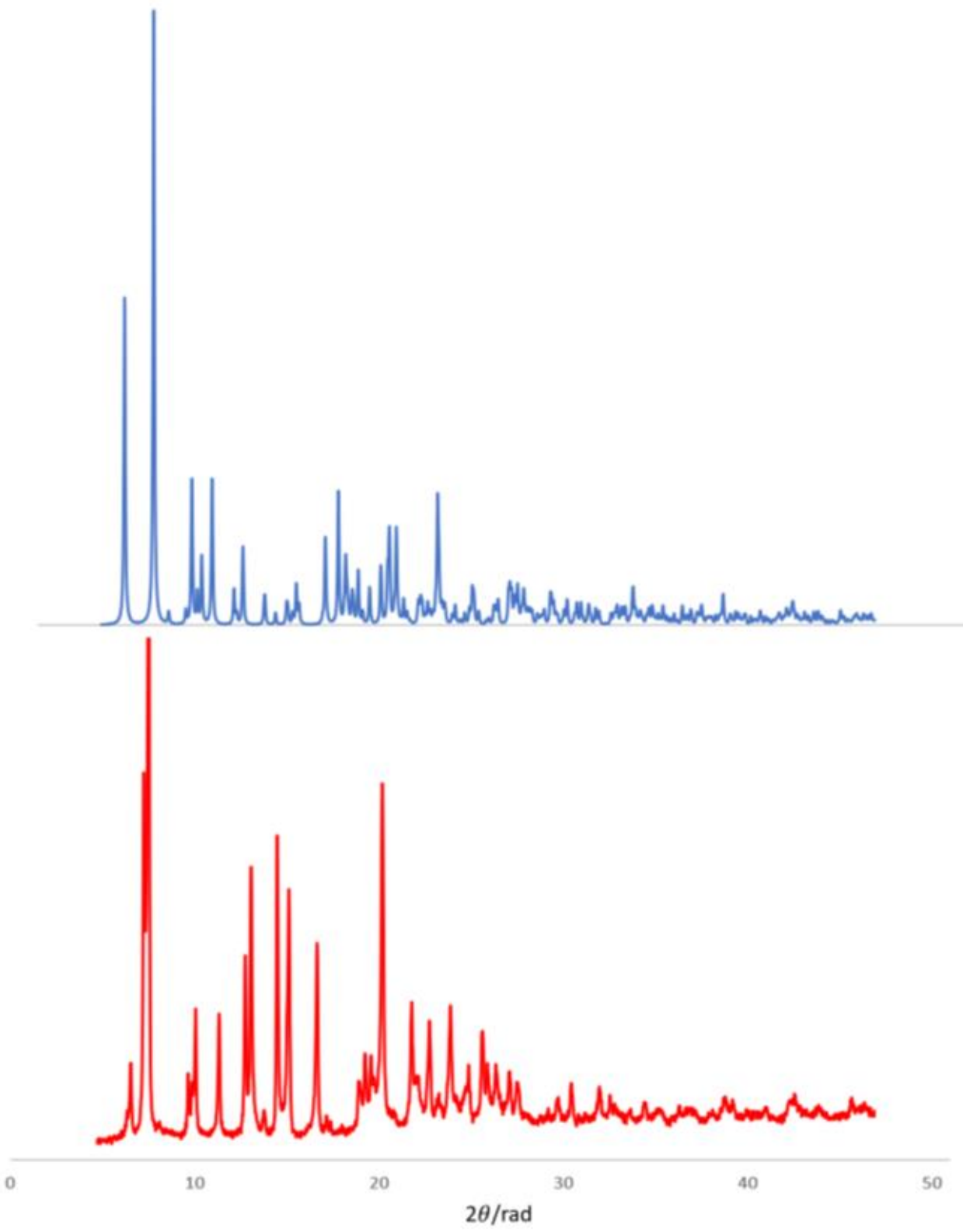


*Figure S94. X-Ray powder diffraction analysis of **1**·ZnSal, showing the predicted pattern (top, blue) and the experimental spectra (bottom, red). PXRD analysis indicated that the material remains crystalline but showed differences from the calculated pattern, suggesting some structural change upon prolonged exposure to vacuum. Nonetheless, $^{1}H$ NMR of the bulk crystalline sample in DMSO-$d_6$ is consistent with the compound identified by single-crystal X-ray diffraction.*

# Proof-of-concept catalytic studies

## General procedure for Bi-catalyzed oxidative cleavage of 1,2-diols

The reaction was carried out with two substrates, pinacol and benzopinacol, and the reaction conditions were adapted from previous studies.[11,12,13]

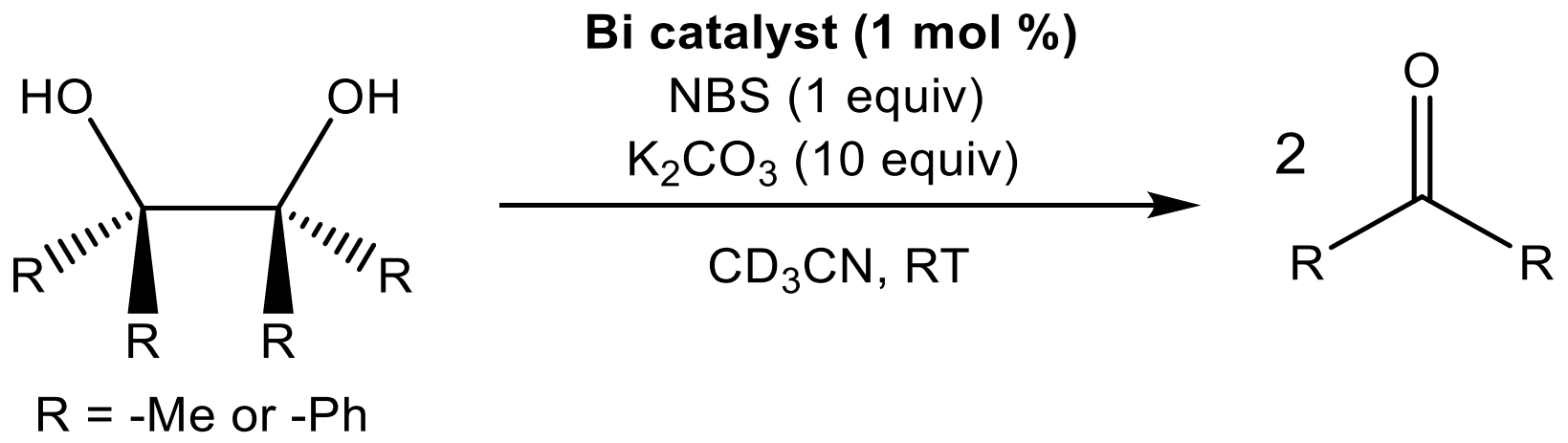


*Scheme S1. Bi-catalyzed oxidative cleavage of 1,2-diols (R = –Me or –Ph).*

In a vial, the corresponding 1,2-diol (0.14 mmol), 1,2-dibromoethane (0.28 mmol, internal reference), $K_2CO_3$ (1.4 mmol, 10 equiv.) and the Bi catalyst (**1** or **1**·ZnSal-$^t$Bu, 1 mol %) were dissolved in 1 mL of $CD_3CN$ and stirred for 5 minutes. To the resulting mixture, a solution of NBS (0.14 mmol, 1 equiv.) in 0.5 mL of $CD_3CN$ was added. The reaction was stirred at room temperature for the desired time. For competitive experiments, 0.07 mmol of each 1,2-diol was added instead of 0.14 mmol. The reaction was monitored using $^1$H NMR after 90 minutes.

Control experiments were performed under otherwise identical conditions using pinacol (0.14 mmol), 1,2-dibromoethane (0.28 mmol, internal standard), $K_2CO_3$ (1.4 mmol, 10 equiv.), and either **3** or **3·ZnSal-$^t$Bu** (1 mol%) in place of the Bi catalyst. After 90 min the oxidation product was observed in <5% yield.

## General procedure for Sb-catalyzed oxidations of α-hydroxyketones

The oxidation reaction of α-hydroxyketones with three substrates, acetoin, butyroin and benzoin, was studied. The reaction conditions were adapted from those previously described for the catalytic oxidation of these substrates by $SbPh_3$.[14]

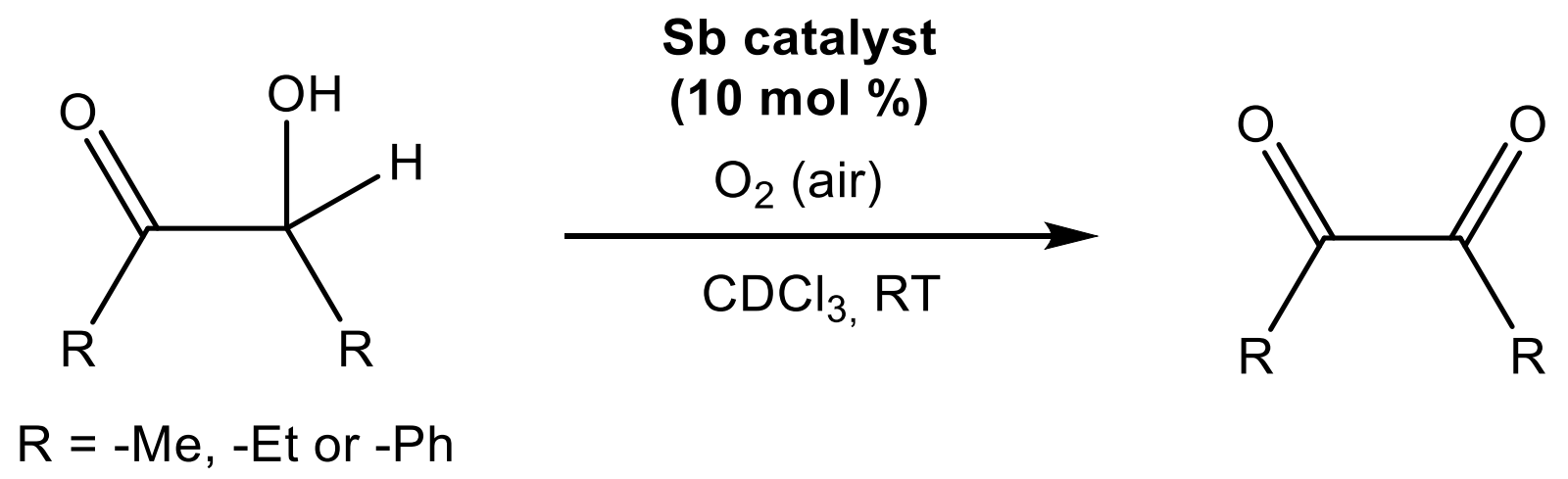


*Scheme S2. Sb-catalyzed oxidations of α-hydroxyketones (R = -Me, -Et or -Ph).*

In a J. Young NMR tube, the corresponding α-hydroxyketone (0.02 mmol), 1,2-dibromoethane (0.01 mmol, internal reference), and the Sb catalyst (**2** or **2**·ZnSal-$^t$Bu, 10 mol %) were dissolved in 1 mL of $CDCl_3$ under air. The reaction was monitored by $^1H$ NMR after 24 h. No product formation was observed under the same conditions in the absence of catalyst (with or without the presence of ZnSal-$^t$Bu). Similarly, no benzil formation was observed in the reaction of benzoin (PhCOCH(OH)Ph) under the same conditions when **3** or **3·ZnSal-$^t$Bu** (10 mol%) was used in place of the Sb catalyst.

## ON/OFF experiments

### *ON/OFF switching by pyridine-$d_5$ addition and removal*

In a J. Young NMR tube, benzoin (0.02 mmol) was dissolved in $CDCl_3$ (1 mL) under air using the catalytic conditions described above in the presence of the Sb capsule **2**·ZnSal-$^t$Bu (0.002 mmol, 10 mol %). After monitoring the reaction for 4 h, pyridine-$d_5$ (0.012 mmol) was added to the sample. Capsule disassembly was observed, leading to the onset of the catalytic reaction (Figure S95b, c). After another 4 h of monitoring the reaction progress, the pyridine-$d_5$ was removed by heating under vacuum, followed by dry $CH_3CN$ addition (5 × 5 mL) to promote its decoordination and evaporation of the volatiles. The sample was redissolved in 1 mL of $CDCl_3$ under air and the reaction progress was monitored again. Capsule reassembly was observed, and the reaction was again found to be supressed (Figure S95d, e).

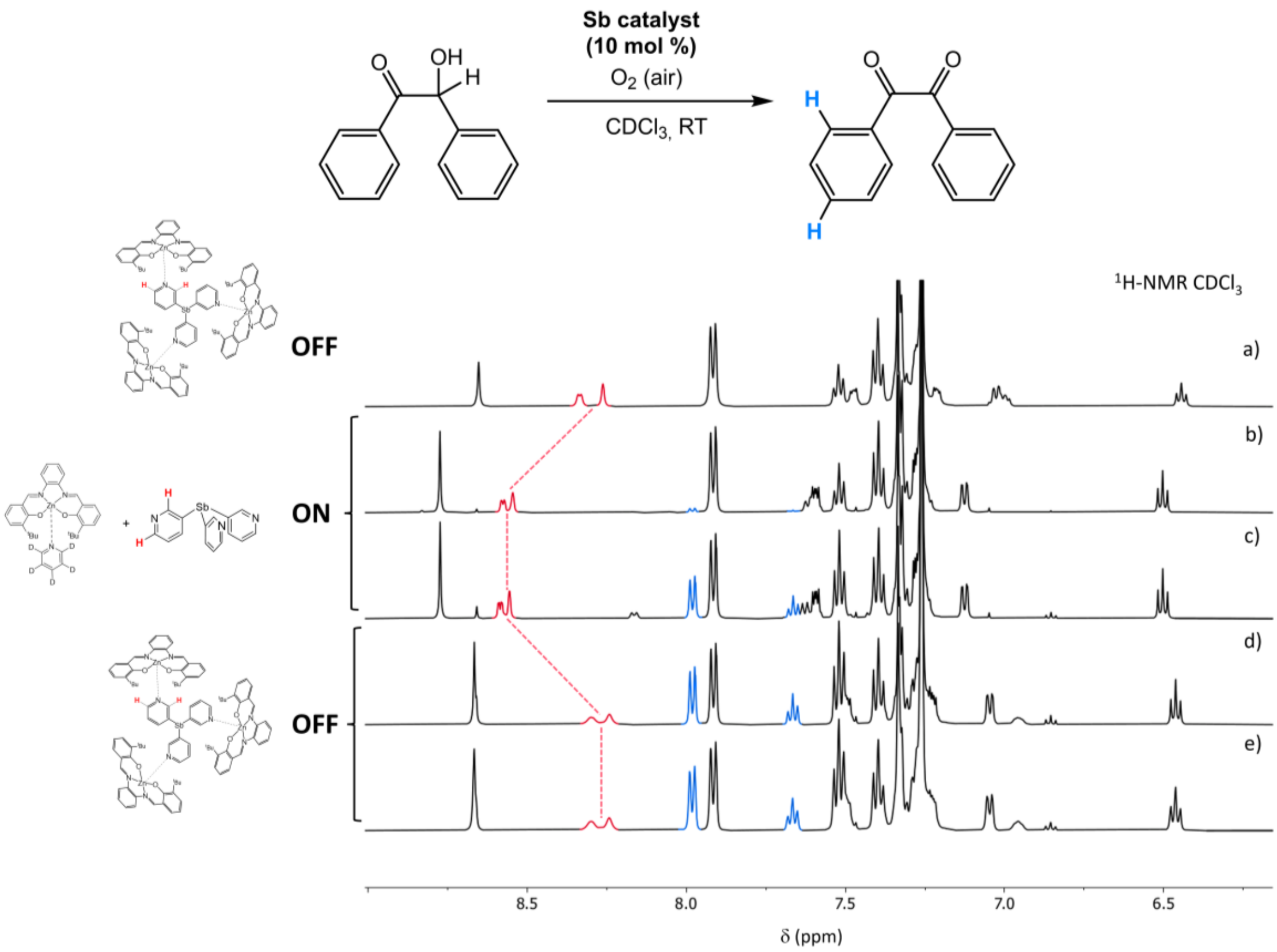


*Figure S95. Stacked $^1$H NMR spectra of the ON/OFF experiment in $CDCl_3$. a) 10 minutes after sample preparation, showing no detectable benzil product formation. Catalyst: **2**·ZnSal-$^t$Bu. b) 4 h after sample preparation and 10 minutes after the addition of py-$d_5$, showing the onset of benzil formation upon switching to the ON state. Catalyst: **2**. c) 8 h after sample preparation and 4 h after the addition of py-$d_5$. Catalyst: **2**. d) 10 h after sample preparation and 10 minutes after the removal of py-$d_5$. Catalyst: **2**·ZnSal-$^t$Bu. e) 14 h after sample preparation and 4 h after the removal of py-$d_5$, showing reaction suppression upon re-encapsulation of **2**. Catalyst: **2**·ZnSal-$^t$Bu.*

### *ON/OFF switching by sequential addition of pyridine-d5 and ZnSal-tBu*

In a J. Young NMR tube, benzoin (0.02 mmol) was dissolved in 1 mL $CDCl_3$ under air using the catalytic conditions described above in the presence of the Sb capsule **2**·ZnSal-tBu (0.002 mmol, 10 mol %). After 4h of monitoring the reaction, an excess of pyridine-d5 (0.012 mmol) was added to the sample. The disassembly of the capsule was observed, leading to the onset of the reaction (Figure S96b, c). After another 4h of monitoring the reaction progress, an excess of ZnSal-tBu (0.024 mmol) was added to the sample, which shifted the equilibrium toward capsule formation. As a result, capsule reassembly was observed and, consequently, the reaction was again observed to be supressed (Figure S96d, e, f).

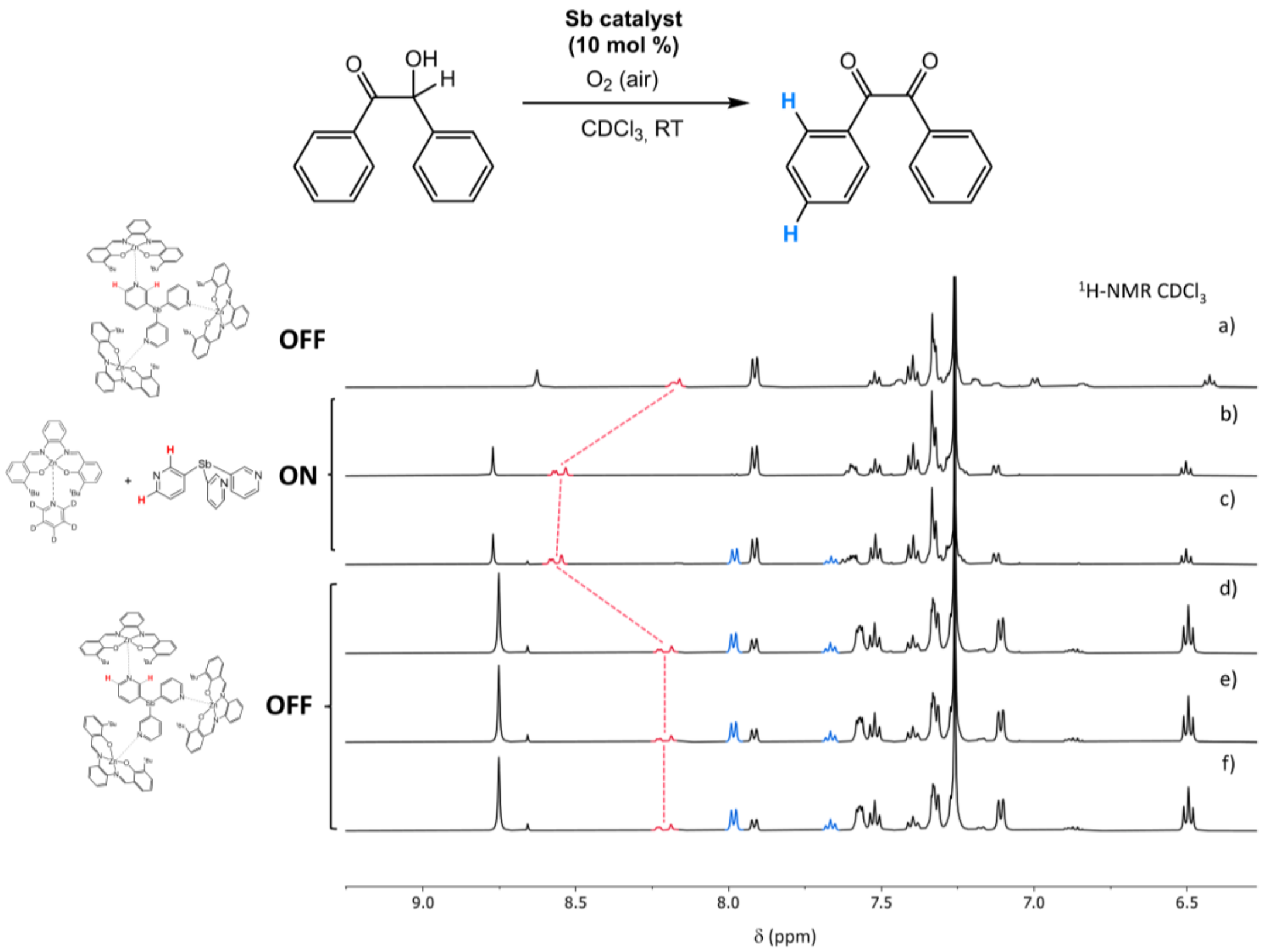


*Figure S96. Stacked ¹H NMR spectra of the ON/OFF experiment in $CDCl_3$. a) 10 minutes after sample preparation. Catalyst: **2**·ZnSal-tBu. b) 4 h after sample preparation and 10 minutes after the addition of py-d5. Catalyst: **2.** c) 8 h after sample preparation and 4 h after the addition of py-d5. Catalyst: **2**. d) 10 h after sample preparation and 10 minutes after the addition of ZnSal-tBu. Catalyst: **2**·ZnSal-tBu. e) 12 h after sample preparation and 2 h after the addition of ZnSal-tBu. Catalyst: **2**·ZnSal-tBu. f) 24 h after sample preparation and 14 h after the addition of ZnSal-tBu. Catalyst: **2**·ZnSal-tBu.*

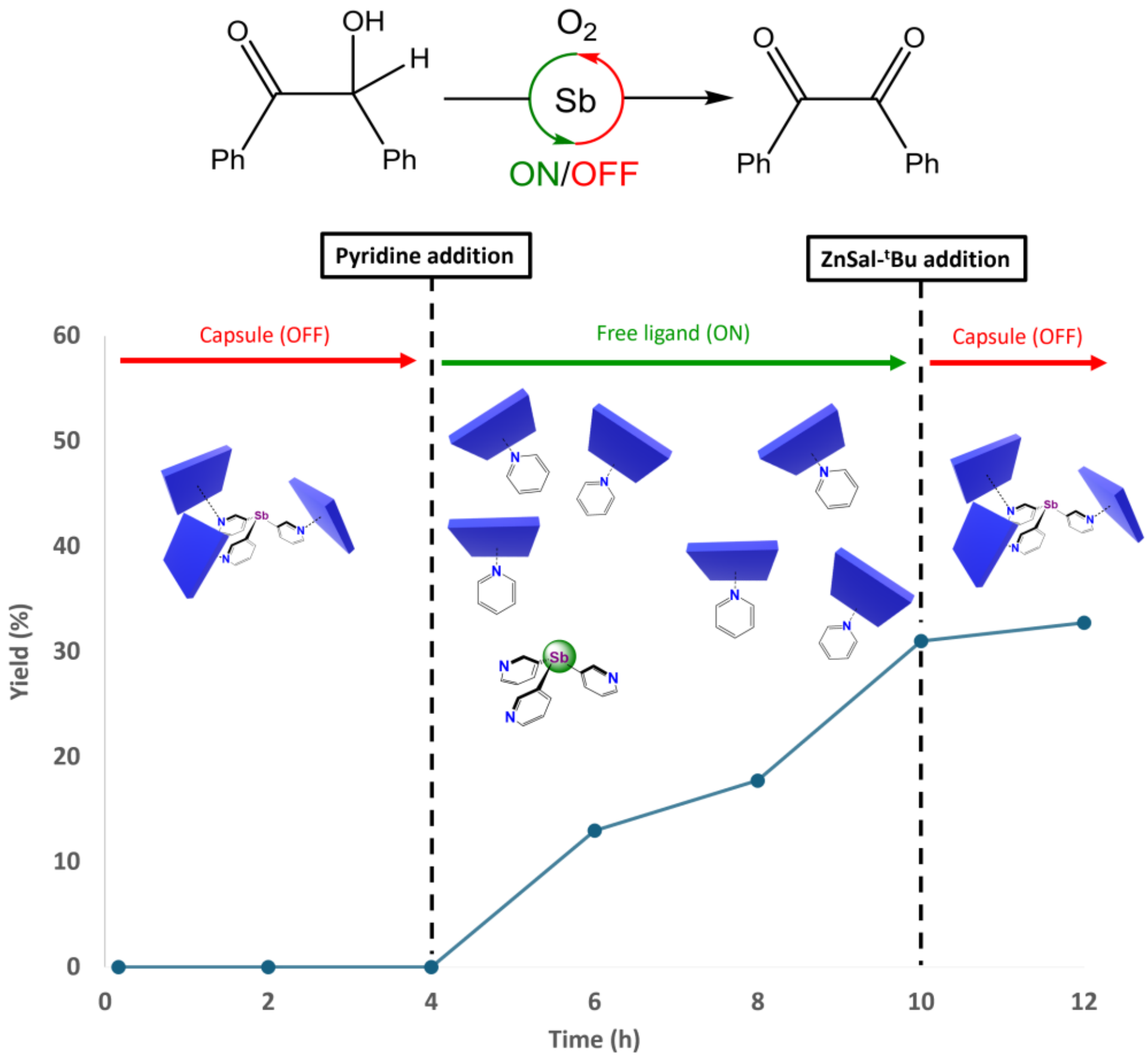


*Figure S97. Reversible ON/OFF control of Sb(3-py)$_3$-catalyzed aerobic oxidation of benzoin through pyridine-induced capsule disassembly and re-encapsulation by addition of ZnSal-$^t$Bu.*

### *OFF/ON switching by addition of acid*

Addition of acetic acid (AcOH, 2 equiv per ZnSal-$^{t}$Bu unit) resulted in an immediate shift of the pyridyl protons (Figure S98, blue signals), consistent with capsule disassembly through complete demetallation of the Zn–salphen units, as evidenced by the full conversion of ZnSal-$^{t}$Bu into 2H-Sal-$^{t}$Bu (Figure S98b, red signals).

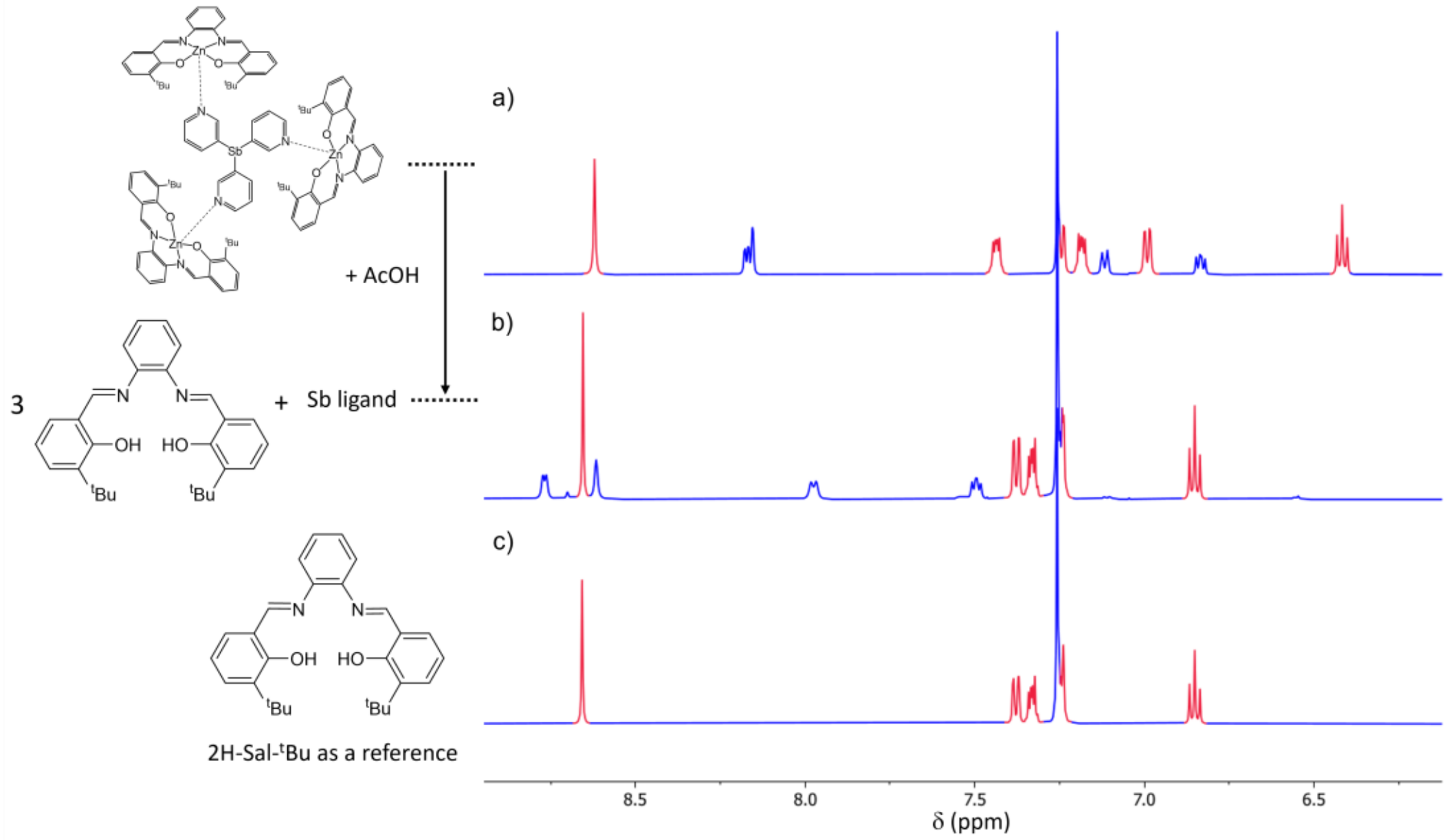


*Figure S98. Stacked $^{1}$H NMR spectra in $CDCl_3$ of the disassembly of **2**·ZnSal-$^{t}$Bu via demetallation of the salphen complex with formation of 2H-Sal-$^{t}$Bu. a) $^{1}$H NMR spectrum of **2**·ZnSal-$^{t}$Bu. Metallosalphen signals are highlighted in red. b) $^{1}$H NMR spectrum after addition of AcOH to **2**·ZnSal-$^{t}$Bu, showing disassembly of the capsule and demetallation of the salphen complex (signals of 2H-Sal-$^{t}$Bu highlighted in red). c) $^{1}$H NMR spectrum of 2H-Sal-$^{t}$Bu as a reference. Note: Following addition of AcOH, the signals of the pyridyl protons do not exactly match those of **2**, possibly reflecting changes in the medium, reversible protonation, and/or coordination to residual Zn(II). Nonetheless, subsequent addition of ZnSal-$^{t}$Bu promotes reassembly of the capsule-like assembly, making encapsulation of **2** reversible (see Fig. S99).*

### *OFF/ON switching by addition of acid under catalytic conditions*

In a J. Young NMR tube, benzoin (0.02 mmol) was dissolved in 1 mL $CDCl_3$ under air using the catalytic conditions described above in the presence of the Sb capsule **2**·ZnSal-$^{t}$Bu (0.002 mmol, 10 mol %). After 4 h of monitoring the reaction, an excess of AcOH (0.04 mmol) was added to the sample. The disassembly of the capsule was observed with concomitant onset of benzil formation upon switching to the ON state.

## *ON/OFF switching by sequential addition of AcOH and ZnSal-$^{t}$Bu*

After capsule disassembly via ZnSal-$^{t}$Bu demetallation with AcOH, two different possibilities were evaluated to reform **2**·ZnSal-$^{t}$Bu. While addition of excess of $NEt_3$ (3 equiv per 1 equiv AcOH) did not lead to the complete reassembly of **2**·ZnSal-$^{t}$Bu, addition of ZnSal-$^{t}$Bu led to the formation of the capsule again (Figure S99).

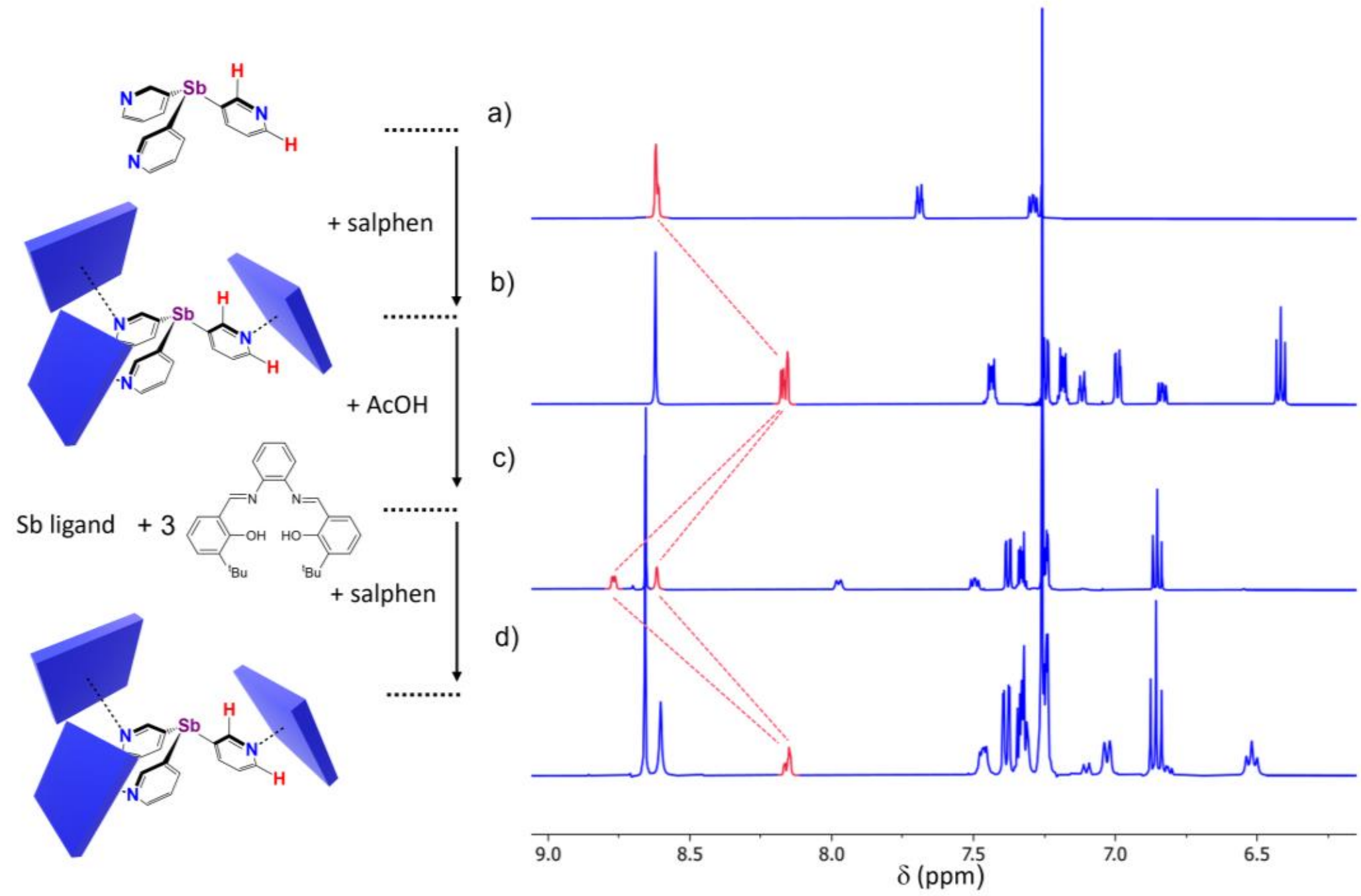


*Figure S99. Stacked $^{1}H$ NMR in $CDCl_3$ spectra with selected signals of the ligand **2** highlighted in red. a) Free ligand **2**. b) After the addition of three equivalents of ZnSal-$^{t}$Bu, showing capsule formation. c) After the addition of AcOH, showing capsule disassembly and release of **2** upon formation of 2H-Sal-$^{t}$Bu. d) After the addition of ZnSal-$^{t}$Bu, showing capsule reassembly.*

In a J. Young NMR tube, benzoin (0.02 mmol) was dissolved in 1 mL $CDCl_3$ under air using the catalytic conditions described above in the presence of the Sb capsule **2**·ZnSal-$^{t}$Bu (0.002 mmol, 10 mol %). After monitoring the reaction for 4 h, an excess of AcOH (0.035 mmol) was added to the sample. The disassembly of the capsule was observed, leading to the onset of the reaction (Figure S100b, c). After a total of 10 h of monitoring the reaction progress, an excess of ZnSal-$^{t}$Bu (0.041 mmol) was added to the sample. Capsule reassembly was observed, and the reaction was again found to be supressed (Figure S100d, e, f).

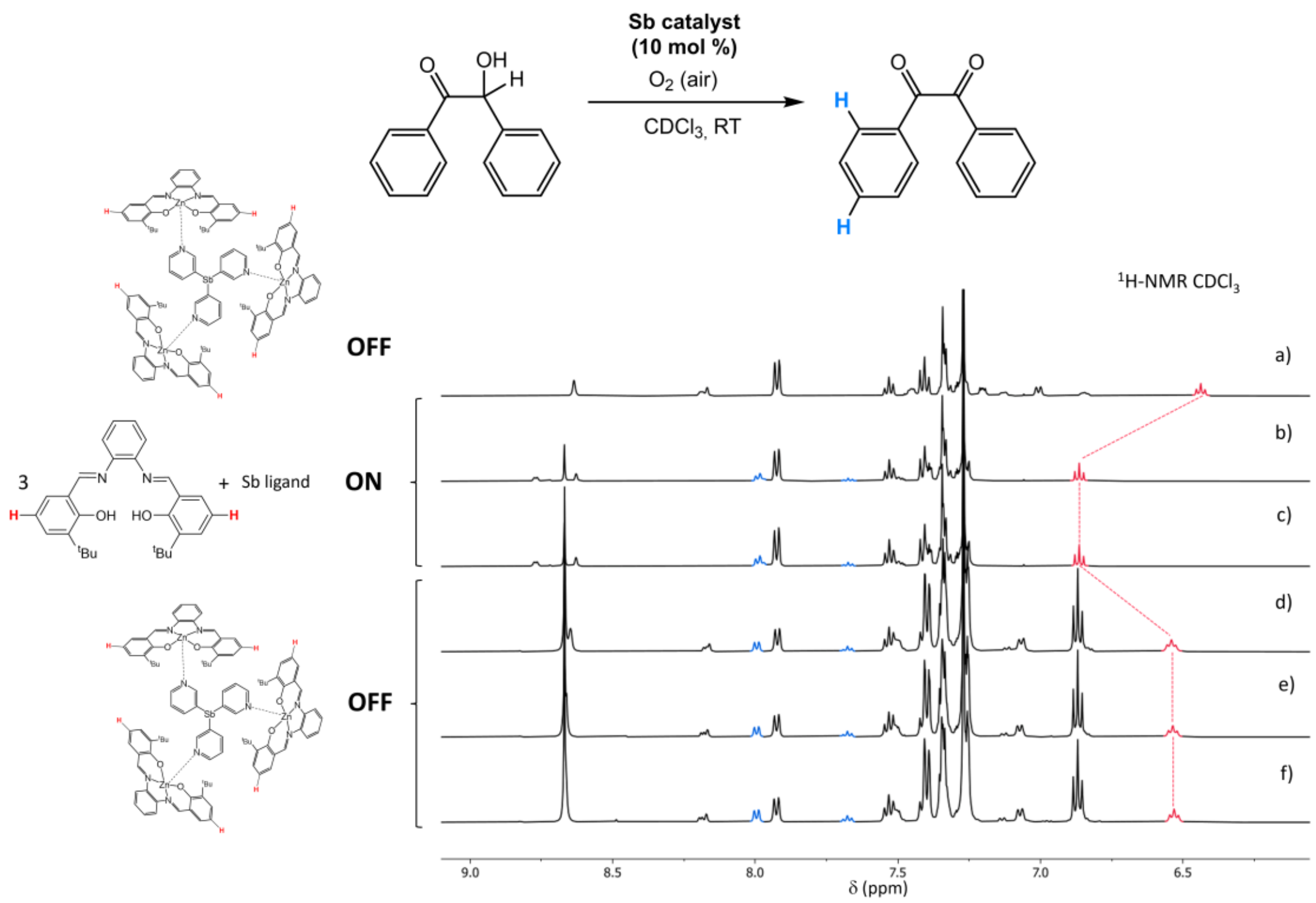


*Figure S100. Stacked $^{1}H$ NMR spectra of the ON/OFF experiment in $CDCl_3$. a) 10 minutes after sample preparation, showing no detectable benzil product formation. Catalyst: **2**·ZnSal-$^{t}$Bu. b) 6 h after sample preparation (2 h after the addition of AcOH), showing the onset of benzil formation upon switching to the ON state. Formation of 2H Sal-$^{t}$Bu is observed, indicating disassembly of **2**·ZnSal-$^{t}$Bu via demetallation. Catalyst: **2**. c) 8 h after sample preparation (4 h after the addition of AcOH). Catalyst: **2**. d) 10 h after sample preparation (10 minutes after the addition of ZnSal-$^{t}$Bu). Formation of **2**·ZnSal-$^{t}$Bu is observed, leading to reaction suppression. Catalyst: **2**·ZnSal-$^{t}$Bu. e) 13 h after sample preparation (3 h after the addition of ZnSal-$^{t}$Bu) showing reaction suppression upon re-encapsulation of **2** and formation of **2**·ZnSal-$^{t}$Bu Catalyst: **2**·ZnSal-tBu. f) 24 h after sample preparation (14 h after the addition of ZnSal-$^{t}$Bu).*

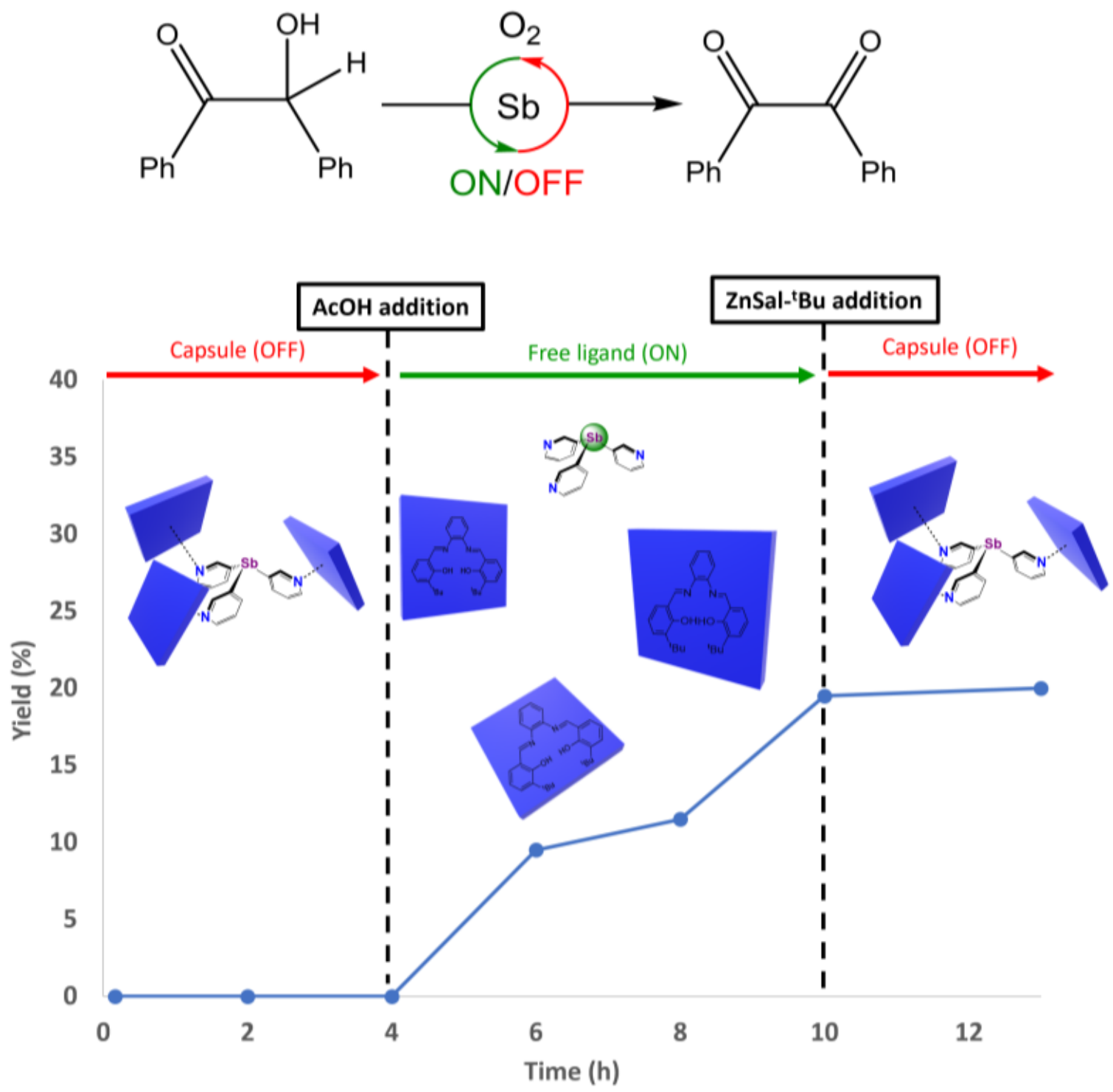


*Figure S101. Reversible ON/OFF control of Sb(3-py)$_3$-catalyzed aerobic oxidation of benzoin through acetic acid-induced capsule disassembly and re-encapsulation by addition of ZnSal-$^t$Bu.*

# References


(1) Neufeld, R.; Stalke, D. Accurate Molecular Weight Determination of Small Molecules via DOSY-NMR by Using External Calibration Curves with Normalized Diffusion Coefficients. *Chem. Sci.* **2015**, *6* (6), 3354–3364. https://doi.org/10.1039/c5sc00670h.

(2) Bachmann, S.; Gernert, B.; Stalke, D. Solution Structures of Alkali Metal Cyclopentadienides in THF Estimated by ECC-DOSY NMR-Spectroscopy (Incl. Software). *Chem. Commun.* **2016**, *52* (87), 12861–12864. https://doi.org/10.1039/c6cc07273a.

(3) Bachmann, S.; Neufeld, R.; Dzemski, M.; Stalke, D. New External Calibration Curves (ECCs) for the Estimation of Molecular Weights in Various Common NMR Solvents. *Chem. - A Eur. J.* **2016**, *22* (25), 8462–8465. https://doi.org/10.1002/chem.201601145.

(4) Thordarson, P. Determining Association Constants from Titration Experiments in Supramolecular Chemistry. *Chem. Soc. Rev.* **2011**, *40* (3), 1305–1323. https://doi.org/10.1039/c0cs00062k.

(5) Thordarson, P. Binding Constants and Their Measurement. In *Supramolecular Chemistry*; Wiley, 2012. https://doi.org/https://doi.org/10.1002/9780470661345.smc018.

(6) Pramanik, S.; Thordarson, P.; Day, V. W.; Bowman-James, K. Oligomeric Phosphate Clusters in Macrocyclic Channels. *CrystEngComm* **2022**, *24* (46), 8047–8051. https://doi.org/10.1039/D2CE00756H.

(7) Álvarez-Llorente, N.; Stasyuk, A. J.; Diez-Varga, A.; Ferrero, S.; Solà, M.; Barbero, H.; Álvarez, C. M. Multitopic Corannulene-Porphyrin Hosts for Fullerenes: A Three-Layer Scaffold for Precisely Designed Supramolecular Ensembles. *Org. Lett.* **2025**, *27* (1), 357–362. https://doi.org/10.1021/acs.orglett.4c04385.

(8) Brynn Hibbert, D.; Thordarson, P. The Death of the Job Plot, Transparency, Open Science and Online Tools, Uncertainty Estimation Methods and Other Developments in Supramolecular Chemistry Data Analysis. *Chem. Commun.* **2016**, *52* (87), 12792–12805. https://doi.org/10.1039/c6cc03888c.

(9) Hu, W.; Xie, J.; Chau, H. W.; Si, B. C. Evaluation of Parameter Uncertainties in Nonlinear Regression Using Microsoft Excel Spreadsheet. *Environ. Syst. Res.* **2015**, *4* (1), 4. https://doi.org/10.1186/s40068-015-0031-4.

(10) Mantina, M.; Chamberlin, A. C.; Valero, R.; Cramer, C. J.; Truhlar, D. G. Consistent van Der Waals Radü for the Whole Main Group. *J. Phys. Chem. A* **2009**, *113* (19), 5806–5812. https://doi.org/10.1021/jp8111556.

(11) Barton, D. H. R.; Motherwell, W. B.; Stobie, A. A Catalytic Method for α-Glycol Cleavage. *J. Chem. Soc. Chem. Commun.* **1981**, No. 23, 1232–1233. https://doi.org/10.1039/C39810001232.

(12) Barton, D. H. R.; Finet, J.-P.; Motherwell, W. B.; Pichon, C. The Chemistry of Pentavalent Organobismuth Reagents: Part IX. Cleavage Reactions of α-Glycols. J. Chem. Soc. Perkin Trans. I, in Press. *Tetrahedron* **1986**, *42* (20), 5627–5636. https://doi.org/https://doi.org/10.1016/S0040-4020(01)88167-9.

(13) Magre, M.; Kuziola, J.; Nöthling, N.; Cornella, J. Dibismuthanes in Catalysis: From Synthesis and Characterization to Redox Behavior towards Oxidative Cleavage of 1,2-

Diols. *Org. Biomol. Chem.* **2021**, *19* (22), 4922–4929. https://doi.org/10.1039/d1ob00367d.

(14) Yasuike, S.; Kishi, Y.; Kawara, S.-I.; Kurita, J. Catalytic Action of Triarylstibanes: Oxidation of Benzoins into Benzyls Using Triarylstibanes under an Aerobic Condition. *Chem. Pharm. Bull. (Tokyo).* **2005**, *53* (4), 425–427. https://doi.org/10.1248/cpb.53.425.
